# Bimolecular Sandwich Aggregates of Porphyrin Nanorings

**DOI:** 10.1021/jacs.4c09267

**Published:** 2024-08-26

**Authors:** Henrik Gotfredsen, Janko Hergenhahn, Fernanda Duarte, Timothy D. W. Claridge, Harry L. Anderson

**Affiliations:** Department of Chemistry, University of Oxford, Chemistry Research Laboratory, Oxford, OX1 3TA, U.K.

## Abstract

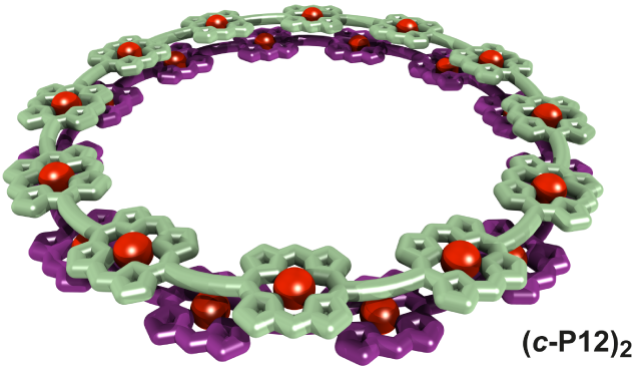

Extended π-systems often form supramolecular aggregates, drastically changing their optical and electronic properties. However, aggregation processes can be difficult to characterize or predict. Here, we show that butadiyne-linked 8- and 12-porphyrin nanorings form stable and well-defined bimolecular aggregates with remarkably sharp NMR spectra, despite their dynamic structures and high molecular weights (12.7 to 26.0 kDa). Pyridine breaks up the aggregates into their constituent rings, which are in slow exchange with the aggregates on the NMR time scale. All the aggregates have the same general two-layer sandwich structure, as deduced from NMR spectroscopy experiments, including ^1^H DOSY, ^1^H–^1^H COSY, TOCSY, NOESY, and ^1^H–^13^C HSQC. This structure was confirmed by analysis of residual dipolar couplings from ^13^C-coupled ^1^H–^13^C HSQC experiments on one of the 12-ring aggregates. Variable-temperature NMR spectroscopy revealed an internal ring-on-ring rotation process by which two π–π stacked conformers interconvert via a staggered conformation. A slower dynamic process, involving rotation of individual porphyrin units, was also detected by exchange spectroscopy in the 8-ring aggregates, implying partial disaggregation and reassociation. Molecular dynamics simulations indicate that the 8-ring aggregates are bowl-shaped and highly fluxional, compared to the 12-ring aggregates, which are cylindrical. This work demonstrates that large π-systems can form surprisingly well-defined aggregates and may inspire the design of other noncovalent assemblies.

## Introduction

The formation of noncovalent multimolecular aggregates is important for the function and properties of proteins,^1−3^ polymers,^4−6^ and small molecules, particularly dyes.^7−11^ Nature exploits and controls the strong tendency of porphyrins to aggregate to achieve precise arrangements of chlorophyll units in photosynthetic machinery and antenna complexes, such as the special pair, chlorosomes, and light-harvesting complexes.^12−14^ The performance of organic electronic devices, such as solar cells, light-emitting diodes, and transistors, depends greatly on the aggregated state (morphology) of semiconductors, and it directly impacts their charge transport properties.^15−18^ Aggregation can give rise to fascinating and useful photophysical phenomena, such as singlet fission^19^ and aggregation-induced emission.^20−22^ Yet, despite the far-reaching implications of aggregation, our ability to control or predict it is still very limited. This is not surprising since molecular aggregates can be inherently difficult to study, in part because they are often not well-defined (i.e., not ascribable to a single structure) and in part because they may not be amenable to structural characterization techniques such as NMR spectroscopy or X-ray crystallography. Too often, any unexpected or puzzling behavior by an extended molecular π-system is attributed to “aggregation”.

Polycyclic aromatic hydrocarbons (PAHs) constitute one class of compounds with a high propensity for aggregation and formation of infinite one-dimensional stacks. It is difficult to gain structural information on infinite or polymeric stacks because they are generally unsuitable for solution-phase NMR spectroscopy or single-crystal X-ray crystallography. Techniques such as 3D electron tomography and electron diffraction only rarely provide detailed information on the molecular arrangement in infinite stacks.^23−25^ In general, the guiding principles of aggregation can be learned from examples of smaller fragments that give rise to stable and discrete aggregates.^26,27^ For example, stacked dimers of PAHs have been achieved through the use of bulky side chains, which prevent further aggregation by shielding the outer π-surfaces.^28,29^ Larger discrete stacks of PAHs have been obtained through backbone engineering which can serve to direct aggregation toward compact structures in order to maximize the number of stabilizing aromatic interactions.^28,30−33^ Large PAH derivatives^32^ and metal cages^34−36^ have been used to host discrete aggregates of small π-rich substrates, also by operating on the premise of suppressed aggregation through steric shielding. Recently, the power of shape-complementarity was illustrated by the assembly of discrete twisted bimolecular helices of twisted PAH ribbons.^37^

Porphyrins often tend to aggregate, and this tendency is enhanced in covalent porphyrin oligomers. Bulky *meso*-substituents are frequently used to suppress aggregation and ensure solubility. We have previously identified the formation of a discrete trimolecular aggregate consisting of linear butadiyne-linked porphyrin oligomers, which is highly sensitive to the choice of substituent.^38^ Cyclic porphyrin oligomers might be expected to behave similarly, although the stacking of rings is accompanied by an energy penalty associated with planarization. Our earlier studies using STM revealed a tendency for large nanorings (consisting of 24–50 porphyrin units)^39,40^ to form stacks of two or three rings on surfaces, while images of smaller rings (consisting of 10–20 units)^40,41^ only showed isolated molecules. The presence of pyridine in the solutions used to deposit nanorings onto the surface suppressed aggregation,^39,40^ which indicates that stacks form in solution prior to surface-deposition, but attempts at characterizing the solution-phase aggregates of these large nanorings were unsuccessful.

**Figure 1:**
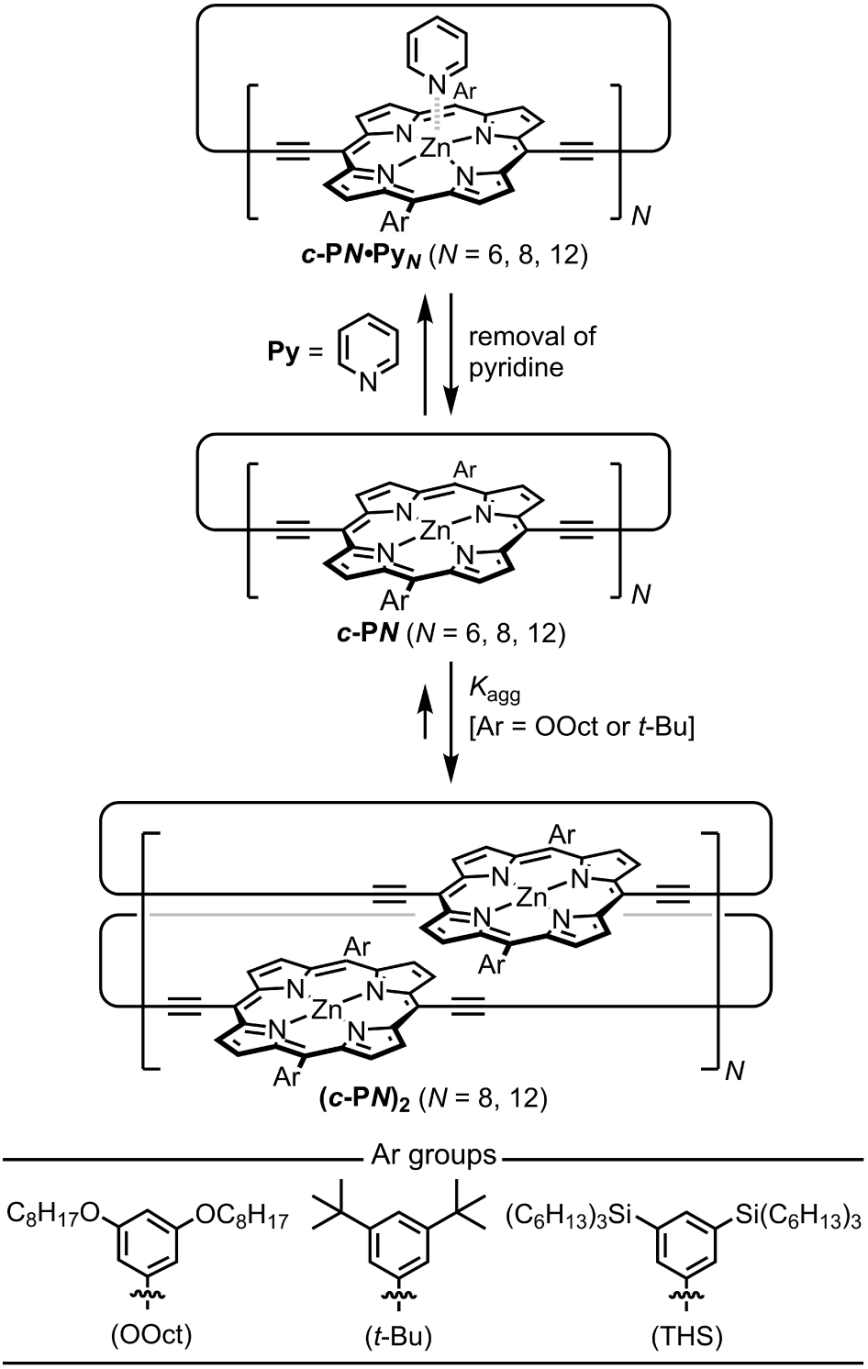
Removal of pyridine from porphyrin nanorings (***c*-P*N***) leads to self-assembly into discrete dimer ring aggregates if the ring size (*N* = 8 or 12, rather than 6) and side chain type are right (OOct or *t*-Bu, rather than THS). Ar groups correspond to 3,5-bis(octyloxy)phenyl (OOct), 3,5-bis(*tert*-butyl)phenyl (*t*-Bu), and 3,5-bis(trihexylsilyl)phenyl (THS).

Here, we present evidence of the formation of stable bimolecular aggregates consisting of nanorings of 8 and 12 porphyrin units. The aggregates exhibit sharp NMR spectra and are formed in solution in noncoordinating solvents such as chloroform, and aggregation is strongly suppressed by ligands such as pyridine. The structure of the aggregates was elucidated by combining ^1^H DOSY, ^1^H–^1^H COSY, TOCSY, NOESY, and ^1^H–^13^C NMR spectroscopy techniques. The DOSY spectra indicate that the aggregates are bimolecular. Insights into dynamics were provided by variable-temperature NMR, ^1^H–^1^H EXSY spectroscopy, and molecular dynamics (MD). Further support for the structure was gained through the analysis of residual dipolar couplings (RDCs) obtained by carbon-coupled HSQC spectroscopy in the case of the 12-ring aggregate. In this work, we have considered porphyrin nanorings, ***c*-P*N***, of different sizes (*N* = 6, 8, and 12) and with different side chains, including 3,5-bis(*tert*-butyl)phenyl (*t*-Bu), 3,5-bis(octyloxy)phenyl (OOct), and 3,5-bis(trihexylsilyl)phenyl (THS), as shown in Figure 1. In the absence of coordinating ligands such as pyridine, 8- and 12 porphyrin nanorings with *t*-Bu or OOct side chains form bimolecular aggregates. In contrast, smaller rings (e.g., *N* = 6), or all rings with THS side chains (***c*-P*N*_THS_**; *N* = 6, 8, or 12), do not aggregate under these conditions.

## Results and Discussion

### Absorption and Emission Spectroscopy

**Figure 2:**
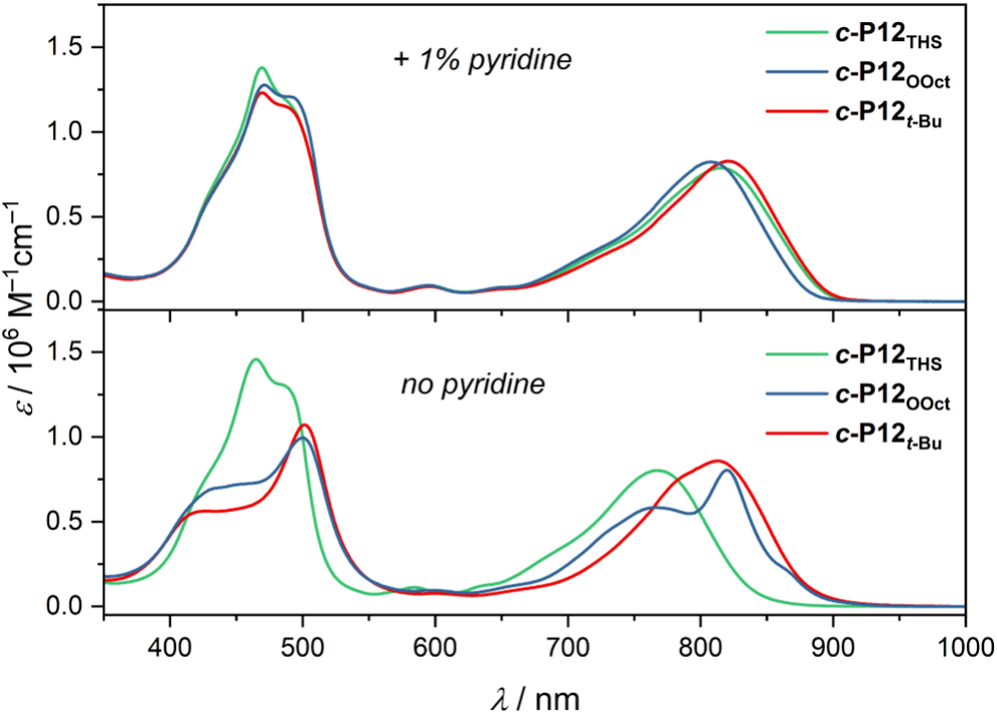
Absorption spectra of 12-porphyrin nanorings ***c*-P12_THS_**, ***c*-P12_OOct_**, and ***c*-P12*_t_*_-Bu_** in the absence and presence of pyridine (1% by volume of solvent) recorded in CDCl_3_ at 25 °C (concentration ca. 1 μM).

The absorption spectra of ***c*-P12_THS_**, ***c*-P12_OOct_**, and ***c*-P12*_t_*_-Bu_** in chloroform in the absence and presence of pyridine are shown in Figure 2. The bulky THS groups make ***c*-P12_THS_** incapable of aggregating (as confirmed by ^1^H NMR spectroscopy). When ***c*-P12_THS_** binds pyridine, the Q absorption band shifts to longer wavelengths by 48 nm, but the shape of the absorption spectrum remains essentially unchanged. The absorption spectra of ***c*-P12_OOct_** and ***c*-P12*_t_*_-Bu_** in the presence of pyridine are essentially identical to that of ***c*-P12_THS_**, implying that none of the rings aggregate under these conditions. On the other hand, in the absence of pyridine, the spectra of ***c*-P12_OOct_** and ***c*-P12*_t_*_-Bu_** feature a split Soret band alongside a Q-band that has been shifted bathochromically by 50 nm relative to ***c*-P12_THS_**. The split Soret band of ***c*-P12_OOct_** and ***c*-P12*_t_*_-Bu_** resembles those of previously studied aggregates^38^ and ladder complexes^42,43^ in which the butadiyne-linked porphyrin units are held in a coplanar arrangement. This implies some degree of torsional rigidification, restricting the porphyrin rotation about the butadiyne links. The difference in absorption in the Soret band region is less stark in the 8-porphyrin nanorings, which points to a higher degree of flexibility and conformational disorder (Supporting Information, Section 15). The absorption spectra of ***c*-P12_OOct_** and ***c*-P12*_t_*_-Bu_** in the absence of pyridine show no dependence on concentration within the range 10^–6^ to 10^–9^ M, implying that the aggregation constant (*K*_agg_) for these two rings is larger than 10^8^ M^–1^ (Supporting Information, Section 15). On the other hand, aggregation of 8-porphyrin nanorings, ***c*-P8_OOct_** and ***c*-P8*_t_*_-Bu_**, are sufficiently weak to exhibit a dependence on concentration, which enabled us to estimate their aggregation constants as ca. 5.0 × 10^7^ M^–1^ and 8.6 × 10^6^ M^–1^, respectively, in CDCl_3_ at 298 K (Supporting Information, Section 15). The fluorescence quantum yields of ***c*-P12_OOct_** (Φ_F_ = 0.02) and ***c*-P12*_t_*_-Bu_** (Φ_F_ = 0.002) increase substantially on addition of pyridine (Φ_F_ = 0.16 for ***c*-P12_OOct_** and Φ_F_ = 0.11 for ***c*-P12*_t_*_-Bu_**), corresponding to a quenching efficiency of ca. 88% and 98%, respectively, in the aggregates. In contrast, the fluorescence quantum yield of ***c*-P12_THS_** (Φ_F_ = 0.26) decreases in the presence of pyridine (Φ_F_ = 0.15). To gain further insight into the strength and cooperativity of aggregation, we performed titrations using pyridine to break up the aggregates. The extent of disaggregation was followed via the change in the Soret band, and the data were fitted to a denaturation model (Supporting Information, Section 16). Bimolecular aggregates of ***c*-P12_OOct_** and ***c*-P12*_t_*_-Bu_**, gave sigmoidal breakup isotherms with Hill coefficients (*n*_H_) of ca. 6.6 and 7.6, respectively. Denaturation of the bimolecular aggregates of ***c*-P8_OOct_** and ***c*-P8*_t_*_-Bu_** also gave sigmoidal breakup isotherms but with smaller Hill coefficients of ca. 3.9 and 3.7, respectively. The fact that *n*_H_ is much greater than unity demonstrates that the disaggregation processes exhibit positive cooperativity, as expected for a process in which binding to many molecules of pyridine is required to break up a bimolecular aggregate.^44^ These UV–vis denaturation curves do not fit well to a two-state model, implying that partially disaggregated intermediates are significantly populated, which is surprising because under the conditions of a ^1^H NMR titration, we only observe aggregate **(*c-*P*N*)_2_** and pyridine complex ***c-*P*N*·Py*_N_***, not intermediates of the type **(*c-*P*N*)_2_·Py*_x_*** (e.g., see later in Figure 8).

### Probing the Structure of the Aggregates by NMR Spectroscopy

#### Proposed Model for the Bimolecular Sandwich Aggregates

**Figure 3:**
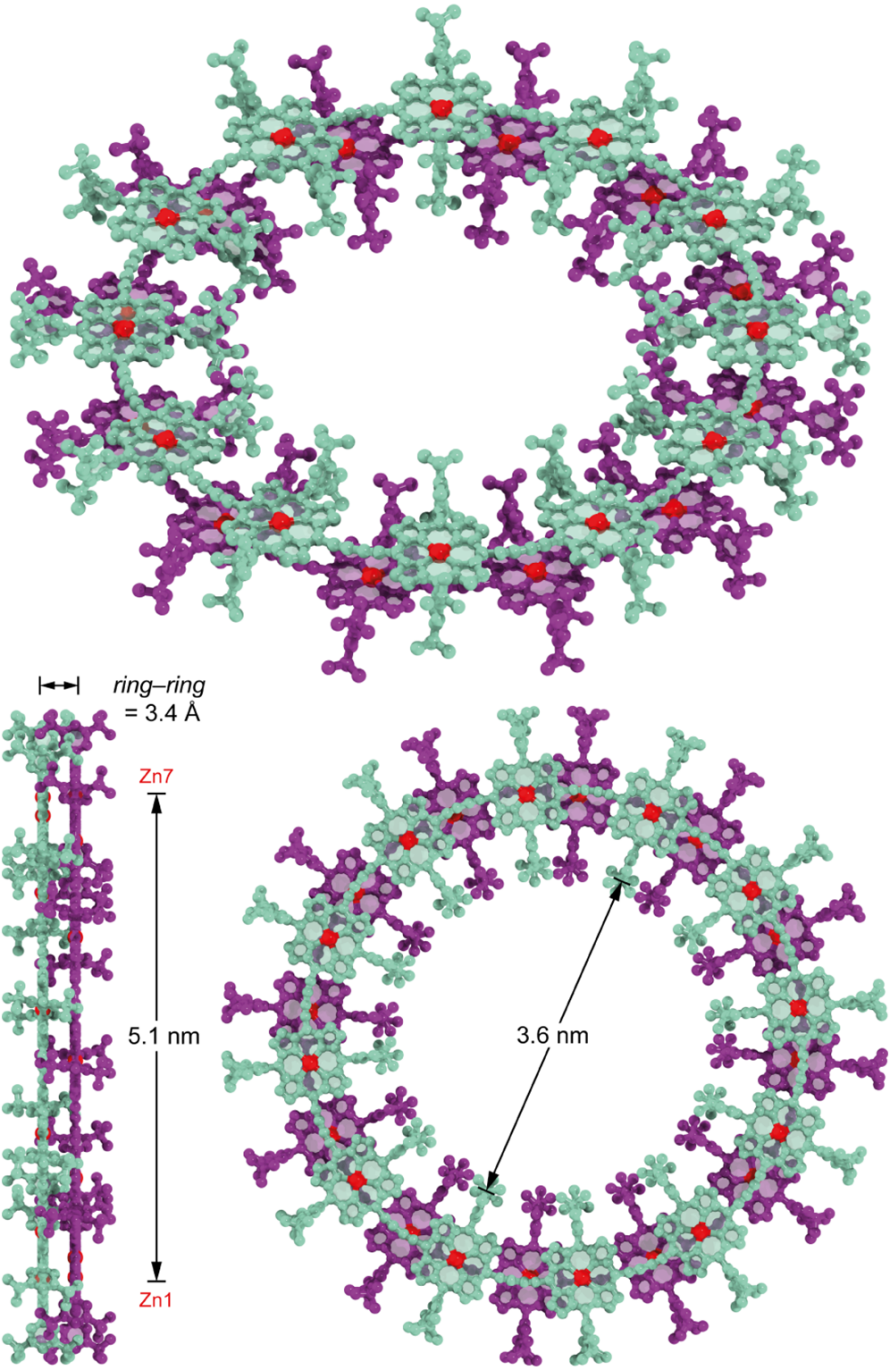
Model of the **(*c*-P12*_t_*_-Bu_)_2_** aggregate. Teal and purple coding of carbon and nitrogen atoms have been used to highlight the two separate rings of the aggregate, while zinc atoms are in red. Hydrogen atoms have been omitted for clarity.

In the NMR analysis presented below, we focus on the ^1^H NMR assignment of the bimolecular sandwich aggregate **(*c*-P12*_t_*_-Bu_)_2_**. Our model for the aggregate, introduced in Figure 3, is an idealized structure constructed using structural parameters from the xTB minimized geometry (Supporting Information, Section 14). The porphyrin units of each nanoring define two parallel planes separated by 3.4 Å, and the model deviates from a perfectly staggered conformer by rotation of one ring by 2.8°, which increases the π–π overlap between porphyrin units, resulting in *S*_24_ symmetry. Calculations indicate that the perfectly staggered conformer with *D*_12d_ symmetry is a transition state through which two low-energy, π–π-stabilized conformers can interconvert by ring rotation. In most cases, this rotation is fast on the NMR time scale, averaging the two low-energy conformers to give virtual *D*_12d_ symmetry. The NMR spectra of the aggregates of ***c*-P12_OOct_**, ***c*-P8*_t_*_-Bu_**, and ***c*-P8_OOct_** can all be assigned with a similar model.

#### ^1^H Labeling System

**Figure 4:**
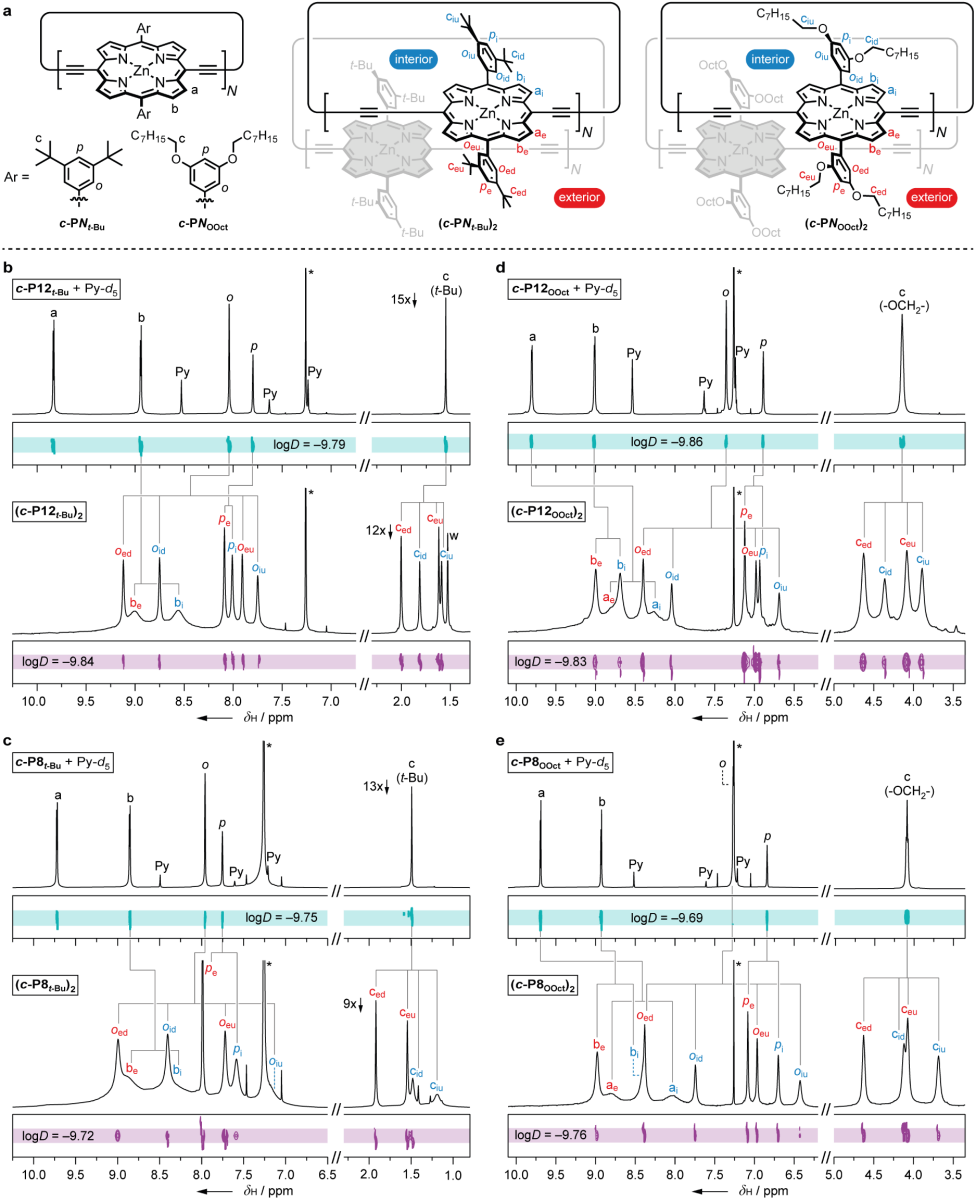
**a**) General structures with proton labels of free nanorings and their bimolecular aggregates. **b–e**) ^1^H and ^1^H DOSY spectra (500 MHz, CDCl_3_, 25 °C) of ***c*-P12*_t_*_-Bu_**, ***c*-P8*_t_*_-Bu_**, ***c*-P12_OOct_**, and ***c*-P8_OOct_** in the presence and absence of pyridine. * = residual solvent signal, Py = pyridine.

The unaggregated nanorings exhibit simple ^1^H NMR spectra, reflecting their high symmetries. The four aromatic signals are assigned to two types of β-pyrrole protons (labeled “*a*” and “*b*”), one type of *ortho*-aryl protons (*o*), and one type of *para*-aryl protons (*p*) (Figure 4a). When two rings associate to form a sandwich, protons on the interior, near the central cavity, become different from those on the exterior side, near the outer perimeter, while protons pointing toward the interface of the sandwich (near the “jam and butter”) become different from those on the outside face pointing away from the interface (Figure 4a). To distinguish between these environments, we introduce the following subscripts: i = interior, e = exterior, and arbitrarily, d = down (interface), and u = up (outside). Furthermore, if rotation between the two π–π stabilized conformers is fast on the NMR time scale, then β-pyrrole protons on opposite sides of the aryl substituent are expected to average into one type of “*a*” and one type of “*b*” environment within the interior and exterior regions of the aggregate. On the other hand, if this process is slow on the NMR time scale, each porphyrin unit is expected to become further desymmetrized resulting in eight distinct β-pyrrole proton environments (as discussed below, Figure 7).

#### 1D ^1^H NMR Spectra

Aggregation of ***c*-P12*_t_*_-Bu_** doubles the number of β-pyrrole signals and results in four *t*-Bu signals (Figure 4b), as expected for the model shown in Figure 3. The four *t*-Bu resonances correspond to two exterior (*c*_ed_ and *c*_eu_) and two interior (*c*_id_ and *c*_iu_), with one *t*-Bu pointing toward the interface (*c*_ed_ and *c*_id_) of the aggregate and one pointing away from it (*c*_eu_ and *c*_iu_) on both the exterior and interior side. Now looking to the aromatic region, four *ortho*-aryl resonances (*o*_ed_, *o*_eu_, *o*_id_, and *o*_iu_) can be assigned using the same logic. Because the *para*-aryl protons lie in the plane of the porphyrin unit, only two *para*-aryl signals (*p*_e_ and *p*_i_) are expected in **(*c*-P12*_t_*_-Bu_)_2_**. The two broad aromatic signals (*b*_e_ and *b*_i_) are assigned as the “*b type*” β-pyrrole protons next to the aryl substituent on the exterior and interior sides. The remaining “*a type*” β-pyrrole protons next to the butadiyne links (*a*_i_ and *a*_e_) are not visible at 25 °C due to exchange broadening (see later, Figure 7). The count of β-pyrrole resonances is consistent with a fast interconversion between the two π–π stabilized conformers on the NMR time scale, as discussed below. Turning to **(*c*-P8*_t_*_-Bu_)_2_**, **(*c*-P12_OOct_)_2_**, and **(*c*-P8_OOct_)_2_** (Figure 4c-e), comparable ^1^H NMR spectra are seen to that of **(*c*-P12*_t_*_-Bu_)_2_**. Notably, the OCH_2_ side chain resonances in the aggregates with OOct groups display the same splitting pattern as the *t*-Bu resonances in the aggregates with *t*-Bu groups, and the “*a type*” β-pyrrole protons are observable at 25 °C in the aggregates with OOct solubilizing groups.

We considered the possibility that the aggregates could consist of more than two nanorings. However, the ^1^H NMR spectra are not consistent with a trimolecular sandwich, for which two distinct porphyrin environments are expected to be in a 1:2 ratio. ^1^H diffusion-ordered NMR spectroscopy (DOSY) revealed a diffusion coefficient of 1.45 × 10^–10^ m^2^ s^–1^ for the **(*c*-P12*_t_*_-Bu_)_2_** aggregate and 1.62 × 10^–10^ m^2^ s^–1^ for its disaggregated state (pyridine complex). Likewise, the three other aggregates only exhibited a minor change in diffusion coefficient, comparing between their aggregated and disaggregated pyridine complexes. In the case of ***c*-P8*_t_*_-Bu_** and ***c*-P12_OOct_**, the disaggregated state even appeared to exhibit a slightly smaller diffusion coefficient than the aggregated state (see the values of log*D* in Figure 4). Such a small variation in diffusion coefficients supports the formation of a compact bimolecular aggregate, which is conformationally restricted compared to the disaggregated rings.

#### 2D NMR Spectra

**Figure 5:**
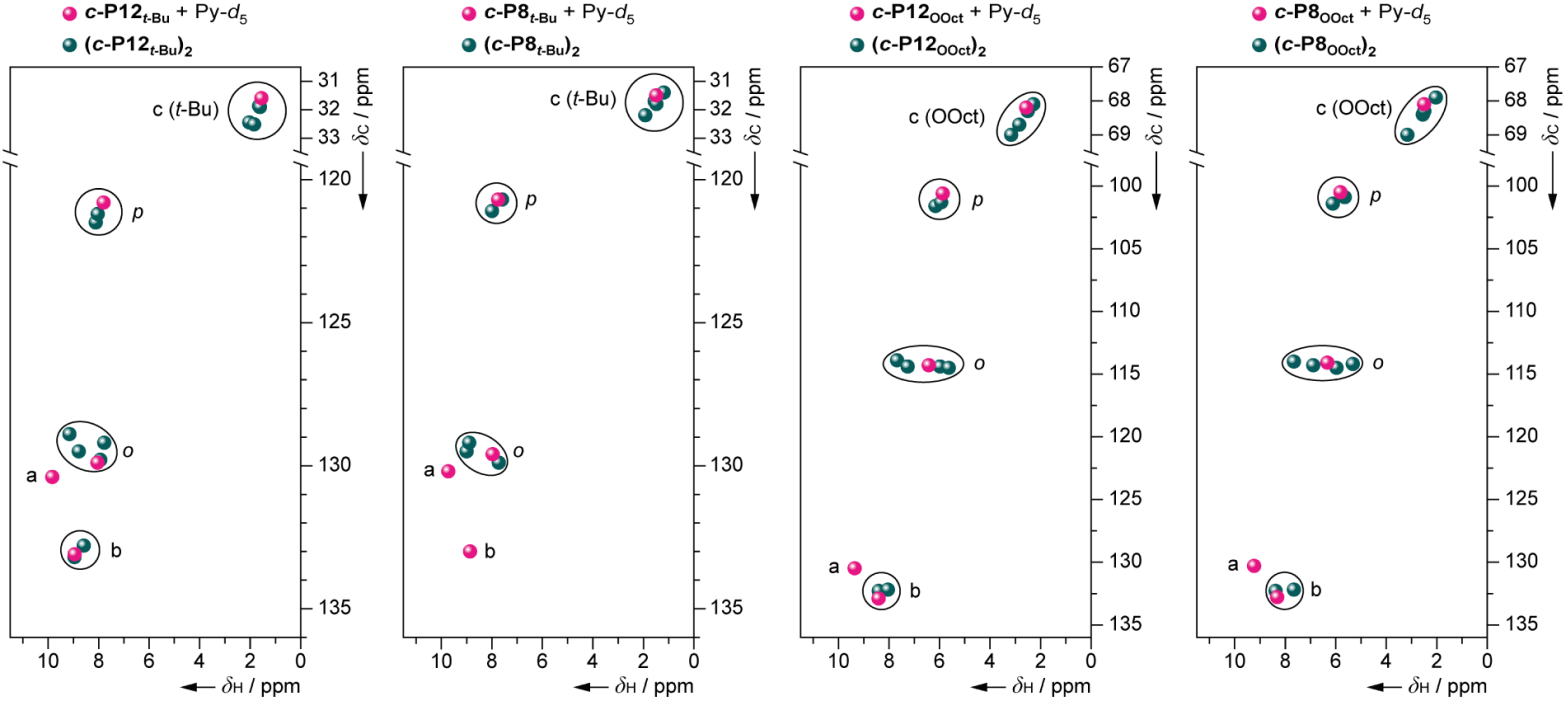
Comparative plots of HSQC cross-peaks for ***c*-P12*_t_*_-Bu_**, ***c*-P8*_t_*_-Bu_**, ***c*-P12_OOct_**, and ***c*-P8_OOct_** in the presence (pink) and absence of pyridine (green). The grouping of cross-peaks measured for aggregates (green) around cross-peaks measured for their free rings in the presence of pyridine (pink) was used to correlate between different types of protons in the free rings and their aggregates. ^1^H signals of “*a*” type β-pyrrole protons in all aggregates are broadened out beyond the limit of detection; thus, only the point for the free rings in the presence of pyridine is shown. Similarly, “*b*” type resonances are not observable in **(*c*-P8*_t_*_-Bu_)_2_**, only in the corresponding free ring.

The ^1^H signals of **(*c*-P12*_t_*_-Bu_)_2_** can be assigned to either the exterior (e) or interior (i) groups of resonances by means of 2D ^1^H–^1^H COSY, TOCSY, and NOESY spectroscopies. No ^1^H–^1^H correlations were observed between the two groups, but it is not immediately apparent which group is exterior and which is interior. Within these two groups, the type of aromatic protons (*ortho*-aryl, *para*-aryl, and β-pyrrole) can be assigned, based on conventional analysis of through-bond and through-space correlations. The assignment of proton types is further supported by ^1^H–^13^C HSQC spectra, because the ^13^C chemical shifts are not very sensitive to aggregation (Figure 5).^38^ For example, in the HSQC spectrum of **(*c*-P12*_t_*_-Bu_)_2_**, the expected number of cross peaks fall within the same ^13^C chemical shift region as *t*-Bu, *para*-aryl, *ortho*-aryl, and β-pyrrolic “*b*” H–C cross peaks in disaggregated ***c*-P12*_t_*_-Bu_**. These assignments were confirmed by ^1^H–^1^H EXSY experiments probing exchange between **(*c*-P12*_t_*_-Bu_)_2_** and ***c*-P12*_t_*_-Bu_·Py_12_** discussed below.

#### NOE Spectroscopy

**Figure 6:**
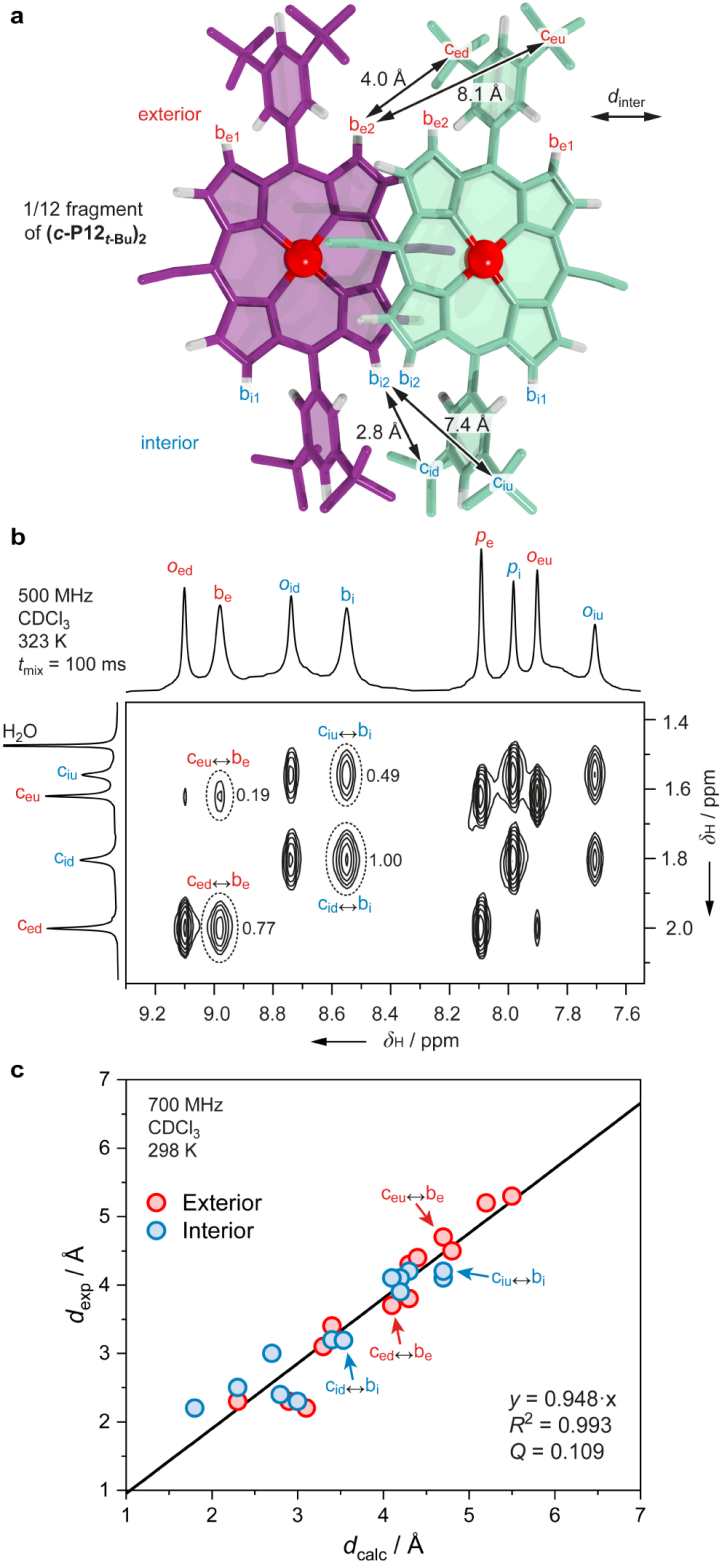
^1^H–^1^H NOESY analysis of **(*c*-P12*_t_*_-Bu_)_2_**. **a**) A 1/12th fragment of the model with the key calculated intermolecular distances used to distinguish between the exterior (e)/interior (i) and up (u)/down (d) proton environments. Hydrogen atoms of the *t*-Bu groups are omitted for clarity. **b**) Region of the NOESY spectrum highlighting key differences in NOE magnitudes for the assignment. **c**) Calculated versus experimental distances for the **(*c*-P12*_t_*_-Bu_)_2_** aggregate.

^1^H–^1^H NOESY spectroscopy allowed us to distinguish exterior vs interior protons, and to distinguish protons pointing toward or away from the interface of the bimolecular aggregate **(*c*-P12*_t_*_-Bu_)_2_**. Because of the close interdigitation of porphyrin units in the model, we expected that several ^1^H–^1^H NOE correlations would originate from a combination of two contributions: an intra-ring NOE and an inter-ring NOE, which would add up to give the observed cross peak (NOE = NOE_intra_ + NOE_inter_). We reasoned this could be useful for differentiating proton environments based on differences in NOE intensity, because the inter-ring contribution should be larger in more sterically crowded regions near the center of the aggregate, while the intra-ring contribution should be similar throughout, i.e., independent of aggregation (Figure 6a). We first measured a ^1^H–^1^H NOESY spectrum at 50 °C because the β-pyrrole resonances *b*_e_ and *b*_i_ are sharper at this temperature (Figure 6b). The assignment can be rationalized based on four key ^1^H–^1^H correlations between *t*-Bu and β-pyrrole resonances listed in Table 1. Based on model distances, the expected NOE distances can be calculated for the four key correlations according to eq 1,
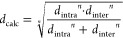
1where *d*_intra_ and *d*_inter_ designate the intra- and inter-ring proton separations, and the factor *n* is the NOE distance dependency. Here, we use a distance exponent *n* = 3, as shown to be appropriate for NOEs to methyl groups.^45^ The expected ranking of experimental NOE magnitudes among the four key correlations follows from the calculated distances: *d*_calc_ = 3.5 Å (*b*_i_ ↔ *c*_id_) < 4.1 Å (*b*_e_ ↔ *c*_ed_) < 4.7 Å (*b*_i_ ↔ *c*_iu_) = 4.7 Å (*b*_e_ ↔ *c*_eu_). In light of the integrated cross peaks (Figure 6b), this allows us to assign both the exterior and interior protons, because the largest cross peak is expected in the interior region, and assign the “up” and “down” environments because the largest cross peak is expected for the latter (the environment close to the interface). In agreement with our hypothesis, the ranking of calculated distances is mainly determined by the differences in inter-ring contributions to the NOE. Once the *t*-Bu signals have been assigned, the *ortho*-aryl signals follow, based on the strength of their NOE correlations to the *t*-Bu signals.

**Table 1:**
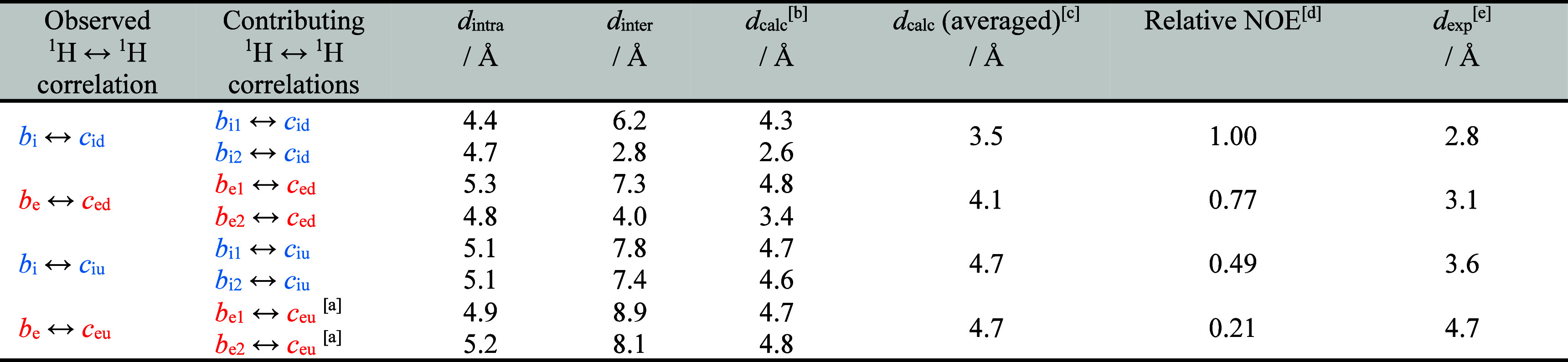
**|** Key Calculated and Experimental NOE Distances in **(*c*-P12*_t_*_-Bu_)_2_**^a^^b^^c^^d^^e^

a Reference correlation used for scaling other interactions of similar type.

b Calculated NOE distances taking into account both intra- and intermolecular contributions, calculated according to eq 1 using model distances and *n* = 3.^45^ Distances to *t*-Bu groups (i.e., *c*_iu_, *c*_id_, *c*_eu_, and *c*_ed_) were measured from the particular methyl group center of mass, resulting in the shortest possible distance.

c Averaged calculated distance taking into account a fast interconversion between both π–π stabilized conformers of **(*c*-P12*_t_*_-Bu_)_2_**.

d Relative NOEs correspond to the integrated cross peak volumes from 2D NOESY (500 MHz, CDCl_3_, 323 K, *t*_mix_ = 100 ms).

e Experimental NOE distances calculated from the integrated NOEs and the reference distance.

We carried out a complete NOE analysis, taking into account all discernible ^1^H–^1^H correlations using a high-resolution data set obtained at 700 MHz at 25 °C, which enabled us to include additional correlations close to the diagonal (Supporting Information, Section 7). Plotting the calculated distances against experimental distances revealed good agreement in support of our assignment (*R*^2^ = 0.993; Figure 6c). If, on the other hand, the exterior/interior, the up/down, or both of these proton environments are switched at the same time, worse fits are obtained to the experimental NOE distances (*R*^2^ = 0.942–0.963; Supporting Information, Section 7).

#### Variable-Temperature NMR Spectroscopy

**Figure 7:**
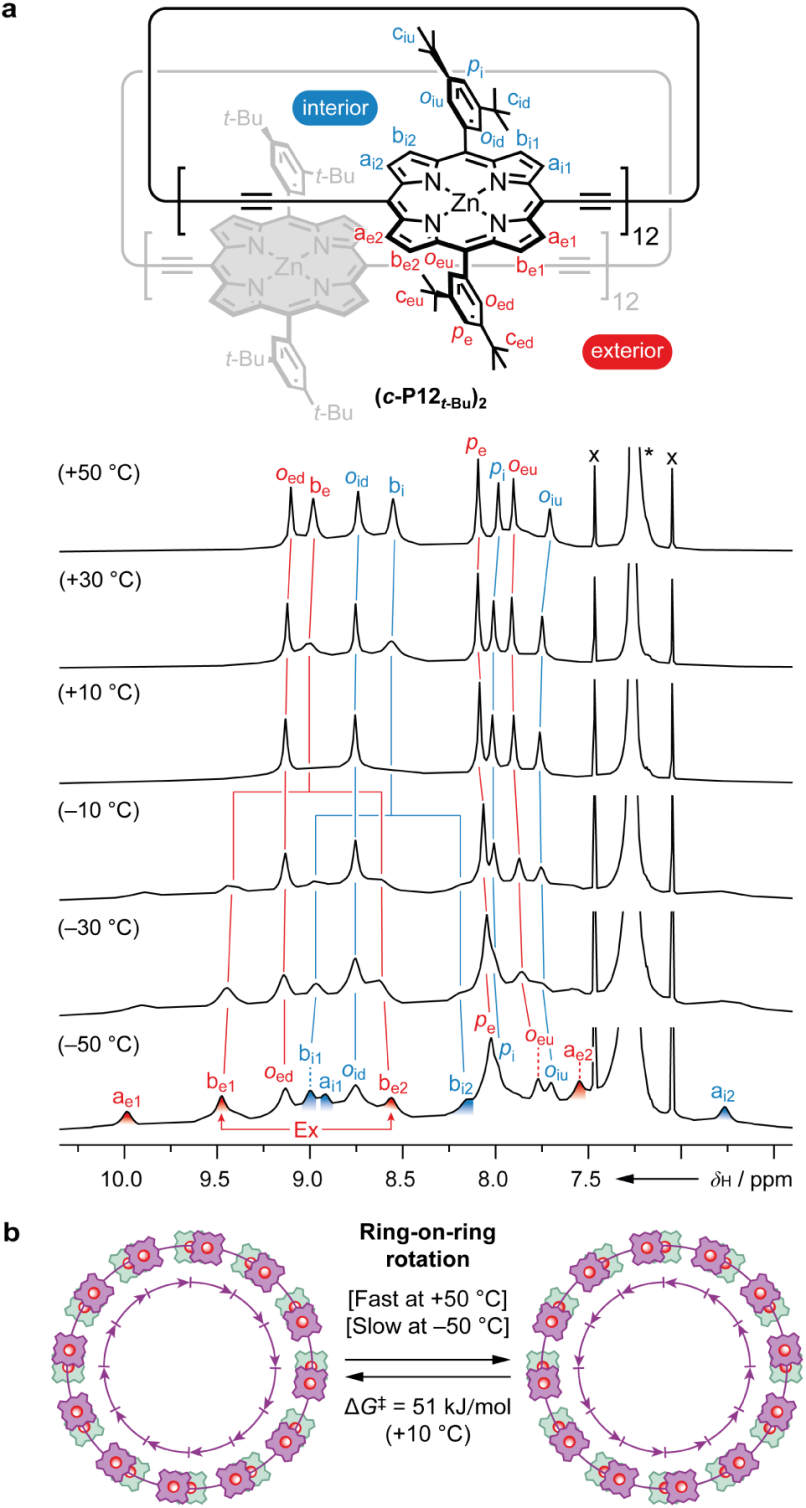
Variable-temperature ^1^H NMR spectra of the bimolecular aggregate of ***c*-P12*_t_*_-Bu_**. **a**) Structure of **(*c*-P12*_t_*_-Bu_)_2_** with proton labeling at −50 °C and ^1^H NMR spectra from +50 °C to −50 °C, recorded at 500 MHz in CDCl_3_. * = residual solvent signal; x = CDCl_3_ satellite signals; Ex = exchange, detected by ^1^H–^1^H EXSY (ROESY) spectroscopy. **b**) Cartoon representation of the ring-on-ring rotation process in **(*c*-P12*_t_*_-Bu_)**_**2**_ leading to fast exchange of β-pyrrole protons at +50 °C, and slow exchange at −50 °C.

We were able to modulate the rate of ring-on-ring rotation to access both fast and slow exchange regimes in **(*c*-P12*_t_*_-Bu_)_2_** by variable-temperature NMR spectroscopy (Figure 7). Cooling **(*c*-P12*_t_*_-Bu_)**_**2**_ from +50 to +10 °C led to broadening of the β-pyrrole resonances *b*_e_ and *b*_i_, followed by the rise of eight new β-pyrrole signals at −50 °C, consistent with a switch from fast to slow exchange. As a result of porphyrin desymmetrization at low temperature, we have introduced the subscripts “1” and “2” to distinguish between β-pyrrole protons being closer to the butadiyne-link or to the porphyrin unit of the neighboring ring, respectively. The following *J*-coupled pairs of “*a*” and “*b*” type β-pyrrole protons could be assigned based on ^1^H–^1^H COSY and TOCSY experiments: *a*_e1_/*b*_e1_, *a*_e2_/*b*_e2_, and *a*_i2_/*b*_i2_, while the remaining *a*_i1_/*b*_i1_ pair is too close to the diagonal to observe any correlations between the two signals. The pairs of β-pyrrole protons were assigned to the exterior or interior regions based on ^1^H–^1^H NOESY correlations to assigned *t*-Bu and *ortho*-aryl resonances. The chemical shift of exchange averaged signals *b*_e_ (δ_H_ = 8.98 ppm) and *b*_i_ (δ_H_ = 8.55 ppm) at +50 °C served as a guide for assigning the contributing β-pyrrole protons *b*_e1_/*b*_e2_ (midpoint = 9.02 ppm) and *b*_i1_/*b*_i2_ (midpoint = 8.57 ppm) at −50 °C. Although the exchange averaged signals of the “*a*” type β-pyrrole resonances are not discernible at +50 °C in CDCl_3_, they are seen at +140 °C in tetrachloroethane-*d*_2_: *a*_e_ (δ_H_ = 8.74 ppm) and *a*_i_ (δ_H_ = 8.09 ppm) (Supporting Information, Section 10). Similarly, the midpoint of the following pairs of exterior and interior “*a*” type β-pyrrole protons are consistent with the chemical shift values of the exchange averaged signals in tetrachloroethane-*d*_2_: *a*_e1_/*a*_e2_ (midpoint = 8.72 ppm) and *a*_i1_/*a*_i2_ (midpoint = 7.84 ppm). The assignment of β-pyrrole protons closer to the butadiyne link (labeled ″1”) or closer to the porphyrin unit (labeled ″2”) of the neighboring ring was rationalized based on chemical shifts. The “*a*” type β-pyrrole protons closer to the porphyrin unit of the neighboring ring are expected to experience a decrease in chemical shift relative to the exchange averaged value, due to shielding by the porphyrin unit. This assumption is also supported by predicted ^1^H chemical shifts from DFT calculations (Supporting Information, Section 14). Thus, the decreased chemical shift of β-pyrrole protons *a*_e2_ (Δδ_H_ = −1.19 ppm) and *a*_i2_ (Δδ_H_ = −1.32 ppm) implies that these protons are closer to the porphyrin unit, whereas the increased shift of *a*_e1_ (Δδ_H_ = +1.24 ppm) and *a*_i1_ (Δδ_H_ = +0.82 ppm) suggests that they are closer to the butadiyne link of the neighboring ring.

The rate and activation barrier for the ring-on-ring rotation process was estimated as 1.96 kHz and 51 kJ mol^–1^, respectively, at the coalescence temperature (*T*_c_ ≈ + 10 °C) of the β-pyrrole *b*-type resonances from their peak separations: *b*_e1_–*b*_e2_ (Δ*v* = 459 Hz) and *b*_i1_–*b*_i2_ (Δ*v* = 425 Hz). Essentially, the same activation barrier (51 kJ mol^–1^) was determined at −50 °C, by 2D ^1^H–^1^H EXSY spectroscopy by following the exchange correlation between *b*_e1_ and *b*_e2_ as a function of mixing times (*t*_mix_ = 20–200 ms), suggesting a small entropic contribution to this barrier (Supporting Information, Section 12).

#### Exchange Spectroscopy

**Figure 8:**
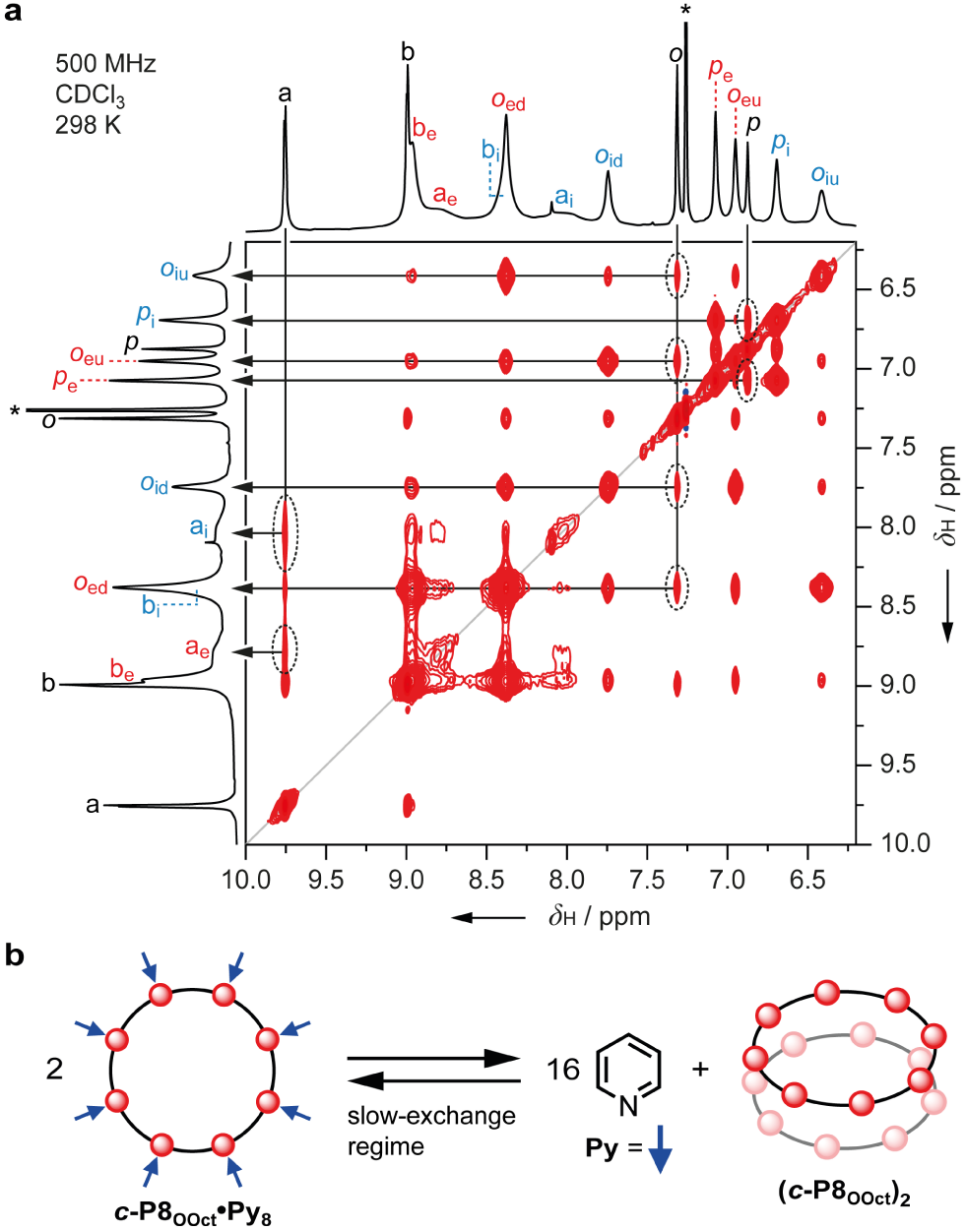
**a**) ^1^H–^1^H EXSY (NOESY) spectrum of a 2.2:1.0 mixture of **(*c*-P8_OOct_)_2_** and ***c*-P8_OOct_·Py_8_** showing direct exchange correlations between the two states (500 MHz, CDCl_3_, 25 °C, *t*_mix_ = 200 ms). **b**) Cartoon representation of the equilibrium between ***c*-P8_OOct_·Py_8_** and **(*c*-P8_OOct_)_2_** giving rise to exchange correlations in 2D EXSY. Key exchange correlations between disaggregated ring and aggregate include: *a* ↔ *a*_i_, *a* ↔ *a*_e_, *o* ↔ *o*_iu_, *o* ↔ *o*_eu_, *o* ↔ *o*_id_, *o* ↔ *o*_ed_, *p* ↔ *p*_i_, and *p* ↔ *p*_e_.

When small amounts of pyridine-*d*_5_ are added to the aggregates, it is possible to record ^1^H NMR spectra that display peaks due to both aggregated and disaggregated species, in slow exchange on the chemical shift time scale, but in fast exchange on the *T*_1_ time scale. This allowed us to correlate assignments directly between the aggregated and disaggregated states. Characterization of the mixtures by 2D ^1^H–^1^H EXSY experiments (Supporting Information, Section 5) revealed direct correlation between signals from the aggregated and disaggregated nanorings, although in some cases these cross peaks were obscured by overlap between the ^1^H resonances from the two states. In the best scenario, 2D EXSY of a 2.2:1.0 mixture of **(*c*-P8_OOct_)**_**2**_ and ***c*-P8_OOct_·Py_8_** allowed us to directly correlate the *para*-aryl, *ortho*-aryl, and “*a*” type β-pyrrole protons of the disaggregated ring with their respective proton resonances in the bimolecular aggregate (Figure 8). In ***c*-P8_OOct_** and ***c*-P8*_t_*_-Bu_**, exchange between the two states could be detected at 25 °C, whereas for ***c*-P12_OOct_** and ***c*-P12*_t_*_-Bu_**, exchange is too slow at 25 °C and it was necessary to heat the solution to 50 °C.

**Figure 9:**
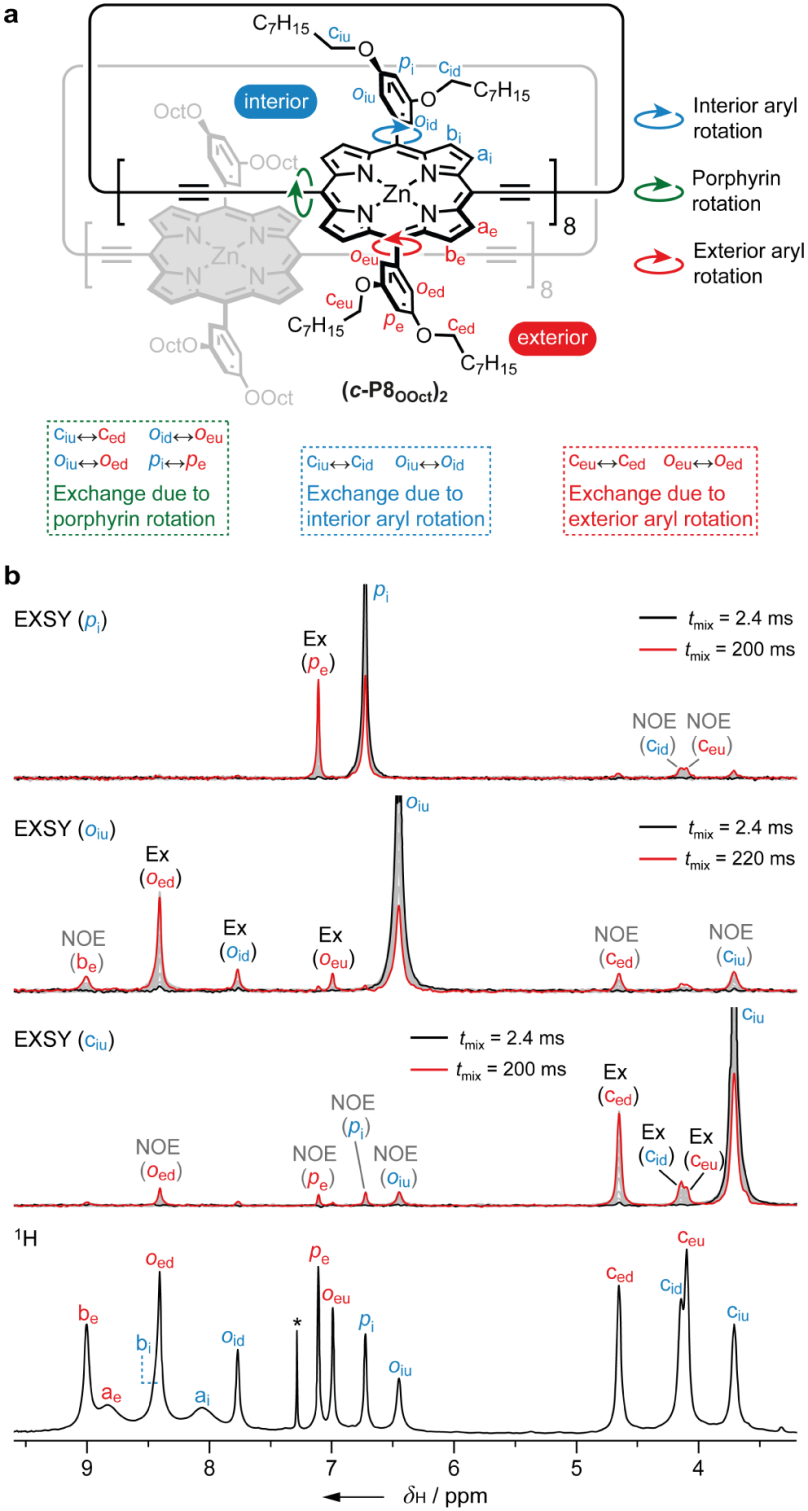
**a**) Structure of **(*c*-P8_OOct_)**_**2**_ with arrows indicating three internal rotation processes: interior aryl rotation (blue arrow), porphyrin rotation (green arrow), and exterior aryl rotation (red arrow), identified based on characteristic proton exchanges listed below. **b**) From top to bottom: selective ^1^H EXSY NMR spectra (CDCl_3_, 500 MHz, 25 °C) at different mixing times for targeted protons *p*_i_ (*t*_mix_ 2.4–200 ms), *o*_iu_ (*t*_mix_ 2.4–220 ms), *c*_iu_ (*t*_mix_ 2.4–220 ms), and ^1^H NMR spectrum of **(*c*-P8_OOct_)_2_**.

2D ^1^H–^1^H ROESY experiments revealed rich dynamic behavior in **(*c*-P8*_t_*_-Bu_)_2_** and **(*c*-P8_OOct_)_2_**, in contrast to the larger 12-ring analogues (Supporting Information, Section 8). The dynamic processes in the 8-ring aggregates manifest in several exchange correlations that can be distinguished from through-space ^1^H–^1^H correlations based on the phase of the cross-peak relative to the diagonal. First, we identified exchange correlations between protons of the same type pointing toward and away from the interface of the aggregate, between *ortho*-aryl (*o*_iu_ ↔ *o*_id_ and *o*_eu_ ↔ *o*_ed_) and *t*-Bu (*c*_iu_ ↔ *c*_id_ and *c*_eu_ ↔ *c*_ed_) protons, which are consistent with a rotation process of the exterior and interior aryl substituent (Figure 9a). Second, we found exchange correlations between exterior and interior proton resonances, namely, *c*_iu_ ↔ *c*_ed_, *o*_id_ ↔ *o*_eu_, *o*_iu_ ↔ *o*_ed_, and *p*_i_ ↔ *p*_e_, which imply rotation of a porphyrin unit. This process exchanges protons pointing toward the interface of the aggregate on the exterior side with protons pointing away from it on the interior side. In contrast, correlations between resonances across the interior/exterior regions that both point toward the interface of the aggregate or both point away from it (e.g., *o*_ed_ ↔ *o*_id_, *o*_eu_ ↔ *o*_iu_, *c*_ed_ ↔ *c*_id_, and *c*_eu_ ↔ *c*_iu_) do not arise from this process. This exchange pattern is consistent with rotation of the porphyrin unit, via partial disaggregation within the bimolecular aggregate. If the rings were to disaggregate completely, the exchange pattern would be expected to reflect a scrambling of the ring faces, which is not observed. It seems likely that a segment of the 8-ring aggregate disaggregates reversibly, providing enough flexibility for the porphyrin unit to rotate before the segment reassociates. The fact that exchange stemming from porphyrin rotation is not observed in either **(*c*-P12*_t_*_-Bu_)_2_** or **(*c*-P12_OOct_)_2_** reflects the greater stability of the 12-ring aggregates, due to stronger π–π interactions and a smaller energy penalty from planarization, in comparison to the 8-ring aggregates (Supporting Information, Section 14). This interpretation is consistent with molecular dynamics simulations of the 8- and 12-ring aggregates (discussed below, Figure 11).

The exchange processes in **(*c*-P8_OOct_)_2_** were probed quantitatively by means of 1D ^1^H EXSY experiments (Figure 9b). By selectively exciting one of three resonances (*p*_i_, *o*_iu_, and *c*_iu_) and recording the ^1^H spectra as a function of mixing time, we tracked the evolution of magnetization as a result of exchange. Excitation of *p*_i_ leads to the transfer of magnetization to *p*_e_ as a result of porphyrin rotation. On the other hand, protons *o*_iu_ and *c*_iu_ are both affected by both porphyrin rotation and interior aryl rotation. Even rotation about the exterior aryl group was probed via excitation of the *o*_iu_ and *c*_iu_ resonances because porphyrin rotation is sufficiently fast. The EXSY experiments resulted in three series of exchange intensities as a function of mixing time (Supporting Information, Section 9). A kinetic model containing all three rotation processes was used to carry out a global fit, yielding rate constants for the porphyrin (6.7 Hz), interior aryl (1.5 Hz), and exterior aryl (1.0 Hz) rotations in **(*c*-P8_OOct_)_2_** at 298 K. The corresponding activation barriers are 68, 72, and 73 kJ mol^–1^ for the porphyrin, interior aryl, and exterior aryl rotation, respectively. The barrier to porphyrin rotation in this aggregate is considerably higher than that previously measured for a related disaggregated 8-ring in its oxidized 6+ state (45.7 kJ mol^–1^).^46^

#### Residual Dipolar Coupling

Dipolar coupling is not normally observed in solution-phase NMR spectra, due to rapid isotropic tumbling, however residual dipolar coupling (RDC) can sometimes be detected at high magnetic fields, particularly for molecules in which many aligned aromatic units result in a high magnetic susceptibility anisotropy (Δχ).^47−50^ The observation of RDCs can provide valuable structural information because the sign and magnitude of the RDC between two nuclei depend on the angle between the internuclear vector and the molecular susceptibility vector.

The first indication that the spectra of **(*c*-P12*_t_*_-Bu_)_2_** are influenced by RDCs came from the appearance of strong correlations (*c*_eu_ ↔ *p*_e_, *c*_ed_ ↔ *p*_e_, *c*_iu_ ↔ *p*_i_ and *c*_id_ ↔ *p*_i_) in the ^1^H–^1^H COSY spectrum, which are formally 5-bond *J*-couplings. We investigated the RDCs in this aggregate by recording ^13^C-coupled HSQC spectra at a range of field strengths from 11.75 (500 MHz) to 22.32 T (950 MHz). In the presence of a significant RDC (^1^*D*_CH_), which scales with the square of the external magnetic field (*B*_0_), the observed one-bond ^1^H–^13^C total splitting (^1^*T*_CH_) becomes the sum of the RDC and the field independent scalar coupling (^1^*J*_CH_) as given by eq 2.
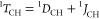
2

**Figure 10:**
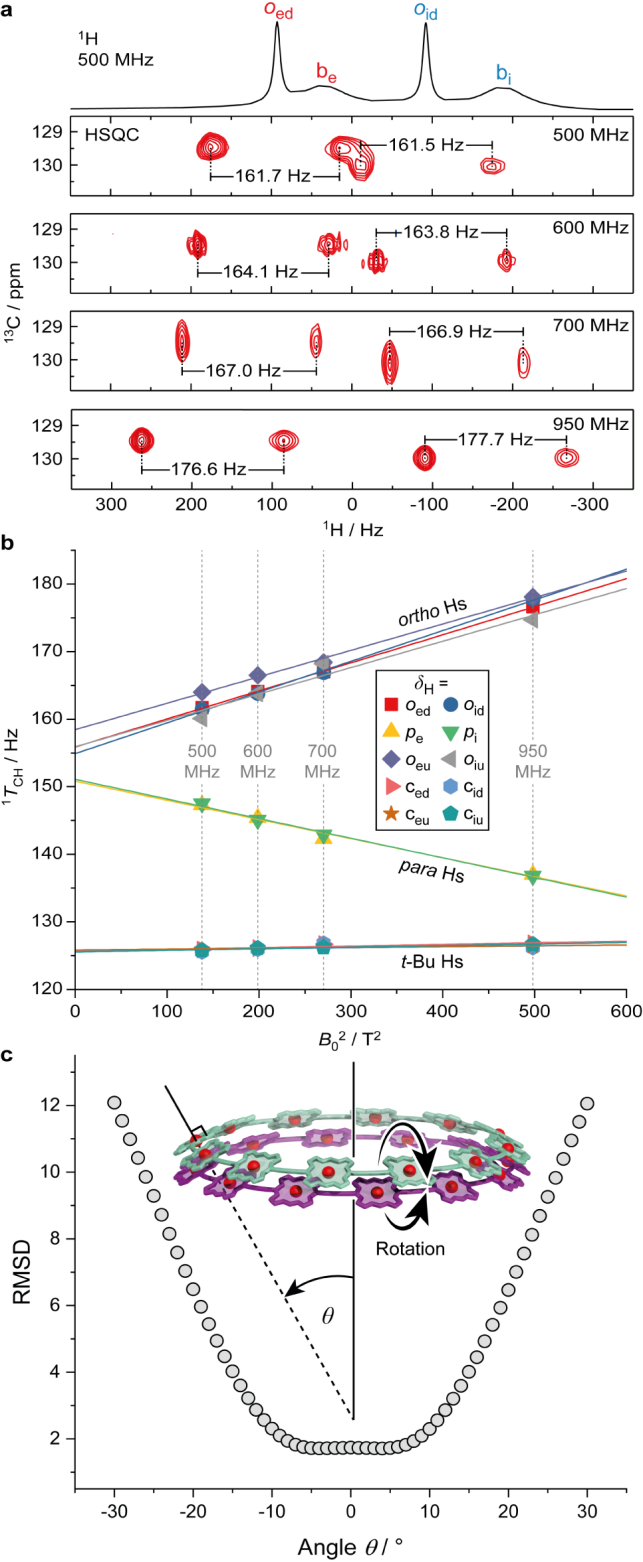
Magnetic field dependence of the aryl side chain ^1^H–^13^C total splittings (^1^*T*_CH_) for the **(*c*-P12*_t_*_-Bu_)_2_** bimolecular aggregate. **a**) ^13^C-coupled HSQC spectra for *ortho*-aryl resonances *o*_ed_ and *o*_id_, showing a field-dependence of ^1^*T*_CH_ resulting from a dipolar coupling contribution. **b**) Dependence of the observed ^1^*T*_CH_ on the square of the magnetic field strength, plotted for both *ortho-* and *para-*aryl side chain protons. **c**) Root-mean-square deviation between experimental and calculated RDCs for model geometries of **(*c*-P12*_t_*_-Bu_)**_**2**_ with varying degrees of porphyrin rotation, given as the angle θ between the plane of the ring (mean plane of 12 Zn atoms) and the plane of the porphyrin units (24-atom mean plane).

It is clear from the ^13^C-coupled HSQC spectra of **(*c*-P12*_t_*_-Bu_)_2_**, that the one-bond ^1^H–^13^C couplings involving *ortho*-aryl and *para*-aryl protons are field dependent (Figure 10a-b). By plotting ^1^*T*_CH_ against the squared magnetic field (*B*_0_^2^), the field independent scalar coupling (^1^*J*_CH_) can be determined as the *y*-intercept by extrapolation to *B*_0_^2^ = 0 (Figure 10b), which allows calculation of the residual dipolar coupling (^1^*D*_CH_; Table 2). ^13^C-coupled HSQC spectra were also recorded for disaggregated ***c*-P12*_t_*_-Bu_·Py_12_** at different field strengths, but no field dependence of ^1^*T*_CH_ was detected (Supporting Information, Section 13).

**Table 2:**
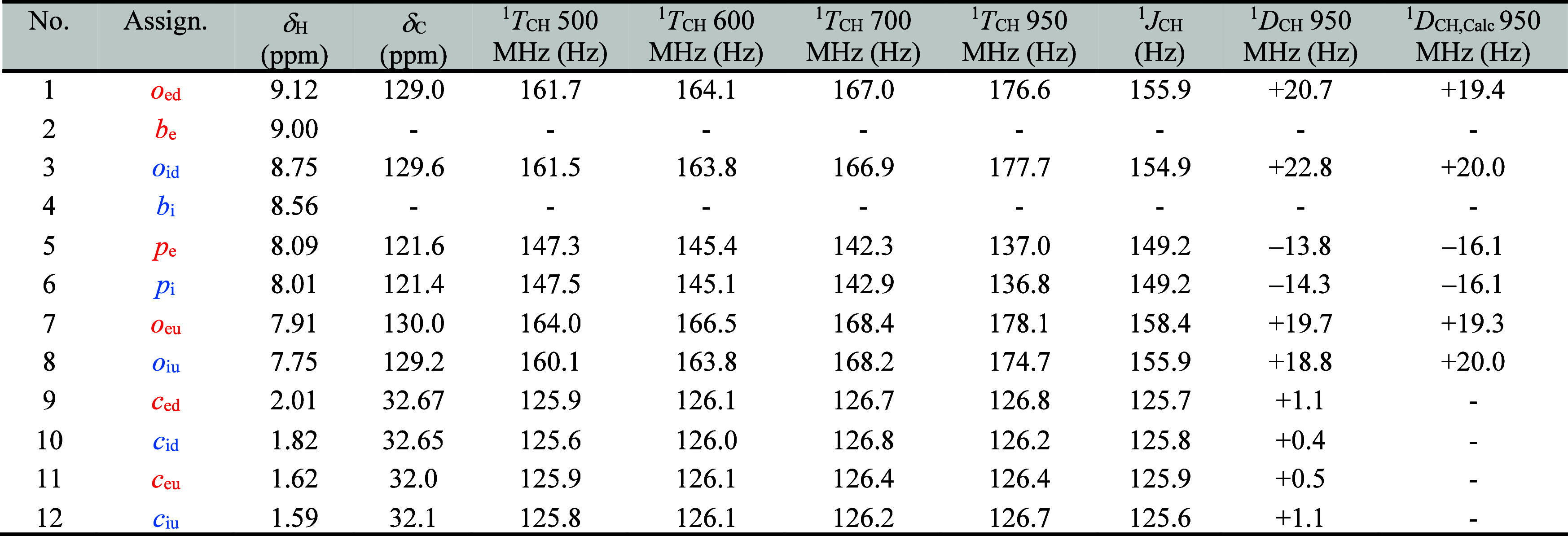
Summary of Experimentally Observed ^1^H–^13^C Total Splittings (^1^*T*_CH_), Scalar Couplings (^1^*J*_CH_), and Residual Dipolar Couplings (^1^*D*_CH_) for **(*c*-P12*_t_*_-Bu_)_2_**^a^

a Field strengths: 22.32 T (950 MHz), 16.44 T (700 MHz), 14.10 T (600 MHz), and 11.75 T (500 MHz). 1D traces of the F2 (1H) dimension from ^13^C-coupled HSQC spectra were used to record the total splittings (^1^*T*_CH_). ^1^*D*_CH,Calc_ values from the program PALES^51^ using a geometry with all porphyrin units coplanar with the nanoring plane and with aryl side chains at 90° relative to the plane of the porphyrin units.

With a proposed geometry and an adequate number of experimentally determined RDCs as a starting point, it is possible to predict the molecular alignment tensor for that structure and use it to calculate the predicted RDCs. A good agreement between the experimental and calculated RDCs validates the proposed structure. Although the limited number of RDCs for **(*c*-P12*_t_*_-Bu_)_2_** is on the border of feasibility, we undertook this analysis using the PALES software developed by Zweckstetter.^51^ Our proposed model of the bimolecular aggregate resulted in a good fit between experimental and predicted RDCs, as evidenced by an *R*^2^ factor of 0.989 (Supporting Information, Section 13). We screened other geometries by rotating the porphyrin units in synchrony out of the plane of the nanorings over a range of ±30°, which resulted in a low root-mean-square deviation (RMSD) for angles in the range ±10° (Figure 10c). We also varied the geometry by rotating the exterior and interior aryl substituents, which gave the best fits with both aryl dihedral angles around 90° relative to the plane of the nanorings (Supporting Information, Section 13).

Based on the experimentally determined dipolar couplings at different field strengths, the magnetic susceptibility anisotropy, Δχ, of **(*c*-P12*_t_*_-Bu_)_2_** can be estimated as −7.7 × 10^–27^ cm^3^ (Supporting Information, Section 13). This value is 7.9 times that of a porphyrin monomer (Δχ = −9.8 × 10^–28^ cm^3^),^52^ which is surprisingly small given that the aggregate contains 24 porphyrin units. This implies that the porphyrin units of **(*c*-P12*_t_*_-Bu_)_2_** are dynamic rather than being rigidly fixed in the same orientation, in contrast to the three-layer stack aggregate of porphyrin tetramers that we studied previously.^38^

**Figure 11:**
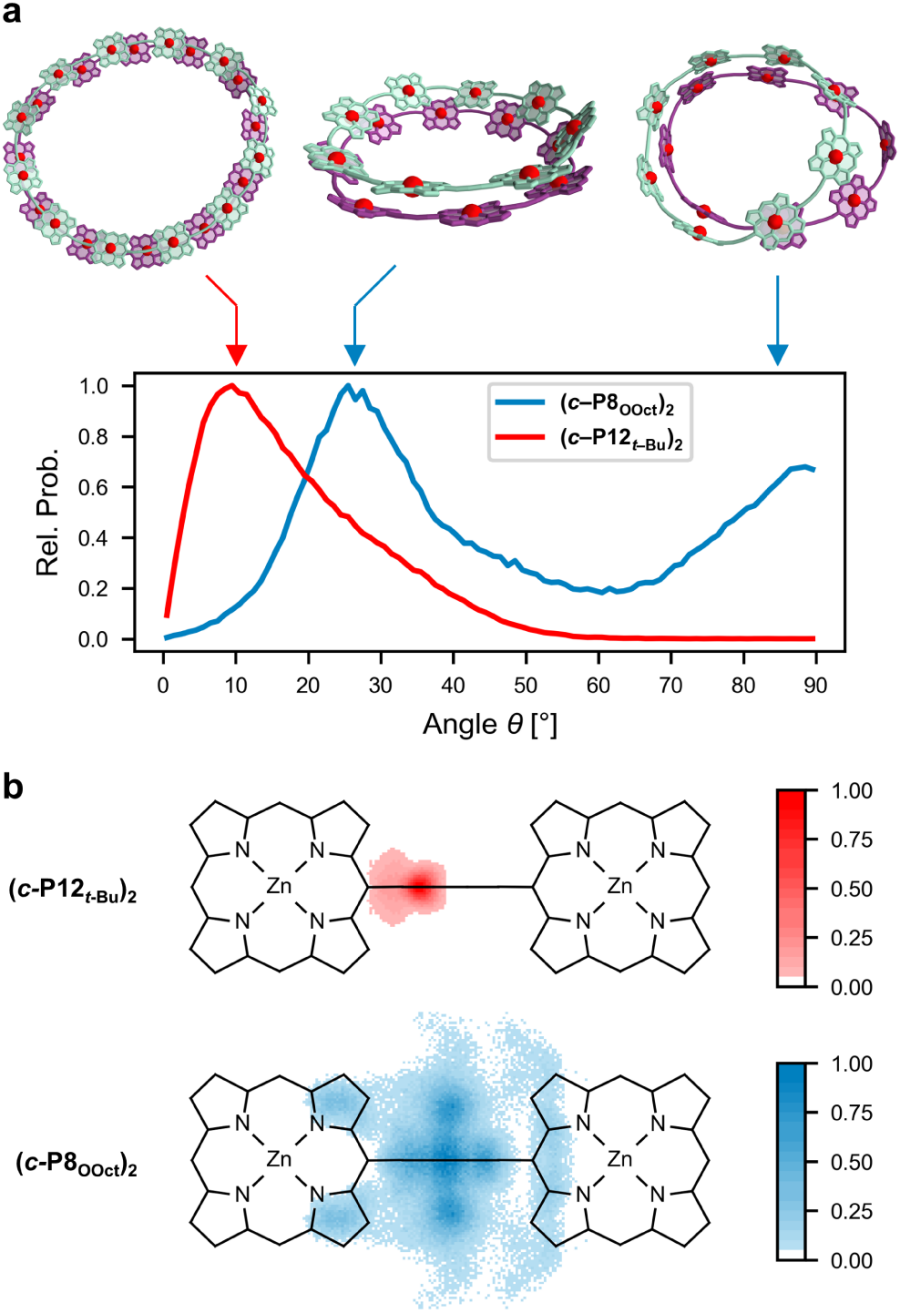
Molecular dynamics simulations of **(*c*-P12*_t_*_-Bu_)_2_** and **(*c*-P8_OOct_)_2_**. **a**) Distribution of angles (as defined in Figure 10c) between porphyrin units and the plane of the nanoring defined by all zinc atoms, together with representative geometries for some of the most populated angles. Side chains and hydrogen atoms are omitted for clarity. **b**) Distribution of porphyrin zinc atom positions in one ring projected onto the plane of the other ring of the aggregate.

We attempted to measure residual dipolar couplings in **(*c*-P8*_t_*_-Bu_)_2_**, **(*c*-P8_OOct_)_2_**, and **(*c*-P12_OOct_)_2_**. In the case of **(*c*-P8*_t_*_-Bu_)_2_** and **(*c*-P12_OOct_)_2_**, it was too difficult to accurately measure ^1^*T*_CH_ at different field strengths, due to the broadness of peaks in the ^1^H dimension. While in the case of **(*c*-P8_OOct_)_2_**, we did not observe a field-dependence of ^1^*T*_CH_, which probably reflects the more dynamic structure and bowl-shaped conformation of this aggregate, as concluded from the molecular dynamics simulations (Figure 11).

## Molecular Dynamics Calculations

To get a more detailed description of the dynamic behavior of the aggregates, we turned to molecular dynamics (MD) calculations and performed simulations of **(*c*-P12*_t_*_-Bu_)_2_** and **(*c*-P8_OOct_)_2_**. These simulations were performed on nanorings with full solubilizing groups in explicit chloroform solvent, in an isothermal–isobaric (NPT) ensemble at 300 K using GROMACS (v. 2019.2).^53^ We used the General AMBER force field,^54^ which provides a satisfactory description of dispersion interactions in π-stacked systems.^55^ Additional force field parameters for the zinc porphyrin environment were developed from DFT level calculations, as reported previously,^56^ using the LEaP program (AMBER20 tools)^57^ in conjunction with MCPB.py^58^ and restrained electrostatic potential (RESP) charge calculations. Torsional parameters for the butadiyne-linked porphyrin units were manually adjusted to reproduce the experimental rotation barriers. Details of the methodology for MD simulations and parametrization are provided in the SI (Section 14.5) and in an earlier publication.^56^

The distribution of angles between the plane of the nanorings, as defined by its zinc atoms and the plane of the porphyrin units (Figure 10c) offers a general description of the flexibility and shape of the aggregates (Figure 11a). The **(*c*-P12*_t_*_-Bu_)_2_** aggregate displays a single distribution of angles centered around ±10°. On the other hand, the **(*c*-P8_OOct_)_2_** aggregate displays a bimodal distribution around ±26° and ±89°. We also considered the zinc atom projection of one porphyrin unit onto the plane of its neighboring nanoring in the aggregates (Figure 11b). A perfectly flat concentric aggregate is expected to show a distribution of projected zinc atoms centered along the line, including the butadiyne link and the zinc atoms of the porphyrin unit of the neighboring ring, while any deviation from planarity will shift the projected zinc atoms off this line. The **(*c*-P12*_t_*_-Bu_)_2_** aggregate exhibits a relatively narrow distribution of projected zinc atoms centered on one side of the butadiyne link. The **(*c*-P8_OOct_)_2_** aggregate exhibits a much more complicated distribution of projected zinc atoms, centered partly symmetrically on the line of the butadiyne link and partly off it, and even partly above the pyrrole ring of the neighboring porphyrin unit (as in the classic Hunter-Sanders geometry of stacked porphyrins.^59^ These simulations suggest that both nanoring units in the **(*c*-P12*_t_*_-Bu_)_2_** aggregate are relatively planar and rigid, whereas the **(*c*-P8_OOct_)_2_** aggregate is fluxional and bowl shaped. In agreement with the porphyrin rotation observed by exchange NMR spectroscopy, MD of **(*c*-P8_OOct_)_2_** also show partial disaggregation of a segment of the aggregate during the time course of the simulation, which is reflected in geometries contributing to the second distribution of angles around ±89° in Figure 11a.

## Conclusions

Our results show that 8- and 12-porphyrin nanorings with *tert*-butyl (*t*-Bu) or octyloxy (OOct) side chains form stable bimolecular sandwich aggregates when dissolved in noncoordinating solvents such as chloroform, in the absence of ligands such as pyridine. The bimolecular structure was deduced from the NMR spectra of the 12-porphyrin nanoring aggregate with *tert*-butyl groups, **(*c*-P12*_t_*_-Bu_)_2_**, including DOSY, COSY, TOCSY, NOESY, and ^1^H–^13^C HSQC experiments, which permitted a full assignment of the ^1^H environments. The deduced structure is supported by analysis of RDCs using PALES software. Variable-temperature NMR spectroscopy of **(*c*-P12*_t_*_-Bu_)_2_** revealed an internal ring-on-ring rotation process that interconverts two degenerate π–π stacked conformers at a rate of 1.96 kHz at 283 K.

EXSY experiments, MD simulations, RDCs, UV–vis dilution, and denaturation titrations all indicate that the 8-ring aggregates are more fluxional and less stable than their 12-ring analogues. The **(*c*-P8_OOct_)_2_** aggregate gives a sharp ^1^H NMR spectrum, even though EXSY experiments show that the aggregate must be highly dynamic in solution in order to allow rotation of its porphyrin units. The **(*c*-P8_OOct_)_2_** aggregate did not show measurable RDCs, in contrast to the larger **(*c*-P12*_t_*_-Bu_)_2_** aggregate. UV–vis titrations also corroborated that the **(*c*-P8_OOct_)_2_** and **(*c*-P8*_t_*_-Bu_)_2_** aggregates are less stable than **(*c*-P12_OOct_)_2_** and **(*c*-P12*_t_*_-Bu_)_2_**. It seems likely that the greater flexibility of the 8-ring aggregates originates from the strain induced by planarization.

The interdigitated mode of aggregation presented here is similar to that of a previously reported three-layer stack aggregate consisting of linear porphyrin tetramers with octyloxy side chains.^38^ However, from the work presented here, it is now clear that both octyloxy and *tert*-butyl side chains allow for the formation of discrete aggregates (i.e., the *tert*-butyl group is not bulky enough to prevent aggregation). It seems likely that the bimolecular, rather than trimolecular, aggregate formation for the nanorings stems from the higher density of side chains on the interior side of rings compared to their linear analogues. Since the interior and exterior sides are bound to become more similar as the ring becomes larger, the formation of trimolecular aggregates is to be expected in larger nanorings. We previously discovered that a 24-porphyrin nanoring ***c*-P24_OOct_** forms two- and three-layer stacks on a Au(111) surface and that the circularity of the stacks increases with the number of rings.^39^ It may be valuable to be able to restrict the conformation of nanorings to enhance the electronic communication and π-conjugation around the nanoring. Hence, understanding the behavior of nanoring aggregates is an important step toward controlling their self-assembly.

## Supporting Information Available

General methods, details of absorption and emission spectroscopy, UV–vis titrations, aggregated and disaggregated NMR spectra, NMR assignments, residual dipolar coupling analysis, and quantum chemical calculations (PDF)
