# Supplementary material for: Bimolecular Sandwich Aggregates of Porphyrin Nanorings
Source: J Am Chem Soc. 2024 Aug 26;146(36):25232–44. doi: 10.1021/jacs.4c09267 (PMC11403599; doi:10.1021/jacs.4c09267)
Supplement: Supplementary file 1 — ja4c09267_si_001.pdf [file ja4c09267_si_001.pdf]

Supporting Information for

Bimolecular Sandwich Aggregates of Porphyrin Nanorings

Henrik Gotfredsen,* Janko Hergenhahn, Fernanda Duarte, Timothy D. W. Claridge and Harry L. Anderson*

Department of Chemistry, University of Oxford, Chemistry Research Laboratory, Oxford OX1 3TA, UK

*Correspondence to: henrik.gotfredsen@chem.ox.ac.uk (H.G.), harry.anderson@chem.ox.ac.uk (H.L.A.)

Table of Contents

Section 1. General Methods ... S2
Section 2. Overview of Nanoring Structures ... S3
Section 3. Overview of ^{1}H NMR Spectra of Nanorings in the Presence and Absence of Pyridine ... S4
Section 4. Comparison of HSQC Spectra of Aggregated and Disaggregated Nanorings ... S10
Section 5. EXSY Characterization of Mixtures of Aggregated and Disaggregated Nanorings ... S12
Section 6. Comments on the ^{1}H NMR Resonance Assignment ... S17
Section 7. ^{1}H–^{1}H NOE Analysis of the **(*c*-P12$_{t\text{-Bu}}$)$_2$** Aggregate ... S24
Section 8. ^{1}H-^{1}H ROESY Characterization ... S28
Section 9. Selective Magnetization Transfer Experiments to Measure Rates of Exchange ... S32
Section 10. Variable-Temperature ^{1}H NMR Series ... S40
Section 11. Comments on the Low-Temperature Assignment of **(*c*-P12$_{t\text{-Bu}}$)$_2$** ... S48
Section 12. Rate and Activation Barrier for the Ring Rotation in **(*c*-P12$_{t\text{-Bu}}$)$_2$** ... S52
Section 13. Residual Dipolar Coupling Measurements of **(*c*-P12$_{t\text{-Bu}}$)$_2$** ... S56
Section 14. Quantum Chemical Calculations ... S68
Section 15. Steady-State Absorption and Fluorescence Spectroscopy ... S78
Section 16. Aggregate Break-Up Experiments ... S83
Section 17. Diffusion-Ordered ^{1}H NMR Experiments on Nanorings ... S99
Section 18. NMR Spectra of Nanorings ... S108
Section 19. References ... S152

Data files relating to this manuscript are openly available via Zenodo; DOI: 10.5281/zenodo.12688099.
The data set includes: (a) PALES files for analyzing RDCs, (b) Coordinates of XTB-optimized geometries, (c) Transition state trajectory geometries, (d) DFT calculated geometries of ***c*-P6**, ***c*-P8**, ***c*-P10**, ***c*-P12** and ***c*-P14** when 2D planar and 3D cylindrical, (e) Molecular dynamics GROMACS input files. (f) Idealized structure for the **(*c*-P12$_{t\text{-Bu}}$)$_2$** aggregate as shown in Figure 3 of the manuscript (*xyz* coordinates).

Section 1. General Methods

Solvents for reactions were obtained from an MBraun MBSPS-5-BenchTop solvent purification system kept under nitrogen. Chloroform-*d* for NMR was stored over K_2CO_3 prior to use. All other reagents and solvents were obtained from commercial suppliers and used as received unless otherwise stated. Thin-layer chromatography (TLC) was carried out using aluminium sheets precoated with silica gel with fluorescence indicator from Merck and visualized under UV light at 254 or 365 nm. Purification by column chromatography was carried out on silica gel (SiO_2, 60 Å, 40−63 µm). Petroleum ether (PE) of boiling range: 40−60 °C was used for chromatography. Size exclusion chromatography (SEC) was carried out using Bio-Rad Bio-Beads S-X1 (40–80 µm bead size). Analytical GPC was carried out using JAIGEL-3H-A (8 × 500 mm) and JAIGEL-4H-A (8 × 500 mm) columns in THF + 1% pyridine as eluent with a flow rate of 1.0 mL/min. Semi-preparative GPC was carried out on a Shimadzu recycling GPC system equipped with a LC-20 AD pump, SPD-20A UV detector and a set either of JAIGEL 3H (20 × 600 mm) and JAIGEL 4H (20 × 600 mm) columns in either toluene + 1% pyridine or THF + 1% pyridine as eluent at a flow rate of 3.5 mL/min.

NMR spectra were recorded on either a Bruker AVIII HD 400, a Bruker AVIII HD 500, a Bruker NEO 600 with a broadband helium cryoprobe, a Bruker AVIII 700 or a Bruker AVIII 950, the latter two both with inverse TCI $^{1}H/^{13}C/^{15}N$ cryoprobes. Chemical shift values are quoted in ppm and coupling constants (J) in hertz to the nearest 0.1 Hz. ^{1}H and ^{13}C NMR spectra are referenced against the residual solvent peak ($CHCl_3$ δ_H = 7.26 ppm, $CDCl_3$ δ_C = 77.16 ppm). Unless stated otherwise, NMR spectra were recorded at 298 K. Diffusion experiments were carried out using the double stimulated echo sequence with bipolar gradients for convection compensation.[1] Diffusion coefficients were obtained by fitting signal intensity decays to the Stejskal-Tanner equation: $I = I_0 \cdot \exp[(\gamma_H \cdot \delta \cdot G)^2 \cdot D \cdot (\Delta - \delta/3)]$, where I and I_0 represent signal areas in the presence and absence of gradient pulses, respectively, D is the diffusion coefficient, G is the gradient strength, γ_H is the ^{1}H magnetogyric ratio, δ is the gradient pulse duration, and Δ is the diffusion time.

Optical spectroscopic measurements were conducted in HPLC grade solvents using fused silica cuvettes (10 mm path length). UV-vis-NIR absorption spectra were acquired on either a Perkin Elmer Lambda 20 spectrometer at 298 K with temperature control by a PTP-1 Peltier unit from Perkin Elmer or a Perkin Elmer Lambda 25 spectrometer at 298 K with a PTP-A Peltier unit from Perkin Elmer.

Porphyrin nanoring aggregates were formed by removal of pyridine from solutions of the nanorings. The porphyrin ring was dissolved in toluene (approx. 4.0 mL per mg of ring) and washed using a sodium acetate buffer solution (pH = 4.6, 4.0 mL per mg of ring × 3), followed by water (4.0 mL per mg of ring × 3), dried over Na_2SO_4, filtered, and concentrated. To remove minor impurities, the nanoring aggregate was further passed through a size-exclusion column (Bio-Beads S-X1, $CHCl_3$, $d \times h$ = 0.5 × 30 cm), followed by a short silica column (SiO_2, approx. $d \times h$ = 0.5 × 6 cm, gradient elution: $CHCl_3$/pentane 3:7 to 5:5) to provide the samples used in this work.

Section 2. Overview of Nanoring Structures

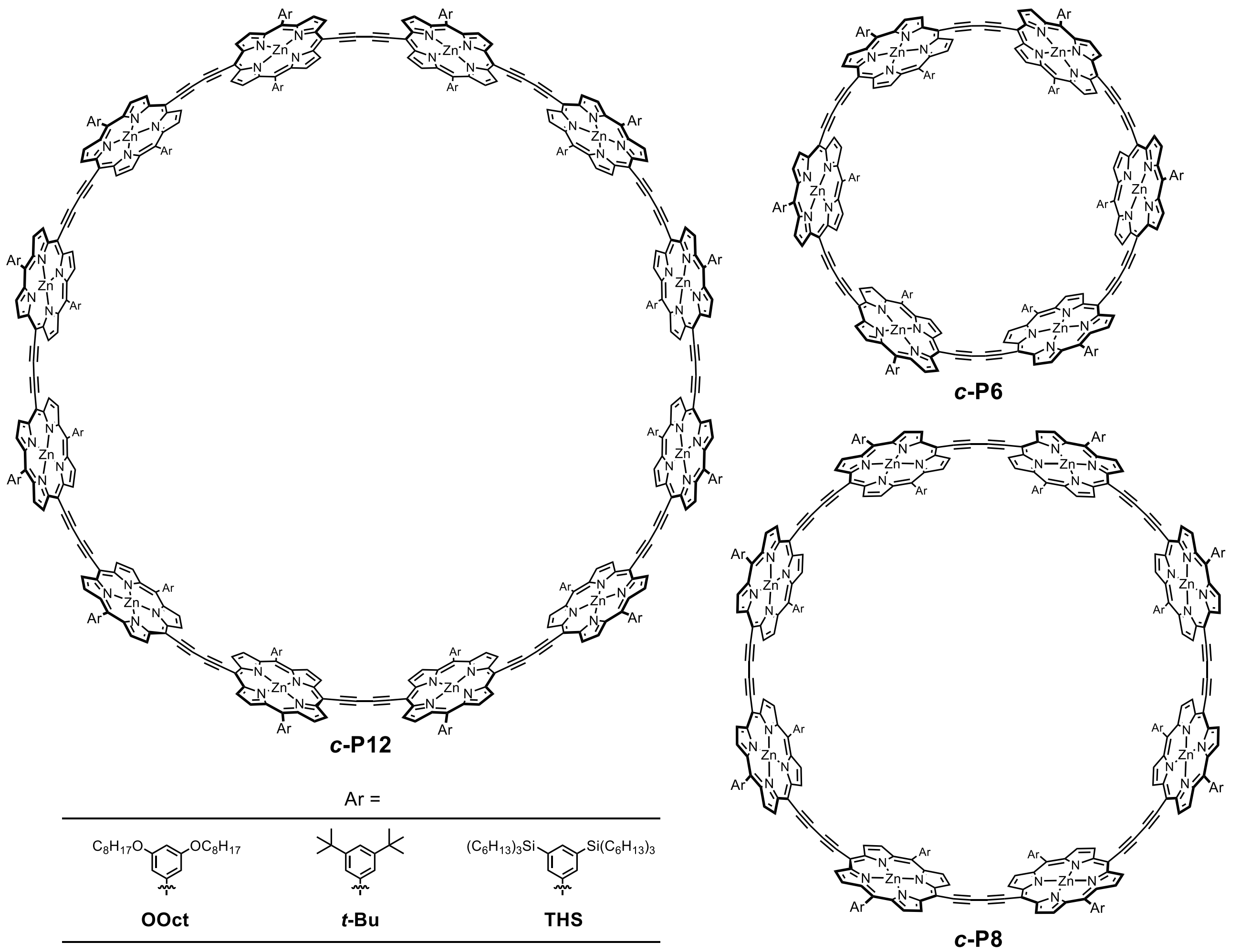

Figure S1. Ring structures included in this study: ***c*-P6**, ***c*-P8**, and ***c*-P12** with 3,5-bis(octyloxy)phenyl (OOct), 3,5-bis(*tert*-butyl)phenyl (*t*-Bu), and 3,5-bis(trihexylsilyl)phenyl (THS) sidechains.

Porphyrin nanorings ***c*-P6 (OOct)**,[1] ***c*-P8 (OOct)**,[2] ***c*-P12 (OOct)**,[3] ***c*-P6 (*t*-Bu)**,[1] ***c*-P8 (*t*-Bu)**,[2] ***c*-P12 (*t*-Bu)**,[3] ***c*-P6 (THS)**,[4] ***c*-P8 (THS)**,[5] and ***c*-P12 (THS)**[6] were synthesized according to published procedures.

Section 3. Overview of ^{1}H NMR Spectra of Nanorings in the Presence and Absence of Pyridine

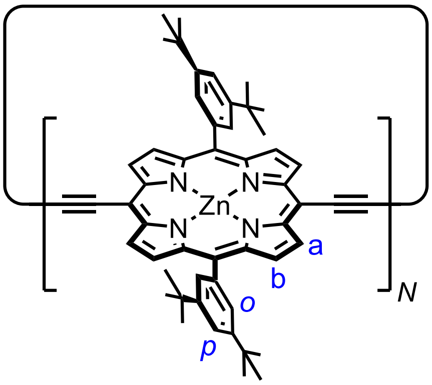

***c*-P*N*$_{t\text{-Bu}}$**
N = 6, 8, 12

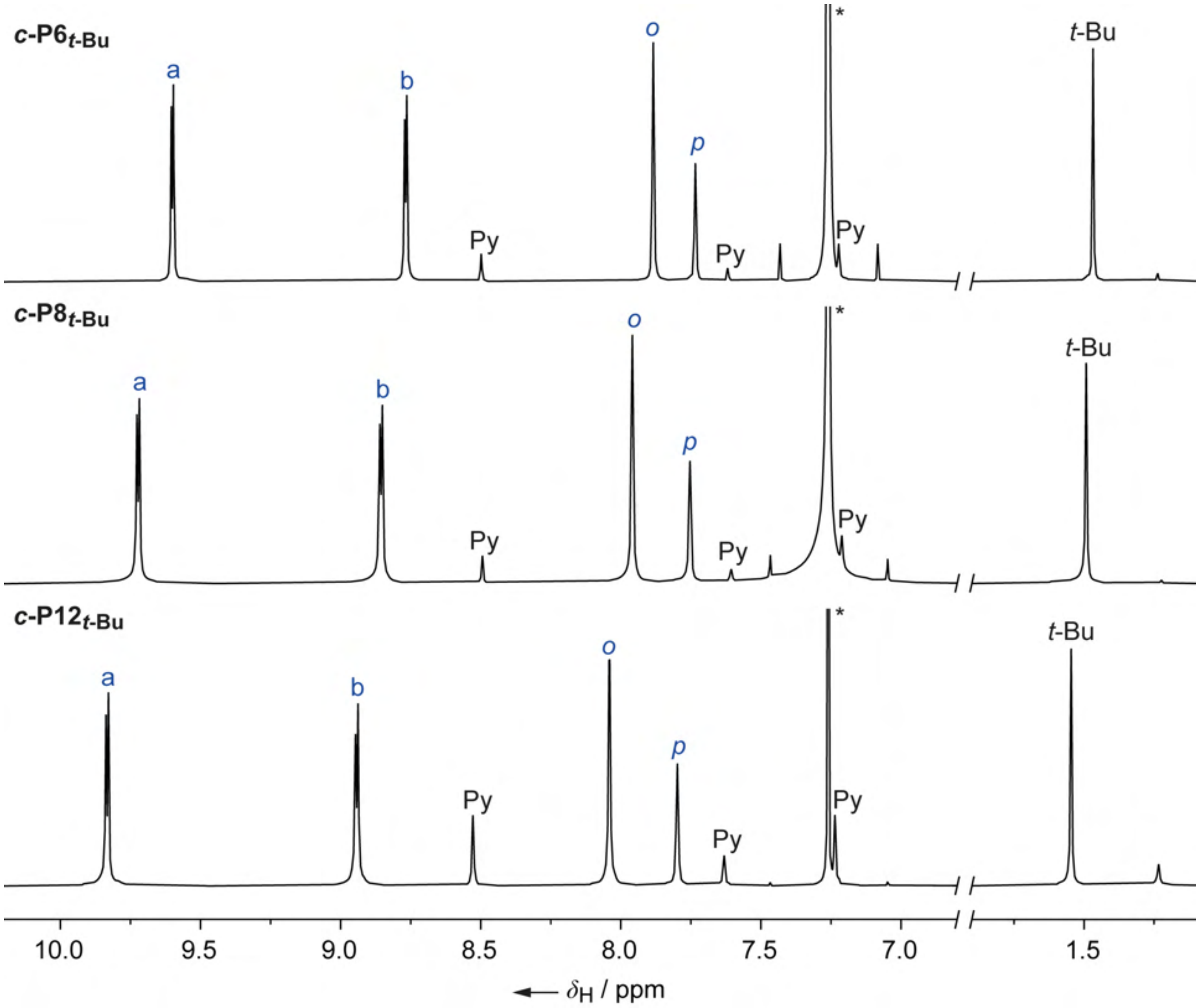

Figure S2. ^{1}H NMR spectra of ***c*-P6**, ***c*-P8**, and ***c*-P12** with *t*-Bu side chains in the presence of 1% pyridine-d_5 (500 MHz, $CDCl_3$, 298 K). * = residual $CHCl_3$; Py = pyridine.

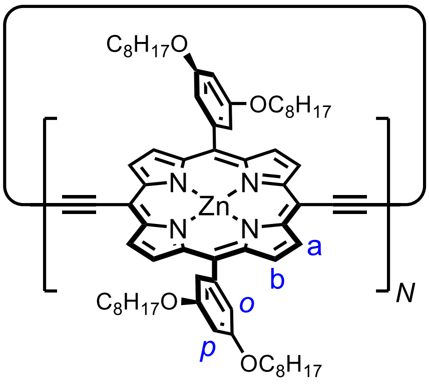

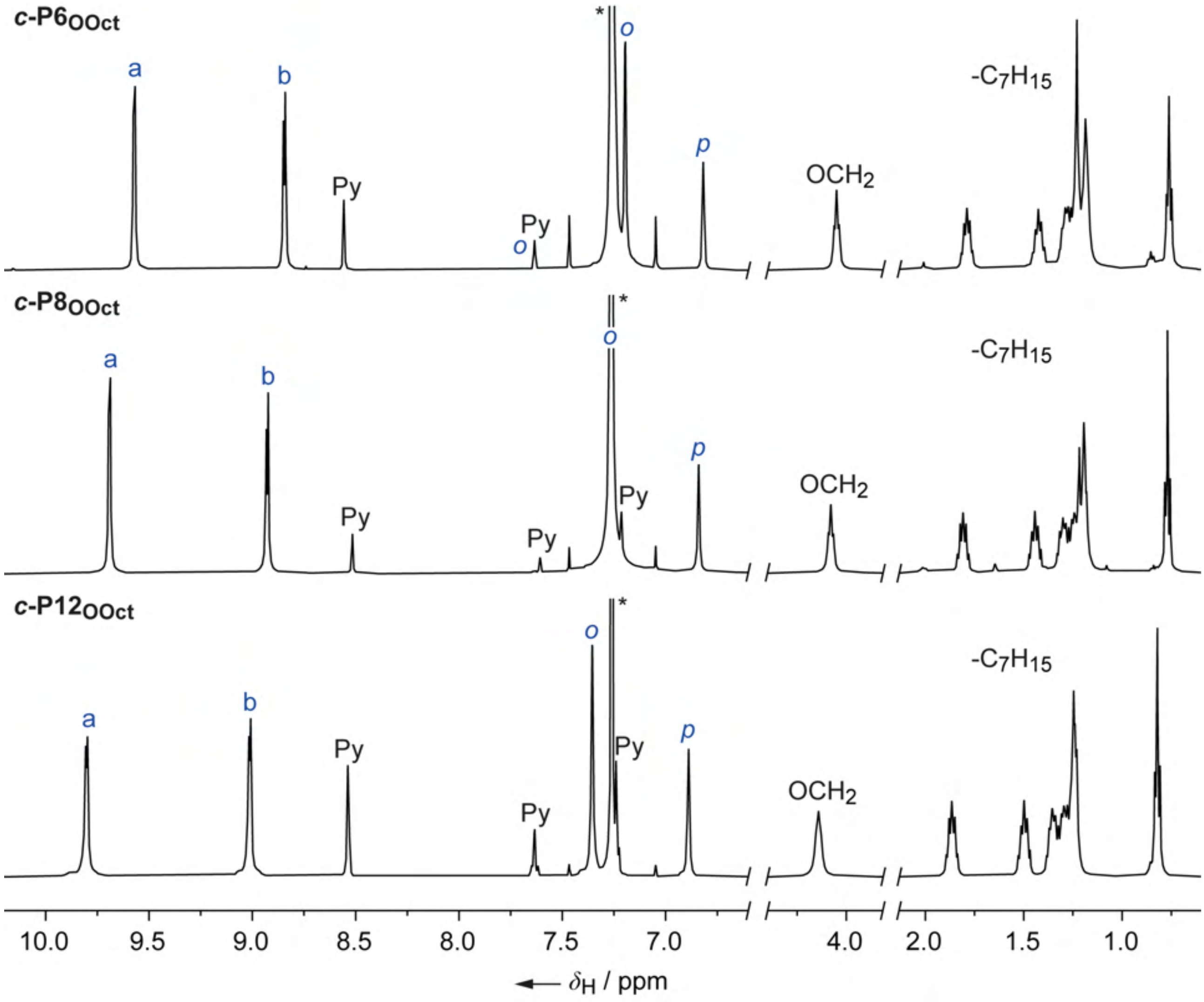

Figure S3. ^{1}H NMR spectra of ***c*-P6**, ***c*-P8**, and ***c*-P12** with OOct sidechains in the presence of 1% pyridine-*d*$_5$ (500 MHz, $CDCl_3$, 298 K). * = residual $CHCl_3$; Py = pyridine.

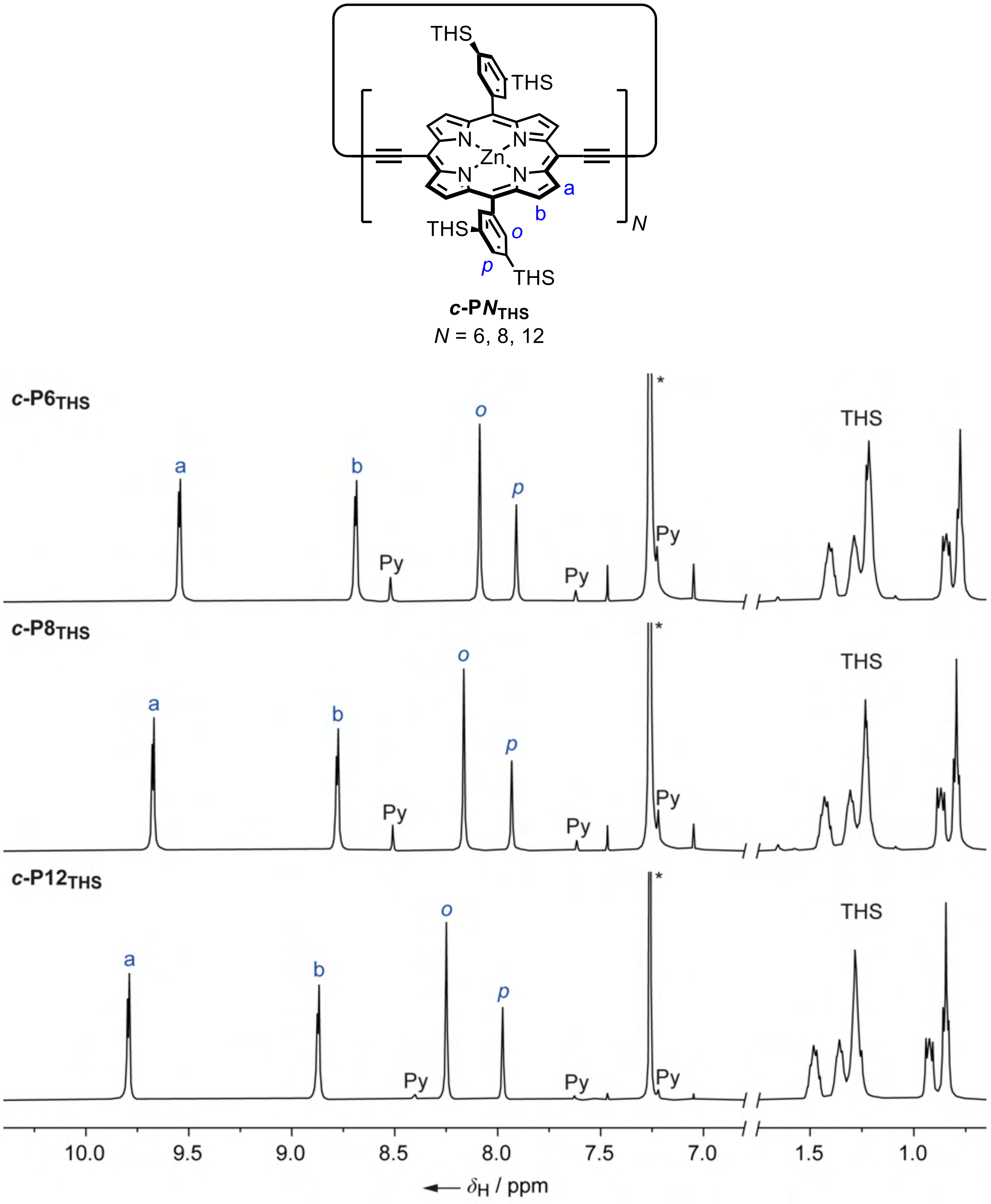

Figure S4. ^{1}H NMR spectra of ***c*-P6**, ***c*-P8**, and ***c*-P12** with THS sidechains in the presence of 1% pyridine-d_5 (500 MHz, $CDCl_3$, 298 K). * = residual $CHCl_3$; Py = pyridine.

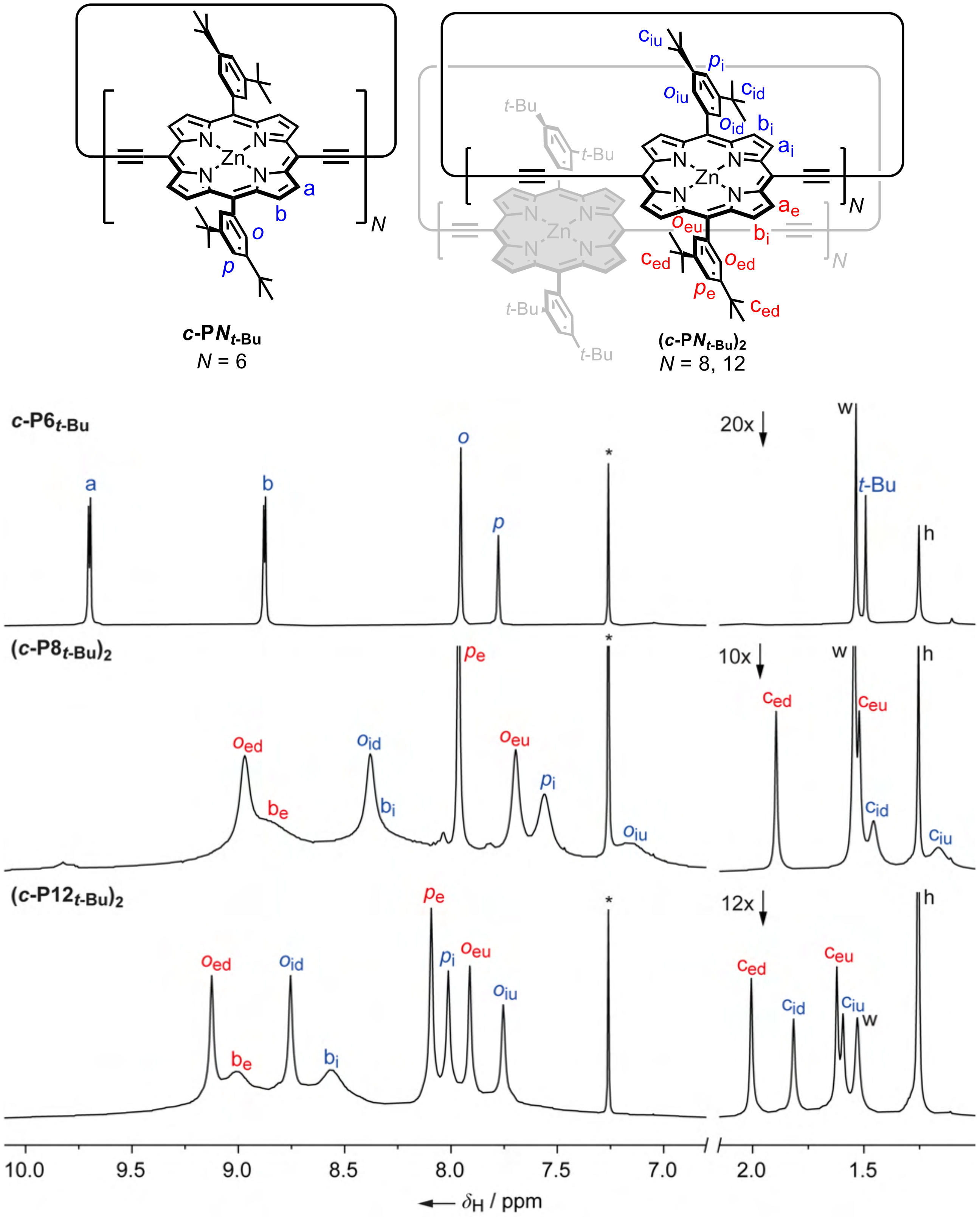

Figure S5. ^{1}H NMR spectra of ***c*-P6**, ***c*-P8**, and ***c*-P12** with *t*-Bu side chains in the absence of pyridine (500 MHz, $CDCl_3$, 298 K). * = residual $CHCl_3$; w = water; h = H-grease.

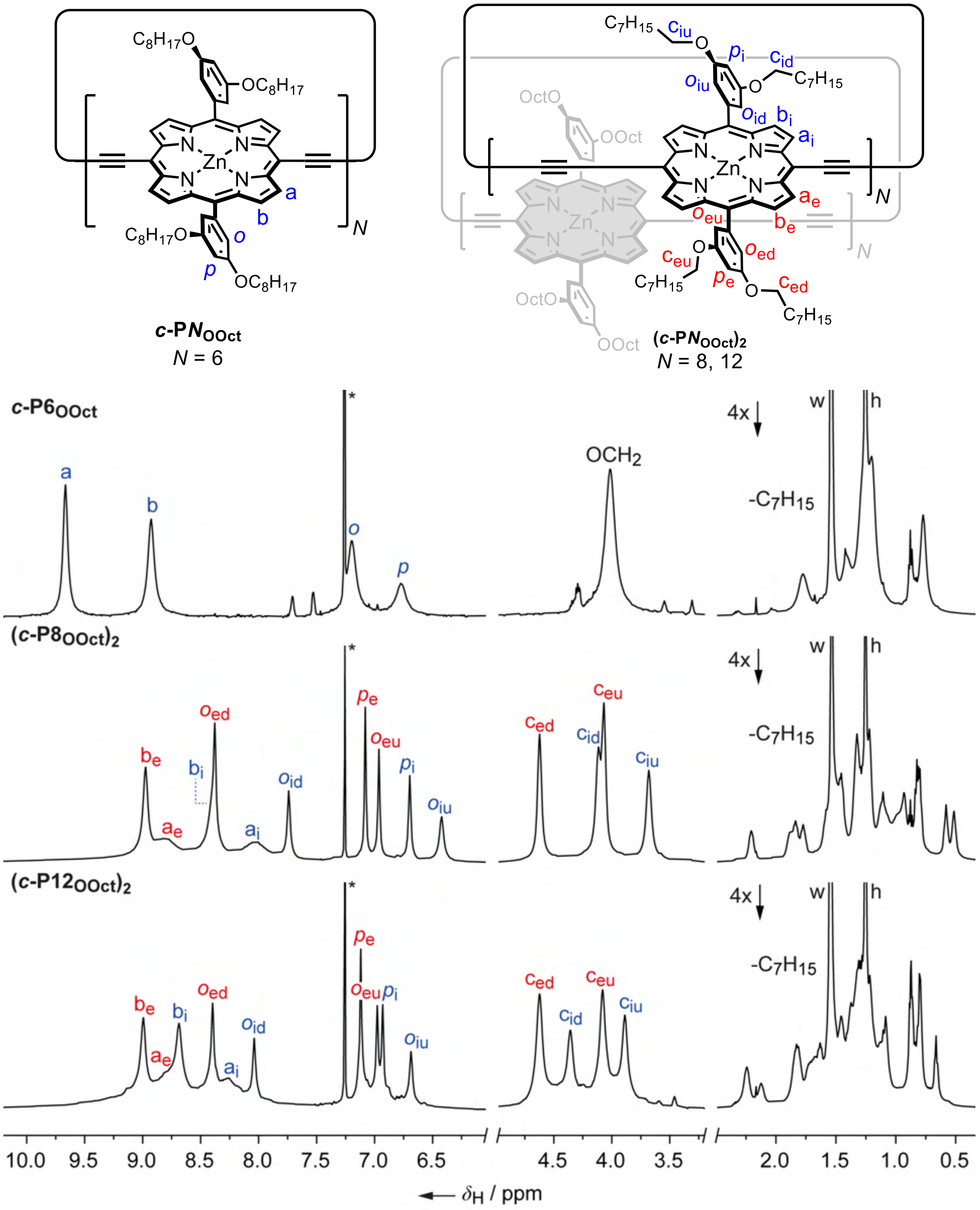

Figure S6. ^{1}H NMR spectra of ***c*-P6**, ***c*-P8**, and ***c*-P12** with OOct sidechains in the absence of pyridine (500 MHz, $CDCl_3$, 298 K). * = residual $CHCl_3$; w = water; h = H-grease.

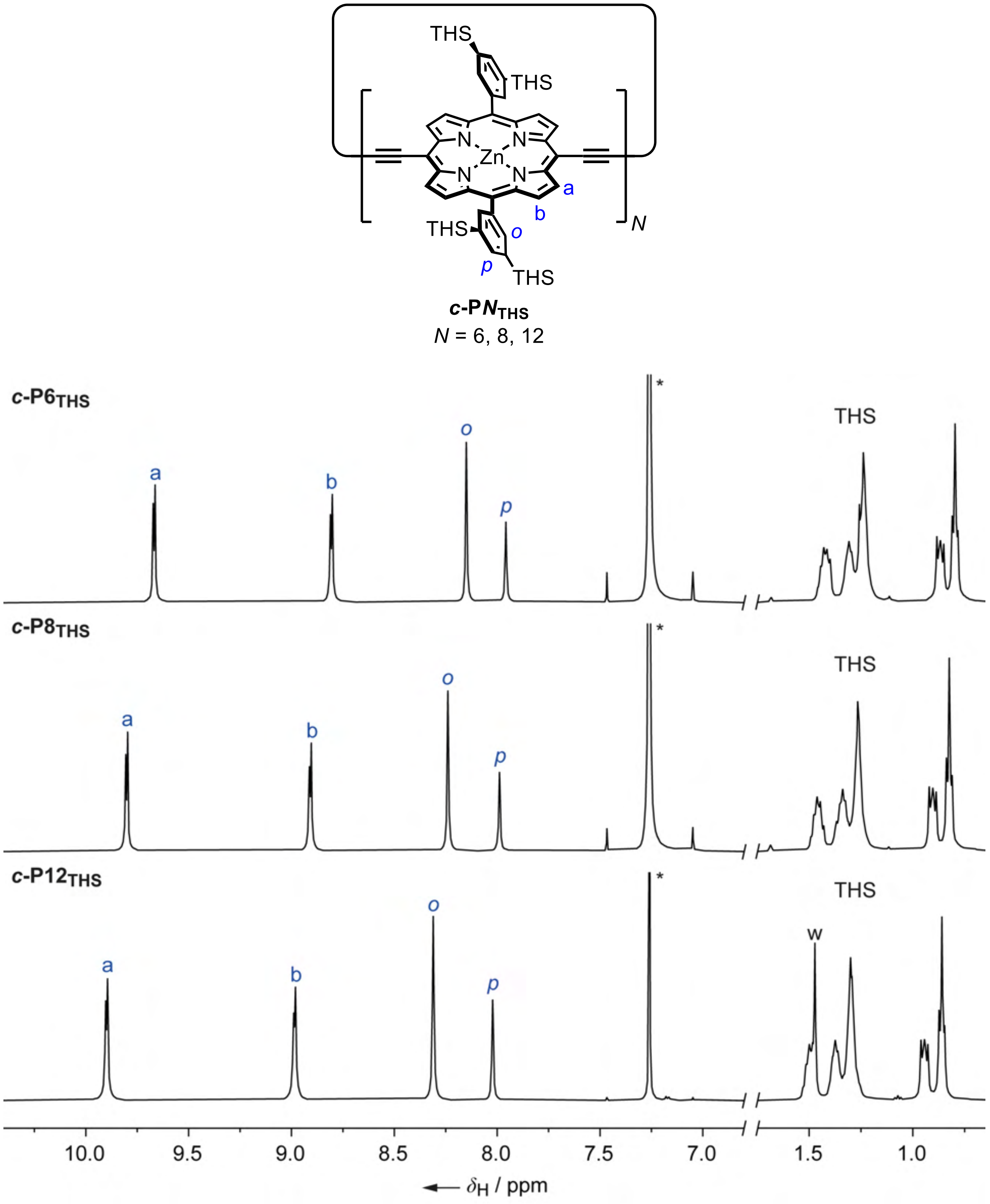

Figure S7. ^{1}H NMR spectra of ***c*-P6**, ***c*-P8**, and ***c*-P12** with THS side chains in the absence of pyridine (500 MHz, $CDCl_3$, 298 K). * = residual $CHCl_3$; w = water.

Section 4. Comparison of HSQC Spectra of Aggregated and Disaggregated Nanorings

As part of assigning the different types of protons observed in dimeric ***c*-P8** and ***c*-P12** nanoring samples, we compared their HSQC signals in the ^{13}C dimension to those of unaggregated samples (i.e., the same rings in the presence of pyridine). The high degree of similarity between monomer and dimer resonances on the ^{13}C chemical shift scale, serve as an indicator of the type of protons in the dimer. That is, by comparison, we can identify 1) the porphyrin beta proton (b; while 'a' is too broad to be observed in the ^{1}H dimension in the dimers), 2) the *ortho* and *para* protons (*o* and *p*), and 3) the sidechain protons (*t*-Bu and -OCH_2-). The actual HSQC spectra used to prepare these plots are included in Section 18.

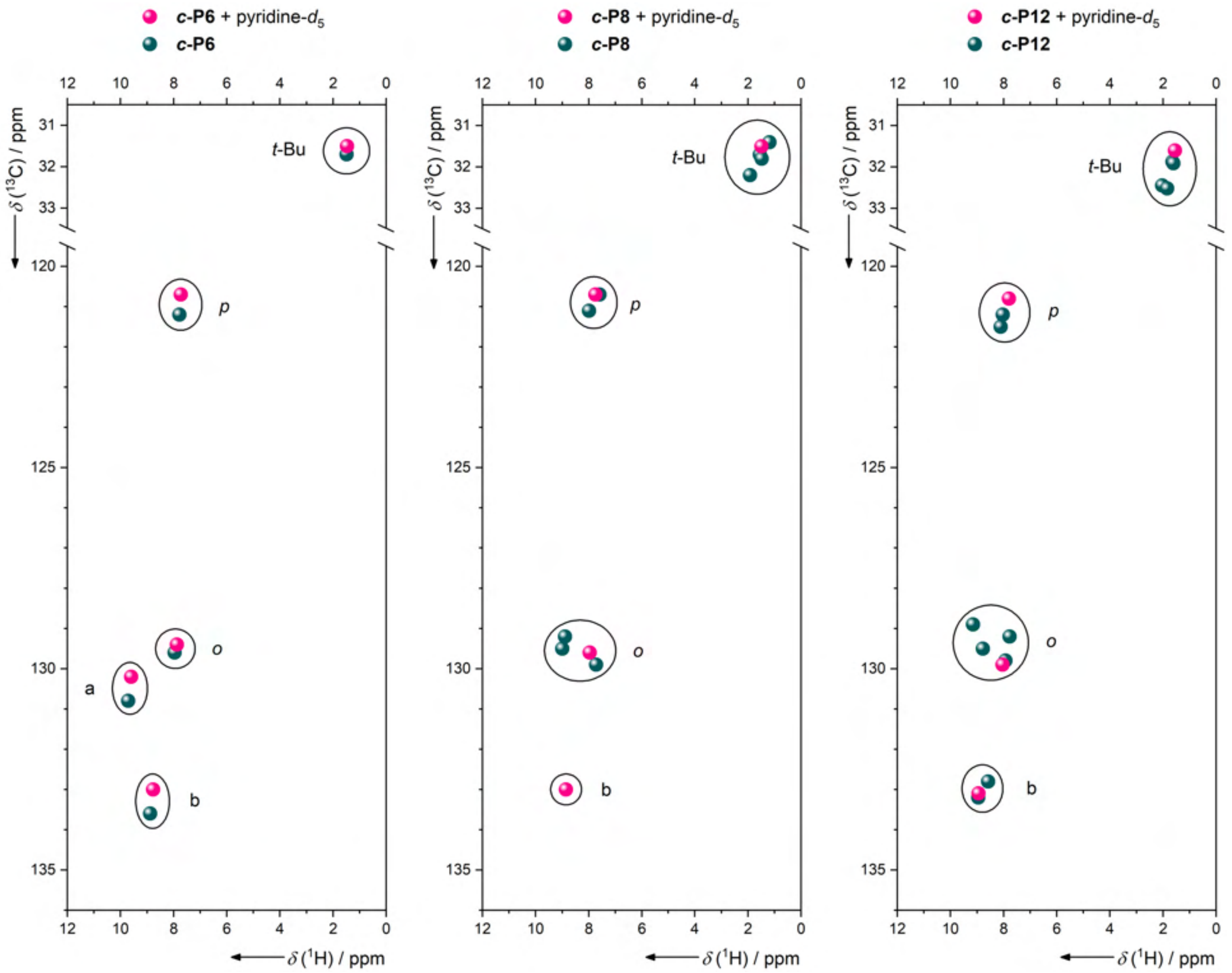

Figure S8. Comparison of the HSQC spectral values of *t*-Bu nanorings (N = 6, 8, 12) in the presence and absence of pyridine-d_5. The high degree of similarity between monomeric and dimeric samples on the ^{13}C chemical shift scale is used as an argument for the assignment of different proton types in the ***c*-P8** and ***c*-P12 (*t*-Bu)** dimers.

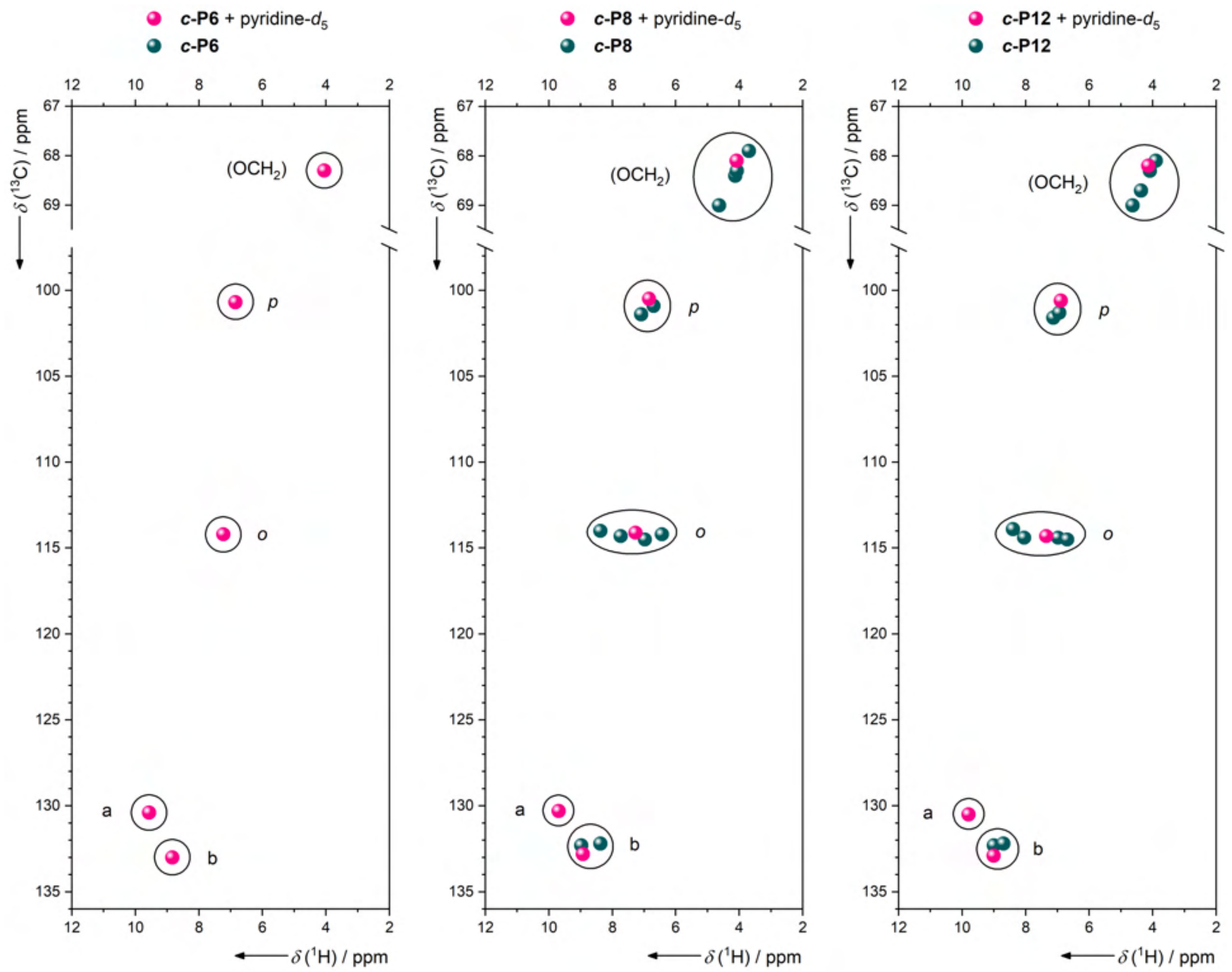

Figure S9. Comparison of the HSQC spectral values of OOct nanorings (N = 6, 8, 12) in the presence and absence of pyridine-d_5. The high degree of similarity between monomeric and dimeric samples on the ^{13}C chemical shift scale is used as an argument for the assignment of different proton types in the ***c*-P8** and ***c*-P12 (OOct)** dimers.

Section 5. EXSY Characterization of Mixtures of Aggregated and Disaggregated Nanorings

NOESY spectra of mixtures of monomer and dimer of ***c*-P8**$_{t\text{-Bu}}$, ***c*-P8**$_{\text{OOct}}$, ***c*-P12**$_{t\text{-Bu}}$, and ***c*-P12**$_{\text{OOct}}$ revealed slow-exchange between the two states (Figure S10). The mixtures were formed by adding pyridine-d_5 to samples of dimeric nanorings. In cases where monomer and dimer signals were sufficiently separated, this allowed us to assign the different types of protons within the dimers, using monomer signals as reference. Overall, the exchange correlations observed in these four cases are consistent with 1) through-space NOESY interactions within monomer and dimer, 2) exchange between monomer and dimer, 3) exchange between the interior and exterior environments of the dimer, which could occur via porphyrin rotation, and 4) NOESY interactions following, either of the two exchange pathways (i.e., stemming from spin-diffusion).

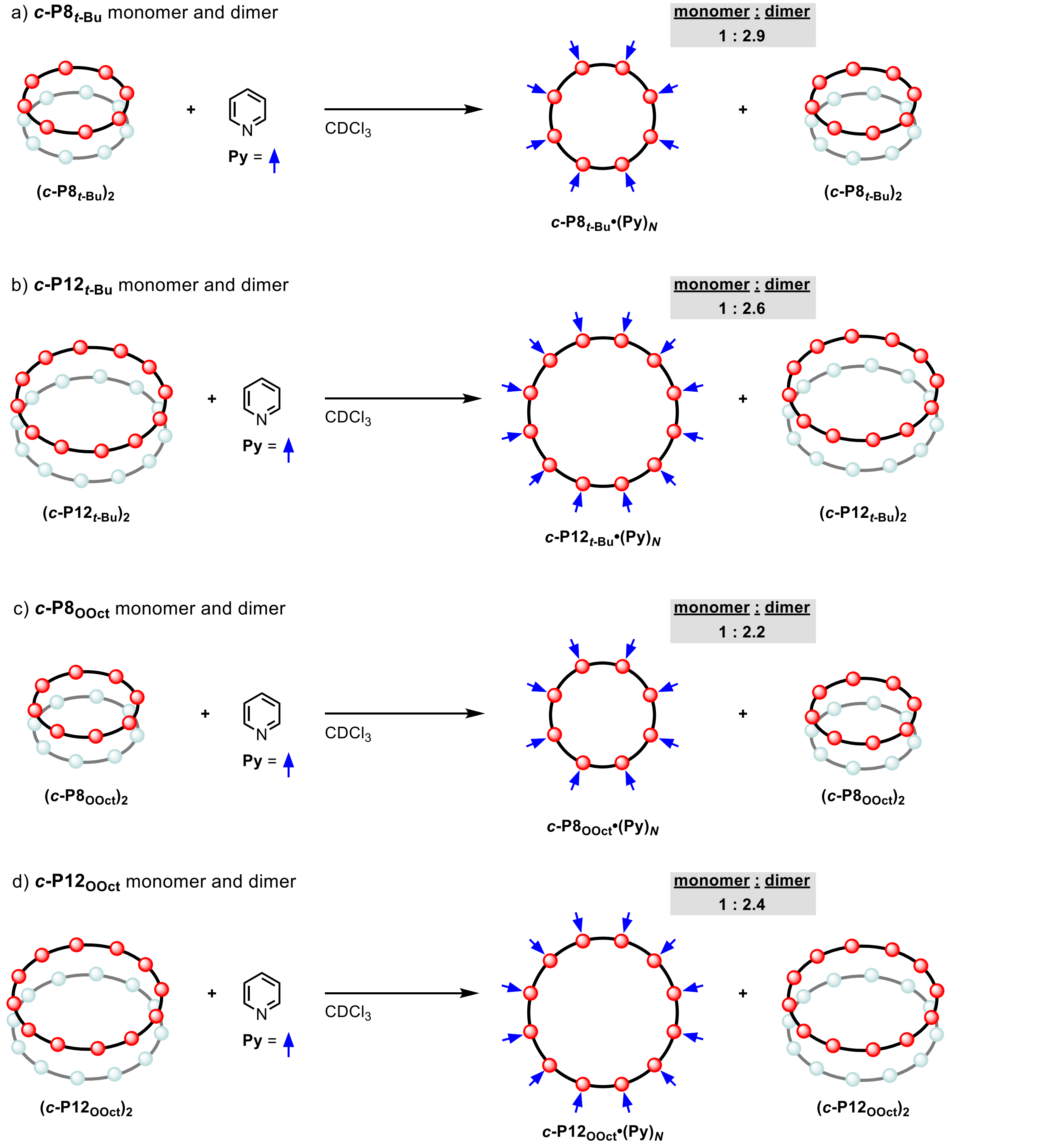

Figure S10. Formation of a mixture of monomer and dimer of nanorings by addition of pyridine-d_5. a) ***c*-P8**$_{t\text{-Bu}}$; b) ***c*-P12**$_{t\text{-Bu}}$; c) ***c*-P8**$_{\text{OOct}}$; d) ***c*-P12**$_{\text{OOct}}$. Ratios indicate compositions of mixtures used in EXSY experiments.

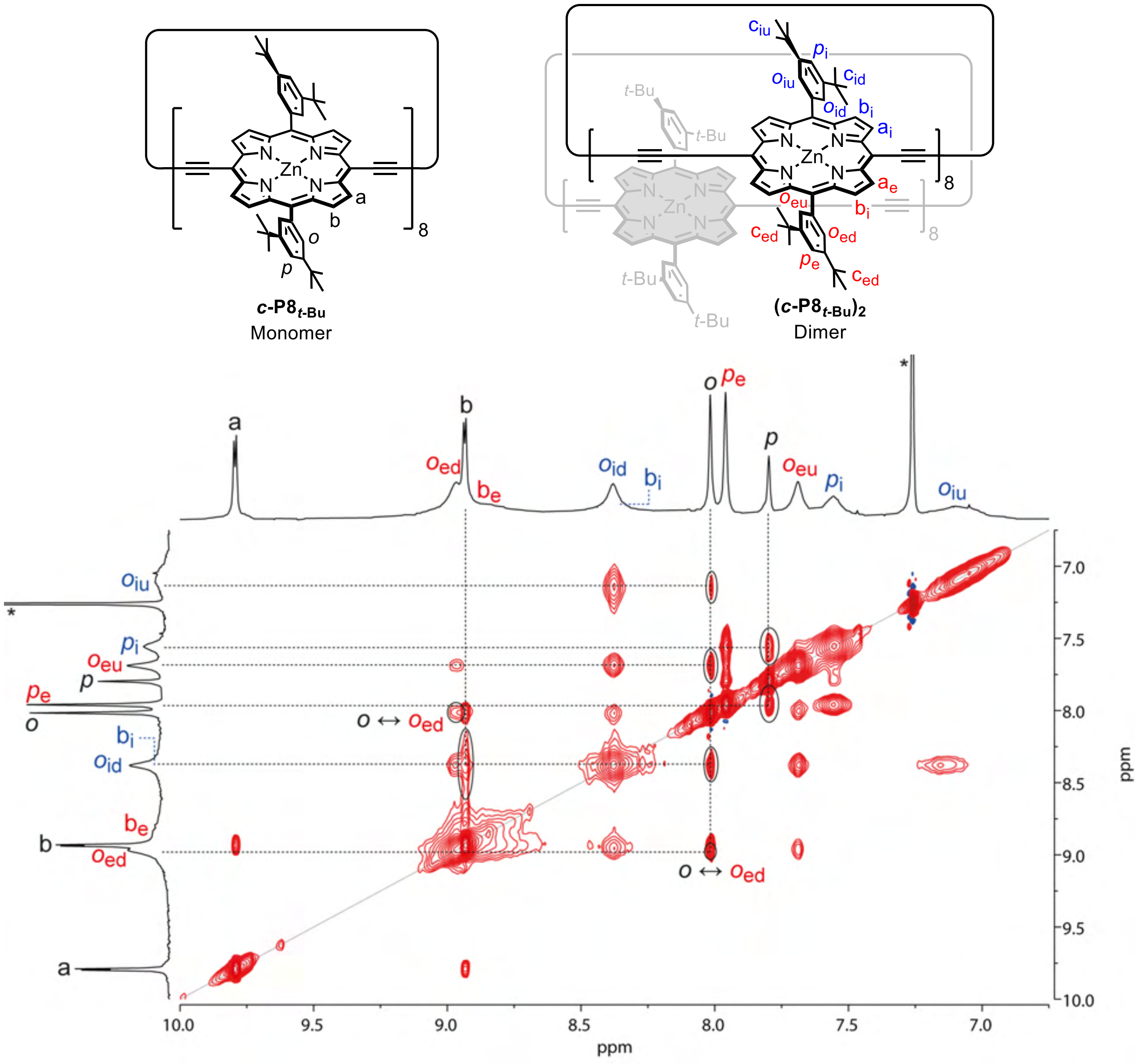

Figure S11. ^{1}H-^{1}H NOESY (500 MHz, $CDCl_3$, 25 °C, t_{mix} = 200 ms) spectrum of a 2.9:1.0 mixture of dimeric and monomeric ***c*-P8*t*-Bu** in the presence of pyridine-d_5. Observed correlations due to exchange between monomer and dimer resonances, allowed us to unambiguously correlate the following dimer signals to known monomer signals: $p \leftrightarrow p_i$, $p \leftrightarrow p_e$, $o \leftrightarrow o_{iu}$, $o \leftrightarrow o_{eu}$, $o \leftrightarrow o_{id}$, $o \leftrightarrow o_{ed}$, and b ↔ b_i. It is expected that monomer-dimer exchange also contributes to the magnitude of other cross-peaks (i.e., b ↔ b_e), but due to overlap in the ^{1}H dimension, it is difficult to discern these unambiguously.

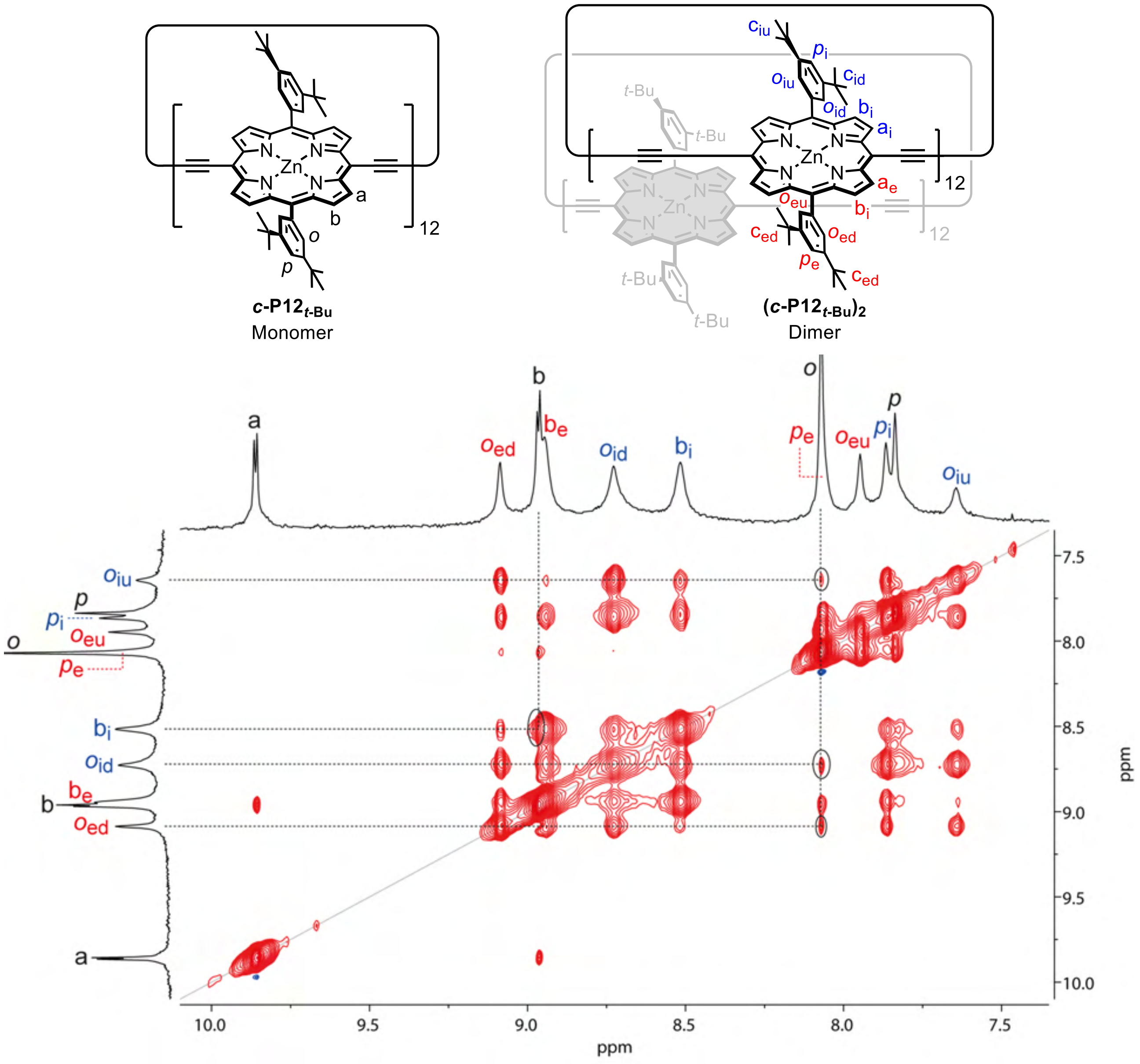

Figure S12. ^{1}H-^{1}H NOESY (500 MHz, CDCl$_3$, 50 °C, t_{mix} = 200 ms) spectrum of a 2.6:1.0 mixture of dimeric and monomeric ***c*-P12**$_{t\text{-Bu}}$ in the presence of pyridine-d_5. Due to the large extent of overlap between monomer and dimer signals, it is difficult to discern any correlations without ambiguity. However, it seems probable that the following correlations are correct: $o \leftrightarrow o_{iu}$, $o \leftrightarrow o_{id}$, and $o \leftrightarrow o_{ed}$, because in the case of ***c*-P8**$_{t\text{-Bu}}$ and ***c*-P8**$_{OOct}$, p_e only correlates to p and p_i, and this also looks to be the case in ***c*-P12**$_{OOct}$, although o_{eu} overlaps with p and p_i. In addition, the correlation of b $\leftrightarrow$ b_i appears to be present, although it is difficult to discern, because of the close-spacing between b and b_e, both of which seem to be correlating to b_i. The correlation $b_i \leftrightarrow b_e$ has to be via a different exchange pathway, for example, via porphyrin rotation, which may occur in the presence of pyridine.

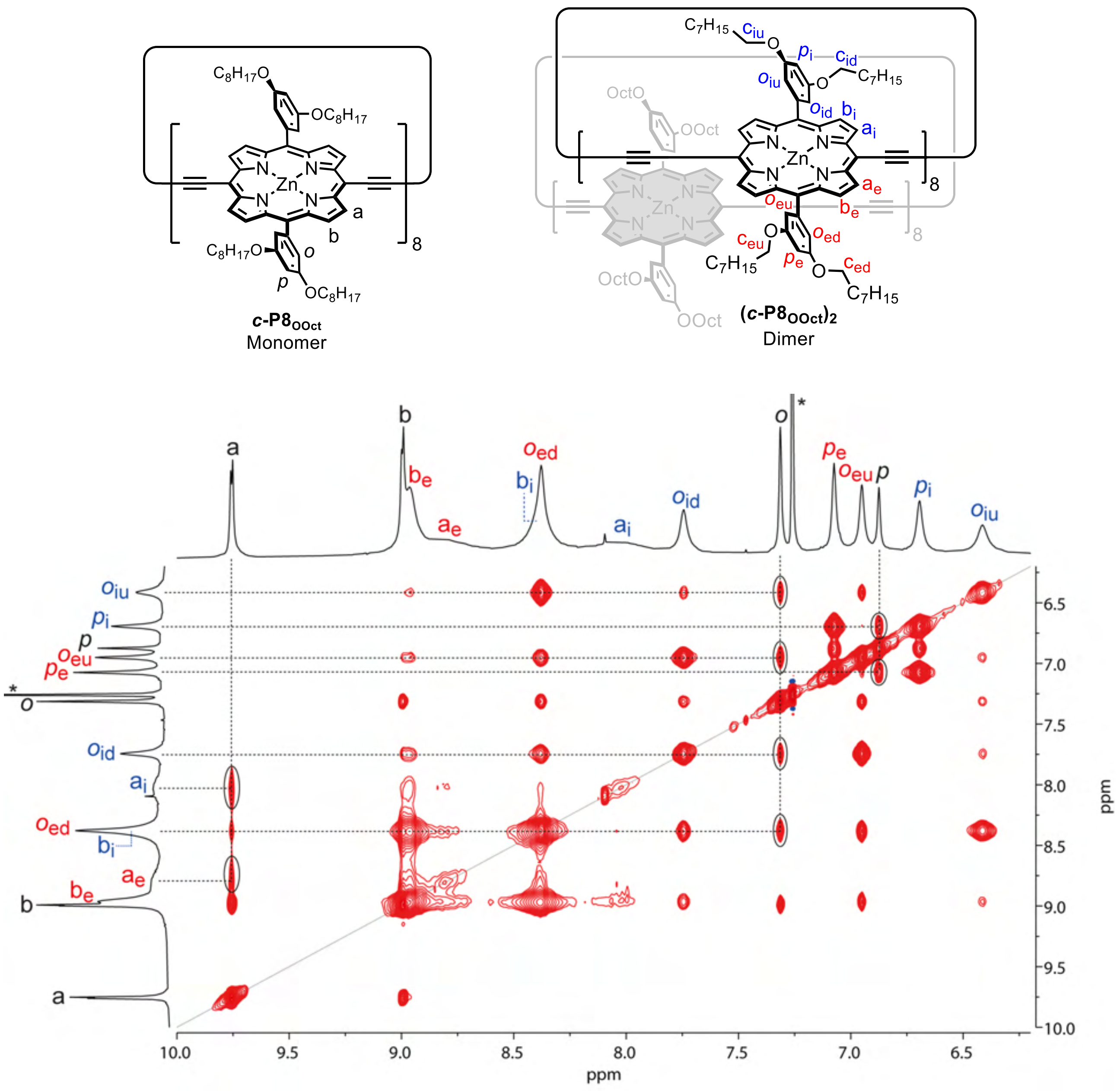

Figure S13. ^{1}H-^{1}H NOESY (500 MHz, $CDCl_3$, 25 °C, t_{mix} = 200 ms) spectrum of a 2.2:1.0 mixture of dimeric and monomeric ***c*-P8$_{OOct}$** in the presence of pyridine-d_5. Observed correlations due to exchange between monomer and dimer resonances, allowed us to unambiguously correlate the following dimer signals to known monomer signals: $p \leftrightarrow p_i$, $p \leftrightarrow p_e$, $o \leftrightarrow o_{iu}$, $o \leftrightarrow o_{eu}$, $o \leftrightarrow o_{id}$, $o \leftrightarrow o_{ed}$, a ↔ a_i, and a ↔ a_e. It is expected that monomer-dimer exchange also contributes to the magnitude of other cross-peaks (i.e., b_i and b_e), but due to overlap in the ^{1}H dimension, this contribution cannot be seen or unambiguously discerned from that of transfer mechanisms involving through-space dipolar interactions.

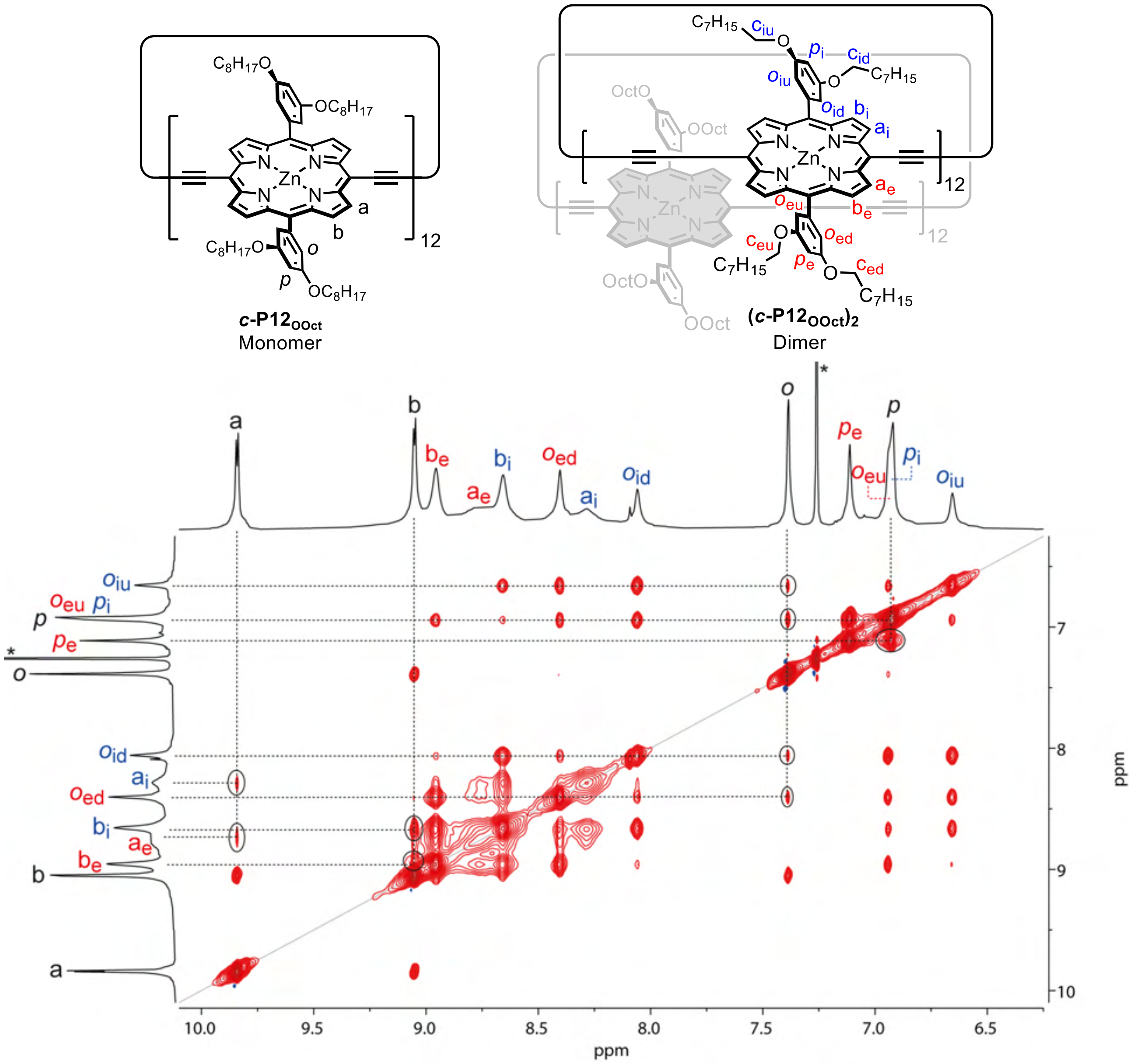

Figure S14. ^{1}H-^{1}H NOESY (500 MHz, $CDCl_3$, 50 °C, t_{mix} = 200 ms) spectrum of a 2.4:1.0 mixture of dimeric and monomeric ***c*-P12$_{OOct}$** in the presence of pyridine-d_5. Observed correlations due to exchange between monomer and dimer resonances, allowed us to unambiguously correlate the following dimer signals to known monomer signals: $o \leftrightarrow o_{iu}$, $o \leftrightarrow o_{id}$, $o \leftrightarrow o_{ed}$, b ↔ b_i, b ↔ b_e, a ↔ a_i, and a ↔ a_e. It is expected that monomer-dimer exchange also contributes to the magnitude of other cross-peaks (i.e., p_i, p_e, and o_{eu}), but due to overlap in the ^{1}H dimension, this contribution cannot be seen or unambiguously discerned from that of transfer mechanisms involving through-space dipolar interactions or other exchange pathways. In the case of ***c*-P8$_{OOct}$**, the $o \leftrightarrow p$ correlation was not observed, which suggests the NOESY contribution is small and the $o \leftrightarrow$ (o_{eu}, p, p_i) cross-peak is likely mainly due to monomer-dimer exchange (i.e., $o \leftrightarrow o_{eu}$).

Section 6. Comments on the ^{1}H NMR Resonance Assignment

To demonstrate consistency between the model of a planarized dimer and the observed NMR data in the absence of pyridine, we set out to make a full assignment of the observed signals, most thoroughly in the case of ***c*-P12$_{t\text{-Bu}}$**.

Later, the assigned protons were used to correlate between observed resonances and model protons as we performed structural analyses based on 1) distances from NOESY and 2) residual dipolar coupling (RDC) values from ^{1}H-^{13}C coupled HSQC. Using the dimer model, both NOESY- and RDC analyses gave good fits to values from experiment.

A model of the ***c*-P12$_{t\text{-Bu}}$** dimer is shown below (Figure S15). This model was made using structural parameters derived from an xTB minimized geometry (Section 7, Figure S19). The point group symmetry of the ring dimer is similar to staggered ferrocene which is D_{5d}. The ring aggregate is D_{12d} (one principle C_{12} axis, twelve perpendicular C_2 axes, and twelve σ_d mirror planes). Each of the porphyrin units can be interconverted by improper rotation about the S_{24} axis. At room temperature and above, its ^{1}H NMR features is most consistent with that of a weighted average between two conformers interconverting via ring rotation in opposite directions. Through this process, each porphyrin unit essentially breaks and reforms a π-aggregate with its two nearest neighbors of the opposing ring. This is suggested from the intermediate-fast exchange of beta porphyrin protons observed at room temperature and above. At lower temperatures (around –50 °C), this process slows down, leading to a desymmetrization of the porphyrin units, i.e., it becomes possible to observe all 8 porphyrin beta protons.

Dimer aggregation gives rise to four different types of proton environments that need to be distinguished. Firstly, these include interior vs. exterior environments, meaning inside and outside the circumferences of the nanorings, and these environments have been labelled using subscripts of ‘i’ for interior and ‘e’ for exterior. Secondly, these include environments that are close to and far from the central interface of the aggregate, and these have arbitrarily been labelled using subscripts of ‘d’ for down (close to the interface) and ‘u’ for up (far from the interface). Both *ortho* and *t*-Bu resonances are split by these environments. For example, the *ortho* resonance on the exterior side, close to the interface (and pointing *down* relative to the top ring), is labelled o_{ed} (*ortho* exterior down). On the other hand, porphyrin beta protons and *para* protons are only split into interior and exterior environments since they are in the same plane as the porphyrin. In addition, at low temperature, the back-and-forth shifting of porphyrin units occurs sufficiently slow to cause a desymmetrization of the porphyrin units. This leads to further splitting of the interior and exterior porphyrin environments, giving in total four beta proton environments, i.e., it becomes possible to distinguish between the side of the porphyrin that is engaged in π–π interactions with the neighboring porphyrin and the porphyrin side which instead is above the butadiyne bridge of the opposing ring. Around room temperature, the ^{1}H NMR spectrum is simpler, because the shifting process is faster, and as a result, the porphyrin appears symmetrical due to conformational averaging. Around room temperature, $a_{i1} = a_{i2}$ (simplified as a_i), $b_{i1} = b_{i2}$ (simplified as b_i), $a_{e1} = a_{e2}$ (simplified as a_e), and $b_{e1} = b_{e2}$ (simplified as b_e).

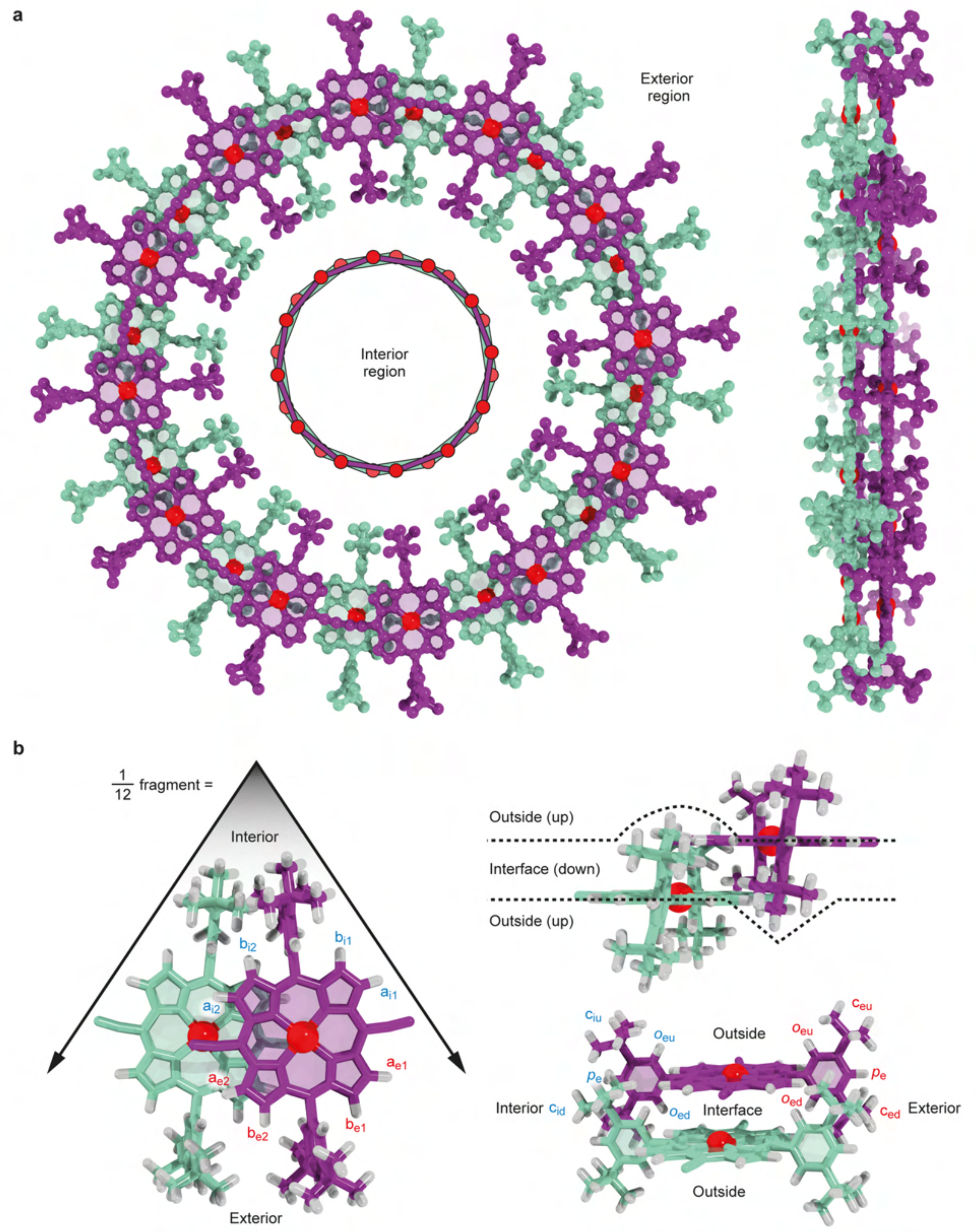

Figure S15. Dimer model of **(*c*-P12$_{t\text{-Bu}}$)$_2$**. a) Full model of D_{12d} symmetry. Colors (purple and cyan) are used to distinguish between the two rings. The two planarized rings are separated by 3.4 Å and rotated by 2.8° degrees from a perfect staggered conformation. Hydrogen atoms omitted for clarity. b) A 1/12 fragment of the dimer with the main four proton environments highlighted (interior vs. exterior, and interface vs. outside). Subscripts are used to distinguish between these environments: i = interior, e = exterior, and chosen arbitrarily, d = down (interface), and u = up (outside).

Size of the aggregate

From diffusion-ordered NMR alone, it is evident that the aggregate structure is discrete and of a size not substantially different to that of its monomer units. The possibilities of it being a dimer or trimer seem most probable. The actual diffusion coefficient of the **(*c*-P12*t*-Bu)2** aggregate ($D = 1.45{\cdot}10^{-10}$ m^{2}s^{-1}) is only marginally smaller than that of its monomer, ***c*-P12*t*-Bu**, ($D = 1.62{\cdot}10^{-10}$m^{2}s^{-1}). Both diffusion coefficients measured in $CDCl_3$ at 298 K (Section 17).

Features of the ^{1}H NMR of the aggregate are most consistent with those expected of a dimer. For a dimeric model of interdigitated rings, only one porphyrin environment is expected (i.e., the protons on any porphyrin are enough to represent all environments of the structure). On the other hand, for a trimeric model of interdigitated rings, two porphyrin environments are needed to encompass all proton environments – in a 1:2 ratio for the porphyrins in the inner and outer layers, respectively. Any porphyrin in a dimer or trimer model will include beta protons ('a' and 'b') and sidechain protons (*ortho* = '*o*', *para* = '*p*', and *t*-Bu = 'c' in this case). What is observed in the ^{1}H NMR of the aggregate is completely in agreement with a single porphyrin environment as expected of a dimer.

The measurement of more ring systems adds further support to a dimeric model. In the absence of pyridine, the ***c*-P8*t*-Bu**, ***c*-P8OOct**, and ***c*-P12OOct** rings all presented ^{1}H NMR features similar to that of the ***c*-P12*t*-Bu** aggregate, consistent with a one-type of porphyrin environment. For some rings, their measured diffusion coefficient was even slightly higher than that of the monomer: (***c*-P8*t*-Bu**: $D = 1.89{\cdot}10^{-10}$ m^{2}s^{-1} (aggregate) vs $D = 1.79{\cdot}10^{-10}$ m^{2}s^{-1} (monomer); ***c*-P8OOct**: $D = 1.73{\cdot}10^{-10}$ m^{2}s^{-1} (aggregate) vs $D = 2.04{\cdot}10^{-10}$ m^{2}s^{-1} (monomer); ***c*-P12OOct**: $D = 1.47{\cdot}10^{-10}$ m^{2}s^{-1} (aggregate) vs $D = 1.38{\cdot}10^{-10}$ m^{2}s^{-1} (monomer)).

Altogether this is consistent with a dimeric structure. The possibility of having the rings arranged in an interdigitated manner, explains how these aggregates obtain their compact structure, relative to their monomer rings. An overview of ^{1}H-NMR spectra is presented in Section 3. All diffusion data is presented in Section 17.

Type of protons

The assignment of proton types in the aggregates is best viewed through HSQC experiments, using the monomer ring in the presence of pyridine as a reference. We found very little change in ^{13}C chemical shift values, comparing carbon atoms directly bonded to the same type of proton in the aggregate or monomer. All ^{1}H signals, sufficiently well-resolved, of the aggregates could be correlated in the ^{13}C dimension to a known proton type in the monomer (see Section 4). To a variable extent, depending on the ring and degree of signal overlap, proton types were also confirmed via direct ^{1}H signal correlation between aggregate and monomer. EXSY spectroscopy of monomer-dimer mixtures revealed cross-peaks stemming from slow-exchange between the two, which enabled the assignment of some aggregate signals. Yet, because of significant signal overlap in the ^{1}H dimension, it is not straightforward to correlate dimer and monomer resonances in ***c*-P12*t*-Bu**. In the case of ***c*-P8*t*-Bu**, ***c*-P8OOct**, and ***c*-P12OOct**, the dispersion of signals turned out to be much more optimal, and as a result, proton correlations between monomer and dimer could be made without ambiguity (see Section 5).

Grouping of resonances into two spin environments (interior and exterior)

Through 2D COSY, TOCSY, and NOESY, two spin environments were identified, which have been labelled as the 'interior' and the 'exterior' environments. The interior spin environment consists of: b_i, o_{id}, p_i, o_{iu}, c_{id}, and c_{iu}, while the exterior spin system environment includes: b_e, o_{id}, p_i, o_{iu}, c_{id}, and c_{iu}. Yet, at this point the interior/exterior grouping of resonances is arbitrary and only with NOESY was it possible to make this assignment (see below). COSY and TOCSY revealed expected *J*-coupling correlations between *ortho*- and *para*- protons, helping to identify these proton types within the interior and exterior groups (Figure S16 and S17 below). In addition, COSY and TOCSY showed unexpected long-range correlations reminiscent of NOE type correlations. First, *t*-Bu type protons coupling with *ortho*- or *para*- type protons from their spin environment (formally a five-bond coupling). Secondly, although only observed in TOCSY, *t*-Bu protons coupling with beta protons ($b_e \leftrightarrow c_{ed}$ and $b_i \leftrightarrow c_{id}$), which is formally a nine-bond coupling. Given it was later

confirmed that the aggregate partially aligns spontaneously in solution in the presence of a magnetic field (Section 13), it would be reasonable for these long-range correlations to originate from dipolar interactions. Interestingly, this coupling between *t*-Bu and beta protons, was only observed for two out of the four *t*-Bu signals, despite the fact, that the intramolecular distance between *t*-Bu and beta protons is the same for any *t*-Bu group. It would seem likely, this correlation is due to a dipolar interaction across the aggregate interface and that the two active *t*-Bu groups (c_{ed} and c_{id}) therefore must be oriented towards it, while the remaining two *t*-Bu groups (c_{eu} and c_{iu}), for which no correlation is seen, are oriented away from it (i.e., they are on the surface of the aggregate).

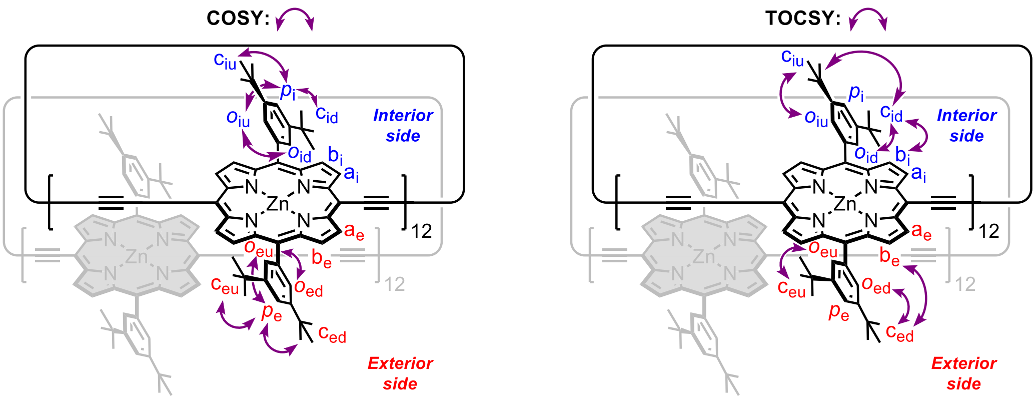

Figure S16. Correlations observed in COSY (left) and additional correlations observed in TOCSY (right). All correlations seen in COSY are also observed in TOCSY.

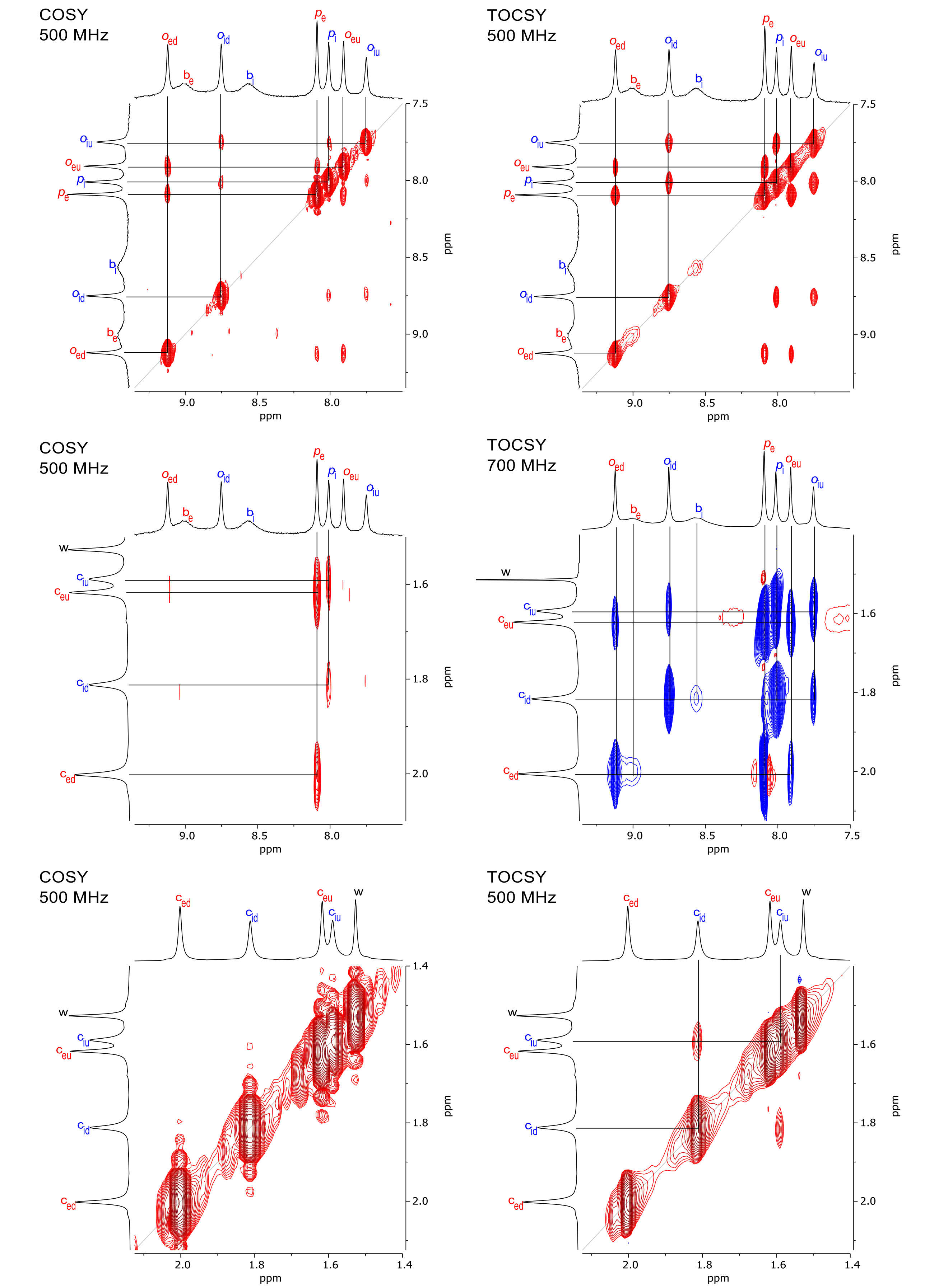

Figure S17. Selected regions of COSY (left) and TOCSY (right) spectra of **(*c*-P12$_{t\text{-Bu}}$)$_2$** (500 MHz, $CDCl_3$, 298 K). Abbreviations: w = water.

Assignment of the *t*-Bu and beta protons to the interior and exterior spin environments was further supported by 2D NOESY, by identifying their correlations with interior/exterior *ortho*- or *para*- protons. We did not observe any NOE correlations across the interior and exterior environments.

The interior spin environment of o_{id}, p_{i}, and o_{iu} can be linked to porphyrin proton b_{i} and *t*-Bu protons c_{id} and c_{iu}, and similarly, the exterior spin environment of o_{id}, p_{i}, and o_{iu} can be linked to porphyrin proton b_{e} and *t*-Bu protons c_{ed} and c_{eu}. Beta proton b_{i} correlates to aryl sidechain protons from the interior spin system (o_{id}, p_{i}, o_{iu}), while b_{e} correlates only to aryl sidechain protons from the exterior spin system (o_{id}, o_{iu}) (Figure S18, left). Likewise, it can be seen that *t*-Bu protons c_{id} and c_{iu} correlates with the same aryl sidechain protons from the interior spin system (o_{id}, p_{i}, o_{iu}), whereas c_{ed} and c_{eu} correlate to the same aryl sidechain protons from the exterior spin system (o_{id}, p_{i}, o_{iu}) (Figure S18, right). Correlations between porphyrin and *t*-Bu protons from the interior region ($b_{i} \leftrightarrow c_{id}$, $b_{i} \leftrightarrow c_{iu}$) can also be seen (Figure S18, right), and vice versa for the exterior region ($b_{e} \leftrightarrow c_{ed}$, $b_{e} \leftrightarrow c_{eu}$).

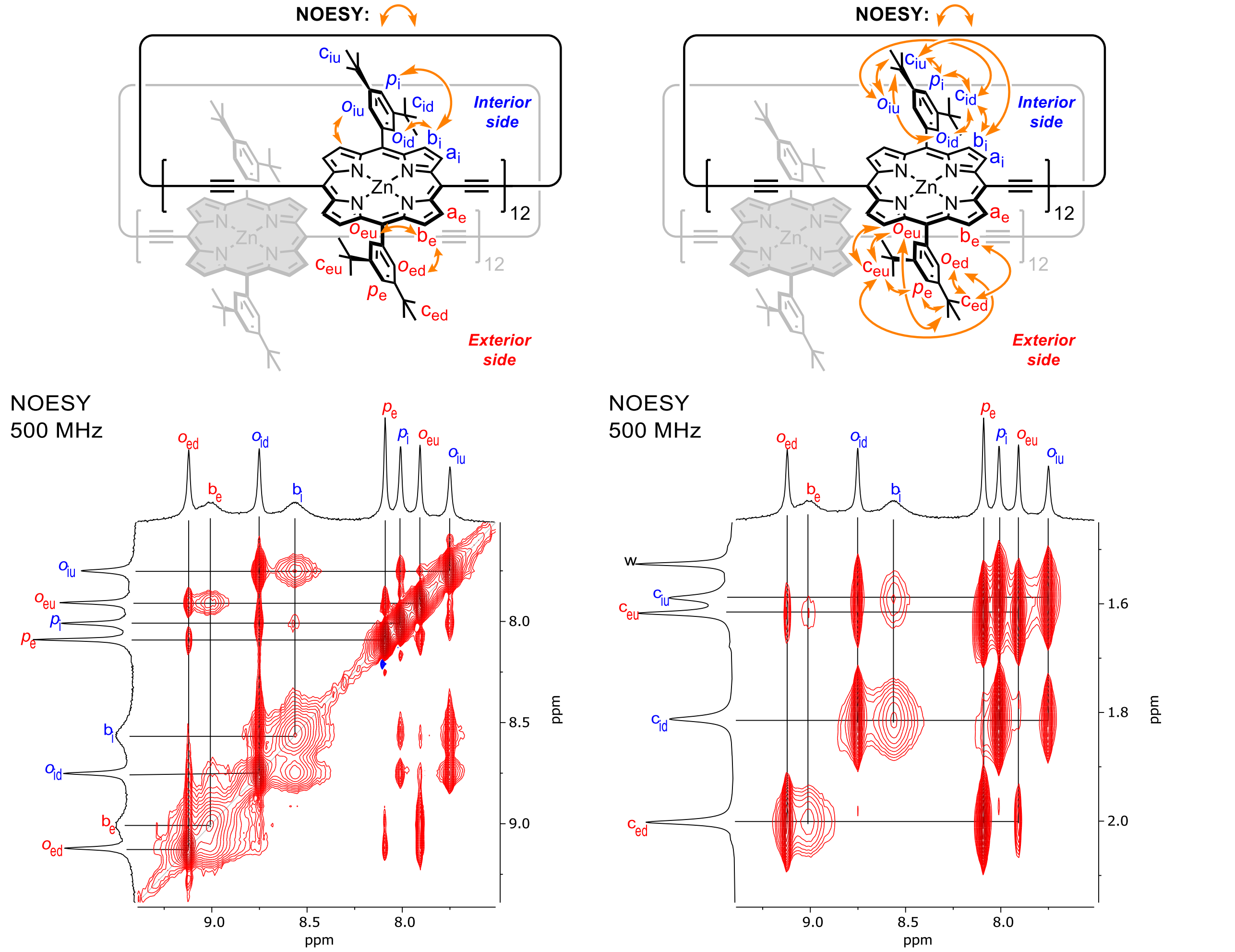

Figure S18. Selected regions of the NOESY spectrum of **(*c*-P12$_{t\text{-Bu}}$)$_2$** (500 MHz, $CDCl_3$, 298 K, mixing time = 200 ms) showing that beta porphyrin protons (b_{e} and b_{i}, left) and *t*-Bu protons (c_{ed}, c_{id}, c_{eu}, and c_{iu}, right) only correlates to aryl sidechain protons from one of the two spin systems (interior or exterior).

Table S1. Summary of relative ^{1}H-^{1}H NOE (above diagonal) and COSY (below diagonal) correlations in **(*c*-P12$_{\textit{t}\text{-Bu}}$)$_2$** (CDCl$_3$, 298 K).

	o_{ed}	b_e	o_{id}	b_i	p_e	p_i	o_{eu}	o_{iu}	c_{ed}	c_{id}	c_{eu}	c_{iu}
c_{iu}			m	m		m		s		s[#]		-
c_{eu}	w	w			m		s		m		-	X
c_{id}			s	s		s		m		-		
c_{ed}	s	s			s		m		-			
o_{iu}			s[#]	s		m		-				
o_{eu}	m	m			m		-					
p_i			m	w		-		X				
p_e	m	vw			-		X					
b_i			s	-								
o_{id}			-			X		X				
b_e	s	-										
o_{ed}	-				X		X					

[#]Relative NOE appears unexpectedly high due to exchange contribution (see Section 8). Relative strengths of NOE correlations marked as s = strong, m = medium, w = weak, vw = very weak.

Distinguishing environments oriented close to and far from the aggregate interface and between interior/exterior environments.

Up/down and interior/exterior assignments are supported by expected relative cross-peak intensities from 2D-NOESY. The up/down labelling, was arbitrarily chosen to describe *t*-Bu and *ortho* protons oriented away from (up) and towards (down) the aggregate interface. In particular, the correlations between the four different *t*-Bu groups (c_{ed}, c_{id}, c_{eu}, and c_{iu}) and the beta protons (b_i and b_e) served particularly useful. Firstly, in terms of the up/down assignment, *t*-Bu protons pointing down towards the interface c_{ed} and c_{id} can be picked out, because they are expected to have stronger NOE correlations to beta protons b_e and b_i than the *t*-Bu protons pointing up (c_{eu} and c_{iu}). This is because the *t*-Bu protons pointing down (c_{ed} and c_{id}) are considerably closer to the beta protons of the nearest porphyrin unit in the opposing ring of the aggregate (Section 7).

Secondly, considering the interior/exterior assignment, stronger correlations are expected on the interior side than on the exterior side. Based on model distances, the expected ranking of *t*-Bu ↔ beta proton NOE correlations, starting from most to least intense is: c_{id} ↔ b_i (d_{eff} averaged = 3.0 Å; relative NOE = 1.21) > c_{ed} ↔ b_e (d_{eff} averaged = 4.1 Å; relative NOE = 0.81) > c_{iu} ↔ b_i (d_{eff} averaged = 4.7 Å; relative NOE = 0.57) ≈ c_{eu} ↔ b_e (d_{eff} averaged = 4.7 Å; relative NOE = 0.40). This is also what is observed when measuring the correlations in 2D-NOESY (Section 7). Finally, the two ortho resonances within the interior and exterior groups can be linked to their closest *t*-Bu group (up or down) based on NOE intensities, which enables the interior/exterior and up/down assignment of ortho resonances. A more comprehensive analysis, including all measurable NOE correlations and model distances, were conducted and turned out to match well with the proposed assignment (Section 7).

Section 7. ^{1}H–^{1}H NOE Analysis of the (*c*-P12$_{t\text{-Bu}}$)$_2$ Aggregate

To support the assignment of up/down and interior/exterior protons, we compared the observed NOE intensities with calculated distances from a dimeric model of ***c*-P12$_{t\text{-Bu}}$**. Using six ^{1}H–^{1}H correlations as scaling factors (distance references), we calculated 22 experimental distances and compared these to distances measured from the model. All four possibilities for the assignment of the interior/exterior and up/down proton environments were evaluated, from which we conclude a clear preference based on the quality of fit (Q and R^2 factors) in favor of the proposed ^{1}H NMR assignment.

For comparison with NOESY distances, representative model distances were calculated by taking both intra- and inter-ring NOE contributions into consideration. Because the rings are close in space, it is essential for several correlations to include the NOE contribution between rings. Considering the two proton regions (interior and exterior), the inter-ring NOE contribution is expected to be larger. Intra- and inter-ring distances were included to calculate effective distances, d_{eff}(calc), contributing to a combined NOE cross peak, according to the following relationship:

$$d_{eff}(calc) = \left(\frac{r_1^n \cdot r_2^n}{r_1^n + r_2^n}\right)^{\frac{1}{n}} \qquad \text{(Eqn. S1)}$$

where r_1 and r_2 designates the intra- and inter-ring proton separations, and the factor n depends on the type of protons involved. In general, the intensity of NOESY signals scales with r^{-6} (n = 6), but it has been showed by Tropp, Koning, and others that NOESY correlations involving methyl groups scale better as r^{-3} (n = 3).[7,8] In addition, here, we have assumed that NOESY correlations involving *tert*-butyl groups scales similarly to methyl groups, i.e., with r^{-3} (n = 3). Because the process by which the two rings can rotate back-and-forth within each other is intermediate-fast at room temperature (see Figure S38 and Section 11), the beta protons are averaged due to exchange and consequently $b_{i1} = b_{i2}$ and $b_{e1} = b_{e2}$. To take account of this, d_{eff}(calc) values involving beta protons have been averaged into a d_{eff}(calc) averaged value (Table S2).

In order to calculate experimental distances from NOESY using a reference distance, we used the following relationship

$$d_{exp} = d_{ref} \cdot \left(\frac{NOE_{ref}}{NOE_{exp}}\right)^{\frac{1}{n}} \qquad \text{(Eqn. S2)}$$

where the subscripts *ref* and *exp* refer to the reference and experimental distance and relative NOE intensity. In choosing proton correlations to serve as references, we chose exterior proton correlations expected to involve only a small inter-ring contribution (see Table S2).

The quality factor (Q factor) provides a percentage of the average disagreement between model and experimental distances. The Q factor was calculated according to the following relationship:

$$Q = \sqrt{\frac{\sum_{i=1}^{N}(d_{calc} - d_{exp})^2}{\sum_{i=1}^{N}(d_{exp})^2}} \qquad \text{(Eqn. S3)}$$

where N refers to the total number of counted NOE correlations.

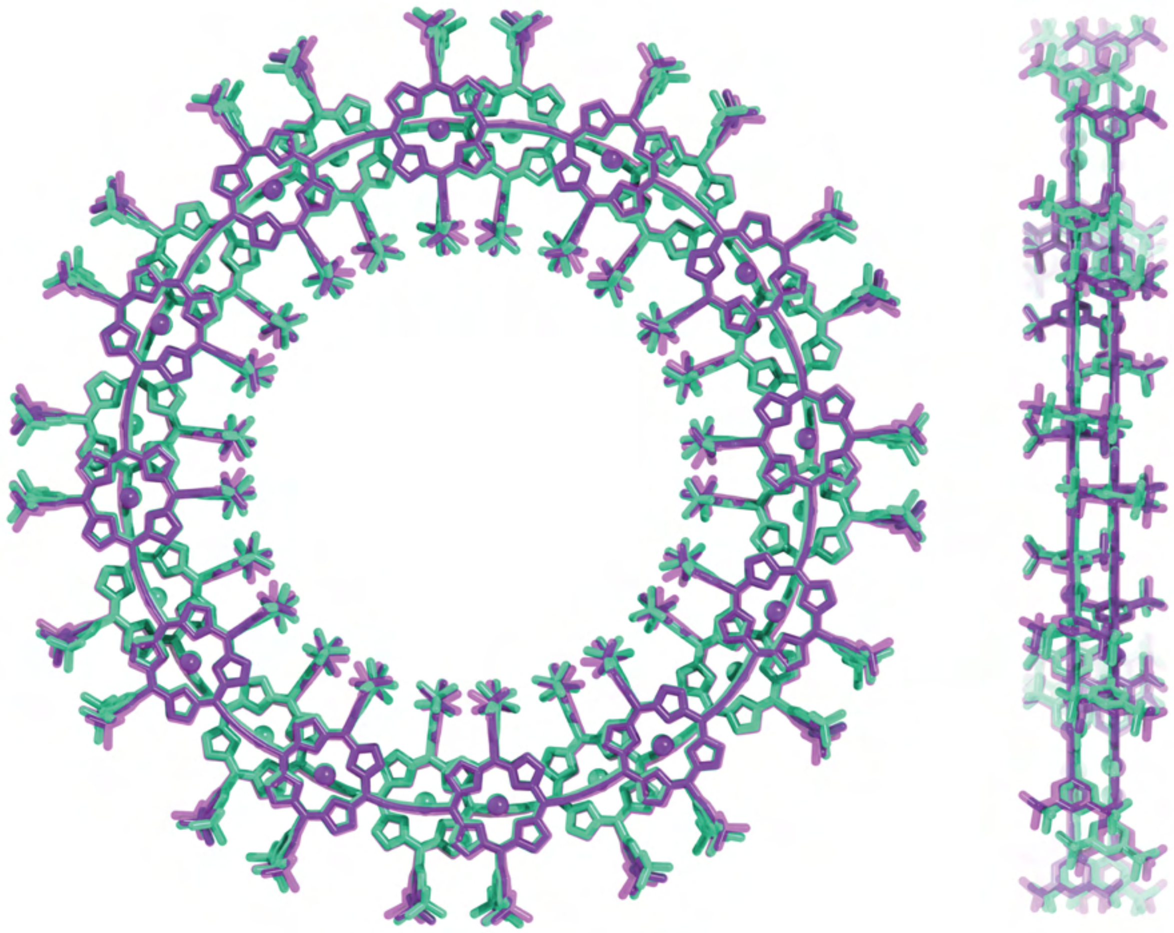

Figure S19. Comparison between ***c*-P12$_{t\text{-Bu}}$** dimer models. Superposition of the xTB minimized model (in green) and an idealized model (in purple) built using parameters derived from the xTB minimized geometry, i.e., the average interior (88°) and exterior (82 °) sidechain dihedrals and extent of porphyrin ring rotation (2.76 °). Root-mean-square-deviation between the two models is 0.71 Å. The average distance between rings in the two models are 3.0 Å (xTB minimized) and 3.4 Å (idealized), measured as the distance between mean planes defined by the C_{meso} atoms of each ring. The idealized model was employed in the analysis of both NOESY and residual dipolar coupling experiments.

Table S2. Summary of ***c*-P12**$_{t\text{-}Bu}$ dimer through-space ^{1}H-^{1}H correlations and their calculated and experimental distances (700 MHz, $CDCl_3$, 25 °C).

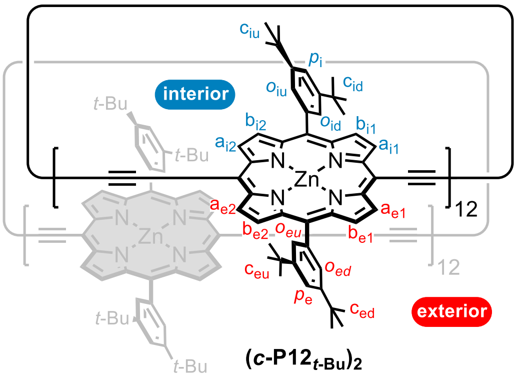

(*c*-P12$_{t\text{-}Bu}$)$_2$

^{1}H ↔ ^{1}H correlation	$d_{intramolecular}$ (calc) / Å	$d_{intermolecular}$ (calc) / Å	d_{eff} (calc)[a] / Å	d_{eff} (calc) averaged[b] / Å	Relative NOE[c]	d_{eff} (exp)[d] / Å
$b_{e1} \leftrightarrow c_{eu}$ (ref)	4.9	8.9	4.65	4.7	0.40	(4.7)
$b_{e2} \leftrightarrow c_{eu}$ (ref)	5.2	8.1	4.81			
$b_{e1} \leftrightarrow c_{ed}$	5.3	7.3	4.76	4.1	0.81	3.7
$b_{e2} \leftrightarrow c_{ed}$	4.8	4.0	3.44			
$b_{i1} \leftrightarrow c_{iu}$	5.1	7.8	4.70	4.7	0.57	4.2
$b_{i2} \leftrightarrow c_{iu}$	5.1	7.4	4.64			
$b_{i1} \leftrightarrow c_{id}$	4.4	6.2	4.31	3.5	1.21	3.2
$b_{i2} \leftrightarrow c_{id}$	4.7	2.8	2.63			
$b_{e1} \leftrightarrow o_{eu}$ (ref)	3.2	8.5	3.20	3.4	0.22	(3.4)
$b_{e2} \leftrightarrow o_{eu}$ (ref)	3.6	7.0	3.59			
$o_{ed} \leftrightarrow b_{e1}$	3.7	7.2	3.69	3.3	0.35	3.1
$o_{ed} \leftrightarrow b_{e2}$	3.1	3.9	2.99			
$b_{e1} \leftrightarrow p_e$	5.0	8.9	4.97	4.8	0.04	4.5
$b_{e2} \leftrightarrow p_e$	4.8	6.7	4.70			
$o_{id} \leftrightarrow b_{i1}$	3.3	4.1	3.17	2.7	0.49	3.0
$o_{id} \leftrightarrow b_{i2}$	3.5	2.3	2.27			
$b_{i1} \leftrightarrow o_{iu}$	3.4	6.9	3.39	3.4	0.30	3.2
$b_{i2} \leftrightarrow o_{iu}$	3.4	5.8	3.38			
$b_{i1} \leftrightarrow p_i$	4.8	6.6	4.69	4.7	0.07	4.1
$b_{i2} \leftrightarrow p_i$	5.0	5.7	4.70			
$p_e \leftrightarrow o_{eu}$ (ref)	4.3	9.8	4.29	4.3	0.05	(4.3)
$p_i \leftrightarrow o_{iu}$	4.3	7.8	4.28	4.3	0.06	4.2
$o_{ed} \leftrightarrow o_{eu}$	4.3	7.3	4.27	4.3	0.10	3.8
$o_{ed} \leftrightarrow p_e$	4.3	7.7	4.28	4.3	0.05	4.3
$o_{id} \leftrightarrow o_{iu}$ (Exc)	4.3	5.6	4.17	4.2	0.30	3.2
$o_{id} \leftrightarrow p_i$	4.3	5.6	4.17	4.2	0.07	4.1
$o_{eu} \leftrightarrow c_{eu}$ (ref)	2.3	12.2	2.29	2.3	1.06	(2.3)
$o_{iu} \leftrightarrow c_{iu}$	2.3	9.6	2.29	2.3	0.78	2.5
$o_{ed} \leftrightarrow c_{ed}$	2.3	6.3	2.26	2.3	1.03	2.3
$o_{id} \leftrightarrow c_{id}$	3.1	4.6	2.84	2.8	0.89	2.4
$p_e \leftrightarrow c_{eu}$	3.1	8.8	3.06	3.1	1.14	2.2
$p_e \leftrightarrow c_{ed}$	3.1	5.5	2.93	2.9	1.12	2.3
$p_i \leftrightarrow c_{iu}$	3.1	6.6	3.00	3.0	1.10	2.3
$p_i \leftrightarrow c_{id}$	2.3	2.3	1.83	1.8	1.21	2.2
$o_{eu} \leftrightarrow c_{ed}$ (ref)	6.3	6.8	5.18	5.2	0.22	(5.2)
$o_{iu} \leftrightarrow c_{id}$	6.3	4.8	4.25	4.2	0.51	3.9
$o_{ed} \leftrightarrow c_{eu}$	6.3	7.9	5.49	5.5	0.21	5.3
$o_{id} \leftrightarrow c_{iu}$	6.3	4.6	4.12	4.1	0.46	4.1
$c_{ed} \leftrightarrow c_{eu}$ (ref)	5.3	5.8	4.39	4.4	2.03	(4.4)
$c_{id} \leftrightarrow c_{iu}$ (Exc)	5.1	3.3	3.05	3.1	7.99	2.8

(ref) are reference correlations used for scaling other interactions of similar type. (Exc) means the observed correlation is dominated by exchange rather than an NOE interaction (based on ROESY, see Section 8), and is thus excluded from the NOE analysis. Distances involving *t*-Bu group hydrogens (i.e., c_{iu}, c_{id}, c_{eu}, and c_{ed}) were measured from the methyl group center of mass, resulting in the shortest possible distance for any particular interaction. [a]An effective calculated distance taking into account both intra- and intermolecular distances was calculated according to Eqn. S1 and using distances from an idealized ***c*-P12**$_{t\text{-}Bu}$ dimer model shown in Figure S19. [b]Since porphyrin rotation leads to intermediate-fast exchange of beta protons (i.e., $b_{e1} \leftrightarrow b_{e2}$ and $b_{i1} \leftrightarrow b_{i2}$) at room temperature, the contribution from each proton of an exchange-pair (for example, $b_{e1} \leftrightarrow b_{e2}$) were averaged. [c]Relative NOEs correspond to the integrated cross peak volumes from 2D NOESY. [d]Experimental NOE distance, as calculated according to Eqn. S2 using one of the reference distances and the particular integrated NOE cross peak.

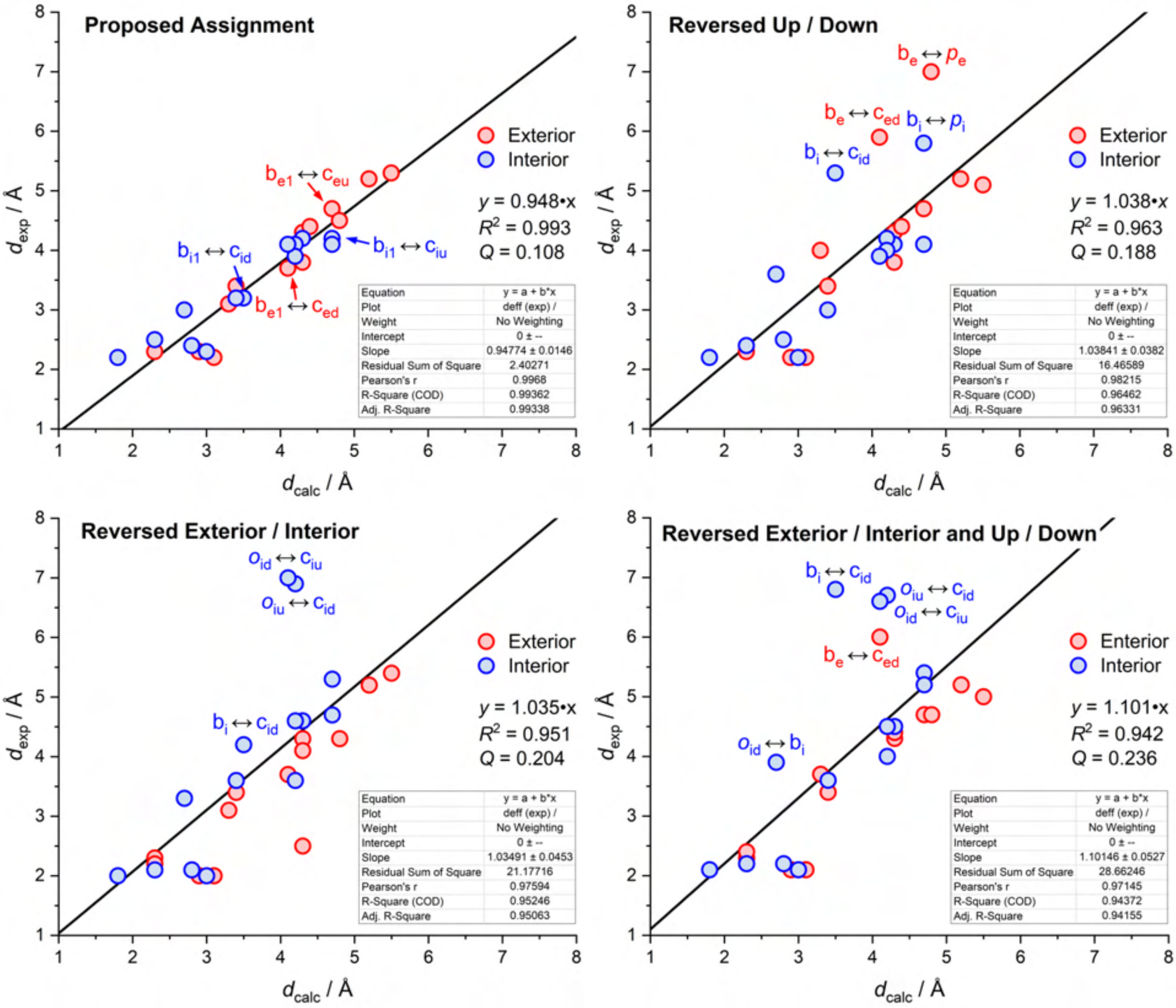

Figure S20. Linear fits of experimental and calculated distances according to the proposed assignment (top, left), a reversed up/down assignment (top, right), a reversed exterior/interior assignment (bottom, left), and a reversed exterior/interior and up/down (left) assignment (bottom, right). Based on the scoring parameters R^2 and Q, the best match is obtained with the proposed assignment.

Section 8. ^{1}H-^{1}H ROESY Characterization

Through 2D-ROESY experiments, we could identify two kinds of exchange processes occurring in the ring aggregates: rotation of the porphyrin units and rotation of the interior and exterior aryl group connected at the *meso* position. ROESY spectra measured for the dimeric aggregates of ***c*-P12**$_{t\text{-Bu}}$, ***c*-P8**$_{t\text{-Bu}}$, ***c*-P12**$_{\text{OOct}}$, and ***c*-P8**$_{\text{OOct}}$ are shown below. ROESY cross-peaks corresponding to exchange and NOE contributions were distinguished based on their difference in phase: positive = exchange (red); negative = NOE (blue).

<u>8- and 12-porphyrin nanoring dimers with *tert*-butyl solubilizing groups</u>

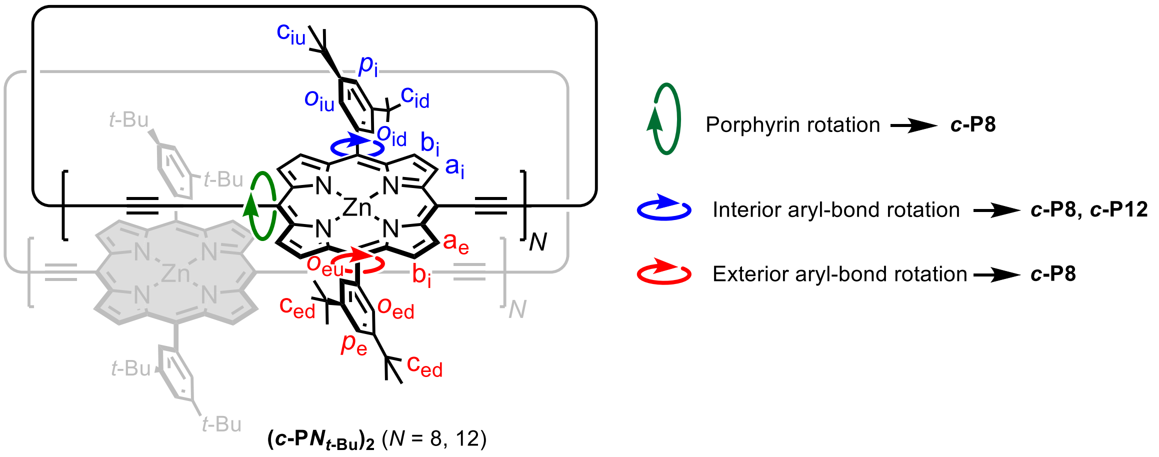

Figure S21. Labelled structure of **(*c*-P*N*$_{t\text{-Bu}}$)$_2$** (*N* = 8, 12) including the physical exchange processes, porphyrin and interior/exterior aryl-bond rotation, revealed by 2D-ROESY.

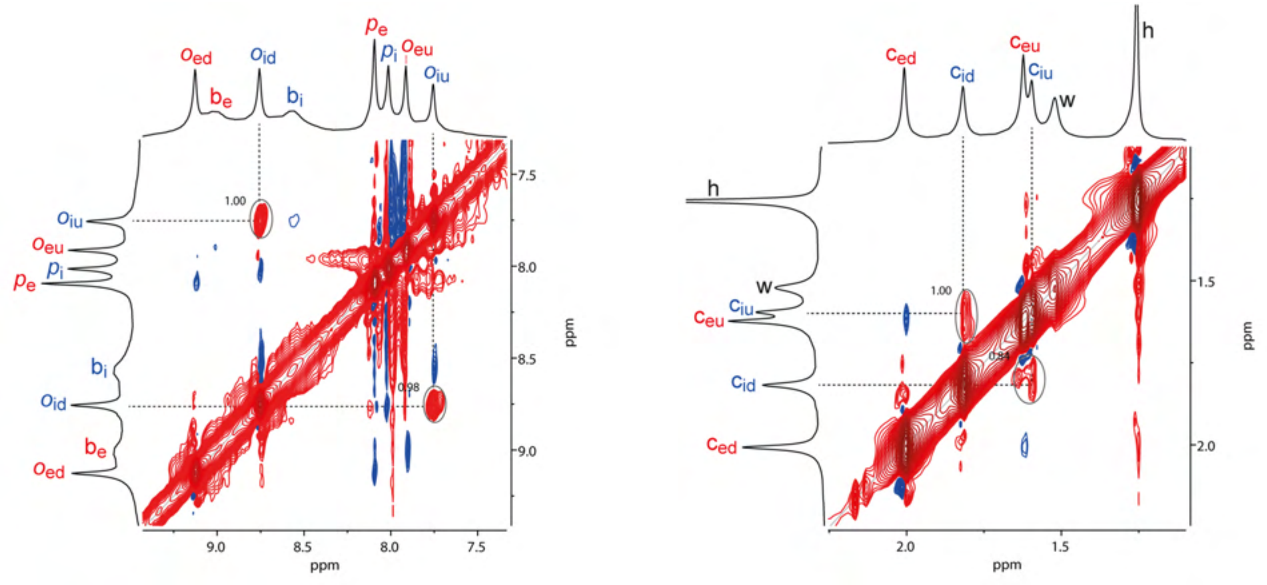

Figure S22. Selected regions from ROESY of the ***c*-P12*t*-Bu** dimer (500 MHz, $CDCl_3$, 298 K, mixing time = 50 ms). Exchange correlations can be seen between aryl *ortho* resonances o_{id}/o_{iu} (left) and between *t*-Bu resonances c_{id}/c_{iu} (right). These correlations imply that one aryl group is rotating faster than the other since exchange is not detected between the red resonances o_{ed}/o_{eu} and c_{ed}/c_{eu}.

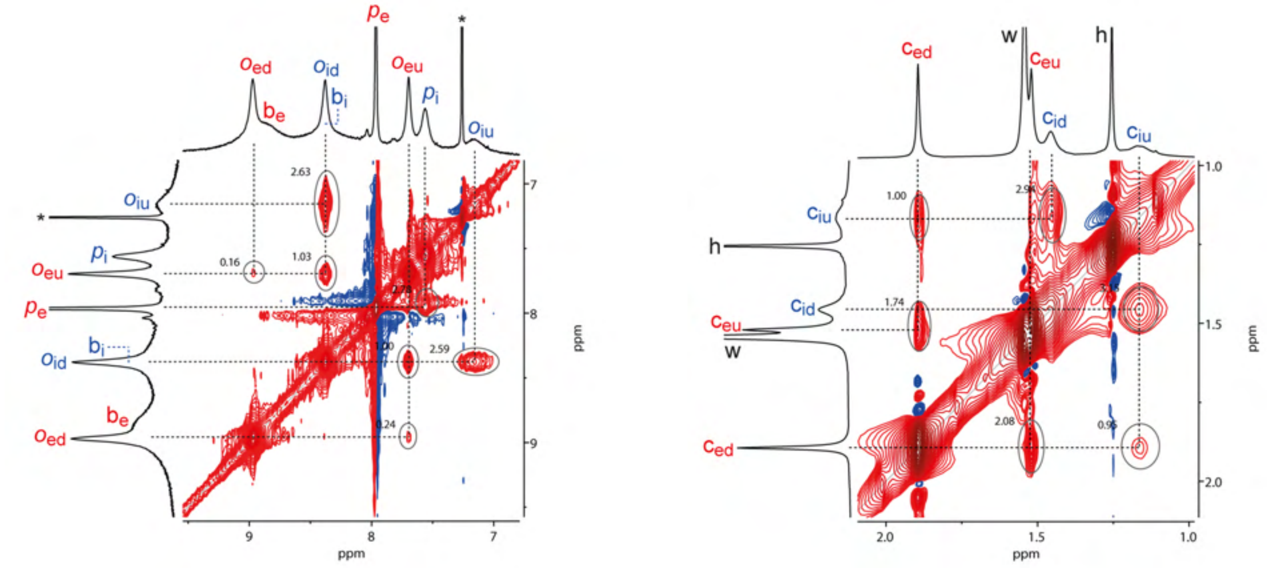

Figure S23. Selected regions from ROESY of the ***c*****-P8**$_{t\text{-Bu}}$ dimer (500 MHz, $CDCl_3$, 298 K, mixing time = 50 ms). Abbreviations: * = residual $CHCl_3$; w = water; h = H-grease. Several exchange correlations can be seen involving aryl *ortho* and *para* resonances (left) as well as between *t*-Bu resonances (right). These correlations point to two different processes being active: 1) *meso*-aryl bond rotation, as seen for the dimer of ***c*****-P12**$_{t\text{-Bu}}$, is implied by both $o_{ed} \leftrightarrow o_{eu}$, $o_{id} \leftrightarrow o_{iu}$, $c_{ed} \leftrightarrow c_{eu}$, and $c_{id} \leftrightarrow c_{iu}$ exchange occurring within the exterior and interior aryl groups; 2) rotation of the porphyrin unit, leading to interconversion of the exterior and interior environments, is implied by exchange between the resonances $p_e \leftrightarrow p_i$, $o_{eu} \leftrightarrow o_{id}$, and $c_{ed} \leftrightarrow c_{iu}$. At the same time, exchange correlations across the interior and exterior environments between resonances of the same type, such as $o_{ed} \leftrightarrow o_{id}$, $o_{eu} \leftrightarrow o_{iu}$, $c_{ed} \leftrightarrow c_{id}$, and $c_{eu} \leftrightarrow c_{iu}$ are not observed, suggesting this exchange process does not occur through complete dissociation of the two rings. Because, in that case, the two ring faces would become scrambled and each kind of *ortho* or *t*-Bu resonance would have an equal probability of ending up pointing either inwards or outwards as a result of exchange. This pattern of exchange is fitting of a dynamic behavior where the rings keep in contact through the rotation process.

8- and 12-porphyrin nanoring dimers with octyloxy solubilizing groups

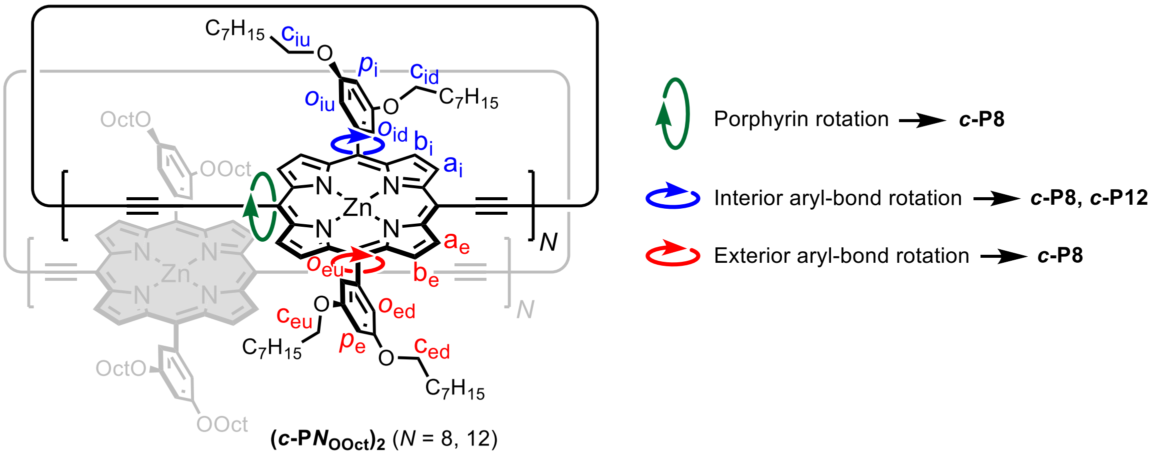

(c-PN$_{OOct}$)$_2$ (*N* = 8, 12)

Figure S24. Labelled structure of **(*c*-P*N*$_{OOct}$)$_2$** (N = 8, 12) including the physical exchange processes, porphyrin and interior/exterior aryl-bond rotation, revealed by 2D-ROESY.

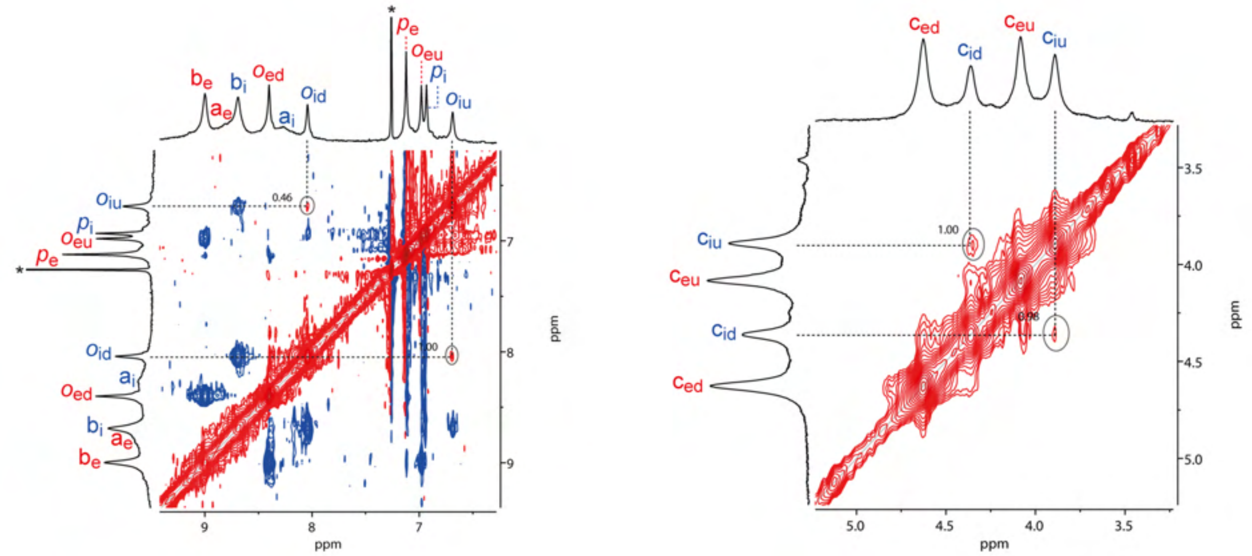

Figure S25. Selected ROESY regions of the ***c*-P12$_{OOct}$** dimer (500 MHz, $CDCl_3$, 298 K, mixing time = 100 ms). Weak exchange correlations between o_{id}/o_{iu} (left) and the OCH_2 resonances c_{id}/c_{iu} (right) can be seen. These correlations implies that one aryl group is rotating faster than the other since exchange is not observed between o_{ed}/o_{eu} and c_{ed}/c_{eu} belonging the exterior aryl group.

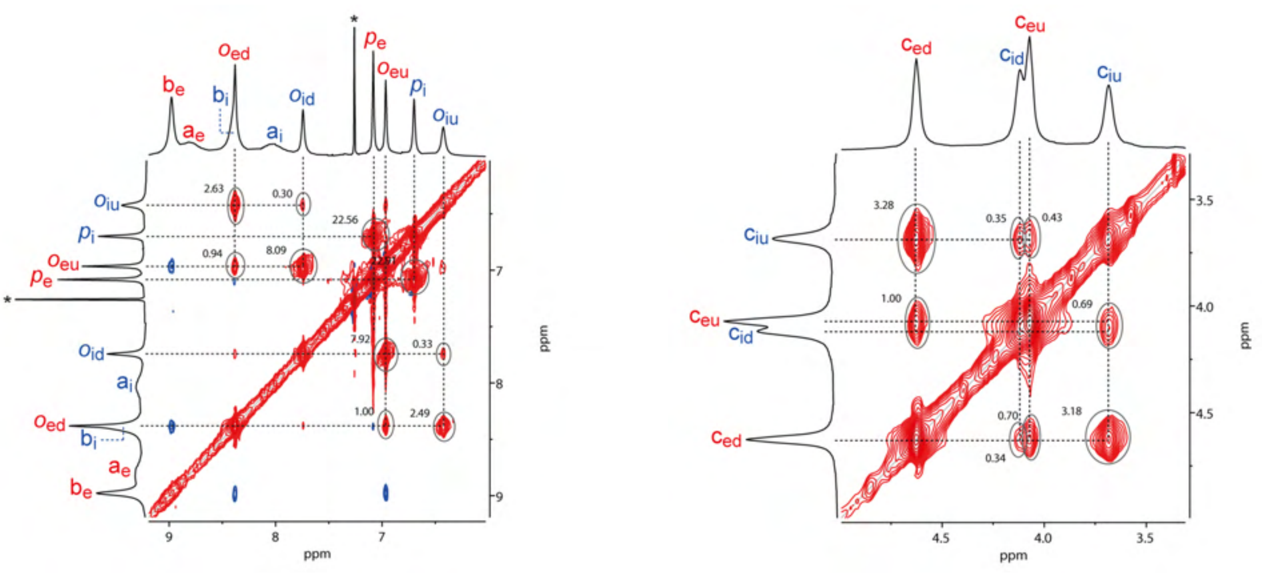

Figure S26. Selected regions from ROESY of the ***c*-P8$_{\mathbf{OOct}}$** dimer (500 MHz, $CDCl_3$, 298 K, mixing time = 100 ms). Several exchange correlations can be seen involving aryl *ortho* and *para* resonances (left) and the OCH_2 resonances (right). The exchange pattern is similar to that seen for the ***c*-P8$_{\boldsymbol{t}\text{-}\mathbf{Bu}}$** dimer. The exchange correlations point to two exchange processes: 1) *meso*-aryl bond rotation, being implied by the following exchange pathways: $o_{ed} \leftrightarrow o_{eu}$, $o_{id} \leftrightarrow o_{iu}$, $c_{ed} \leftrightarrow c_{eu}$, and $c_{id} \leftrightarrow c_{iu}$; 2) rotation of the porphyrin unit, leading to interconversion of the exterior and interior environments, which is implied by exchange between the resonances $p_e \leftrightarrow p_i$, $o_{ed} \leftrightarrow o_{iu}$, $o_{id} \leftrightarrow o_{eu}$, $c_{ed} \leftrightarrow c_{iu}$, $c_{id} \leftrightarrow c_{eu}$. As with the ***c*-P8$_{\boldsymbol{t}\text{-}\mathbf{Bu}}$** dimer, it is noted that exchange occurs primarily between opposite types of ortho and OCH_2 resonances (interior becomes exterior or vice versa), suggesting the two rings do not entirely dissociate through this process.

Section 9. Selective Magnetization Transfer Experiments to Measure Rates of Exchange

Exchange processes revealed by 2D ROESY were studied in more detail using 1D selective NOESY experiments recorded at different mixing times (between 2.4 and 200 ms) in order to extract the rates of exchange. For this purpose, the selnogp pulse sequence was used and in case of each selective excitation, the width of the pulse was optimized by variation of its length (20 to 50 ms).[9–11]

We looked at the exchange in ***c*-P8**$_{\mathbf{OOct}}$, ***c*-P12**$_{\mathbf{OOct}}$, and ***c*-P12**$_{\boldsymbol{t}\mathbf{\text{-}Bu}}$. The exchanging ^{1}H resonances of ***c*-P8**$_{\boldsymbol{t}\mathbf{\text{-}Bu}}$ were too broad to quantitatively measure exchange.

In ***c*-P8**$_{\mathbf{OOct}}$, it was possible to measure the rate of all three exchange processes (porphyrin rotation, interior aryl-bond rotation, and exterior aryl-bond rotation). For ***c*-P12**$_{\mathbf{OOct}}$ and ***c*-P12**$_{\boldsymbol{t}\mathbf{\text{-}Bu}}$, the rate of interior aryl-bond rotation was measured. We were unable to observe exchange corresponding to exterior aryl-bond rotation, at least at room temperature.

The measured exchange intensities were normalized using the integral sum of all resonances involved in the exchange process and plotted against the mixing time. Next, the evolution of exchange intensity was fitted to an appropriate kinetic model (see below) using the Berkeley Madonna software package to obtain the observed rate constants of exchange.[12,2] The observed rate constants were used to calculate the rotational barriers using the Eyring equation. However, first the observed rate constants were doubled to reflect the rate of the actual physical process of rotation. As described by Green and Wong, the observed rate of exchange is not identical to the rate of the physical process which give rise to the exchanging resonances.[13,14] This is because only magnetization transferred in one direction is measured, i.e., from the resonance being selectively excited to its exchange partner. For a two-state exchange system of equal populations, the probability of the exchange process leading to observable transferred magnetization is one half, because the transition state of the exchange process can move in both directions with equal probabilities. Therefore, the physical rate constant (k_{chem}) is twice the observed exchange rate constant (k_{obs}):

$$k_{chem} = 2 \cdot k_{obs} \qquad \text{(Eqn. S4)}$$

The Eyring equation was used to calculate rotational barriers ($\Delta G^{\ddagger}$) at 298 K from the physical rate constants:

$$\Delta G^{\ddagger} = R \cdot T \cdot ln\left(\frac{k_B \cdot T}{h \cdot k_{chem}}\right) \qquad \text{(Eqn. S5)}$$

Where $R = 8.314\ \text{J}\cdot\text{K}^{-1}\cdot\text{mol}^{-1}$, $k_{\text{B}} = 1.381\cdot10^{-23}\ \text{J}\cdot\text{K}^{-1}$, and $h = 6.626\cdot10^{-34}\ \text{J}\cdot\text{s}$ were used. All rate constants and rotational barriers are summarized in Table S3.

The dimer of ***c*-P8** with octyloxy sidechains

Exchange within the ***c*-P8$_{OOct}$** dimer was probed by selective excitation of the p_i, o_{iu}, and c_{iu} resonances. We decided to use resonances oriented away from the interface of the aggregate, because this would limit the loss of magnetization due to NOE contributions, which are expected to be more severe closer to the interface. Exchange between *para* resonances is only affected by porphyrin rotation. On the other hand, exchange between *ortho* or OCH_2 resonances is affected by both porphyrin rotation and aryl-bond rotation.

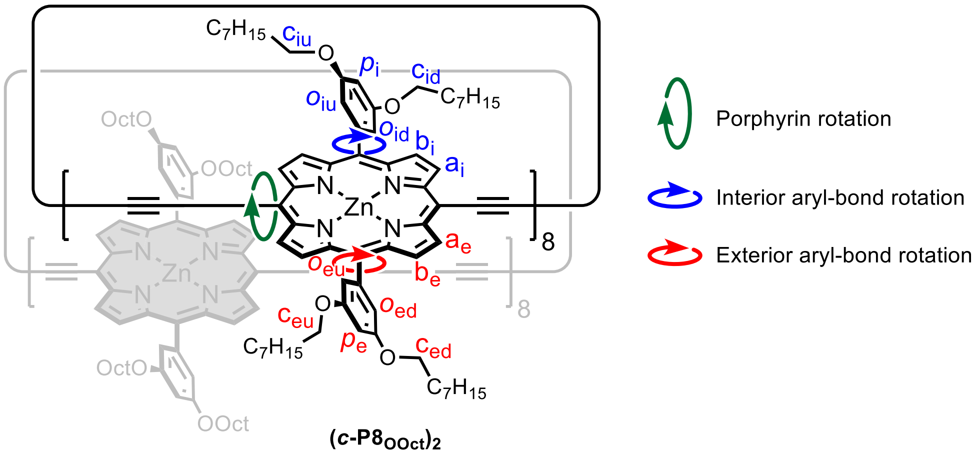

Figure S27. Exchange processes observed in the ***c*-P8$_{OOct}$** dimer.

1D EXSY spectra measured with selective excitation of p_i, o_{iu}, and c_{iu} are shown below in Figure S28. Exchange (Exc) and NOE contributions are labelled accordingly.

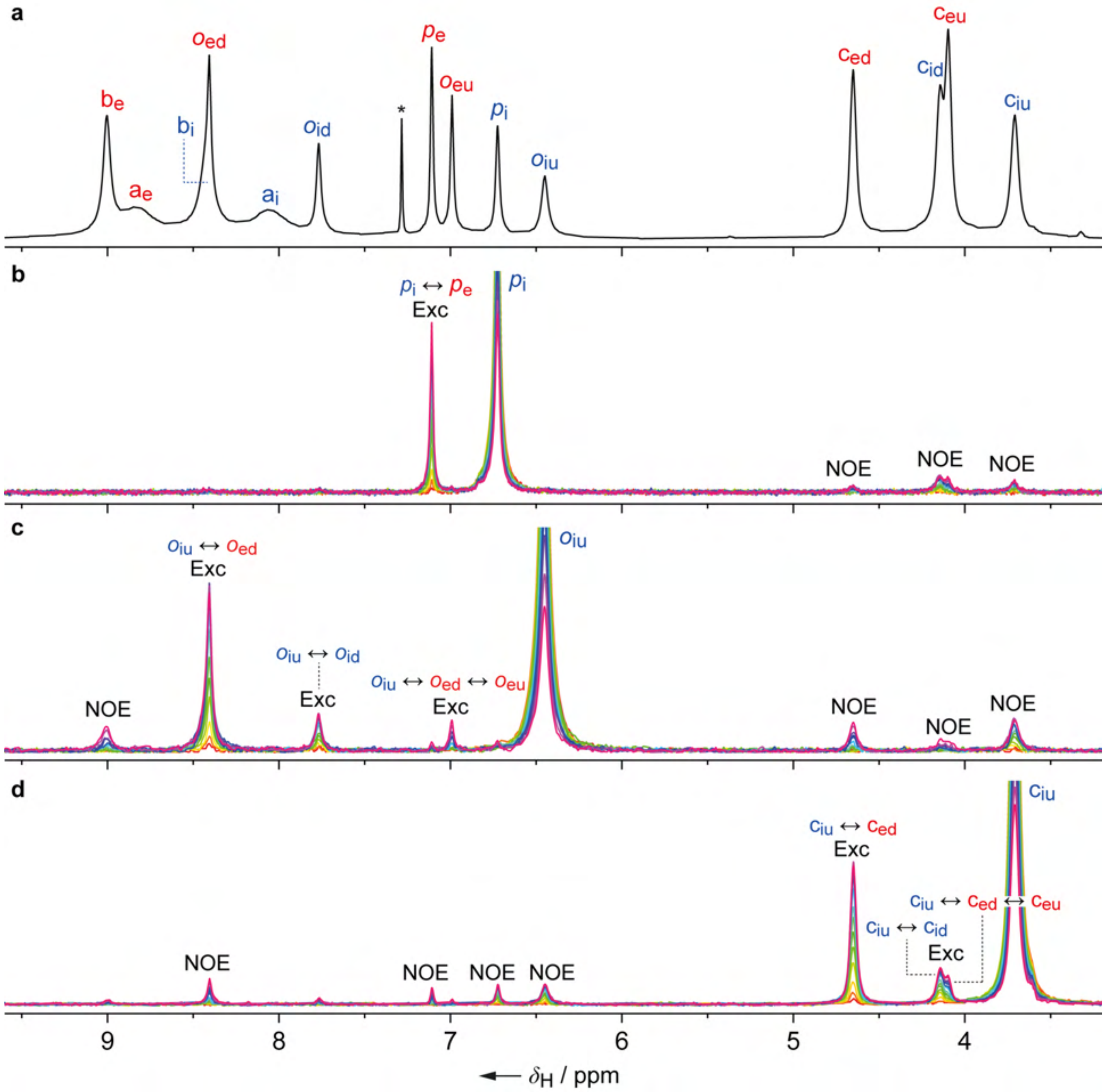

Figure S28. ^{1}H and 1D EXSY spectra of the ***c*-P8$_{OOct}$** dimer ($CDCl_3$, 298 K, 500 MHz). a) ^{1}H spectrum. b-d) 1D EXSY spectra (mixing times = 2.4, 5.0, 10, 20, 30, 40, 50, 60, 80, 100, 120, 140, 160, 180 and 200 ms) with selective excitation of the p_i *para* resonance (b), o_{iu} *ortho* resonance (c), and c_{iu} OCH_2 resonance (d).

The signal intensity of each signal at each mixing time was normalized using the integral sum of all exchanging resonances. The resulting plots of normalized signal intensities as a function of mixing time were fitted to a global kinetic model shown in Figure S29.

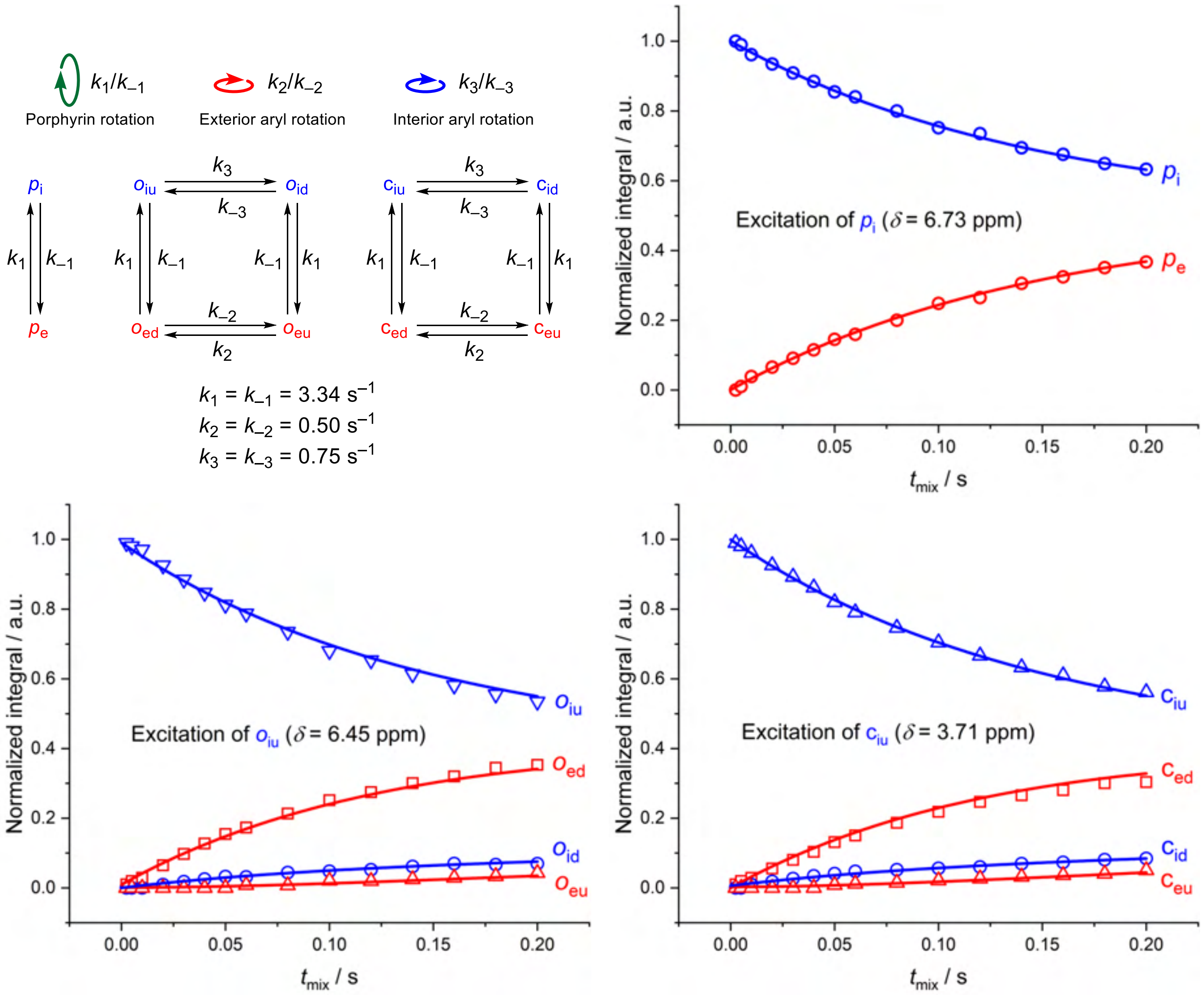

Figure S29. Kinetic model and plots of normalized exchange intensity as a function of mixing time for the ***c*-P8**$_{\mathbf{OOct}}$ dimer. Top left: kinetic model used to simultaneously fit all three series of exchange intensity evolution. The root mean square deviation of the fit is 0.070. Top right: evolution of exchange intensity as a result of excitation of p_i (porphyrin rotation). Bottom left: evolution of exchange intensity as a result of excitation of o_{iu} (porphyrin, interior, and exterior rotation). Bottom right: evolution of exchange intensity as a result of excitation of c_{iu} (porphyrin, interior, and exterior rotation).

Exchange within the dimer of ***c*-P12** with *t*-Bu sidechains

Exchange within the ***c*-P12$_{t\text{-Bu}}$** dimer was probed by selective excitation of the o_{iu} resonance. Exchange between interior *ortho* resonances $o_{iu} \leftrightarrow o_{id}$ is only affected by aryl-bond rotation, since porphyrin rotation does not occur in this system.

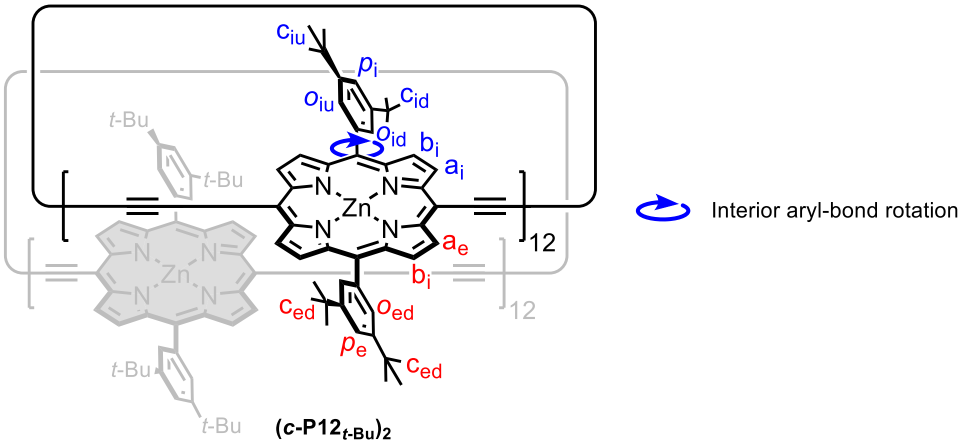

Figure S30. Exchange processes observed in the ***c*-P12$_{t\text{-Bu}}$** dimer.

Although 2D ROESY did not suggest exchange between *ortho* resonances on the exterior side, we wondered whether it would be possible to detect a slower exchange by 1D EXSY. However, first we wanted to confirm the existence of an exchange process. A long 1D ROESY experiment with a selective excitation of the exterior o_{eu} resonance showed no measurable exchange between o_{eu} and o_{ed}, but instead pointed to a weak NOE correlation between the two (Figure S31). Therefore, we concluded that only the interior aryl group is rotating at a measurable rate at 298 K.

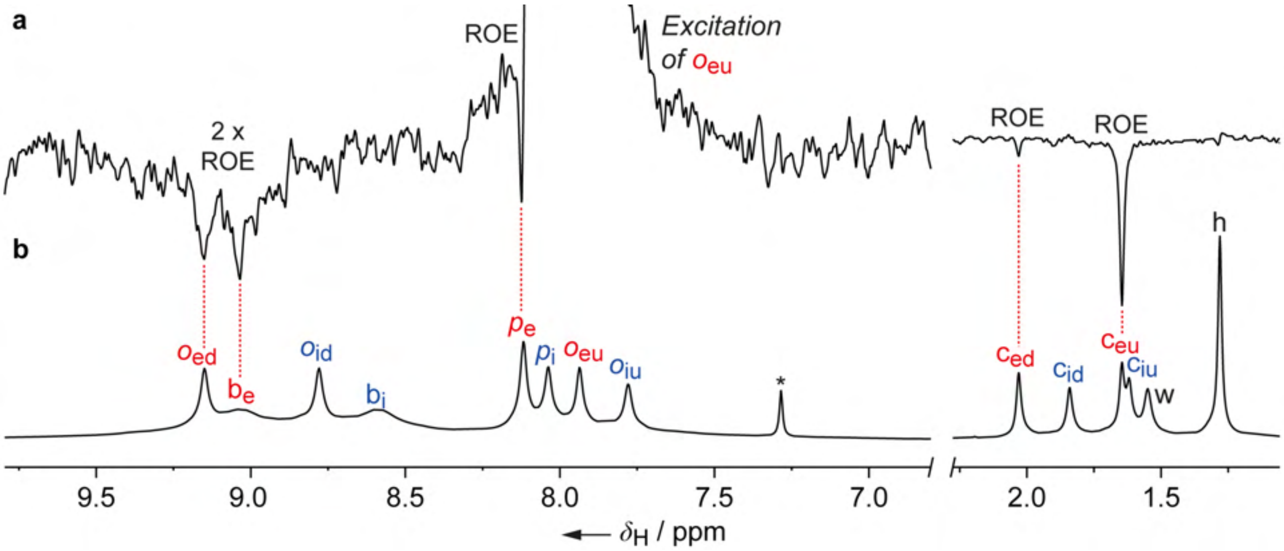

Figure S31. 1D EXSY (ROESY) and ^{1}H spectra of the ***c*-P12$_{t\text{-Bu}}$** dimer ($CDCl_3$, 298 K, 500 MHz). a) 1D EXSY with selective excitation of o_{eu}, resulting in ROEs of other exterior resonances, but no sign of exchange, as evidenced by the negative phasing of the signals compared to the peak of excitation. b) ^{1}H spectrum.

1D EXSY spectra measured with selective excitation of o_{iu} are shown in Figure S32. Exchange (Exc) and NOE contributions are labelled accordingly.

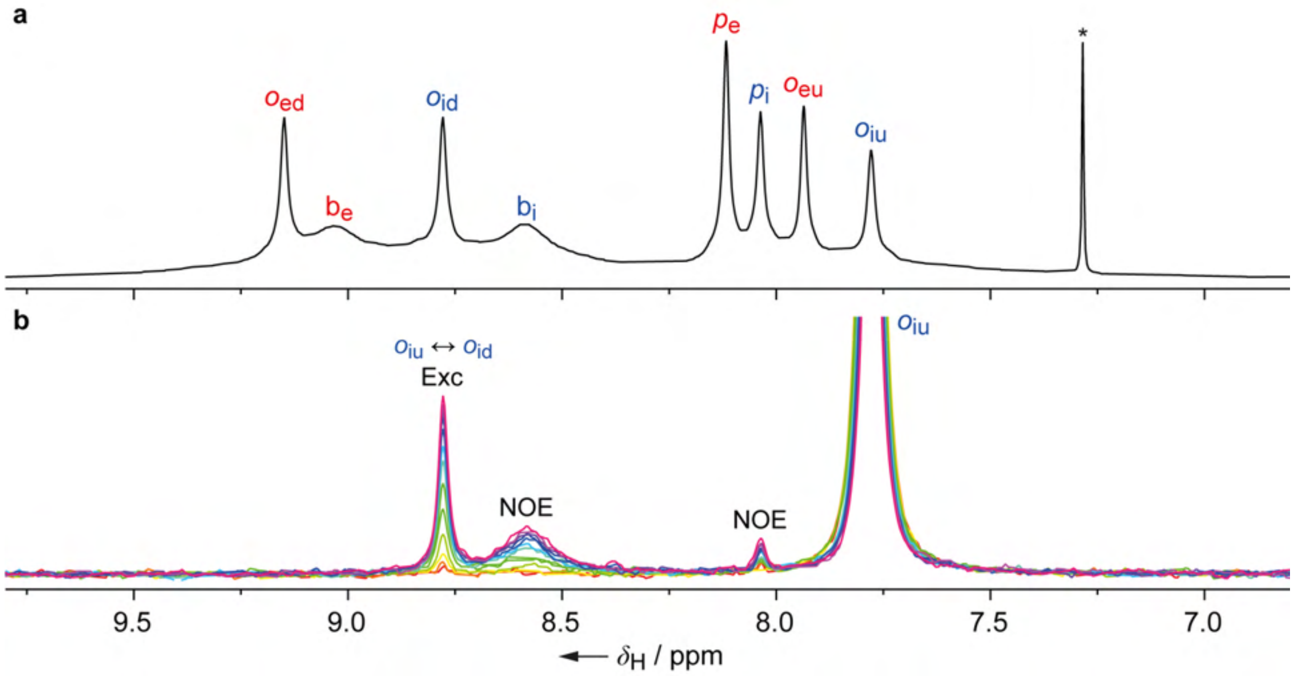

Figure S32. ^{1}H and 1D EXSY spectra of the ***c*-P12$_{t\text{-Bu}}$** dimer ($CDCl_3$, 298 K, 500 MHz). a) ^{1}H spectrum. b) 1D EXSY spectrum with selective excitation of the o_{iu} resonance.

Again, the signal intensity of each signal at each mixing time was normalized using the integral sum of all exchanging resonances. The resulting plots of normalized signal intensities as a function of mixing time were fitted to a two-state kinetic model shown in below Figure S33.

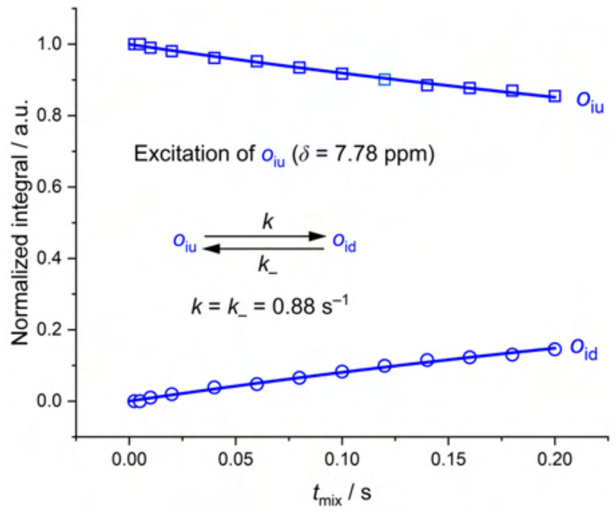

Figure S33. Kinetic model and plot of normalized exchange intensity as a function of mixing time for the ***c*-P12$_{t\text{-Bu}}$** dimer. Root mean square deviation of the fit is 0.0067. Evolution of exchange intensity as a result of excitation of o_{iu} (interior aryl rotation).

Exchange within the ***c*-P12** dimer with octyloxy sidechains

Exchange within the ***c*-P12**$_{\mathbf{OOct}}$ dimer was probed by selective excitation of the o_{iu} resonance. Exchange between exterior *ortho* resonances $o_{iu} \leftrightarrow o_{id}$ is only affected by aryl-bond rotation, since porphyrin rotation does not occur in this system.

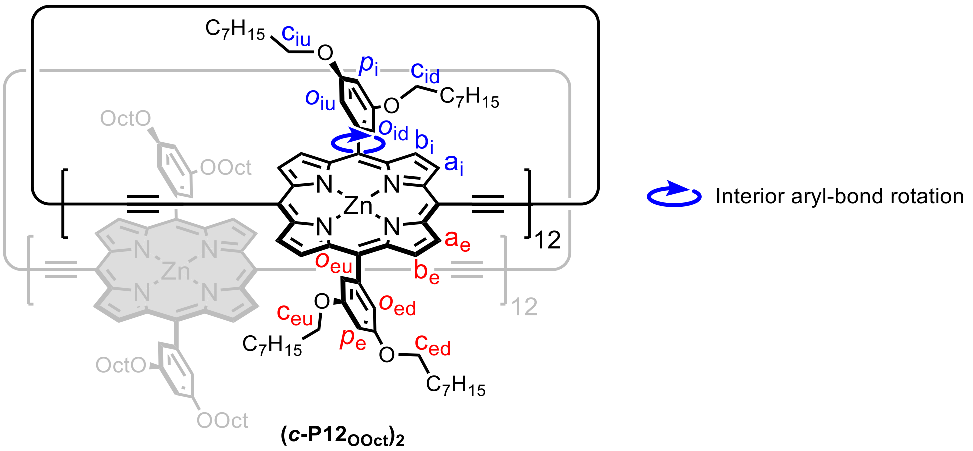

Figure S34. Exchange processes observed in the ***c*-P12**$_{\mathbf{OOct}}$ dimer.

1D EXSY spectra measured with selective excitation of o_{iu} are shown below in Figure S35. Exchange (Exc) and NOE contributions are labelled accordingly.

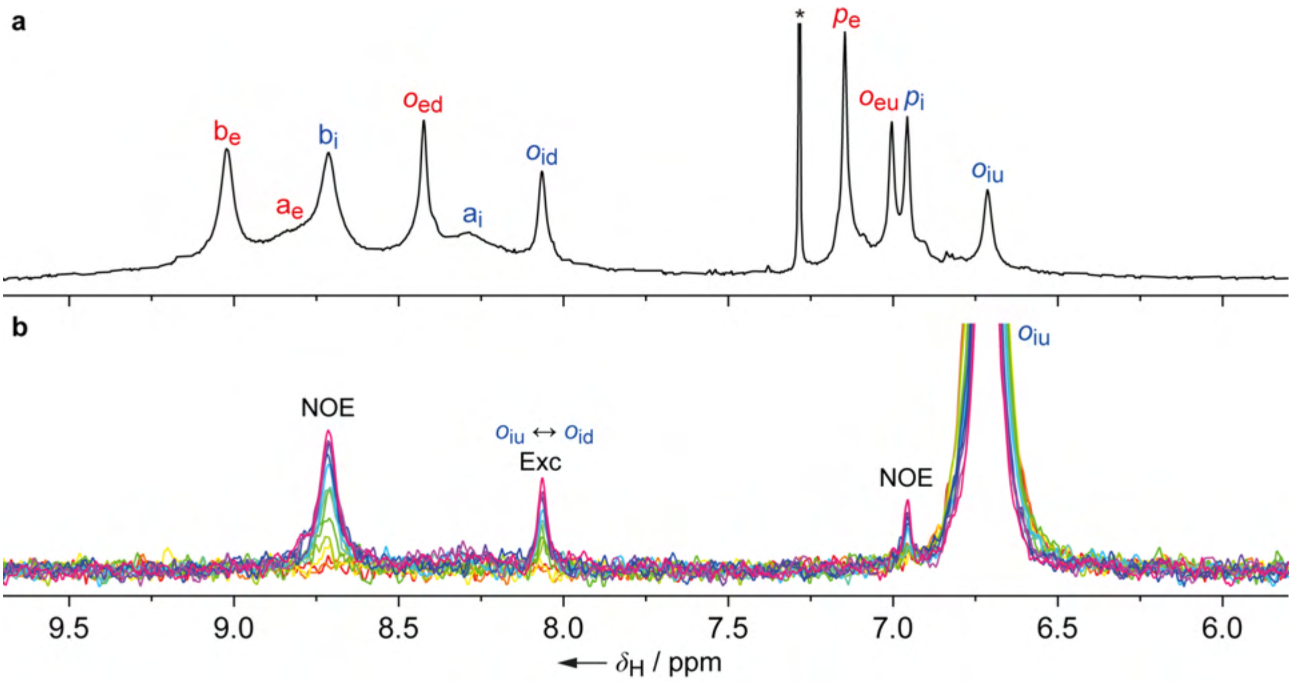

Figure S35. ^{1}H and 1D EXSY spectra of the ***c*-P12**$_{\mathbf{OOct}}$**)** dimer ($CDCl_3$, 298 K, 500 MHz). **a**) ^{1}H spectrum. **b**) 1D EXSY spectrum with selective excitation of o_{iu}.

The signal intensity of each signal at each mixing time was normalized using the integral sum of all exchanging resonances. The resulting plots of normalized signal intensities as a function of mixing time were fitted to a two-state kinetic model shown in below Figure S36.

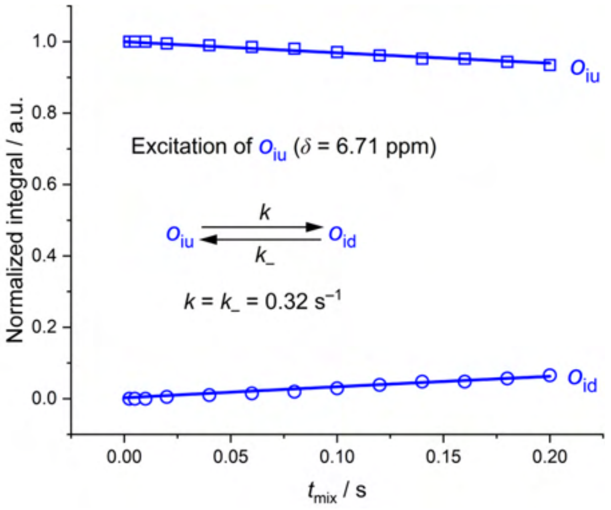

Figure S36. Kinetic model and plot of normalized exchange intensity as a function of mixing time for the ***c*-P12$_{OOct}$)** dimer. Root mean square deviation of the fit is 0.0076. Evolution of exchange intensity as a result of excitation of o_{iu} (interior aryl rotation).

Table S3. Summary of rate constants (a) and energy barriers (b) measured by 1D magnetization transfer experiments in $CDCl_3$ at 298 K.

	Porphyrin rotation			Exterior aryl rotation			Interior aryl rotation		
	k_1	k_{chem}	$\Delta G^{\ddagger}$	k_2	k_{chem}	$\Delta G^{\ddagger}$	k_3	k_{chem}	$\Delta G^{\ddagger}$
**(*c*-P8$_{OOct}$)$_2$**	3.34	6.68	68.3	0.50	1.00	73.0	0.75	1.50	72.0
**(*c*-P12$_{OOct}$)$_2$**	(c)	-	-	(c)	-	-	0.32	0.64	74.1
**(*c*-P8$_{t\text{-}Bu}$)$_2$** (d)	-	-	-	-	-	-	-	-	-
**(*c*-P12$_{t\text{-}Bu}$)$_2$**	(c)	-	-	(c)	-	-	0.88	1.76	71.6

(a)Rate constants in units of s^{-1}. (b)Energy barriers in units of kJ mol^{-1}. (c)Process not observed. (d)Resonances too broad to determine exchange rates.

Section 10. Variable-Temperature ^{1}H NMR Series

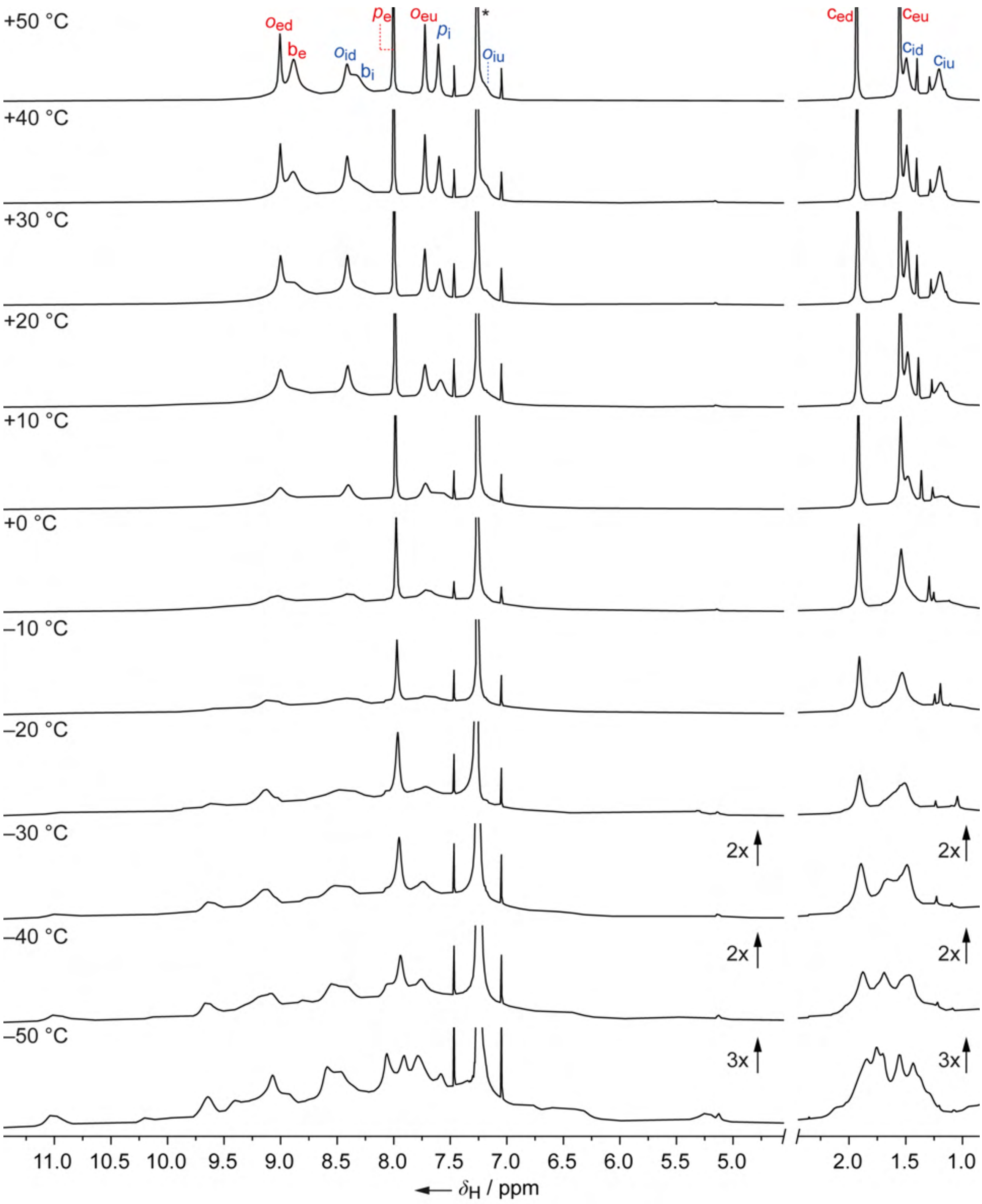

Figure S37. Variable-temperature ^{1}H-NMR (500 MHz, $CDCl_3$) of ***c*-P8**$_{t\text{-Bu}}$ in the absence of pyridine. Abbreviations: * = residual $CHCl_3$ signal.

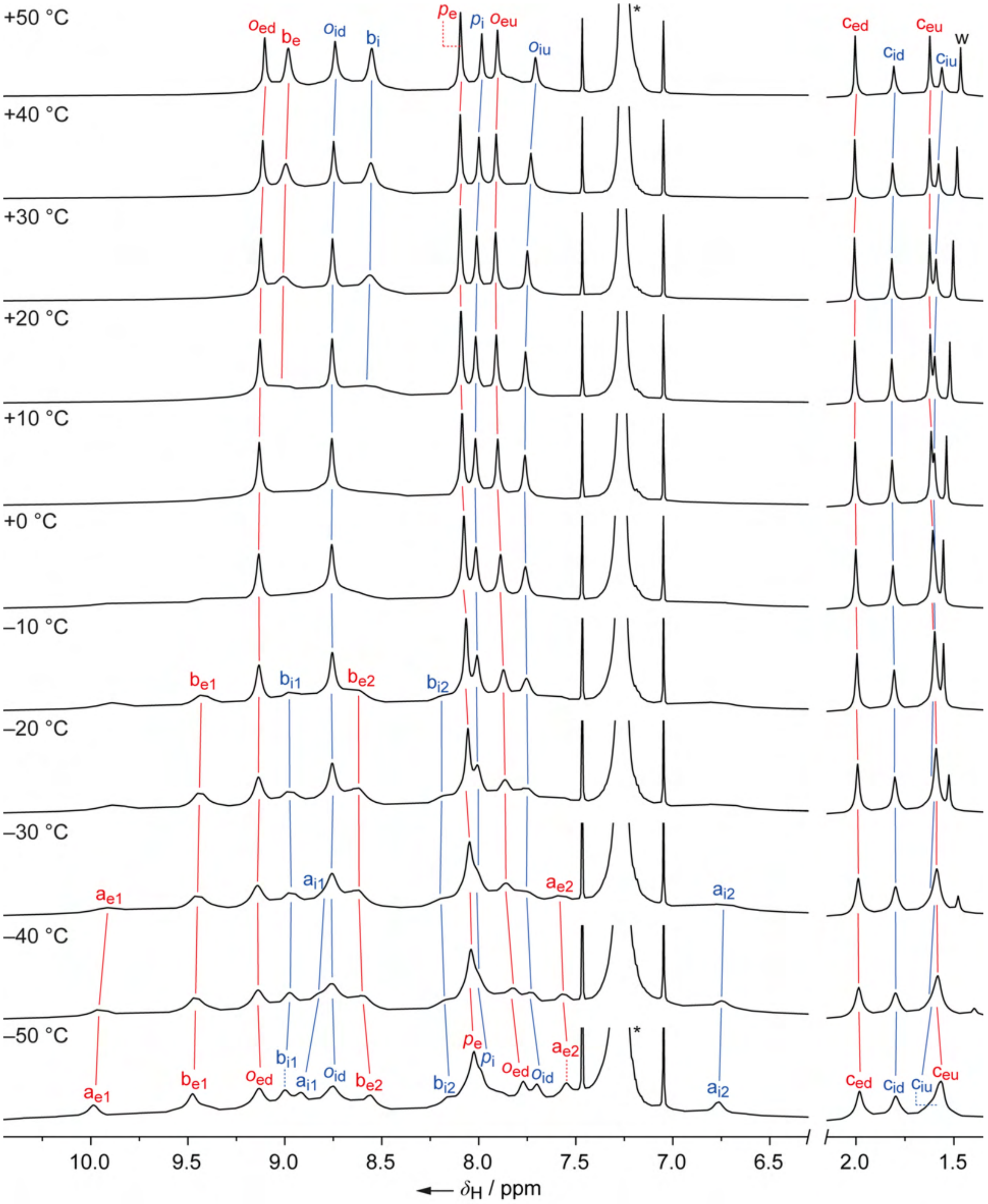

Figure S38. Variable-temperature ^{1}H-NMR (500 MHz, $CDCl_3$) of ***c*-P12$_{t\text{-Bu}}$** in the absence of pyridine. Abbreviations: * = residual $CHCl_3$ signal; w = water.

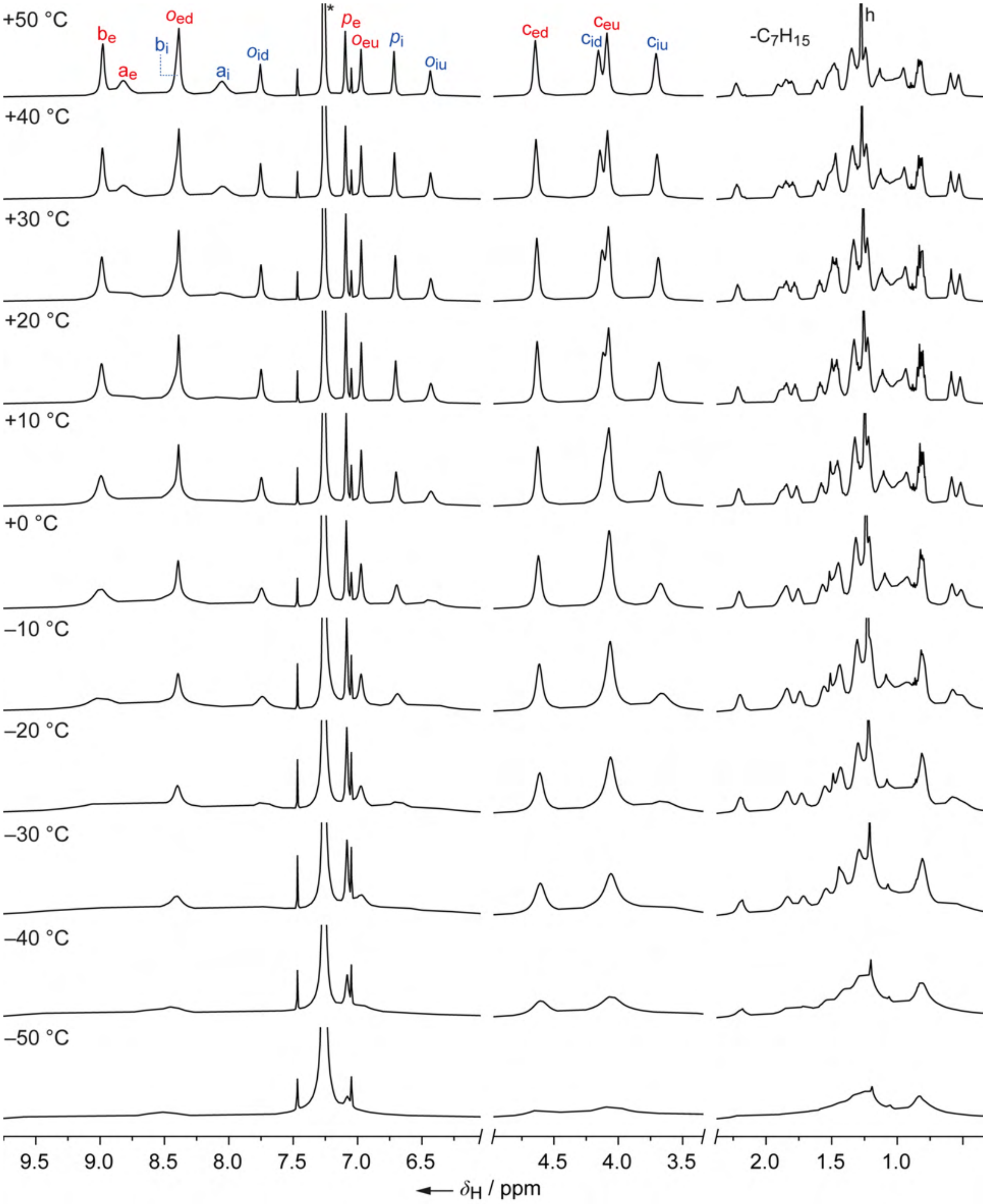

Figure S39. Variable-temperature ^{1}H-NMR (500 MHz, $CDCl_3$) of ***c*-P8$_{OOct}$** in the absence of pyridine. Abbreviations: * = residual $CHCl_3$ signal; h = H-grease.

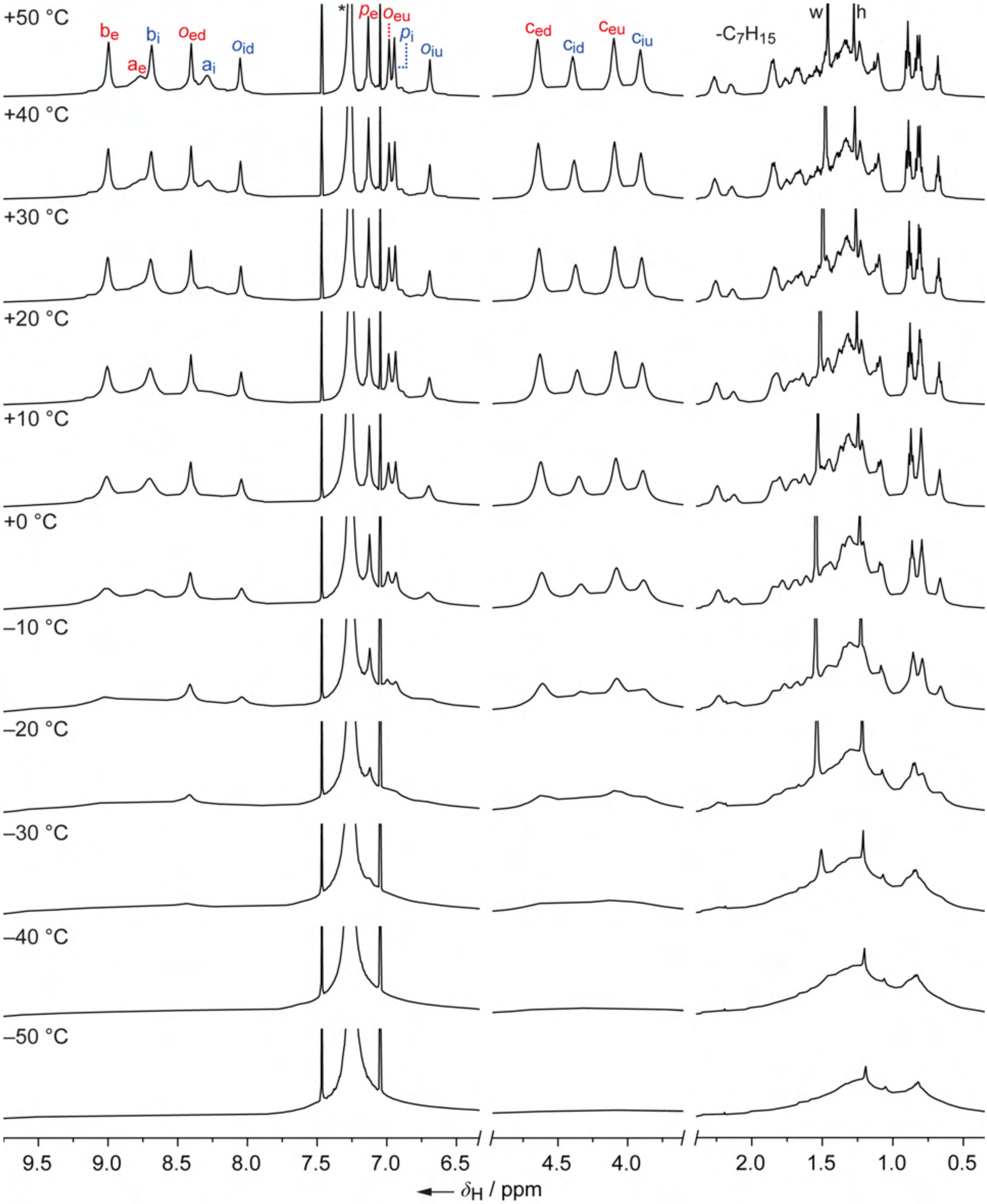

Figure S40. Variable-temperature ^{1}H-NMR (500 MHz, $CDCl_3$) of ***c*-P12$_{\mathbf{OOct}}$** in the absence of pyridine. Abbreviations: * = residual $CHCl_3$ signal; w = water; h = H-grease.

In tetrachloroethane-d_2 (+25 to +140 ℃):

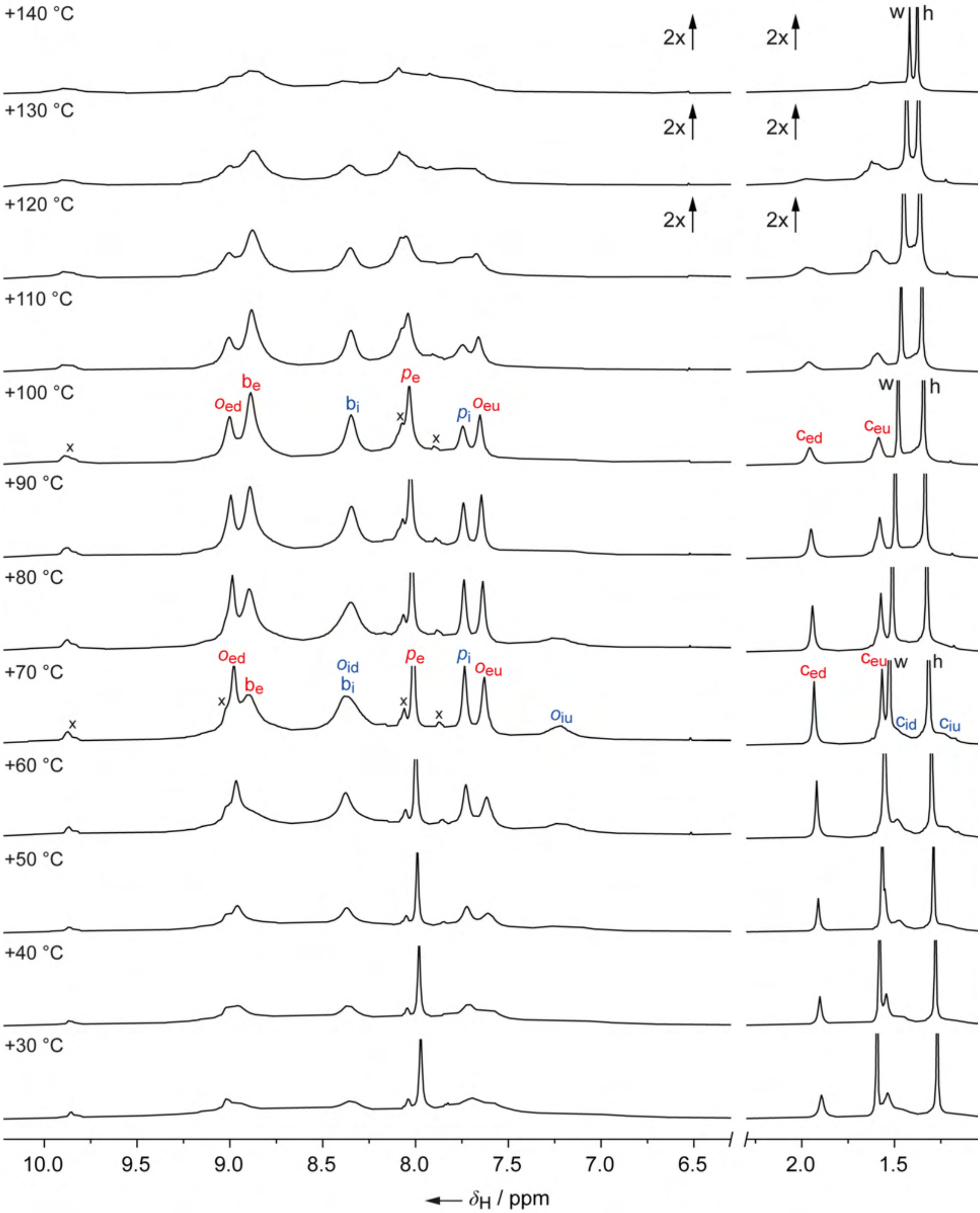

Figure S41. Variable-temperature ^{1}H-NMR (500 MHz, tetrarchloroethane-d_2) of ***c*-P8$_{t\text{-}Bu}$** in the absence of pyridine. Abbreviations: * = residual $CHCl_3$ signal; w = water; h = H-grease.

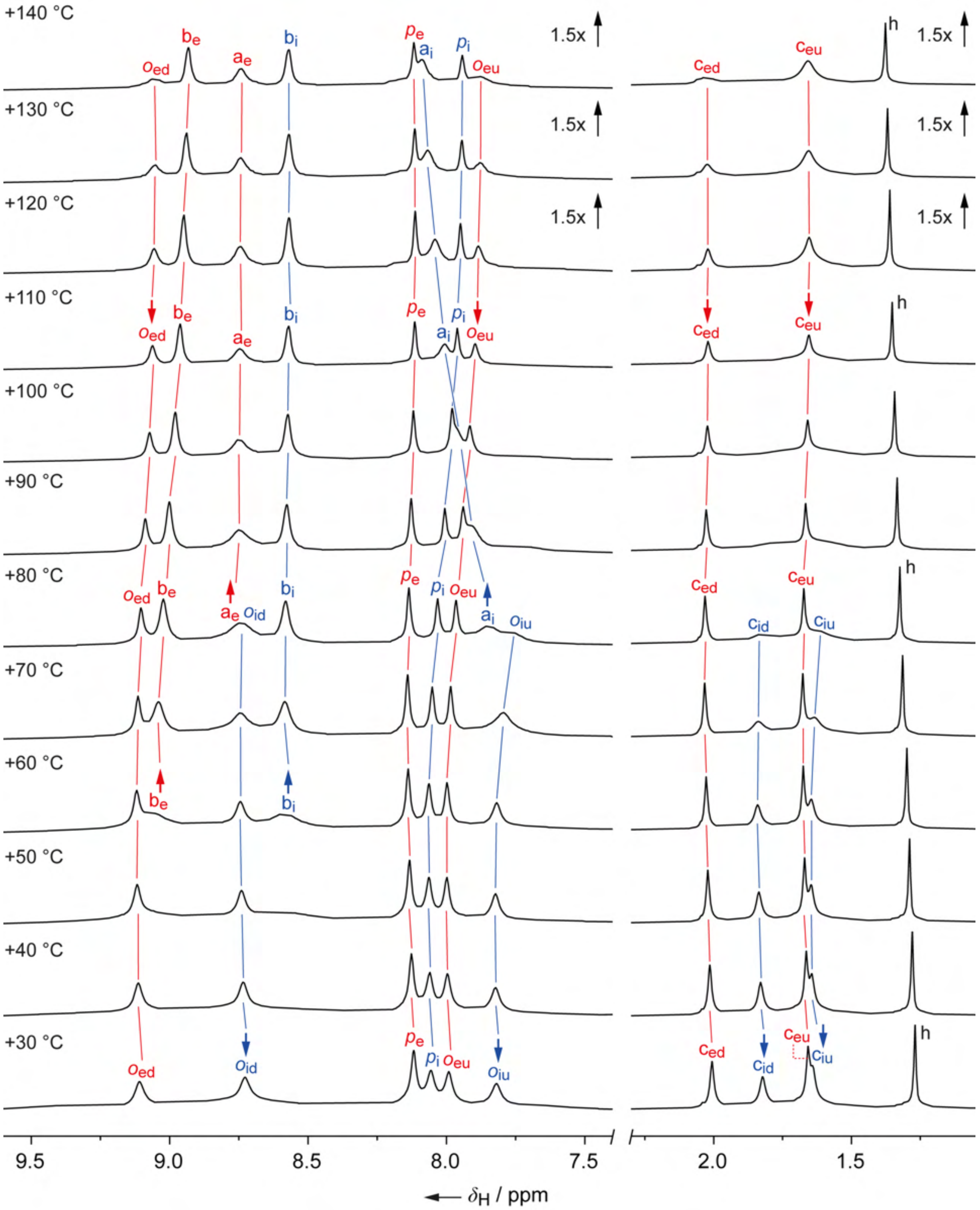

Figure S42. Variable-temperature ^{1}H-NMR (500 MHz, tetrarchloroethane-d_2) of ***c*-P12$_{t\text{-Bu}}$** in the absence of pyridine. Abbreviations: * = residual $CHCl_3$ signal; w = water; h = H-grease.

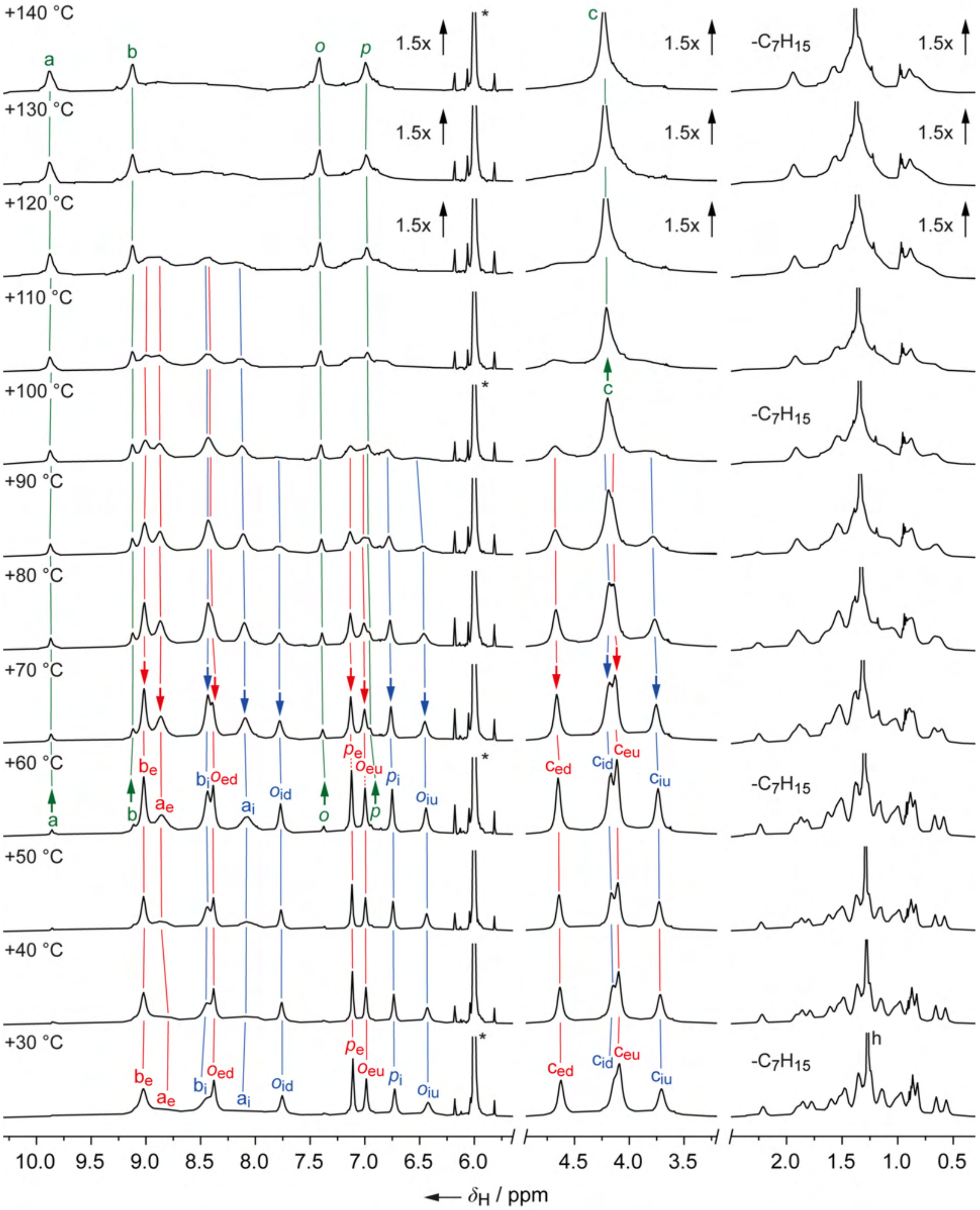

Figure S43. Variable-temperature ^{1}H-NMR (500 MHz, tetrachloroethane-d_2) of ***c*-P8$_{OOct}$** in the absence of pyridine. Abbreviations: * = residual $CHCl_3$ signal; w = water; h = H-grease.

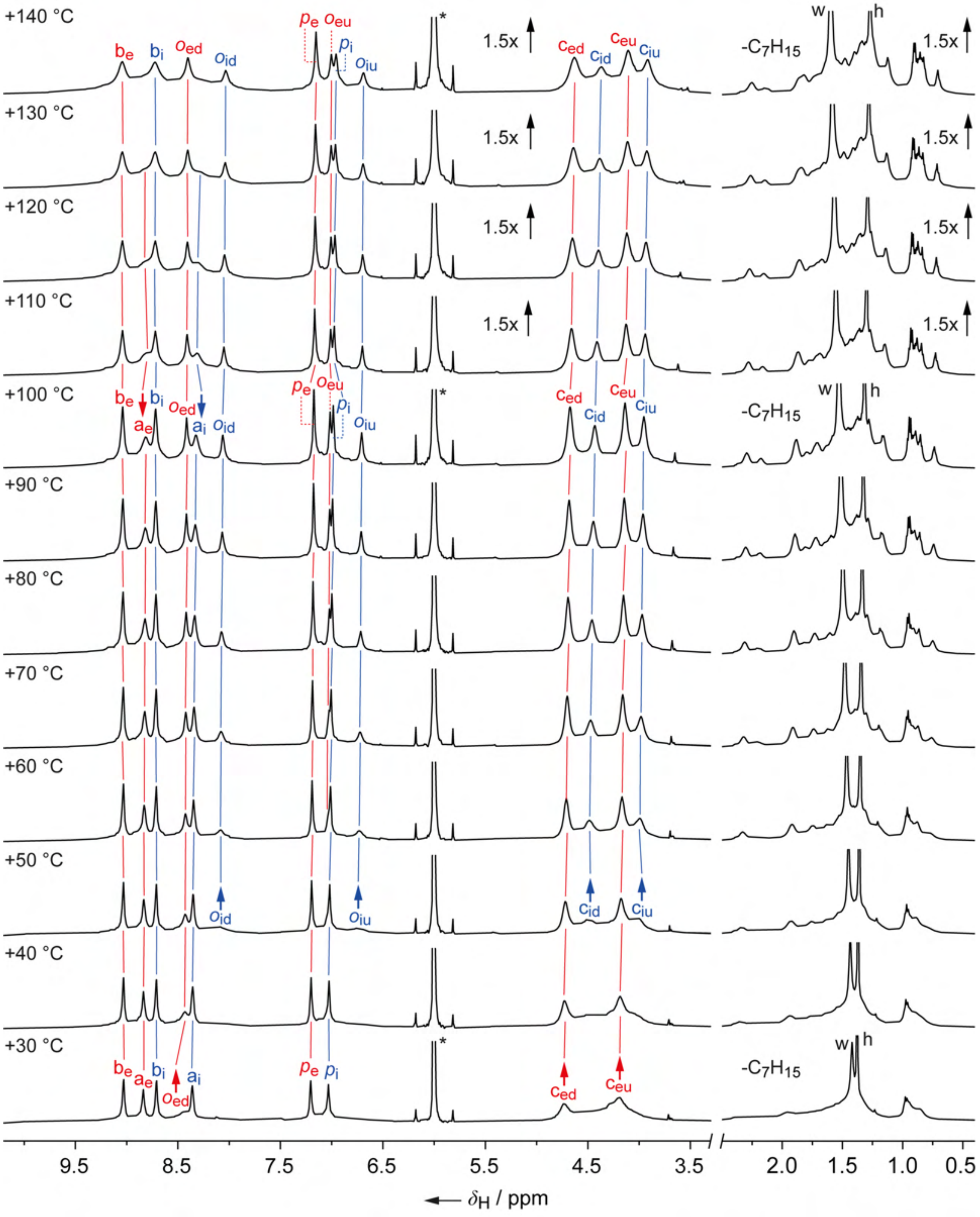

Figure S44. Variable-temperature ^{1}H-NMR (500 MHz, tetrarchloroethane-d_2) of ***c*-P12$_{\mathbf{OOct}}$** in the absence of pyridine. Abbreviations: * = residual $CHCl_3$ signal; w = water; h = H-grease.

Section 11. Comments on the Low-Temperature Assignment of (c-P12$_{t\text{-Bu}}$)$_2$

During cooling of the sample from +50 °C to –50 °C, beta protons b_e and b_i coalesce (at around +10 °C) and eight new signals emerge (see also Section 10, Figure S38 and Figure S45 below). The change is consistent with porphyrin desymmetrization, caused by deceleration of a sliding motion by which each individual porphyrin translates across the closest butadiyne-link of the opposing ring. A barrier is expected for this process, because each porphyrin can form a π-overlap with only one of its two nearest neighbouring porphyrins from the opposing ring.

At room temperature and above this back-and-forth process results in fast exchange of the b-type beta protons. Fast exchange of a-type beta protons is not seen in $CDCl_3$, even at +50 °C, but it may be noted, that in tetrachloroethane-d_2 at higher temperatures, fast exchange is observed for both a- and b-type protons (Figure S42).

The identity of sidechain resonances can be tracked across the temperature series from +50 °C to –50 °C. Therefore, the assignment of interior (p_i, o_{id}, o_{iu}, c_{id}, and c_{iu}) and exterior (p_e, o_{ed}, o_{eu}, c_{ed}, and c_{eu}) resonances is clear, leaving the eight new signals, corresponding to a- and b-type beta protons. From 2D-TOCSY, strong correlations between the beta proton pairs (a/b) are to be expected, and from these observations, it is evident that a_{e1}/b_{e1}, a_{e2}/b_{e2}, and a_{i2}/b_{i2} form three beta proton pairs (Table S4 and Figures S46 and S47). It seems likely that correlations of the last pair (a_{i1}/b_{i1}) are not observed, because they cannot be resolved from the spectrum diagonal. From 2D NOESY, it can be seen which spin environment (interior or exterior) each a/b pair is associated with, based on their correlations to already assigned *ortho*- and *t*-Bu type resonances. As was also found to be the case at 25 °C, NOESY correlations at –50 °C happens to be confined to one of two environments: interior or exterior (Table S4 and Figures S48 and S49).

To assign signals as a- or b- type, we attempted to acquire an HSQC spectrum, but were unable to observe any cross-correlations for any of the ^{1}H signals in the aromatic region, probably because of their large line-width. Based on chemical shift intuition, one may anticipate that the a-type signals will exhibit a larger chemical shift difference ($\Delta\delta_H$) from porphyrin desymmetrization, compared to the b-type signals, because a-type protons will be more exposed to ring-current effects from neighbouring porphyrins in the opposing ring. This has been supported by NMR chemical shift calculations (Section 14).

In terms of ^{1}H chemical shift differences, at –50 °C, we measured: $\Delta\delta_H$ (ppm) = 2.44 (a_{e1}/a_{e2}), 0.57 (b_{e1}/b_{e2}), 2.16 (a_{i1}/a_{i2}), and 0.85 (b_{i1}/b_{i2}). The assignment of a- and b-type protons also makes sense considering their chemical shift values under fast- and slow-exchange. In terms of b-type protons, the midpoint of the b_{e1}/b_{e2} pair (δ_H = 9.02 ppm), measured at –50 °C, is comparable to that of the averaged signal b_e (δ_H = 8.95 ppm), measured at +50 °C. Similarly, the midpoint of the b_{i1}/b_{i2} pair (δ_H = 8.53 ppm), measured at –50 °C, is comparable to that of the averaged signal b_i (δ_H = 8.58 ppm), measured at +50 °C. In terms of a-type protons, the mid-point of pairs a_{e1}/a_{e2} (δ_H = 8.77 ppm) and a_{i1}/a_{i2} (δ_H = 7.85 ppm) measured at –50 °C are also comparable to the chemical shifts seen for the averaged signals a_e (δ_H = 8.74 ppm) and a_i (δ_H = 8.09 ppm), respectively, measured at +50 °C, although the latter averaged signals are measured in tetrachloroethane-d_2 at 140 °C. The shift of interior a_i deviates more, but the high-temperature VT-series also suggests this resonance to exhibit a higher temperature dependence, compared to exterior a_e (Figure S42).

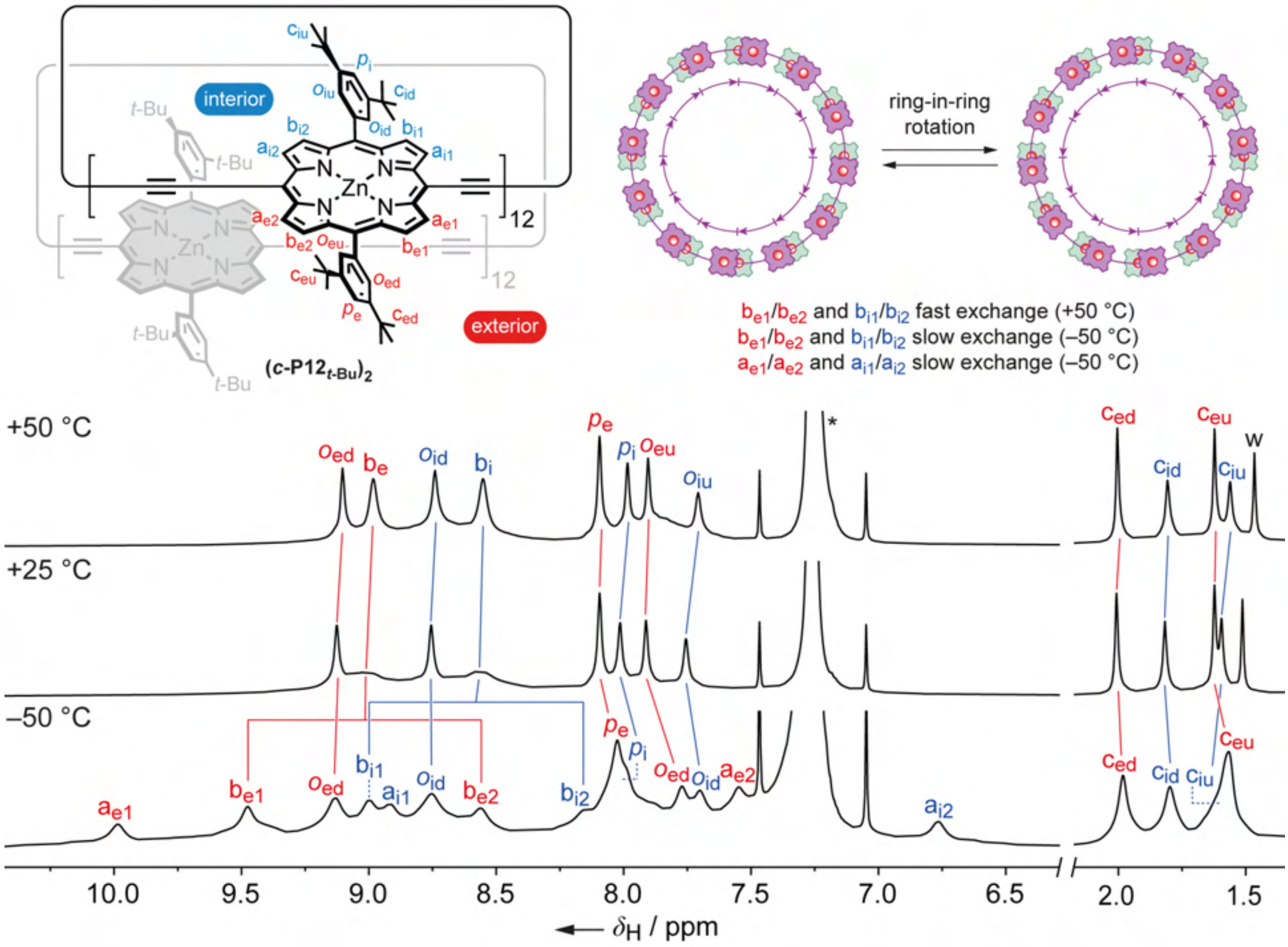

Figure S45. Summary of the change in the ^{1}H NMR spectrum of **(*c*-P12$_{t\text{-Bu}}$)$_2$** between +50 and –50 °C (500 MHz, $CDCl_3$). Full VT-series shown in Section 10, Figure S42. * = residual $CHCl_3$; w = water.

Table S4. Overview of NOESY (left) and TOCSY (right) correlations of **(*c*-P12$_{t\text{-Bu}}$)$_2$** at –50 °C (500 MHz, $CDCl_3$).

	a_{e1}	b_{e1}	o_{ed}	b_{i1}	a_{i1}	o_{ed}	b_{e2}	b_{i2}	p_e	p_i	o_{eu}	o_{iu}	a_{e2}	a_{i2}	c_{ed}	c_{id}	c_{iu}	c_{eu}	
c_{eu}		w	w				w		s		s		w		m				
c_{iu}				w		m				s		s				s			
c_{id}				s	s	s		s		s				m					
c_{ed}	w	m	s				s		s	m			m						
a_{i2}				s	s	m		m	m			w							
a_{e2}	m	m	si				s				m								
o_{iu}				w	w	w		w		m							s		
o_{eu}	w	m	m				m		m									s	
p_i				w	w	m										s	s		
p_e		w	m				w								s			s	
b_{i2}				m	m	m								m					
b_{e2}	w	m	m										s						
o_{ed}				s	s												m		
a_{i1}														w			w		
b_{i1}														w					
o_{ed}	m	s							w						s				
b_{e1}	s																		
a_{e1}		s											w						

Relative strengths of NOE (above diagonal) and TOCSY (below diagonal) correlations indicated as s = strong, m = medium, w = weak.

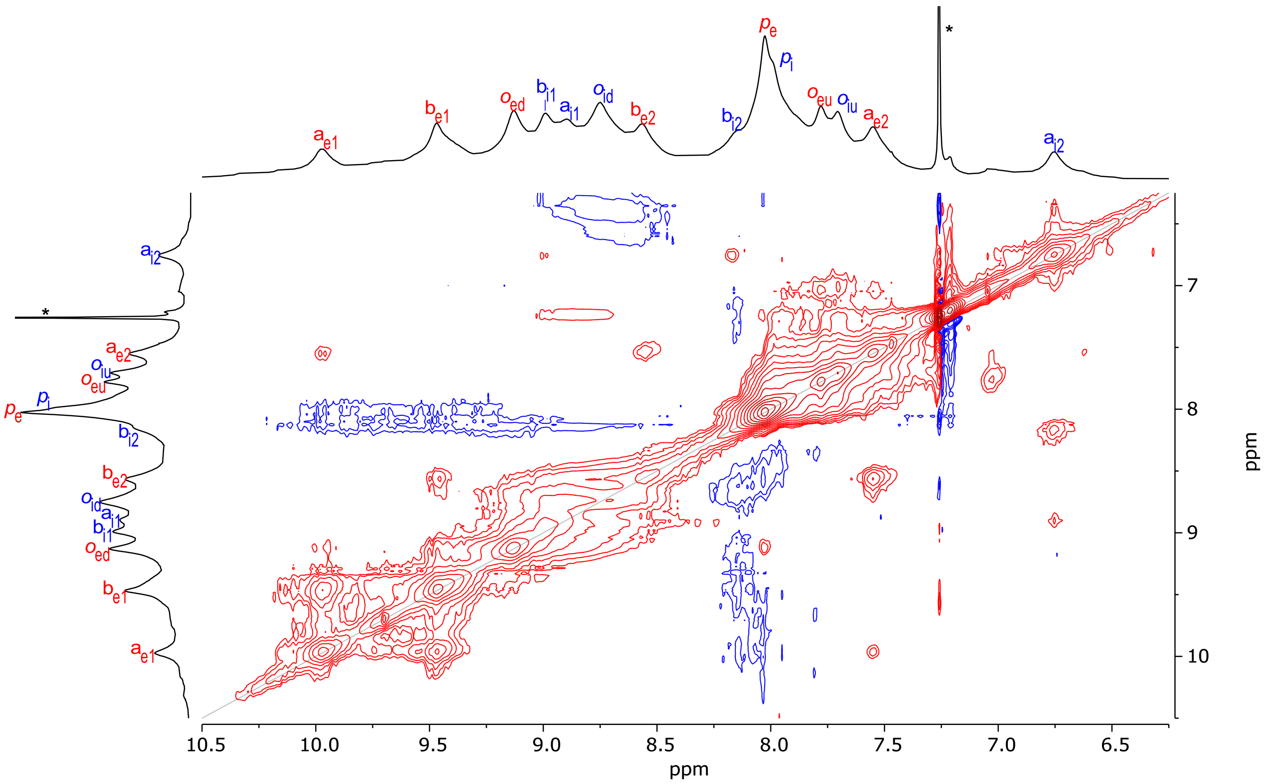

Figure S46. Expanded region of the TOCSY spectrum of **(*c*-P12$_{t\text{-Bu}}$)$_2$** (500 MHz, $CDCl_3$, –50 °C, mixing time = 40 ms) showing correlations between aromatic protons. * = residual $CHCl_3$.

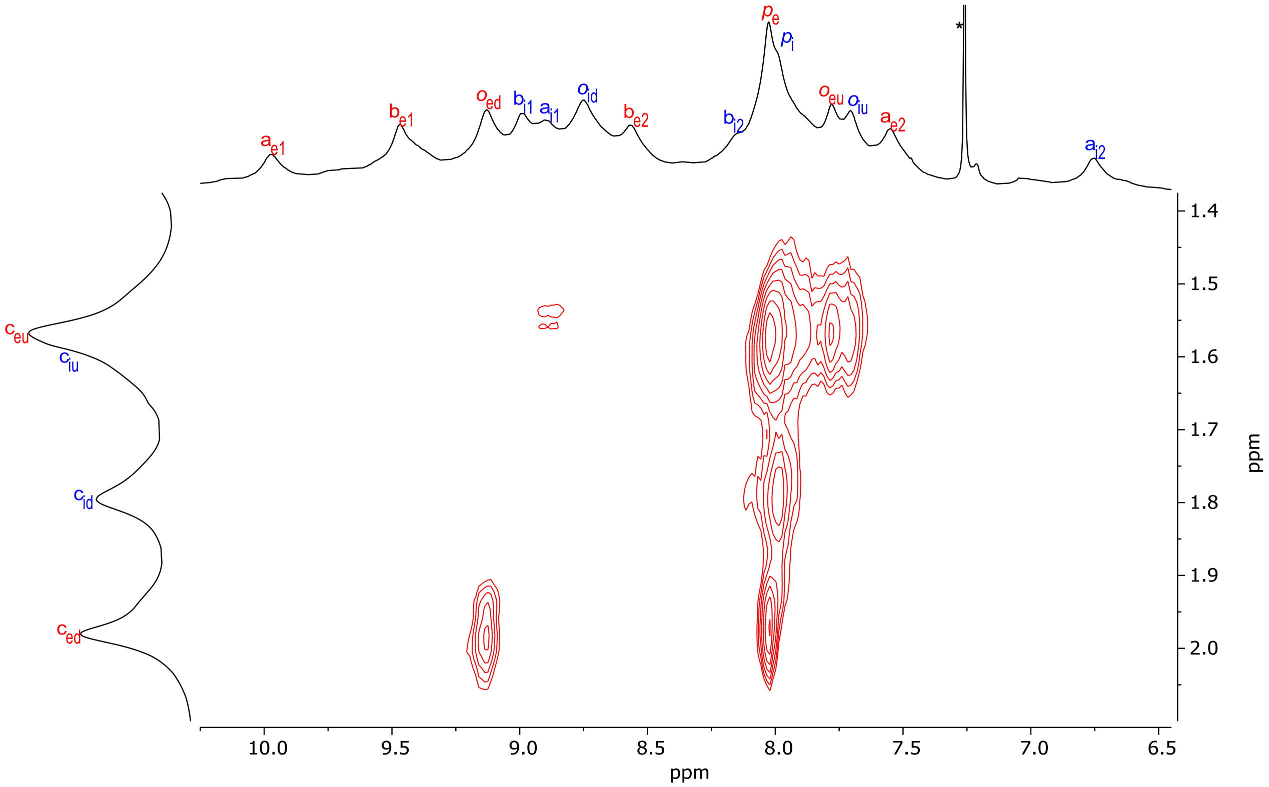

Figure S47. Expanded region of the TOCSY spectrum of **(*c*-P12$_{t\text{-Bu}}$)$_2$** (500 MHz, $CDCl_3$, –50 °C, mixing time = 40 ms) showing correlations between aromatic and *t*-Bu protons. * = residual $CHCl_3$.

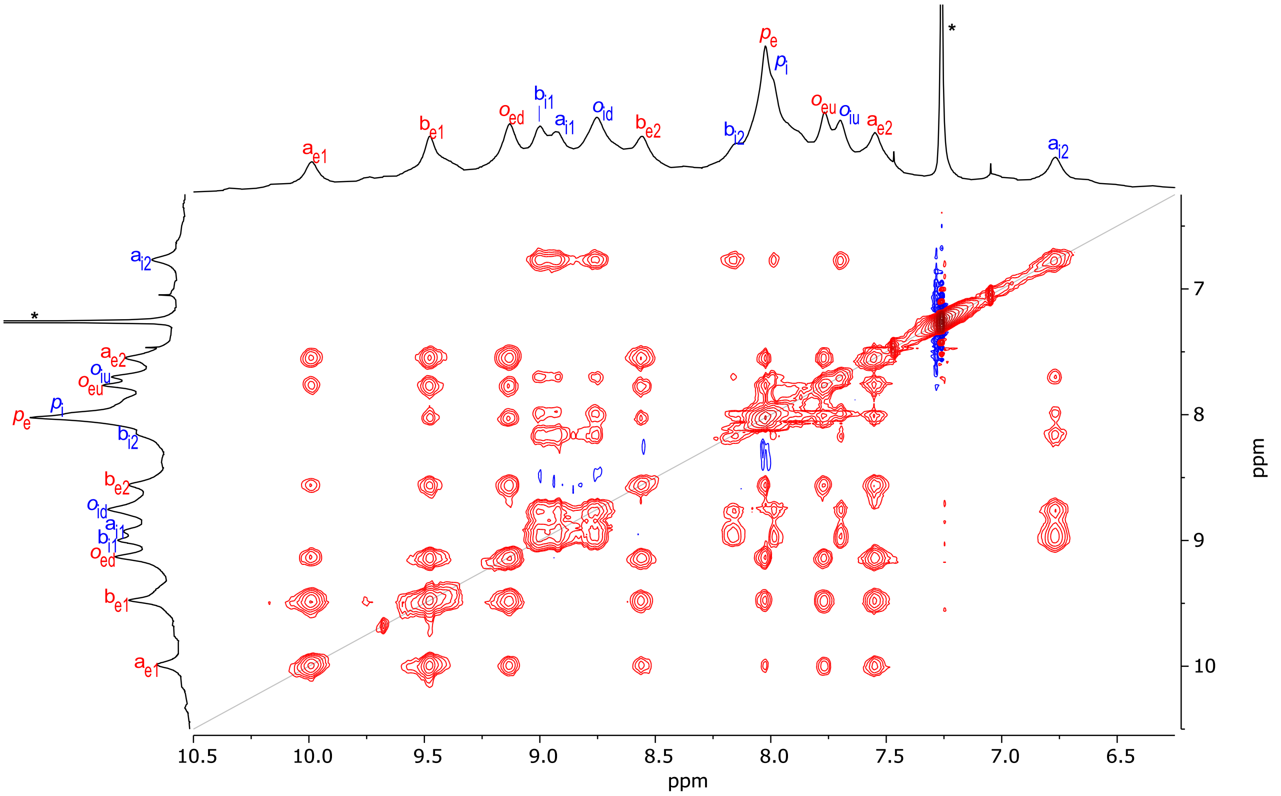

Figure S48. Expanded region of the NOESY spectrum of **(*c*-P12$_{t\text{-Bu}}$)$_2$** (500 MHz, $CDCl_3$, –50 °C, mixing time = 300 ms) showing correlations between aromatic protons.

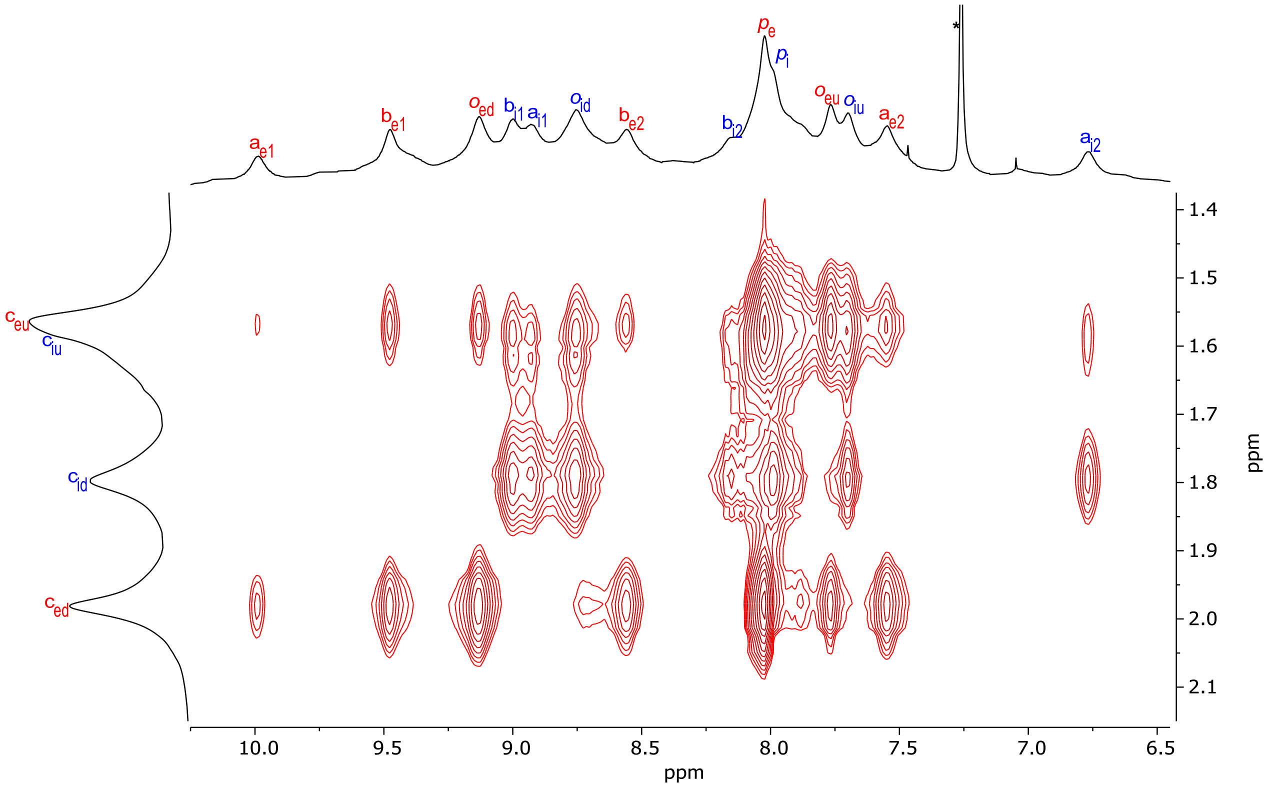

Figure S49. Expanded region of the NOESY spectrum of **(*c*-P12$_{t\text{-Bu}}$)$_2$** (500 MHz, $CDCl_3$, –50 °C, mixing time = 300 ms) showing correlations between aromatic and *t*-Bu protons.

Section 12. Rate and Activation Barrier for the Ring Rotation in (*c*-P12$_{t\text{-Bu}}$)$_2$

We have measured the rate and activation barrier of ring rotation at two temperatures: 1) at –50 °C under slow-exchanging conditions via 2D-EXSY using the b_{e1} ↔ b_{e2} proton pair, and 2) at +10 °C, the coalescence temperature of the b-type proton pairs b_{e1} ↔ b_{e2} and b_{i1} ↔ b_{i2}. This enabled us to crudely estimate the rate and activation barrier for ring rotation.

Firstly, at –50 °C, the beta proton pairs are in the slow-exchanging regime. As a result, exchange correlations are expected between beta protons: a_{e1} ↔ a_{e2}, b_{e1} ↔ b_{e2}, a_{i1} ↔ a_{i2}, and b_{i1} ↔ b_{i2}, which may be observed by 2D-EXSY. Yet, since the porphyrin units have desymmetrized across the center, NOEs may also contribute to observable correlations within these proton pairs (Figure S50 below). Based on intra- and intermolecular distance measurements on a minimized geometry of the ***c*-P12$_{t\text{-Bu}}$** dimer, we found that the b_{e1} ↔ b_{e2} pair is expected to be the pair least affected by NOE contributions (Table S5 below). This was further supported by 2D-ROESY, which showed a strong exchange cross-peak for this proton pair (Figure S51 below). To determine the rate of exchange at –50 °C, the evolution of magnetization transfer within b_{e1} ↔ b_{e2} was measured at different mixing times: 25, 35, 50, 75, 100, 125, 150, 175, and 200 ms by 2D-EXSY (Figure S52). The evolution of magnetization at b_{e1} and b_{e2} was normalized using the total peak volume of their integrated cross- and diagonal peaks and plotted against mixing time (Table S6 and Figure S53 below). The exchange rate constant was obtained by fitting these data to a 2-state exchange model using the Berkeley Madonna Software.[12]

Secondly, at the temperature of signal coalescence, T_c, the rate of exchange may be determined if the original chemical shift separation ($\Delta\nu$) is known (Eqn. S6). We measured the original chemical shift separation for beta-proton pairs b_{e1} ↔ b_{e2} and b_{i1} ↔ b_{i2} at –50 °C, in order to determine the rate of exchange at the point of coalescence (T_c = +10 °C) between these signals (Section 10, Figure S38).

$$k_{exc} = \frac{\pi\cdot\Delta\nu}{\sqrt{2}} \qquad \text{(Eqn. S6)}$$

As reported by Green and co-workers for a 2-state exchange process of equal populations, the rate of exchange is only half the rate of the physical process giving rise to that exchange process (see also Section 9 on exchange).[13] Hence the rate of ring rotation is related to the rate of exchange as given by Eqn. S7:

$$k_{rot} = 2k_{exc} \qquad \text{(Eqn. S7)}$$

The rate of ring rotation, k_{rot}, is related to the Gibbs energy of activation for the exchange event, $\Delta G_{rot}^{\ddagger}$, via the Eyring equation (Eqn. S8):

$$k_{rot} = \frac{k_B\cdot T}{h} e^{\frac{-\Delta G^{\ddagger}}{R\cdot T}} \qquad \text{(Eqn. S8)}$$

where, R is the gas constant, T the temperature, h the Planck constant, and k_B the Boltzmann constant. Rates and activation barriers are summarized in Table S7.

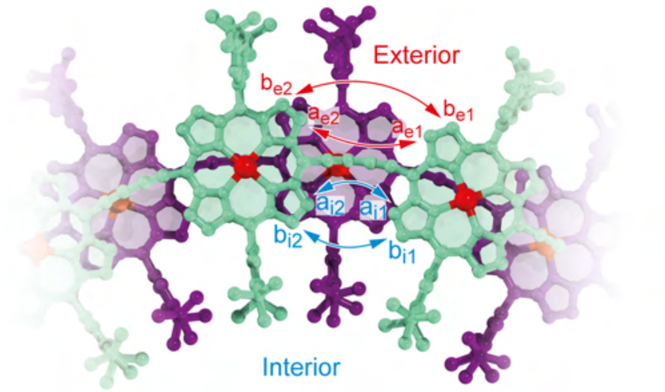

Figure S50. 1/8th fragment of the ***c*-P12*t*-Bu** dimer model including highlighted beta protons, affected by ring-in-ring rotation. Each pair of beta protons may have some intra- and some intermolecular contribution to an NOE correlation, in addition to an exchange correlation. Only the intramolecular correlation is indicated with double arrows. Intra- and intermolecular distances shown in Table S5 below were measured to evaluate the expected extent of NOE vs. exchange contribution towards the cross correlation within each beta proton pair.

Table S5. Intra- and intermolecular proton-proton distances of beta protons, measured using the optimized ***c*-P12*t*-Bu** dimer model. The b_{e1} ↔ b_{e2} correlation is most suitable for following the exchange process, because the signal contribution due to NOEs is expected to be the smallest out of the four proton pairs.

Beta H–H correlations	Intramolecular distance (calc) / Å	Intermolecular distance (calc) / Å	Effective distance (calc)[a] / Å
a_{e1} ↔ a_{e2}	5.8	6.6	5.4
b_{e1} ↔ b_{e2}	10.4	6.8	6.7
a_{i1} ↔ a_{i2}	3.1	6.0	3.1
b_{i1} ↔ b_{i2}	5.8	5.7	5.1

[a] Calculated using both intra- and intermolecular distances, according to the formula in Section 7 and assuming a $1/r^6$ distance dependence.

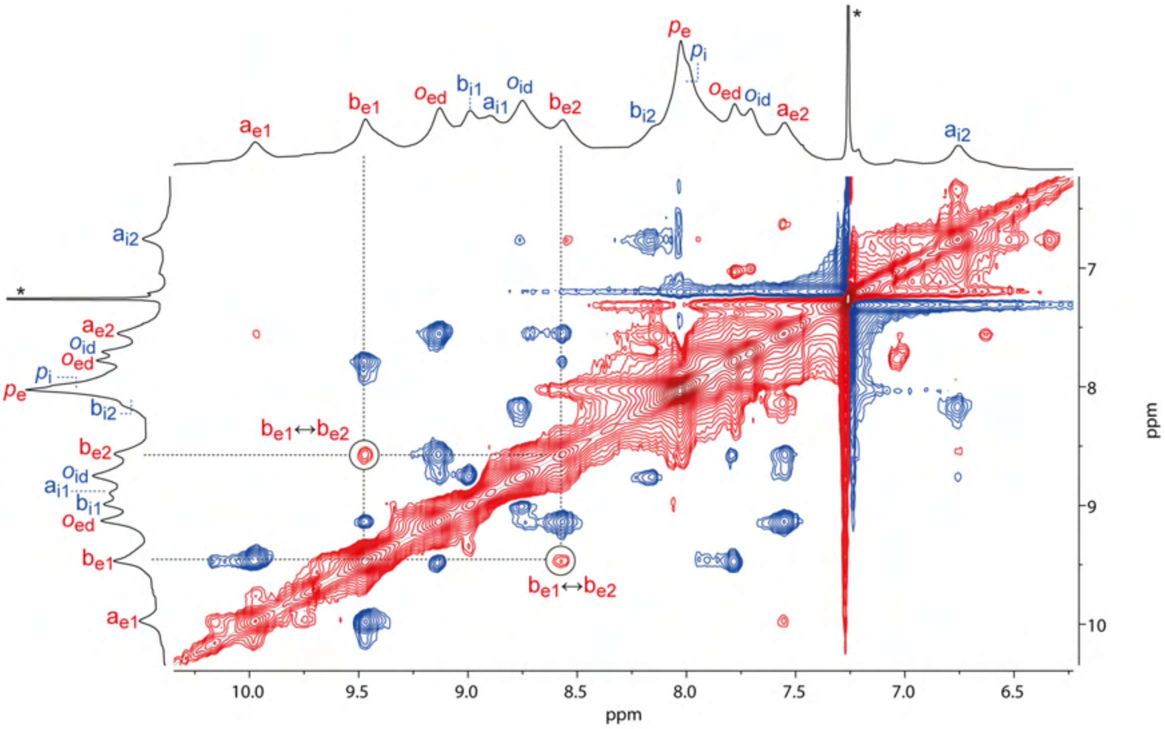

Figure S51. Selected region from the 2D-ROESY of the ***c*-P12*t*-Bu** dimer (500 MHz, $CDCl_3$, –50 °C, mixing time = 50 ms). The exchange cross-peak between b_{e1} and b_{e2} was used to estimate the exchange rate, resulting from ring rotation.

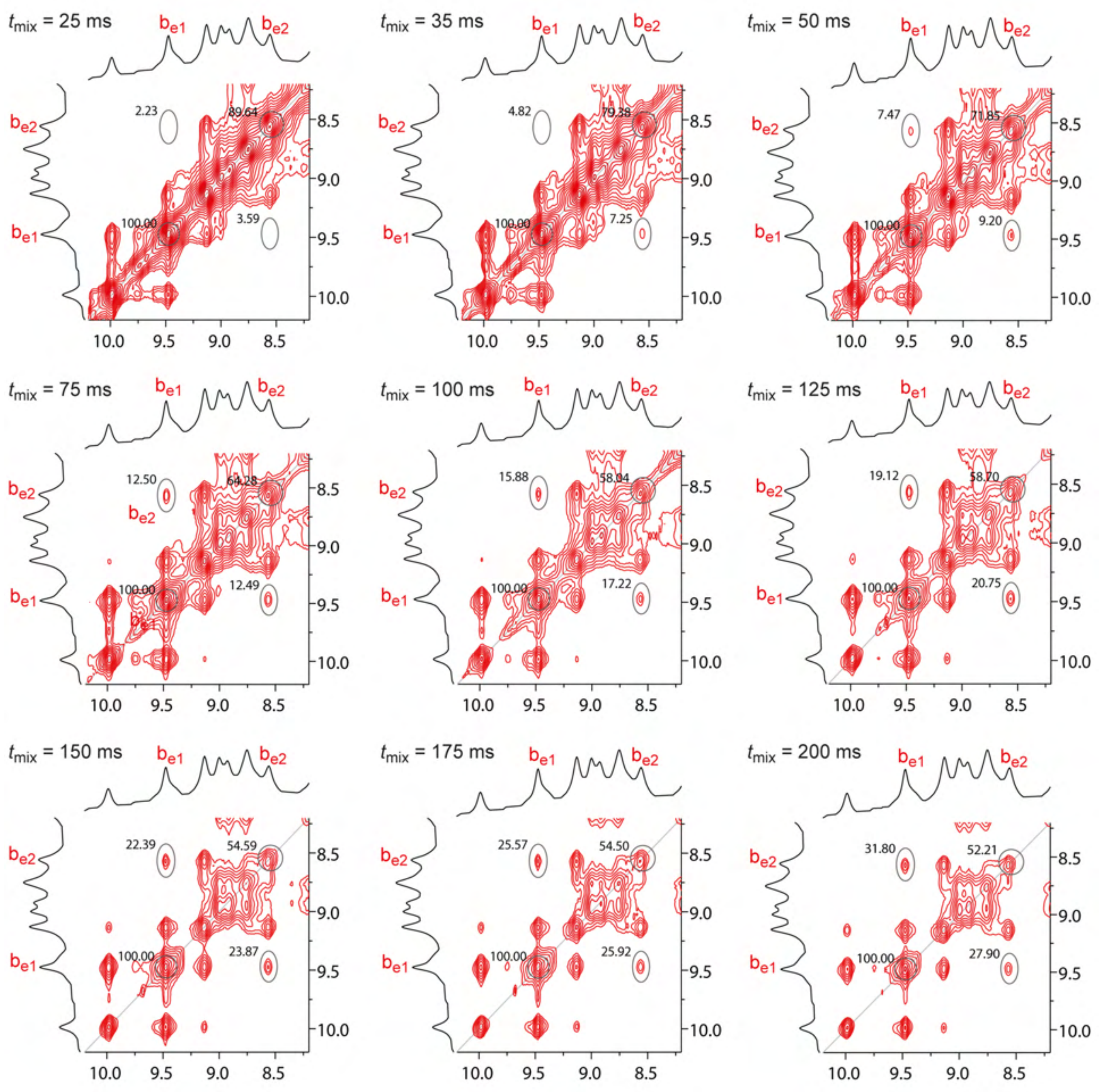

Figure S52. Series of 2D-NOESY (EXSY) spectra acquired at different mixing times (t_{mix} = 20–200 ms) to estimate the rate of exchange due to ring rotation. The diagonal peaks b_{e1} ↔ b_{e1} and b_{e2} ↔ b_{e2} were integrated using the ranges [δ_{f2} = 9.57–9.38; δ_{f1} = 9.57–9.38] and [δ_{f2} = 8.65–8.43; δ_{f1} = 8.65–8.43], respectively, throughout the series. The b_{e1} ↔ be1 cross peaks were integrated using the ranges [δ_{f2} = 9.55–9.40; δ_{f1} = 8.71–8.42] (above the diagonal) and [δ_{f2} = 8.63–8.48; δ_{f1} = 9.60–9.34] (below the diagonal) throughout the series.

Table S6. Mixing times and integrated diagonal and cross peak volumes used to estimate the exchange rate from ring rotation.

t_{mix} / s	b_{e1}↔b_{e1} diagonal volume	b_{e2}↔b_{e2} diagonal volume	b_{e1}↔b_{e2} Cross peak volume[a]	Total signal volume 1[b]	Total signal volume 2[c]	Normalized b_{e1}↔b_{e1} diagonal volume[d]	Normalized b_{e1}↔b_{e2} Cross peak volume 1[d]	Normalized b_{e2}↔b_{e2} diagonal volume[e]	Normalized b_{e1}↔b_{e2} Cross peak volume 2[e]
20	100	90	2.9	102.9	92.9	0.97182	0.96878	0.02818	0.03122
35	100	79	6.05	106.05	85.05	0.94295	0.92887	0.05705	0.07113
50	100	72	8.35	108.35	80.35	0.92293	0.89608	0.07707	0.10392
75	100	64	12.5	112.5	76.5	0.88889	0.8366	0.11111	0.1634
100	100	58	16.5	116.5	74.5	0.85837	0.77852	0.14163	0.22148
125	100	59	20	120	79	0.83333	0.74684	0.16667	0.25316
150	100	55	23	123	78	0.81301	0.70513	0.18699	0.29487
175	100	55	26	126	81	0.79365	0.67901	0.20635	0.32099
200	100	52	30	130	82	0.76923	0.63415	0.23077	0.36585

[a] Average of cross peak volumes above and below the diagonal. [b] Sum of b_{e1} ↔ b_{e1} diagonal and b_{e1} ↔ b_{e2} cross peak volumes. [c] Sum of b_{e2} ↔ b_{e2} diagonal and b_{e1} ↔ b_{e2} cross peak volumes. [d] Normalized using total signal volume 1. [e] Normalized using total signal volume 2.

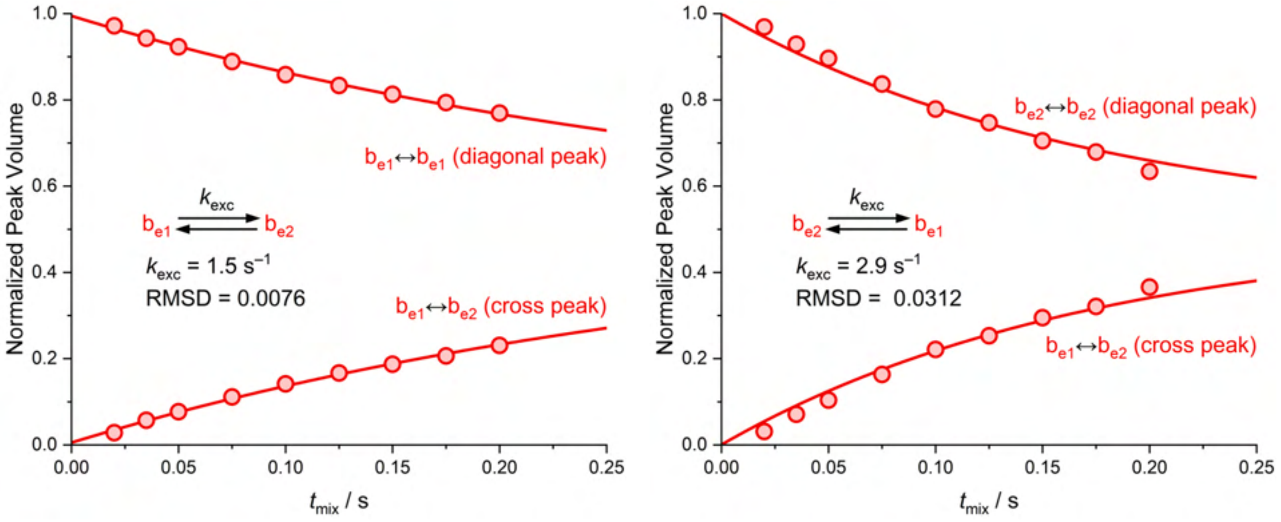

Figure S53. Fitting of the normalized signal volumes versus mixing time for the exchange correlation b_{e1} ↔ b_{e2}. Left: the b_{e1} ↔ b_{e1} diagonal peak volume was used. Right: the b_{e2} ↔ b_{e2} diagonal volume was used. In both cases, the average cross-peak volume from above and below the diagonal is used for the off-diagonal, b_{e1}↔ b_{e2} correlation. The signal evolutions were fitted to a two-state model as shown to obtain two values for the exchange rate constant (k_{exc}). The obtained rate of exchange, corresponds to one half the rate of rotation (i.e., $k_{rot} = 2k_{exc}$), since only 50% of ring rotations result in a measurable cross-peak. The rate of rotation is estimated to be k_{rot} = 4.4 s^{-1} (± 1.98), using the average value of the two exchange rate constants obtained from fitting (k_{exc} = 1.5^{-1} and k_{exc} = 2.9 s^{-1}). The associated energy barrier for this rotation process is estimated to be, $\Delta G_{rot}^{\ddagger}$ = kJ mol^{-1} (± 0.8).

Table S7. Summary of experimental parameters used to determine the rate and activation barrier of ring rotation at 223 K and 283 K.

T / °K (°C)	H–H pair	$\Delta\nu$ / Hz (ppm)	k_{exc} / s^{-1}	k_{exc} (averaged) / s^{-1}	k_{rot} / s^{-1}	$\Delta G^{\ddagger}$ / kJ mol^{-1}
283 (10)	b_{e1} ↔ b_{e2}	459 (0.92)	1.02·10^3	9.80·10^2 ± 0.56·10^2	1960 ± 112	51.4 ± 0.1
	b_{i1} ↔ b_{i2}	425 (0.85)	9.4·10^2			
223 (–50)	b_{e1} ↔ b_{e2}	-	1.5	2.2 ± 0.99	4.4 ± 1.98	51.3 ± 0.8
	b_{e2} ↔ b_{e1}	-	2.9			

Section 13. Residual Dipolar Coupling Measurements of (c-P12$_{t\text{-Bu}}$)$_2$

The total one-bond H-C coupling constants ($^1T_{CH}$) were measured for the distinct side chain protons including those belonging to the *tert*-butyl groups at four different field strengths (500, 600, 700, and 950 MHz). It has not been possible to observe H-C correlations for any of the beta protons due to the broad nature of these signals. The ^{13}C-coupled HSQC spectra are shown in Figure S54-S61. The $^1T_{CH}$ values were measured using 1D traces of the F2 (^{1}H) dimension from the ^{13}C-coupled HSQC spectra. The $^1T_{CH}$ values obtained for each proton environment has been plotted against the magnetic field strength squared, in order to determine both scalar and dipolar coupling constants ($^1T_{CH} = {}^1J_{CH} + B_0^2 \cdot {}^1D_{CH}$). The J coupling is obtained as the y-intercept at $B_0 = 0$ Tesla. Linear fits for each of the proton resonances are shown in Figures S62. All coupling constants are summarised in Table S8 and S9.

We first measured the dimer and monomer of ***c*-P12$_{t\text{-Bu}}$**, i.e., in the absence and presence of pyridine-d_5. No residual dipolar couplings were detected in the case of monomeric ***c*-P12$_{t\text{-Bu}}$** in the presence of pyridine, as evidenced from the lack of a field strength dependence of $^1T_{CH}$ (Figure S62). We also looked for residual dipolar couplings in the ***c*-P8$_{OOct}$** and ***c*-P12$_{OOct}$** dimers. In case of the ***c*-P8$_{OOct}$** dimer, we did not observe a field-dependence of $^1T_{CH}$. In case of the ***c*-P12$_{OOct}$** dimer, it was too difficult to accurately measure $^1T_{CH}$ at different field strengths, due to the broadness of peaks in the ^{1}H dimension. Similarly, this was also the case for the ***c*-P8$_{t\text{-Bu}}$** dimer.

The residual dipolar couplings provide orientational information about the structure of the ***c*-P12$_{t\text{-Bu}}$** aggregate. The sign and magnitude of the dipolar coupling, D_{ab}, between two nuclei ‘a’ and ‘b’ depends on the angle θ between their internuclear vector and the direction of the external magnetic field, according to Eqn. S9:

$$D_{ab} = D_{max} \cdot \left\langle \frac{3cos^2\theta - 1}{2} \right\rangle = \frac{\mu_0 \cdot h \cdot \gamma_a \cdot \gamma_b}{(2\pi r_{ab})^3} \cdot \left\langle \frac{3cos^2\theta - 1}{2} \right\rangle \qquad \text{(Eqn. S9)}$$

where μ_0 denotes the permittivity in vacuum, h the Planck constant, γ_a and γ_b the gyromagnetic ratios of the nuclei, and r_{ab} the distance between the involved nuclei.[15,16] To evaluate the aggregate structure, we used the observed RDCs together with different geometries of the ***c*-P12$_{t\text{-Bu}}$** dimer as inputs for the PALES (Prediction of ALignmEnt from Structure) programme by Zweckstetter.[17] PALES was used to calculate the molecular alignment tensor and residual dipolar couplings in an iterative manner, to obtain the best fit to experimental RDCs. Using the idealized model of the ***c*-P12$_{t\text{-Bu}}$** dimer with porphyrin units in the plane of the nanorings and sidechain dihedral angles from xTB energy minimization, we obtained a good fit to the measured RDCs (Q of 0.097) as shown in Figure S63. We also screened different structural configurations close to this structure, by varying either the interior or exterior dihedrals (between 70–110°) (Table S10 and Figures S64 and S65), or the porphyrin angles (between –10° and +10°) relative to the plane of the rings (Table S11 and Figures S66 and S67). Rotation of the interior dihedral angle with the exterior dihedral being fixed or vice versa, gave in both cases a parabola shaped trend with a minimum in Q at a 90° dihedral for the sidechain being rotated (Q = 0.092 with the interior side being fixed at 88°; Q = 0.096 with the exterior side being fixed at 82°). When the porphyrin units are twisted out of the plane of the nanorings, we found two shallow Q-minima on opposite sides of the flat conformation (0 °, Q = 0.097) at an angle of –4° (Q = 0.096) and +4° (Q = 0.095), shown in Figure S66. Finally, we fixed the interior and exterior dihedral angles at 90° and twisted the porphyrin units out of the plane of the nanorings (Table S11), which gave a similar double parabola with Q-minima at –4° and +4° (Q = 0.090 in both cases). Altogether, the analysis suggests the model to be a good fit to the experimental RDCs allowing for some extent of structural flexibility in terms of the sidechain dihedral angles and the angle of the porphyrin units, which is to be expected for these nanoring dimers.

Below are also included a clarification of the labelling system used for the PALES input files is given in Figure S69.

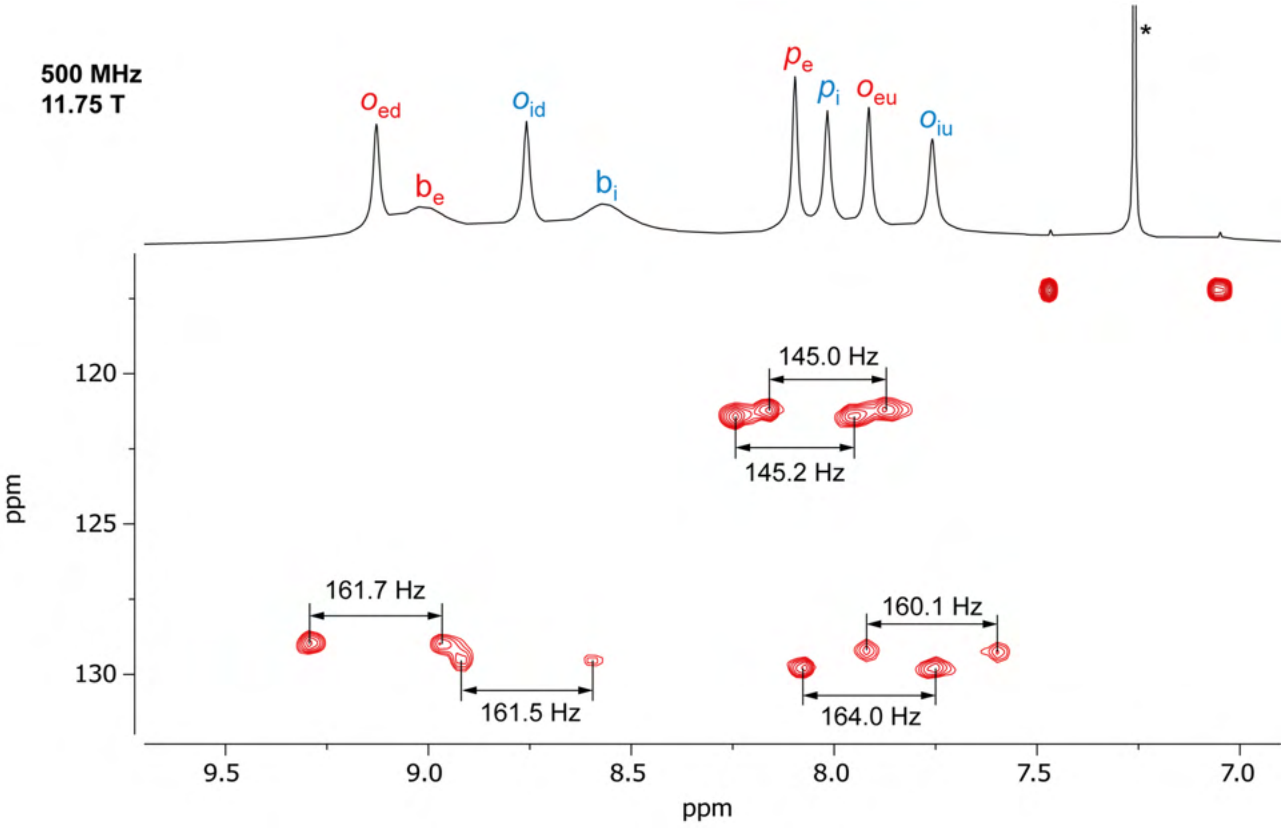

Figure S54. ^{1}H-^{13}C coupled HSQC (500 MHz, $CDCl_3$, 298 K) spectrum of ***c*-P12**$_{t\text{-}Bu}$ in absence of pyridine.

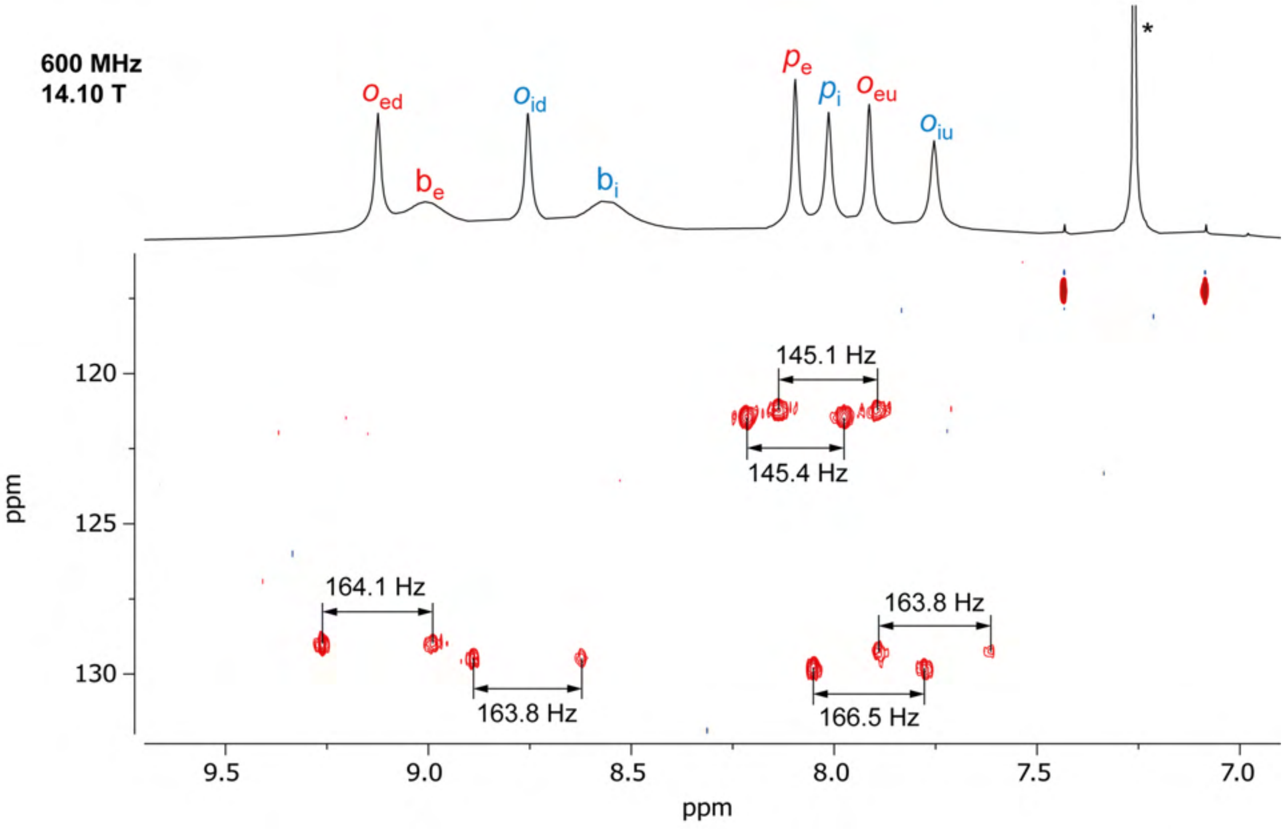

Figure S55. ^{1}H-^{13}C coupled HSQC (600 MHz, $CDCl_3$, 298 K) spectrum of ***c*-P12**$_{t\text{-}Bu}$ in absence of pyridine.

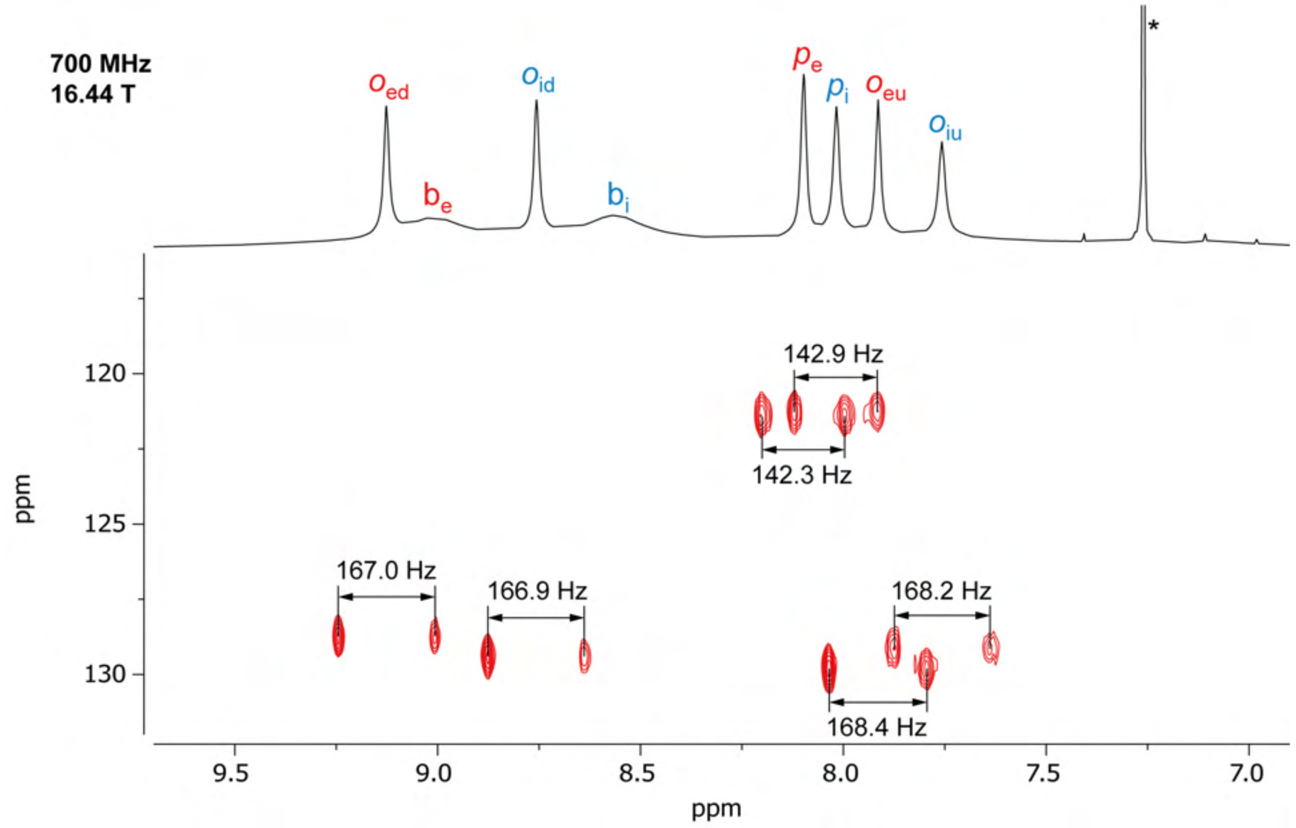

Figure S56. ^{1}H-^{13}C coupled HSQC (700 MHz, $CDCl_3$, 298 K) spectrum of ***c*-P12**$_{t\text{-}Bu}$ in absence of pyridine.

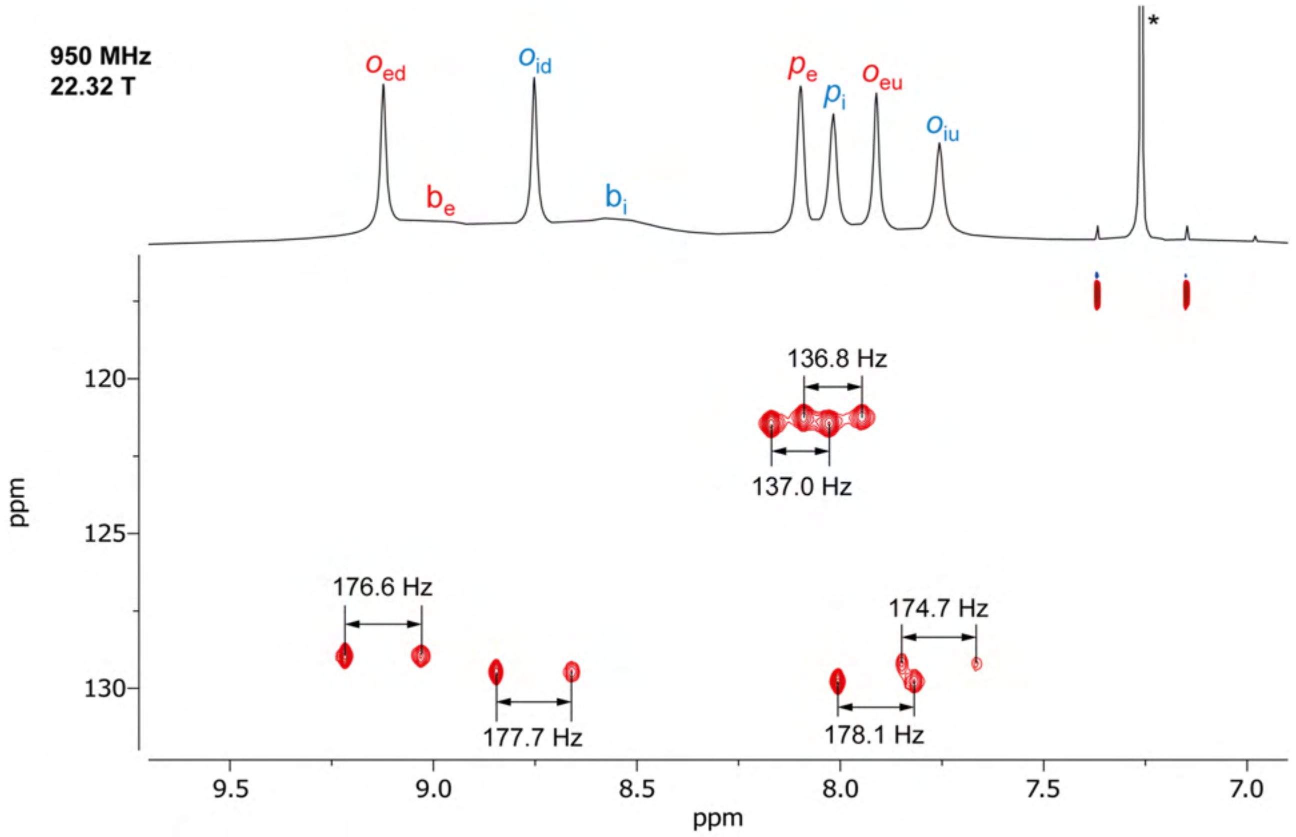

Figure S57. ^{1}H-^{13}C coupled HSQC (950 MHz, $CDCl_3$, 298 K) spectrum of ***c*-P12**$_{t\text{-}Bu}$ in absence of pyridine.

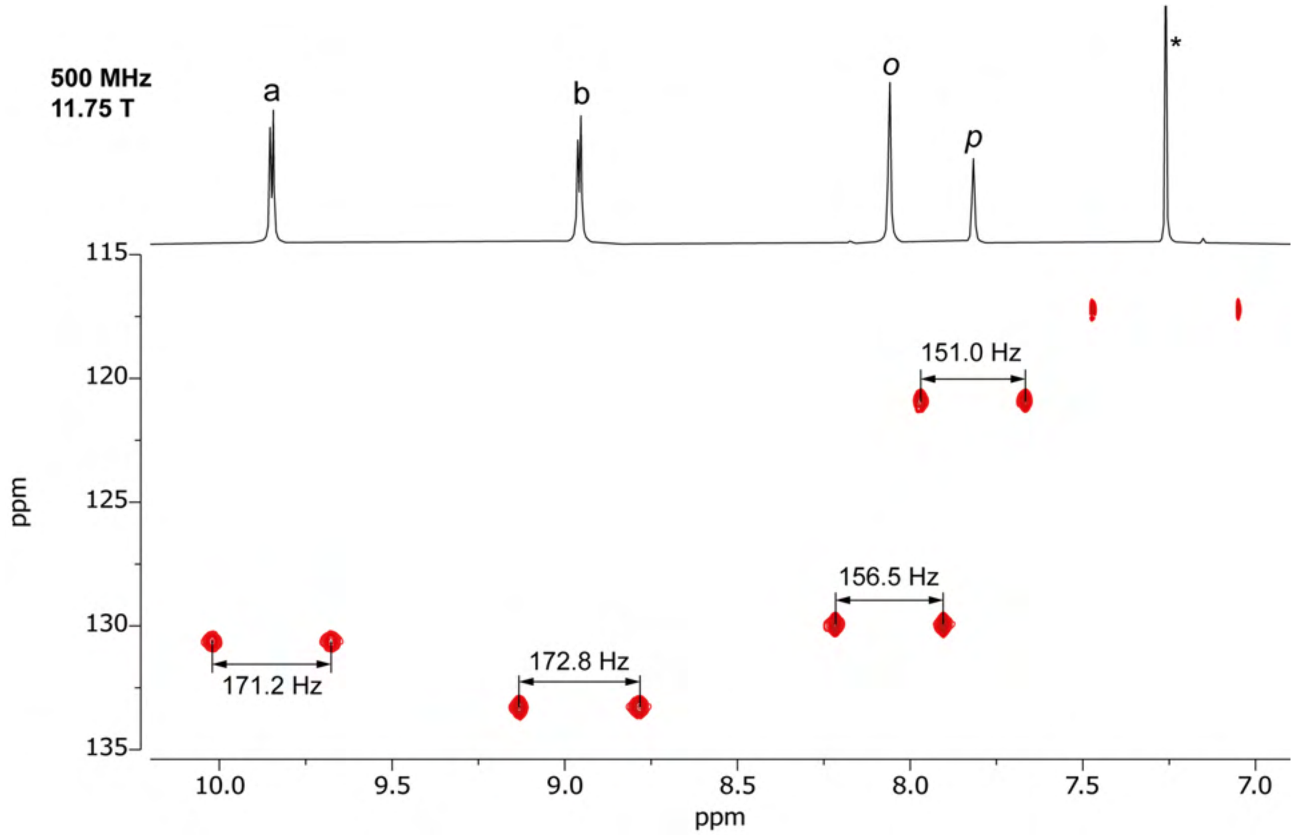

Figure S58. ^{1}H-^{13}C coupled HSQC (500 MHz, $CDCl_3$ + 1% pyridine-d_5, 298 K) spectrum of ***c*-P12$_{t\text{-Bu}}$**.

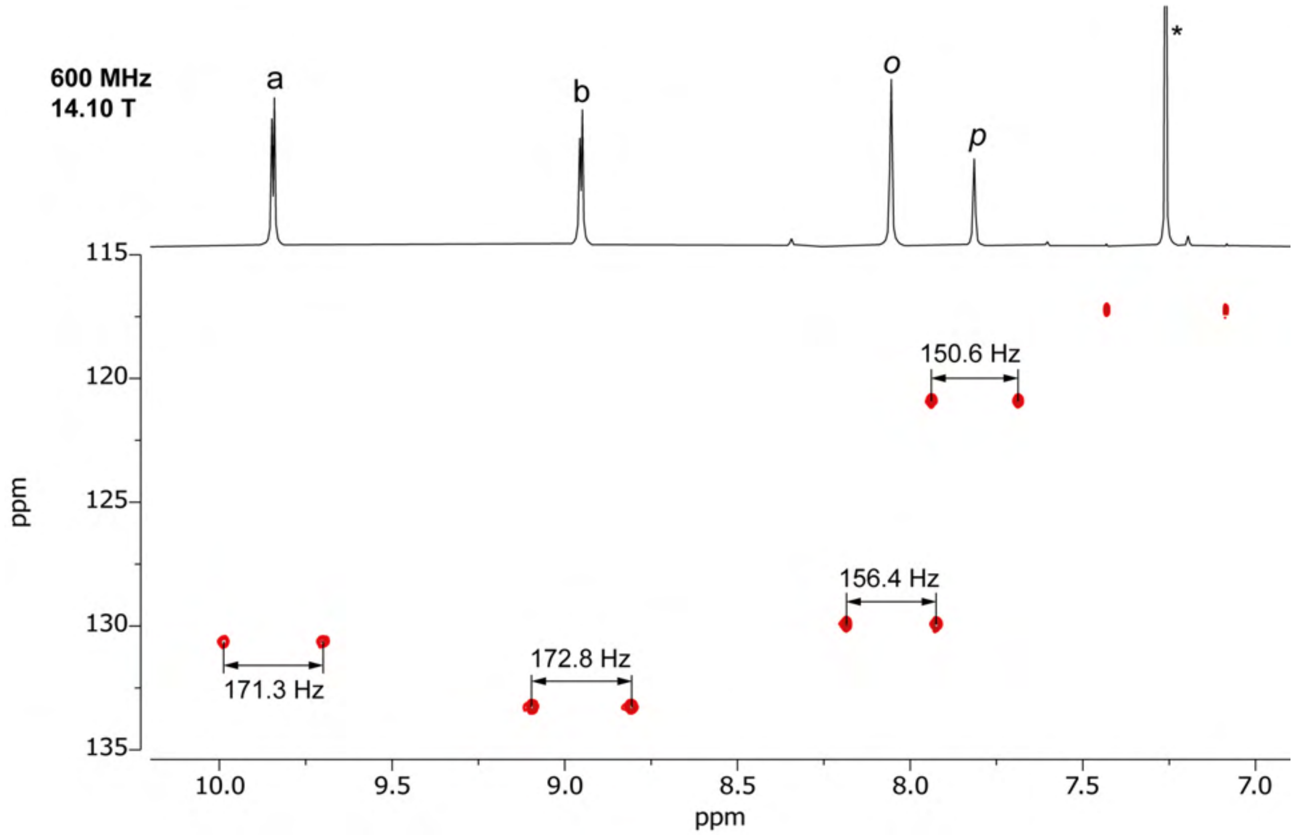

Figure S59. ^{1}H-^{13}C coupled HSQC (600 MHz, $CDCl_3$ + 1% pyridine-d_5, 298 K) spectrum of ***c*-P12$_{t\text{-Bu}}$**.

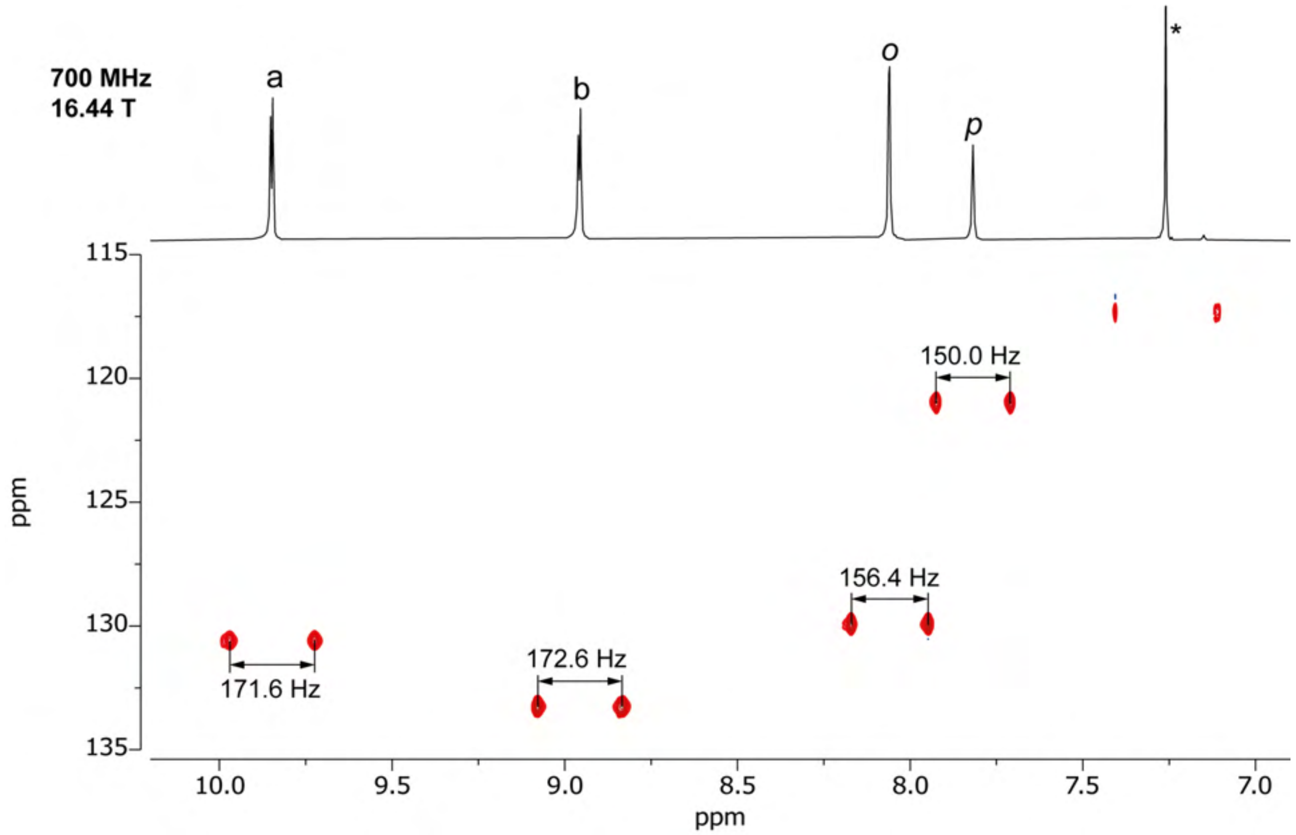

Figure S60. ^{1}H-^{13}C coupled HSQC (700 MHz, $CDCl_3$ + 1% pyridine-*d*$_5$, 298 K) spectrum of ***c*-P12$_{t\text{-Bu}}$**.

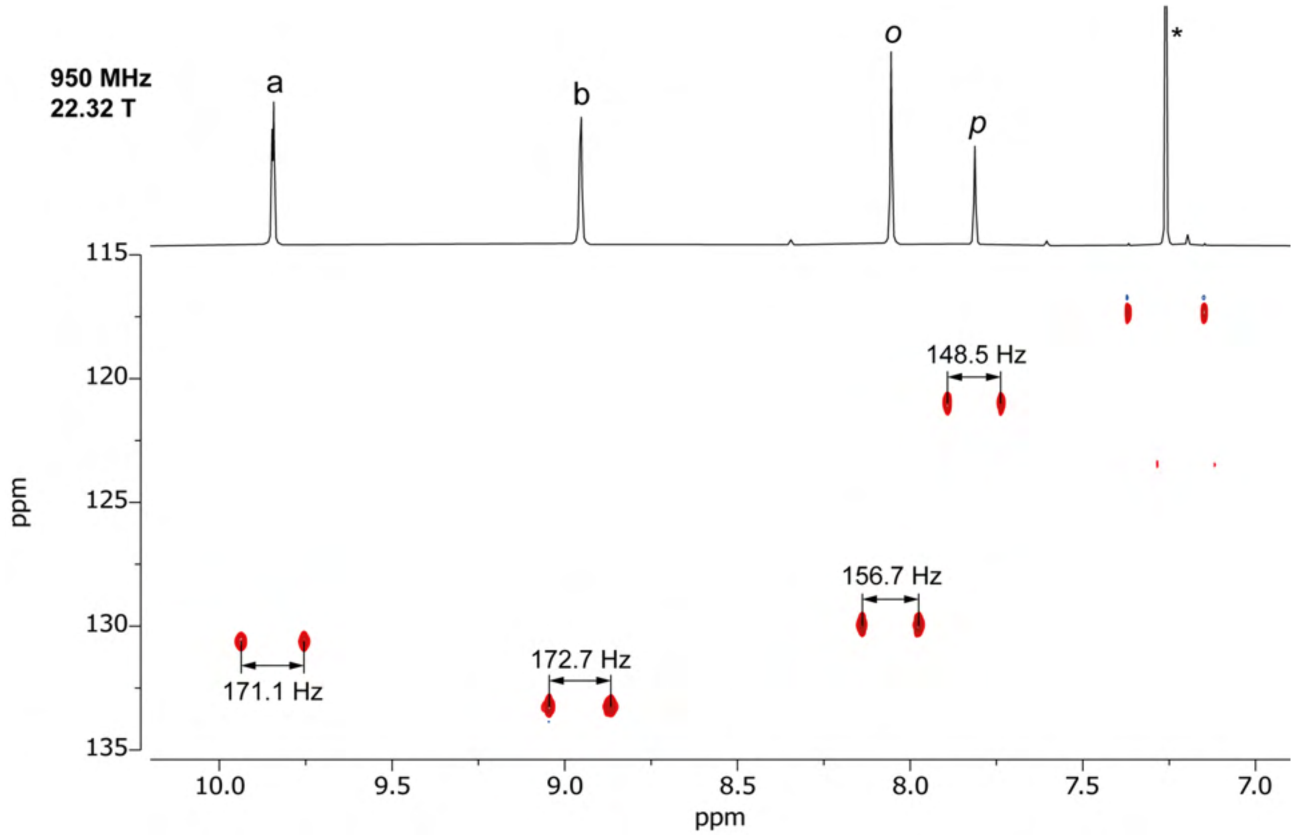

Figure S61. ^{1}H-^{13}C coupled HSQC (950 MHz, $CDCl_3$ + 1% pyridine-*d*$_5$, 298 K) spectrum of ***c*-P12$_{t\text{-Bu}}$**.

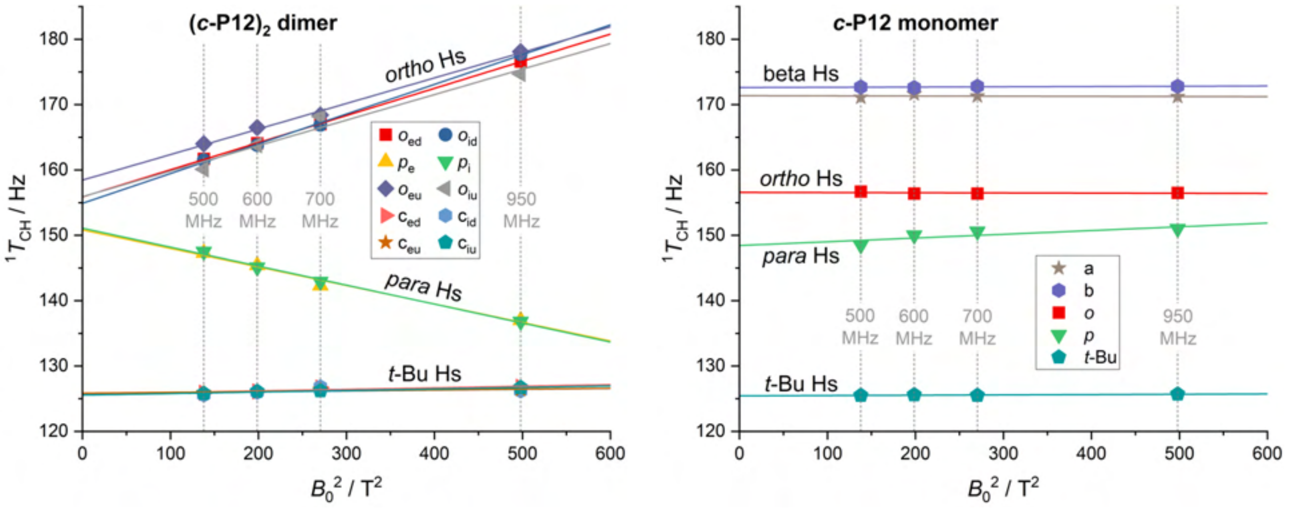

Figure S62. Magnetic field dependence of the observed ^{1}H–^{13}C total splittings ($^1T_{CH}$) in the dimer (left) and monomer of ***c*-P12$_{t\text{-Bu}}$**. Lines are linear best-fits to the $^1T_{CH}$ datapoints at different field strengths. For each proton, the linear best fit was extrapolated to $B_0^2 = 0$ T to obtain the field-independent scalar coupling ($^1J_{CH}$) as the *y*-intercept. Subsequently, the dipolar coupling ($^1D_{CH}$) was determined as $^1D_{CH} = {}^1T_{CH} - {}^1J_{CH}$. The corresponding values are listed in Tables S13.1 and S13.2 below.

Table S8. Summary of experimentally observed ^{1}H-^{13}C total splittings ($^1T_{CH}$), scalar couplings ($^1J_{CH}$), and residual dipolar couplings ($^1D_{CH}$) for **(*c*-P12$_{t\text{-Bu}}$)$_2$**.

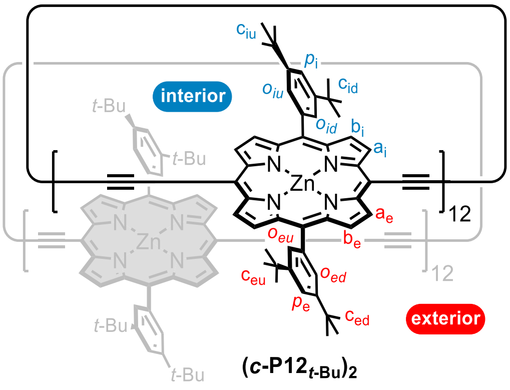

No.	Assign.	δ_H (ppm)	δ_C (ppm)	$^1T_{CH}$ 500 MHz (Hz)	$^1T_{CH}$ 600 MHz (Hz)	$^1T_{CH}$ 700 MHz (Hz)	$^1T_{CH}$ 950 MHz (Hz)	$^1J_{CH}$ (Hz)	$^1D_{CH}$ 950 MHz (Hz)	$^1D_{CH,Calc}$ 950 MHz (Hz)
1	o_{ed}	9.12	129.0	161.7	164.1	167.0	176.6	155.9	+20.7	+19.4
2	b_e	9.00	-	-	-	-	-	-	-	-
3	o_{id}	8.75	129.6	161.5	163.8	166.9	177.7	154.9	+22.8	+20.0
4	b_i	8.56	-	-	-	-	-	-	-	-
5	p_e	8.09	121.6	147.3	145.4	142.3	137.0	149.2	–13.8	–16.1
6	p_i	8.01	121.4	147.5	145.1	142.9	136.8	149.2	–14.3	–16.1
7	o_{eu}	7.91	130.0	164.0	166.5	168.4	178.1	158.4	+19.7	+19.3
8	o_{iu}	7.75	129.2	160.1	163.8	168.2	174.7	155.9	+18.8	+20.0
9	c_{ed}	2.01	32.67	125.9	126.1	126.7	126.8	125.7	+1.1	-
10	c_{id}	1.82	32.65	125.6	126.0	126.8	126.2	125.8	+0.4	-
11	c_{eu}	1.62	32.0	125.9	126.1	126.4	126.4	125.9	+0.5	-
12	c_{iu}	1.59	32.1	125.8	126.1	126.2	126.7	125.6	+1.1	-

Field strengths: 22.32 T (950 MHz), 16.44 T (700 MHz), 14.10 T (600 MHz), and 11.75 T (500 MHz). 1D traces of the F2 (^{1}H) dimension from ^{13}C-coupled HSQC spectra were used to record the total splittings ($^1T_{CH}$). $^1D_{CH,Calc}$ values from the programme PALES using a geometry with all porphyrin units coplanar with the nanoring plane and with aryl sidechains at 90° relative to the plane of the porphyrin units.

Table S9. Summary of experimentally observed ^{1}H-^{13}C total splittings ($^1T_{CH}$), scalar couplings ($^1J_{CH}$), and residual dipolar couplings ($^1D_{CH}$) for ***c*-P12$_{t\text{-Bu}}$** in the presence of pyridine.

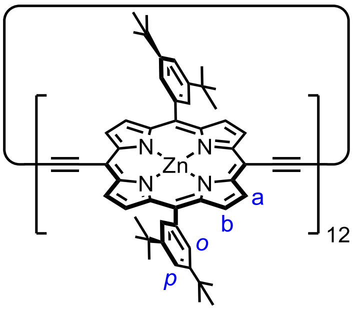

***c*-P12$_{t\text{-Bu}}$**

No.	Assign.	δ_H (ppm)	δ_C (ppm)	$^1T_{CH}$ 500 MHz (Hz)	$^1T_{CH}$ 600 MHz (Hz)	$^1T_{CH}$ 700 MHz (Hz)	$^1T_{CH}$ 950 MHz (Hz)	$^1J_{CH}$ (Hz)	$^1D_{CH}$ 950 MHz (Hz)	$^1D_{CH,Calc}$ 950 MHz (Hz)
1	a	9.84	130.6	171.2	171.3	171.6	171.1	171.4	–0.3	-
2	b	8.95	133.3	172.8	172.8	172.6	172.7	172.6	+0.1	-
3	*o*	8.06	130.0	156.5	156.4	156.4	156.7	157.0	–0.3	-
4	*p*	7.81	120.9	151.0	150.6	150.0	148.5	148.4	+0.1	-
5	*t*-Bu	1.56	-	125.7	125.5	125.6	125.5	125.4	+0.1	-

Field strengths: 22.32 T (950 MHz), 16.44 T (700 MHz), 14.10 T (600 MHz), and 11.75 T (500 MHz). 1D traces of the F2 (^{1}H) dimension from ^{13}C-coupled HSQC spectra were used to record the total splittings ($^1T_{CH}$).

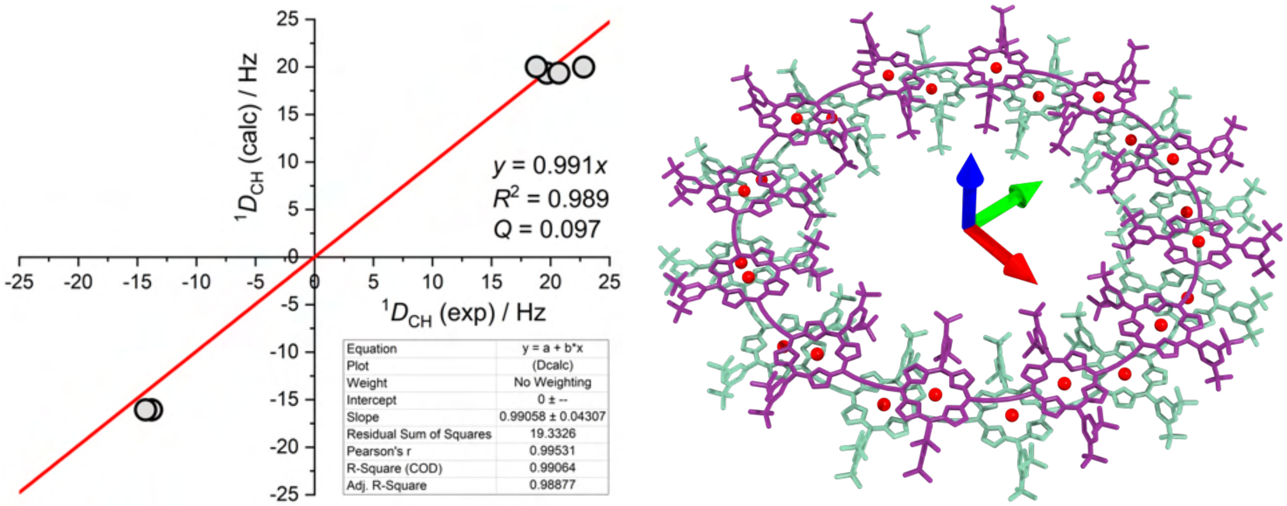

Figure S63. Left: linear fit of observed and calculated residual dipolar coupling values for the ***c*-P12$_{t\text{-Bu}}$** dimer. Right: PALES input structure, oriented with the molecular alignment tensor along the *z*-axis (blue arrow).

Table S10. Screening of dihedral angles. Left: screening of the dihedral angle with the interior dihedral constrained at 88°. Right: screening of the interior dihedral angle with the exterior dihedral constrained at 82°. Dihedral angles of 82° (exterior) and 88° (interior) are derived from an xTB geometry optimization of the ***c*-P12**$_{t\text{-Bu}}$ dimer. Plots and geometries are shown in Figure S13.11 and S13.12.

Interior dihedral = 88°			Exterior dihedral = 82°	
Exterior dihedral / °	*Q*		Interior dihedral / °	*Q*
70	0.1562		70	0.1678
75	0.1195		75	0.1293
80	0.1002		80	0.1079
82	0.0968		85	0.0987
85	0.0933		88	0.0968
90	0.0918		90	0.0963
95	0.0931		95	0.0986
100	0.0995		100	0.1077
105	0.1175		105	0.1288
110	0.1529		110	0.167

Table S11. Screening of the angle between porphyrins and the ring plane, while dihedral angles of the exterior sidechains were constrained. Plots and geometries are shown in Figure S13.13 and S13.14.

**Angle between porphyrins and ring plane** Exterior = 82° and interior = 88° (†) / °	***Q***		**Angle between porphyrins and ring plane** Exterior = interior = 90° (†) / °	***Q***
-10	0.1264		-10	0.1224
-9	0.1151		-9	0.1108
-8	0.1068		-8	0.1023
-7	0.1009		-7	0.0963
-6	0.0975		-6	0.0927
-5	0.0958		-5	0.0905
-4	0.0956		-4	0.0902
-3	0.0957		-3	0.0904
-2	0.0958		-2	0.0904
-1	0.0964		-1	0.0909
0	0.0968		0	0.0913
1	0.0964		1	0.0909
2	0.0962		2	0.0908
3	0.0957		3	0.0904
4	0.0950		4	0.0902
5	0.0958		5	0.0909
6	0.0974		6	0.0926
7	0.1006		7	0.0961
8	0.1063		8	0.1022
9	0.1144		9	0.1108
10	0.1256		10	0.1222

(†) Exterior and dihedral angles for the sidechains are fixed at these values.

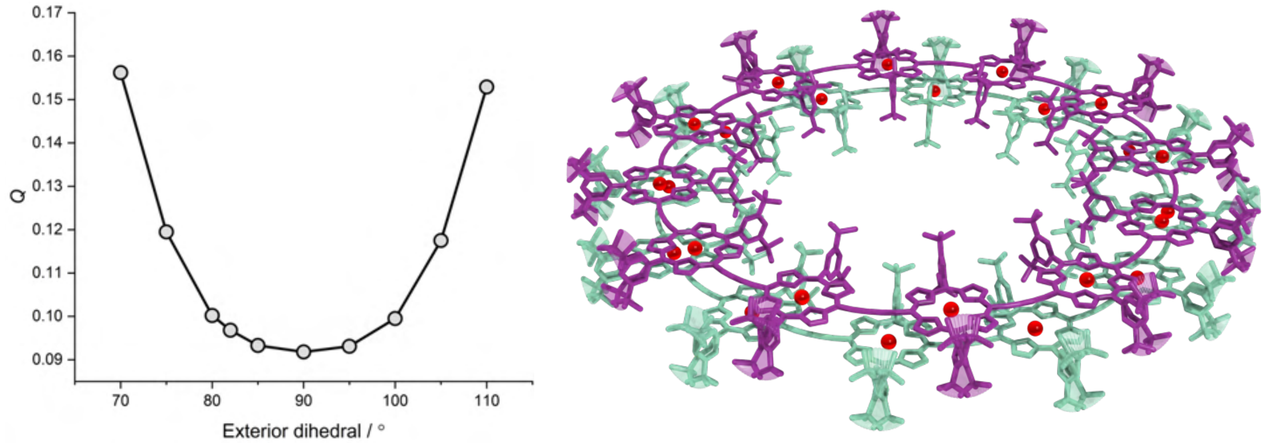

Figure S64. Left: plot of *Q*-values versus exterior dihedral angles with fixed interior dihedral (88°). Right: superposition of all ***c*-P12**$_{t\text{-Bu}}$ dimer geometries. Lowest *Q* of 0.092 obtained for exterior dihedral of 90°, while a marginally higher *Q* of 0.097 was obtained for exterior dihedral of 88°.

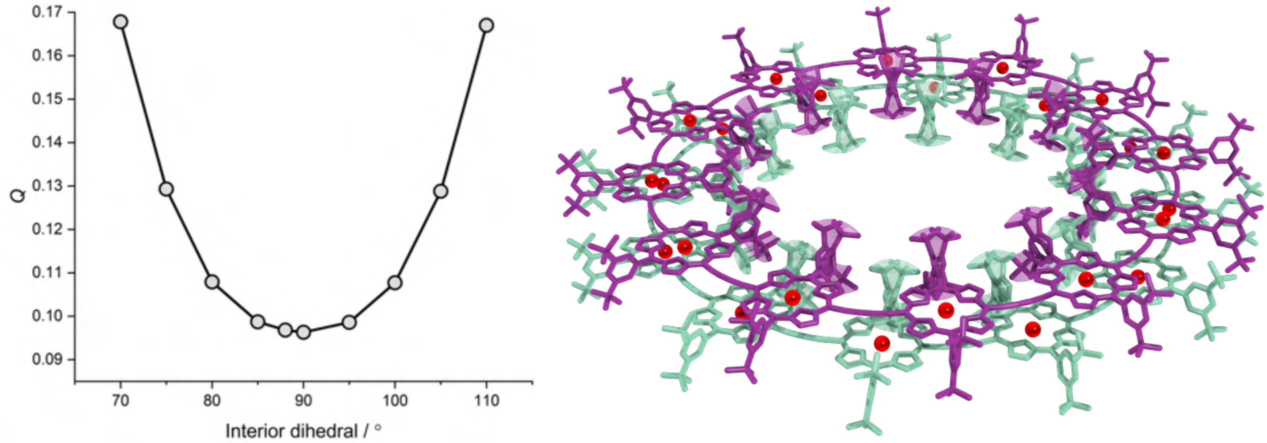

Figure S65. Left: plot of *Q*-values versus interior dihedral angles with fixed exterior dihedral (82°). Right: superposition of all ***c*-P12**$_{t\text{-Bu}}$ dimer geometries. Lowest *Q* of 0.097 and 0.096 was obtained for interior dihedrals of 88° or 90°, respectively.

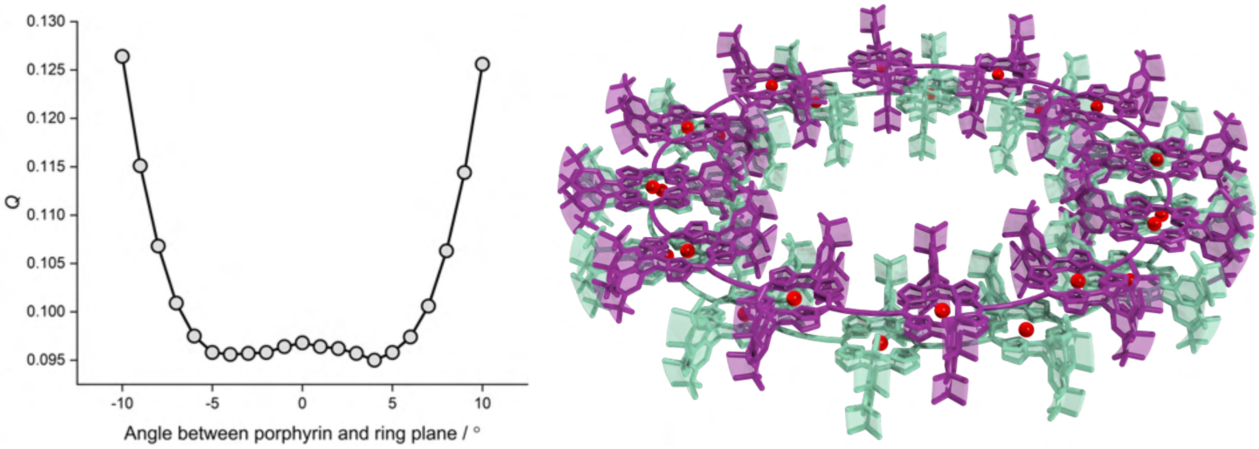

Figure S66. Left: plot of Q-values versus angle between porphyrins and ring plane. Exterior and interior dihedrals fixed at 82° and 88°, respectively. Right: superposition of all ***c*-P12$_{t\text{-Bu}}$** dimer geometries. Two shallow minima are seen on opposite sides of 0°, i.e., at –4° (Q = 0.096) and +4° (Q = 0.095). This implies that the ring dimer exhibits some flexibility which enables porphyrin units to rotate slightly out of the plane of the ring.

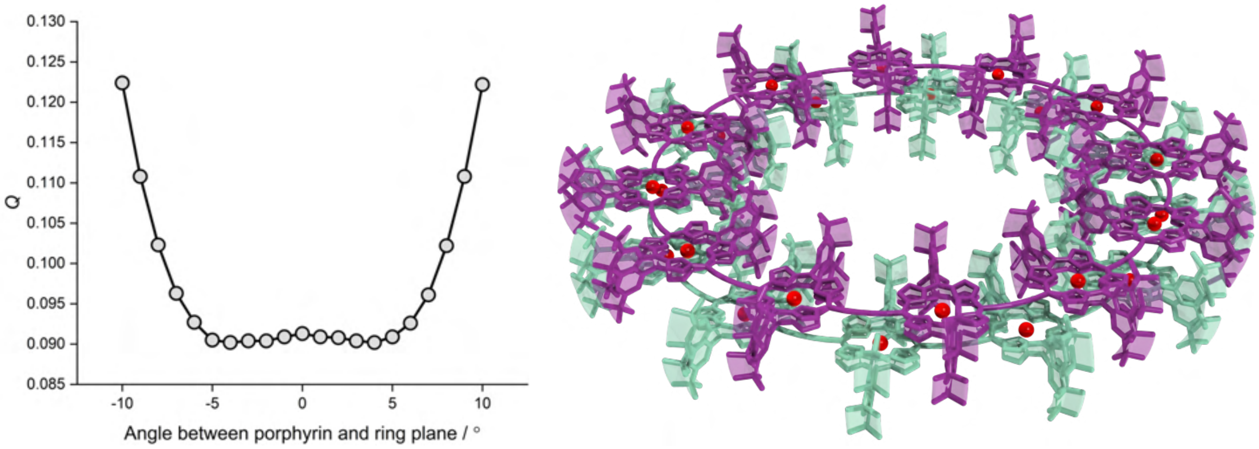

Figure S67. Left: plot of Q-values versus angle between porphyrins and ring plane. Exterior and interior dihedrals fixed at 90°. Right: superposition of all ***c*-P12$_{t\text{-Bu}}$** dimer geometries. Two shallow minima are seen on opposite sides of 0°, i.e., at –4° (Q = 0.090) and +4° (Q = 0.090). This implies that the ring dimer exhibits some flexibility which enables porphyrin units to rotate slightly out of the plane of the ring.

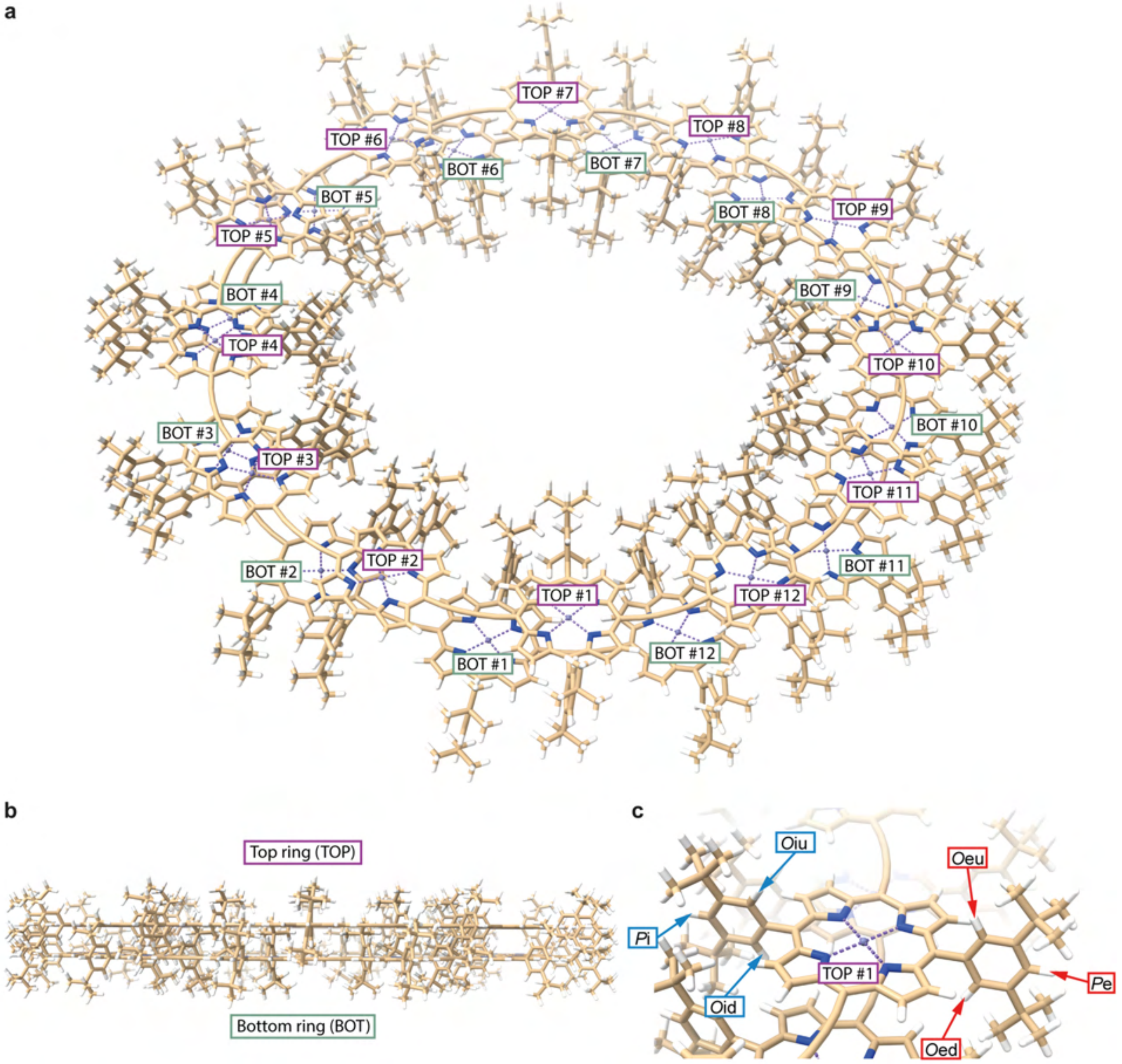

Figure S68. PALES input geometry of the ***c*-P12**$_{t\text{-Bu}}$ dimer. Labels indicate naming of porphyrin units, carbons, and protons in the PALES input geometry file.

The magnetic susceptibility anisotropy of **(*c*-P12*t*-Bu)2** was estimated starting from equation S10, stating the relationship between the dipolar coupling ($^1D_{CH}$) and the magnetic field squared (B_0^2):

$$^1D_{CH} = \frac{\gamma_C \gamma_H h}{2\pi^2 r_{C-H}^3}\left(\frac{3}{2}cos^2\theta - \frac{1}{2}\right)\frac{\Delta\chi}{15kT}B_0^2 \qquad \text{(Eqn. S10)}$$

where γ_C and γ_H are the gyromagnetic ratios of the ^{13}C and ^{1}H nuclei, h is the Planck constant, $r_{C–H}$ is the bond length between the two nuclei, θ is the angle between the ^{13}C–^{1}H inter-nuclear vector and the molecular z-axis (i.e., the principal axis) which is perpendicular to the plane of the porphyrin nanorings in **(*c*-P12*t*-Bu)2**, k is the Boltzmann constant, T is temperature, and $\Delta\chi$ is the magnetic susceptibility anisotropy.[18]

A linear fit of $^1D_{CH}$ versus B_0^2 (Figure S69), provides the slope ($^1D_{CH}$ / B_0^2), which can be used to determine $\Delta\chi$, as given by the following rearrangement of Eqn S10:

$$\Delta\chi = \frac{^1D_{CH}}{B_0^2}\frac{15kT2\pi^2 r_{C-H}^3}{\gamma_C\gamma_H h\left(\frac{3}{2}cos^2\theta - \frac{1}{2}\right)} \qquad \text{(Eqn. S11)}$$

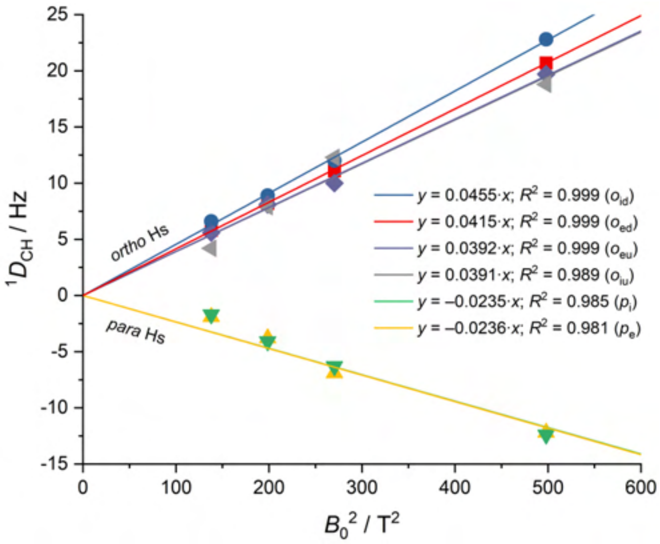

Figure S69. Linear fit of experimental dipolar coupling $^1D_{CH}$ of **(*c*-P12*t*-Bu)2** versus the external magnetic field squared (B_0^2), yielding slope-values ($^1D_{CH}$ / B_0^2) used to estimate the magnetic susceptibility anisotropy.

Table S12. Tabulated values for the slopes in Figure S69 and calculated values of $\Delta\chi$ using the average slope values for the *ortho* and *para* protons.

Proton	Slope ($^1D_{CH}$ / B_0^2) / Hz·T^{-2}	$\Delta\chi$ / cm^3
o_{id}	0.0455	-
o_{ed}	0.0415	-
o_{eu}	0.0392	-
o_{iu}	0.0391	-
Average *ortho*	0.0413	–8.98 × 10^{-27}
p_i	–0.0235	-
p_e	–0.0236	-
Average *para*	–0.0236	–6.41 × 10^{-27}

Values used for the calculation of $\Delta\chi$ using equation S11: k = 1.381·10^{-23} m^2·kg·s^{-2}·K; T = 298 K; $r_{C–H}$ = 1.1·10^{-8} cm (obtained from the proposed model); γ_H = 267.522·10^6 rad·s^{-1}·T^{-1}; γ_C = 67.283·10^6 rad·s^{-1}·T^{-1}; h = 6.626·10^{-34} m^2·kg·s^{-1}; θ = 30° (C–H$_{ortho}$), 90° (C–H$_{para}$).

The estimated $\Delta\chi$ of **(*c*-P12*t*-Bu)2** was calculated, as the average between the value obtained from *ortho* and *para* protons, to be –7.7 × 10^{-27} cm^3, corresponding to that of 7.9 porphyrin monomers ($\Delta\chi$ = –9.8 × 10^{-28} cm^3), or in other words, $\Delta\chi$ of a porphyrin monomer is preserved by 33% in **(*c*-P12*t*-Bu)2**.

Section 14. Quantum Chemical Calculations

14.1. Structure Optimizations

Methodology. Structures of aggregates were optimized by sampling conformations using the CREST program[19] at the GF2-xTB level of theory implemented in the xTB program[20]. The lowest energy conformer was subsequently minimized at the PBE0+D3BJ/def2-SVP+def2-TZVP(Zn) level of theory as implemented in ORCA 5.0.3[21] and with the AMBER force field[22] with additional parameters developed for Zn-porphyrin[23] using GROMACS (v. 2019.2 double precision)[24].

Structures of ring aggregates were minimized using the xTB program using the default settings for minimization and convergence criteria. We used artificially generated, fully symmetric structures with 0° shift as starting points for the optimizations. Analytical and numerical frequency calculations to test the minimum were attempted but not successful due to the large size of the system.

Results. Bimolecular stacks of porphyrin monomers and nanorings with phenyl, methoxy and *t*-butyl sidechains were optimized. The distance *d* and the shift *s* offset between the porphyrin nanorings in a dimeric stack as defined in Figure S70 are listed in Table S13. Compared to DFT optimized structures the xTB structures tend to have a smaller distance between porphyrins but a comparable shift. MM optimized structures in comparison have a similar distance to DFT optimized structures but a larger shift. This is probably caused by a steeper dihedral angle between the porphyrin and the phenyl side chain which pushes the porphyrins further apart, as can be seen in Figure S71.

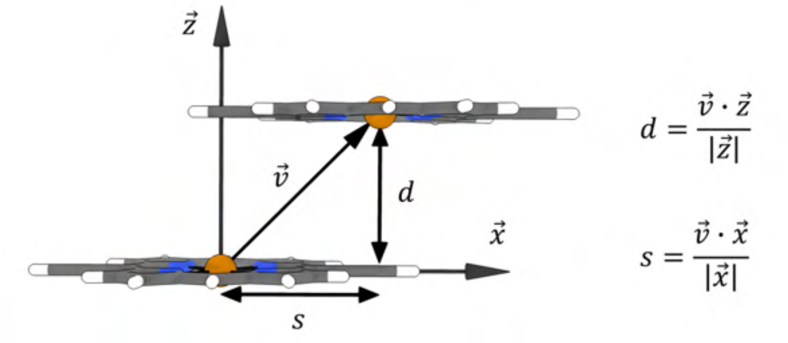

Figure S70. Definition of porphyrin distance *d* and porphyrin shift *s* using the vector *v* connecting the centers of mass of the porphyrins and the vector *p* perpendicular to the plane of the porphyrin.

Table S13. Structural parameters of porphyrin monomer stacks.

Structure		Distance *d* / Å	Shift *s* / Å
	xTB	3.18	3.02
**(*l*-P1$_{Phe}$)$_2$**	DFT	3.37	2.99
	MM	3.51	2.54
	xTB	3.15	1.39
**(*l*-P1$_{OMe}$)$_2$**	DFT	3.36	1.31
	MM	3.39	2.74
	xTB	3.13	3.29
**(*l*-P1$_{t\text{-}Bu}$)$_2$**	DFT	3.39	3.35
	MM	3.32	4.46

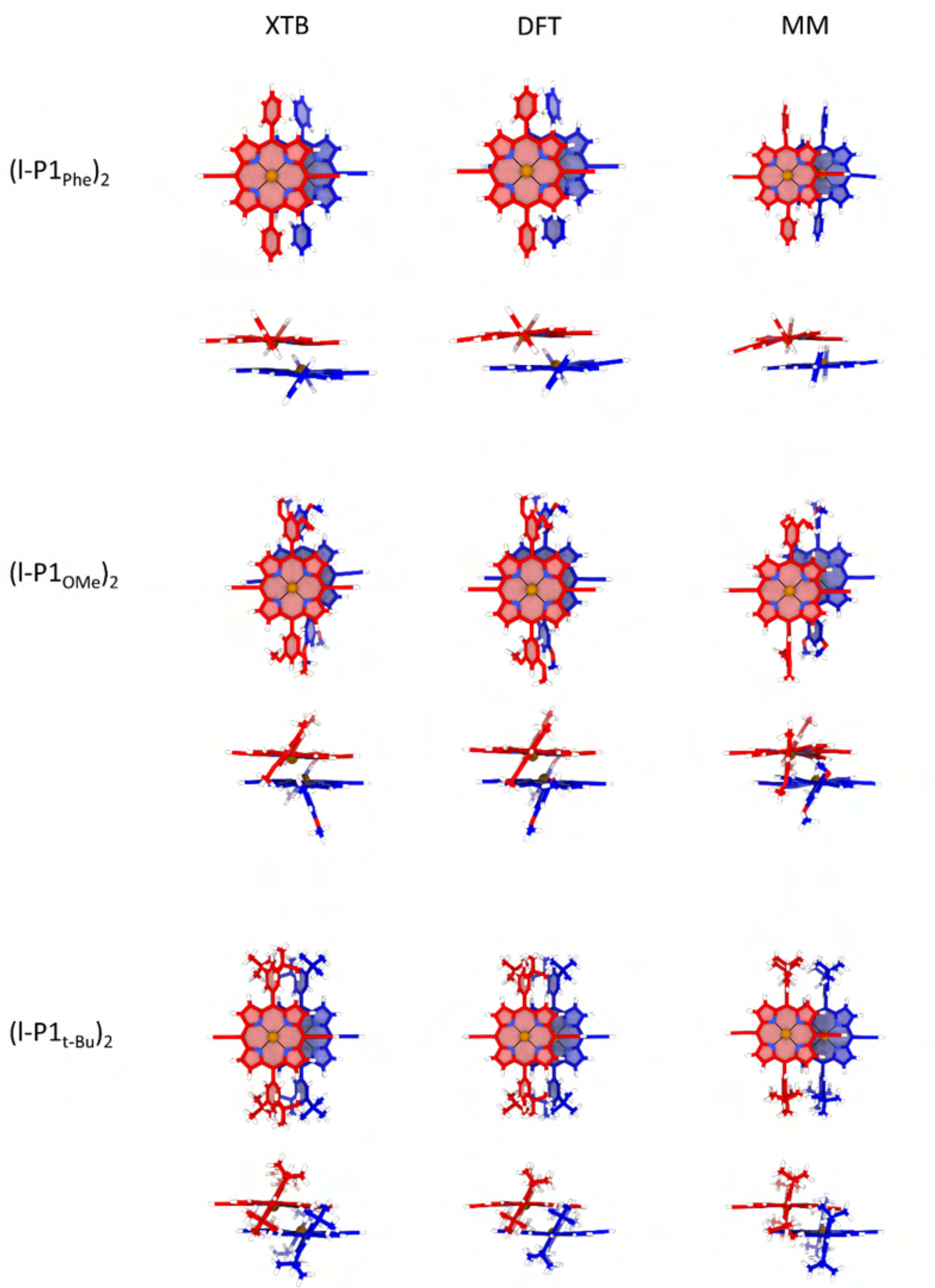

Figure S71. Structures of porphyrin monomer stacks.

Structures of nanoring aggregates **(*c*-P8$_{OMe}$)$_2$** and **(*c*-P12$_{t\text{-}Bu}$)$_2$**, as well as their simpler analogues with phenyl sidechains, **(*c*-P8$_{Phe}$)$_2$** and **(*c*-P12$_{Phe}$)$_2$**, have been optimized. The octyloxy sidechain was truncated as a methoxy group. The optimized structures are shown in Figure S72 and Table S14 lists the average distances and shifts between the porphyrin units as defined for the monomeric systems, and the rotational shift between rings in degrees. The average distance between stacked porphyrins is comparable to the optimized structures of the monomer stacks. The shift is larger in the ring aggregates (i.e., less well stacked on top of each other) due to the larger clash between interior sidechains caused by the curvature of the rings.

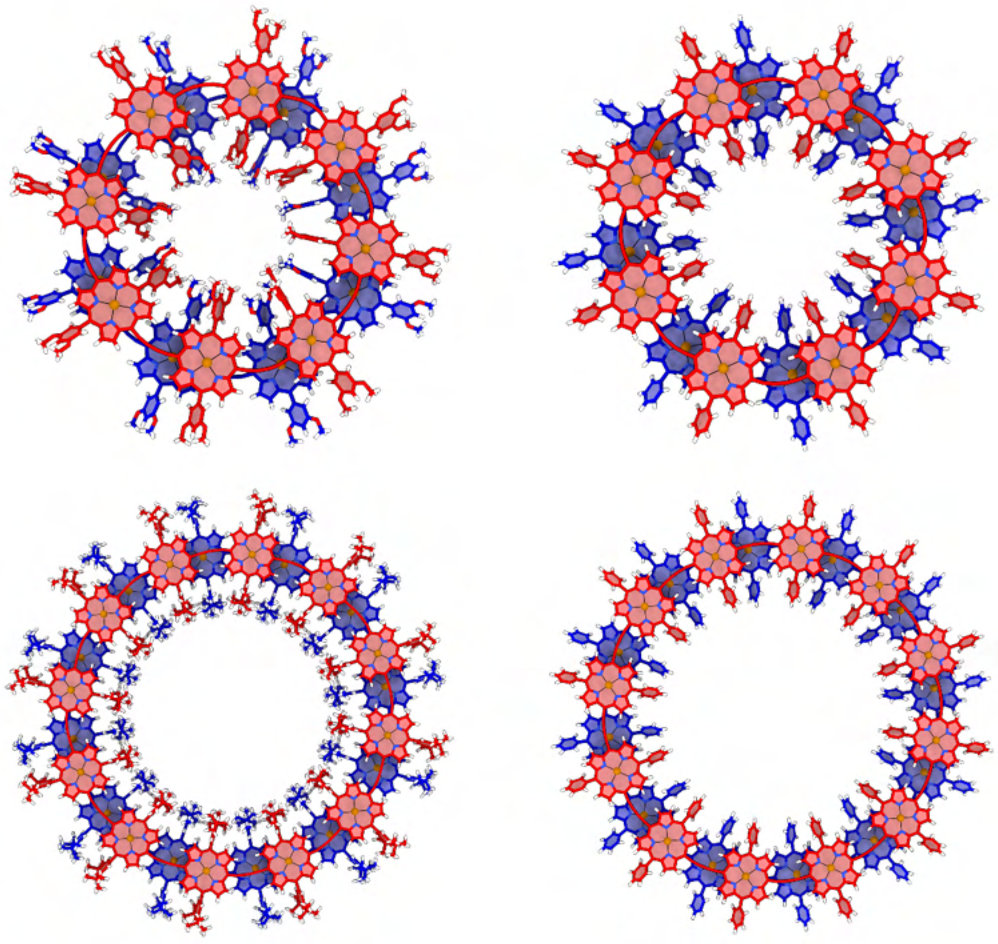

Figure S72. Structures of ring aggregates. Top: **(*c*-P8$_{OMe}$)$_2$** (left) and **(*c*-P8$_{Phe}$)$_2$** (right). Bottom: **(*c*-P12$_{t\text{-}Bu}$)$_2$** (left) and **(*c*-P12$_{Phe}$)$_2$** (right).

Table S14. Structural parameters of ring aggregates from xTB optimized structures.

Structure	Sidechain	Average Distance d / Å	Average Shift s / Å	Rotation / °
**(*c*-P8$_{OMe}$)$_2$**	Methoxy	2.616	4.454	7.86
**(*c*-P8$_{Phe}$)$_2$**	Phenyl	2.641	4.893	5.76
**(*c*-P12$_{t\text{-}Bu}$)$_2$**	*t*-Butyl	2.634	5.441	2.76
**(*c*-P12$_{Phe}$)$_2$**	Phenyl	2.579	5.256	3.18

14.2. Planarization energy

Methodology. Energies were calculated at the PBE0+D3BJ/def2-SVP+def2-TZVP(Zn) level of theory as implemented in ORCA 5.0.3[21]. A minimum structure and a planarized structure were obtained for each ring size using default optimisation settings. In the planarized structure all atoms were restricted to the $z = 0$ plane. While this is not the optimal planarized structure as it inhibits the normal buckling of porphyrins, it is difficult to define an internal coordinate which allows some level of distortion and still gives the desired planarization. Therefore, this approach was chosen as a compromise and should still provide insights into the energy trends of planarization.

Results. There is an energy penalty to rotating all porphyrins into the plane of the ring. The energy differences between optimized and planarized structures are listed in Table S15 and plotted in Figure S73. They show a decrease in the energy penalty with increasing ring size as the strain in the systems is reduced and there is less steric clash between neighbouring porphyrins. This leads to an easier aggregation of planarized porphyrin rings of larger systems (along with a larger number of positive π–π stacking interactions).

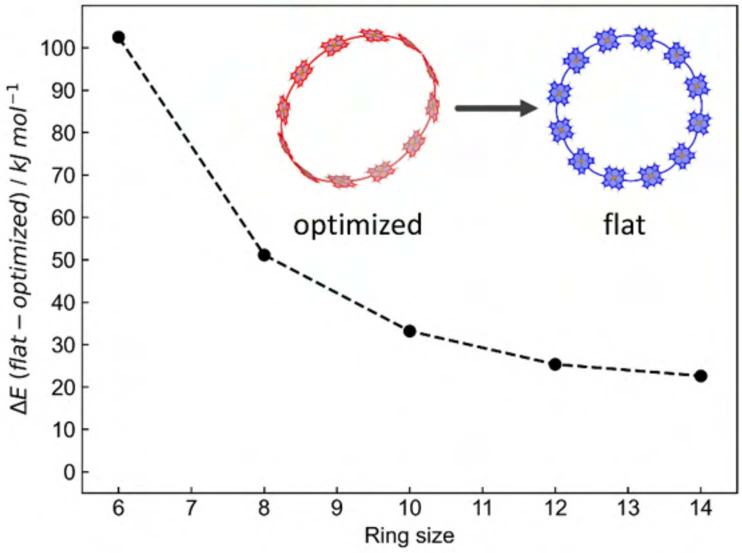

Figure S73. Variation in planarization energy for varying ring sizes. Inset shows optimized and planarized structures for ***c*-P12**.

Table S15. Energies of geometry optimized structures. *ΔE* is the energy difference between optimized (opt) and planar (flat) structures.

Structure	*E*(opt) / a.u.	*E*(flat) / a.u.	*ΔE* / a.u.	*ΔE* / kJ mol^{-1}
***c*-P6**	-17500	-17500	0.03906	102.5
***c*-P8**	-23333.4	-23333.4	0.01949	51.2
***c*-P10**	-29166.7	-29166.7	0.01264	33.2
***c*-P12**	-35000.1	-35000.1	0.00965	25.3
***c*-P14**	-40833.4	-40833.4	0.00862	22.6

14.3. Ring-in-ring rotation in (c-P12$_{t\text{-Bu}}$)$_2$ and estimation of its barrier

Methodology. An initial transition state (TS) and energy profile were obtained via the nudged-elastic band (NEB) method[25] implemented in ORCA 5.0.3[21] at the GF2-xTB level of theory using the ORCA-xTB interface. The start and end points for the NEB calculation were obtained using the xTB program by minimizing a symmetric structure which was offset by one degree to either direction. The TS could not successfully be optimized using the ORCA TSopt keyword as it could not successfully keep track of the correct mode that corresponded to the reaction coordinate. Instead, we used Translation and Rotation Internal Coordinates (TRIC)[26] as implemented in the Pysisyphus program.[27]

In addition, the energy profile of the ring shift was also obtained from Molecular Mechanics calculations using GROMACS (v. 2019.2 double precision),[24] see Section 14.5. for details on parameters. The reaction coordinate was defined by restraining distances between the centers of mass of adjacent porphyrins in different rings using GROMACS' COM pulling functionality giving a total of 24 restraints. The restraint distances were varied between 0.750 nm (symmetric structure) and 0.60 nm/0.90 nm (shifted structure) and chosen so as to maintain the correct aggregate distance d and only vary the shift s. Structures were then optimized with the L-BFGS algorithm with a convergence threshold of 0.2 kJ mol^{-1} nm^{-1}.

Results.

The ring rotation between the two possible π–π stabilized conformers proceeds via a symmetric transition state (see Figure S14.5a). The transition state had a single negative frequency with an unusually low value of -0.1912 cm^{-1} arising from a transition state not involving any covalent bond breaking (Table S16). The semiempirical xTB calculations yielded an energy barrier of 192. kJ mol^{-1} which is considerably higher than the experimental barrier of 51 kJ mol^{-1}. This may be in part due to the fact that convergence was only achieved with loose convergence criteria (maximum force of 1.38×10^{-3} au, mean force of 1.53×10^{-4} au) and also that the calculations were done in vacuum where dispersion forces will have an exaggerated effect.

In contrast, the energy profile obtained from molecular mechanics calculations is in much better agreement with our experimental results, giving an energy barrier of 40.9 kJ mol^{-1}. The energy minima were found at ± 2.7° similar to the minimum obtained from xTB calculations. In addition, the energy profile shows a steep increase in energy as the shift angle goes to more positive or negative values due to the increased clash between porphyrin sidechains (Figure S74).

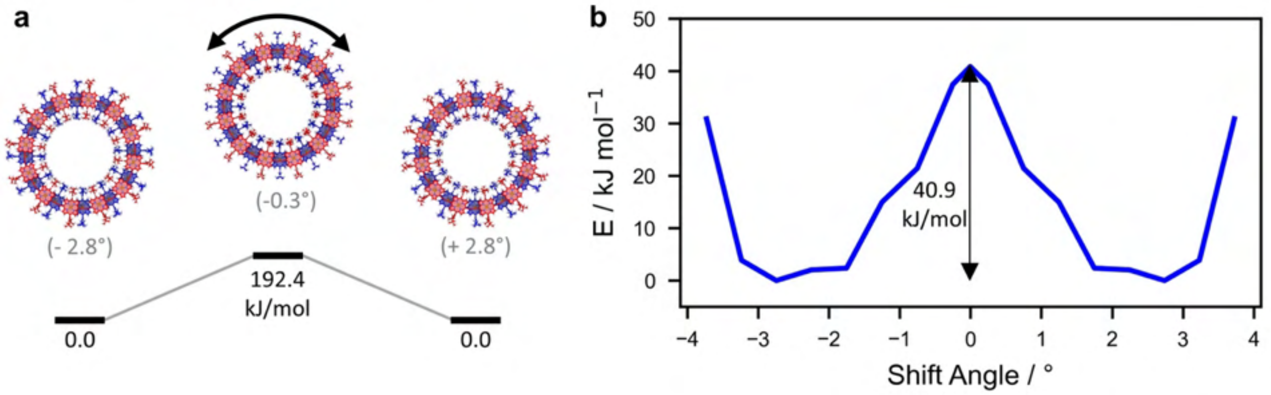

Figure S74. (a) Structures of minima and transition states with their associated energies calculated with xTB. (b) Energy profile of ring shift motion obtained from MM calculations.

Table S16. Energies and negative frequencies calculated for optimized structures and TS.

Structure	Absolute Energy / Eh	Neg. Frequencies / cm^{-1}
Minimum A	-3581.0524	-
Minimum B	-3581.0524	-
TS	-3580.9791	-0.1912

14.4. NMR Chemical Shift Calculations

Methodology. NMR calculations were carried out on geometries of **(*c*-P12$_{t\text{-Bu}}$)$_2$** obtained from the transition state trajectory. Five geometries were selected starting from the lowest energy conformation to the transition state geometry. The selected geometries correspond to a calculated ring rotation of ca. 2.8° (lowest energy conformation), 1.5°, 0.9°, 0.3°, and –0.3° (transition state geometry). Each geometry was dissected into four fragments of trimer aggregates, which were used in the NMR calculations. NMR shielding values were calculated using the GIAO method at the mPW1PW91/6-31G(d,p) level of theory and using the polarizable continuum model (PCM) with chloroform as solvent.[28] The isotropic proton shielding values were extracted for the two central porphyrin units of each trimer aggregate, for all four fragments constituting a geometry, and then averaged. The averaged proton shielding values were converted into their ^{1}H chemical shift values by subtracting them from the calculated shielding value of tetramethylsilane ($\delta_H = \sigma_{TMS} - \sigma_H$) at the same level of theory.

Results.

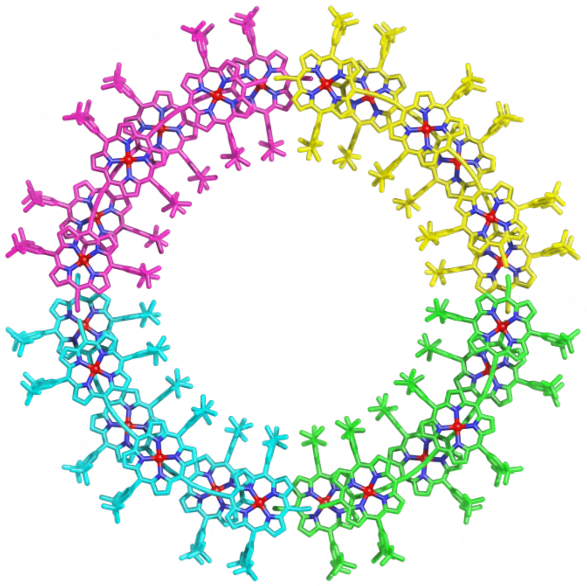

Figure S75. Example of how the lowest energy conformation of **(*c*-P12$_{t\text{-Bu}}$)$_2$** was cut into four fragments of aggregated trimers, each submitted for NMR calculations.

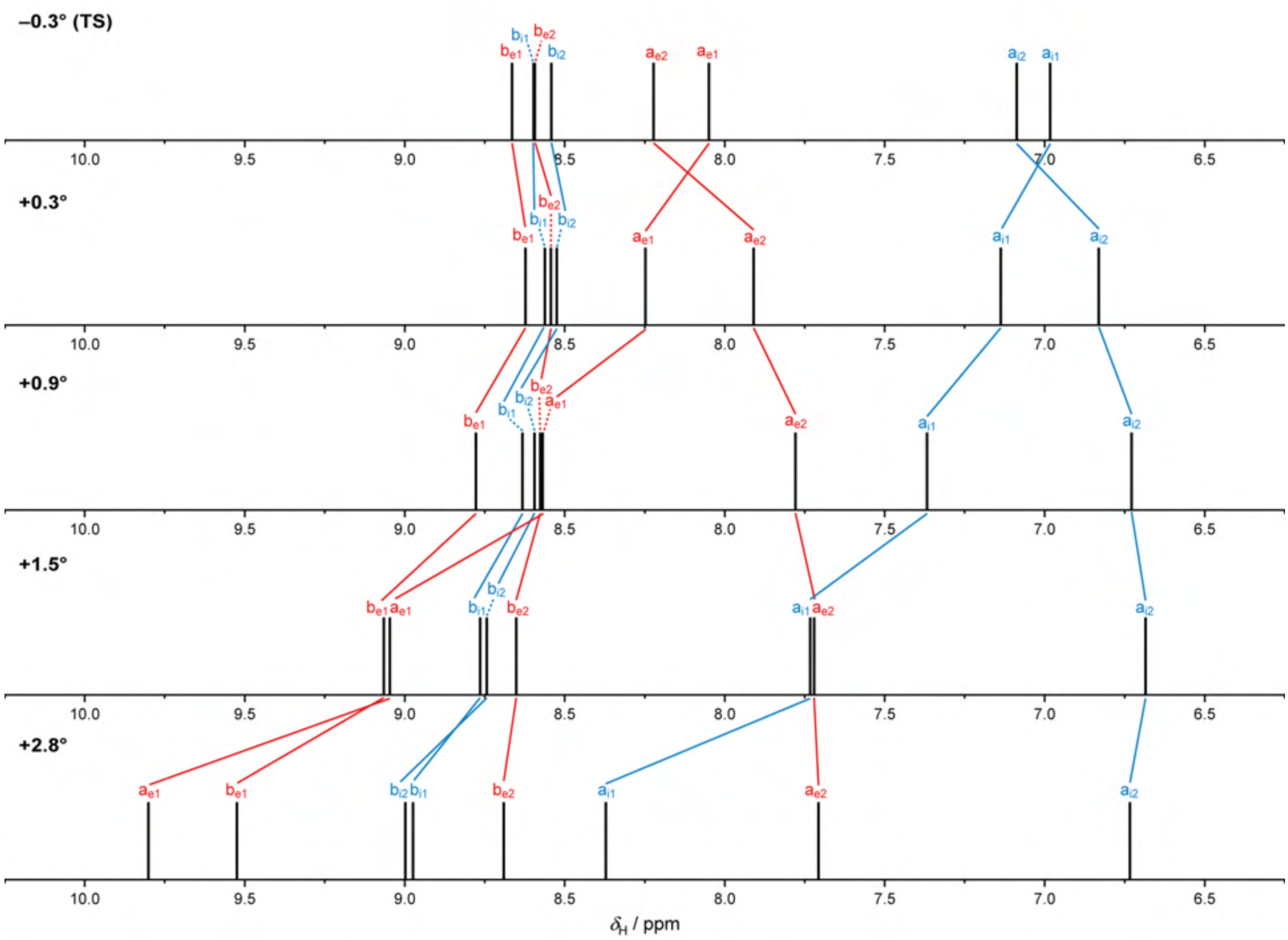

Figure S76. Calculated ^{1}H-NMR spectra for geometries along the transition state trajectory from the transition state geometry (top) to the lowest energy conformation (bottom).

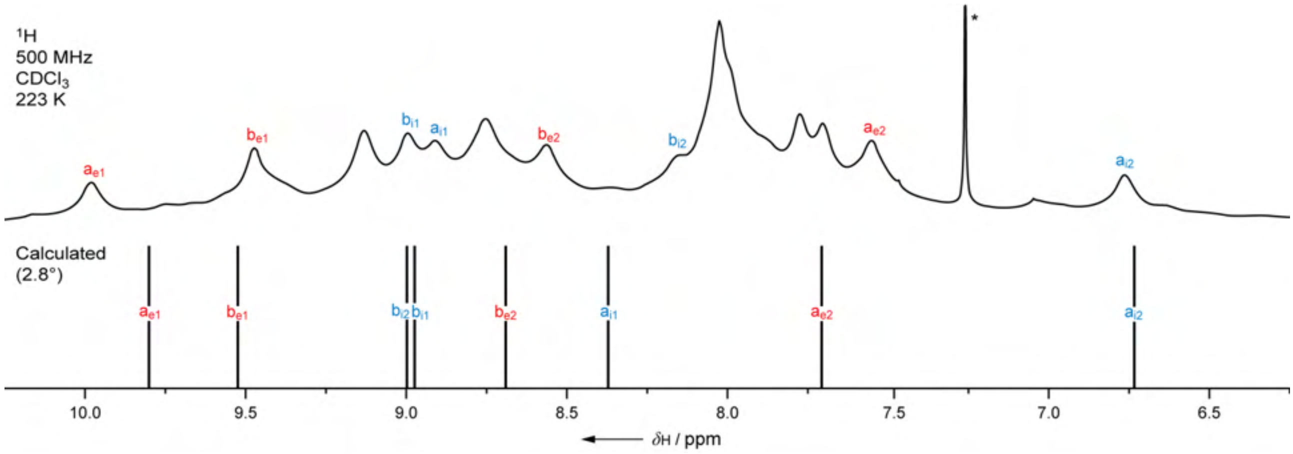

Figure S77. Comparison between experimental (top) and calculated (bottom) beta proton resonances of **(*c*-P12$_{t\text{-Bu}}$)$_2$**. * = $CHCl_3$. The mean absolute error (MAE) between observed and calculated beta proton resonances is 0.33. The calculated order of resonances follows that expected from chemical intuition, i.e., $a_{i2} < a_{i1}$ and $a_{e2} < a_{e1}$, which is in agreement with our assignment of the two porphyrin environments '1' and '2'. Protons a_{i2} and a_{e2} are shifted further up-field (relative to a_{i1} and a_{e1}) as a consequence of being over the perimeter of the porphyrin unit in the neighboring nanoring.

14.5. Molecular dynamics simulations

Methodology. All molecular dynamics (MD) simulations were performed in an isothermal-isobaric (NPT) ensemble at 300 K and 1 bar with a time step of 2 fs using GROMACS (v. 2019.2).[24] All MD simulations were performed including the full solubilizing groups as indicated below. Systems were minimized using the steepest descent algorithm for 5000 steps or until the maximum force on any atom was below 1000 kJ mol^{-1} nm^{-1} and subsequently equilibrated using a velocity-rescaling thermostat[29] and Parrinello-Rahman barostat.[30] Porphyrin residues were restrained during temperature equilibration with position restraints to allow the solubilizing chains to equilibrate with the solvent first. All simulations were performed in explicit chloroform[31] with three-dimensional periodic boundary conditions. The box sizes were chosen by leaving 1 nm distance between solute and box boundary. Long-range electrostatic interactions were calculated using the particle mesh Ewald method.[32] All bond lengths involving hydrogen atoms were constrained with the LINCS algorithm.[33]

Parameters. Simulations employed the General AMBER force field[22] with modifications to parameters for zinc ions and porphyrin connections as previously published.[23] In addition, modifications were made to the butadiyne connections between porphyrins as they tended to favor a perpendicular arrangement of porphyrins rather than staying co-planar, as is found experimentally. Proper dihedral angle parameters were added to four pathways involving the atoms shown in Figure S78a; alkyne atoms were not used in the parameters to avoid structural distortions. Different values for the barrier height of the dihedral angle k_θ were screened by simulating a butadiyne-linked dimer system. The energy profiles were calculated based on a Boltzmann distribution obtained from the dihedral angle populations of five 100 ns MD simulations (Figure S78b). The barrier height of the dihedral angle k_θ was then tuned to reproduce a value close to the experimental value[34] (see Figure S78c). The final parameters are listed in Table S17.

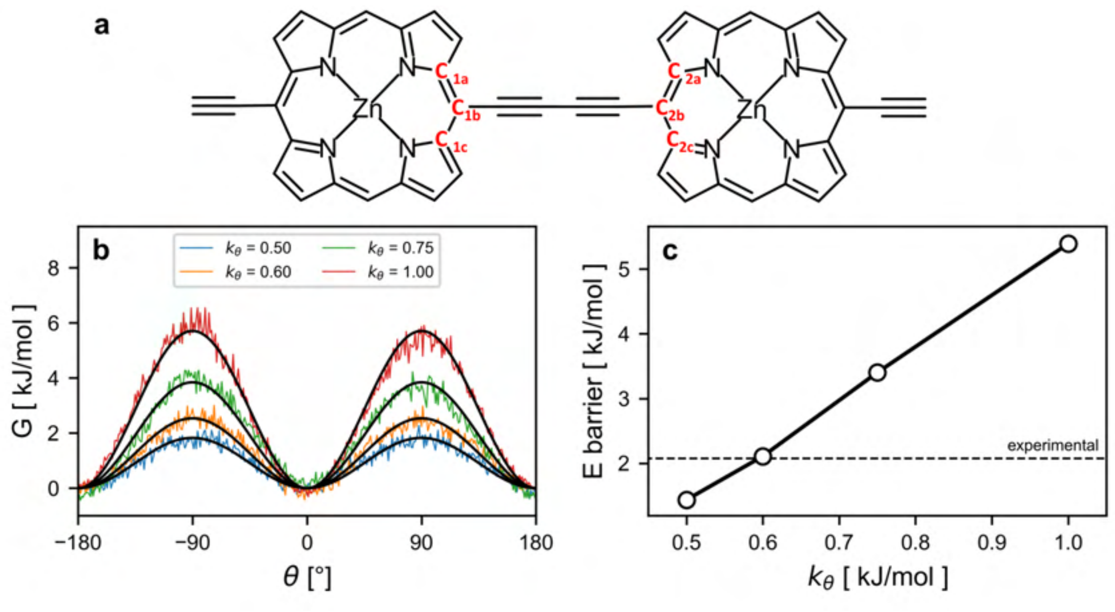

Figure S78. a) Structure of butadiyne-linked porphyrin dimer with atoms involved in additional force field parameters indicated in red. b) Energy profiles relative to co-planar orientation obtained for different values of k_θ and c) corresponding barrier heights.

Table S17. Additional force field parameters. Atom labels are shown in Figure S78a.

**Atom 1**	**Atom 2**	**Atom 3**	**Atom 4**	**Angle [°]**	**k_θ [kJ/mol]**	**Multiplicity**
C_{1a}	C_{1b}	C_{2b}	C_{2a}	180.0	0.6	2
C_{1a}	C_{1b}	C_{2b}	C_{2c}	180.0	0.6	2
C_{1c}	C_{1b}	C_{2b}	C_{2a}	180.0	0.6	2
C_{1c}	C_{1b}	C_{2b}	C_{2c}	180.0	0.6	2

MD simulations on a linear tetramer

Aggregates of a linear butadiyne-linked tetramer, ***l*-P4**, have been chosen as a test system to validate the MD simulations for investigating aggregation behavior, since aggregates of ***l*-P4** have been investigated previously.[35] Simulations were run for 50 ns and started with an assembled trimeric aggregate. The simulations display a stable trimeric aggregate with octyloxy side chains, consistent with the experimentally observed results. However, with *t*-butyl sidechains instead, the trimeric aggregate did not disaggregate, contrary to what would be expected from experiment. Presumably, disaggregation would require longer simulation times than what is feasible to simulate, which shows that meta-stable states may be present in the simulations.

MD simulations on (*c*-P8$_{OOct}$)$_2$ and (*c*-P12$_{t\text{-Bu}}$)$_2$ aggregates

Molecular dynamics trajectories were run for the dimeric aggregates of ***c*-P8** with octyloxy side chains, **(*c*-P8$_{OOct}$)$_2$**, and for ***c*-P12** with *t*-butyl side chains, **(*c*-P12$_{t\text{-Bu}}$)$_2$**. This provided insights into the geometry of the aggregates and into their dynamic processes. The simulations were started form the fully symmetric idealized structures and were repeated three times with different initial random seeds. Both complexes were stable over a simulation time of 100 ns. The **(*c*-P12$_{t\text{-Bu}}$)$_2$** aggregate has a well-defined conformation corresponding to that described in the main text. This is reflected in the unimodal distribution of angles, θ, between the planes of porphyrins and the plane of the overall ring, which was obtained in each of the three runs. The **(*c*-P8$_{OOct}$)$_2$** aggregate, on the other hand, behaved differently in the three simulations and two different distinct conformations were observed: one resembles that of the **(*c*-P12$_{t\text{-Bu}}$)$_2$** aggregate except for a larger angle, corresponding to a more "bowl-shaped" structure (see Figure S79a **(*c*-P8$_{OOct}$)$_2$** Run 1). This larger θ also leads to a larger distance between the porphyrin rings compared to the more planar **(*c*-P12$_{t\text{-Bu}}$)$_2$** aggregate (see Figure S79b). In the other conformation (Run 2 and 3), porphyrins are predominantly perpendicular to the plane of the overall ring which results in the two porphyrin rings being offset to each other with each being on the outside of the structure on half of the ring. This shows the higher fluxionality of the **(*c*-P8$_{OOct}$)$_2$** system with multiple accessible local minima.

Of particular interest is Run 2 of the **(*c*-P8$_{OOct}$)$_2$** aggregate as it displayed a partial disaggregation of two porphyrin units during the simulation. While those porphyrins are detached from the other ring, they experience an increased rotational flexibility and the porphyrins can rotate to angles above 90° (see Figure S79c). In principle, full rotation of the porphyrin should be possible, consistent with the observed exchange correlations in EXSY experiments. However, this is expected to happen at time scales beyond our simulations.

MD simulations were also used to quantify the increased rigidity of the **(*c*-P12$_{t\text{-Bu}}$)$_2$** aggregate structure compared to the single-stranded system. The flattening factor which describes the ellipticity of a cyclic system was calculated as show in Figure S80. Both the single-stranded and the aggregated systems display similar average flattening factors (0.156 and 0.166 respectively) but the single-stranded structure has a considerably broader distribution of values with a standard deviation of 0.070 compared to 0.038 for the **(*c*-P12$_{t\text{-Bu}}$)$_2$** system, consistent with a rigidification due to aggregation.

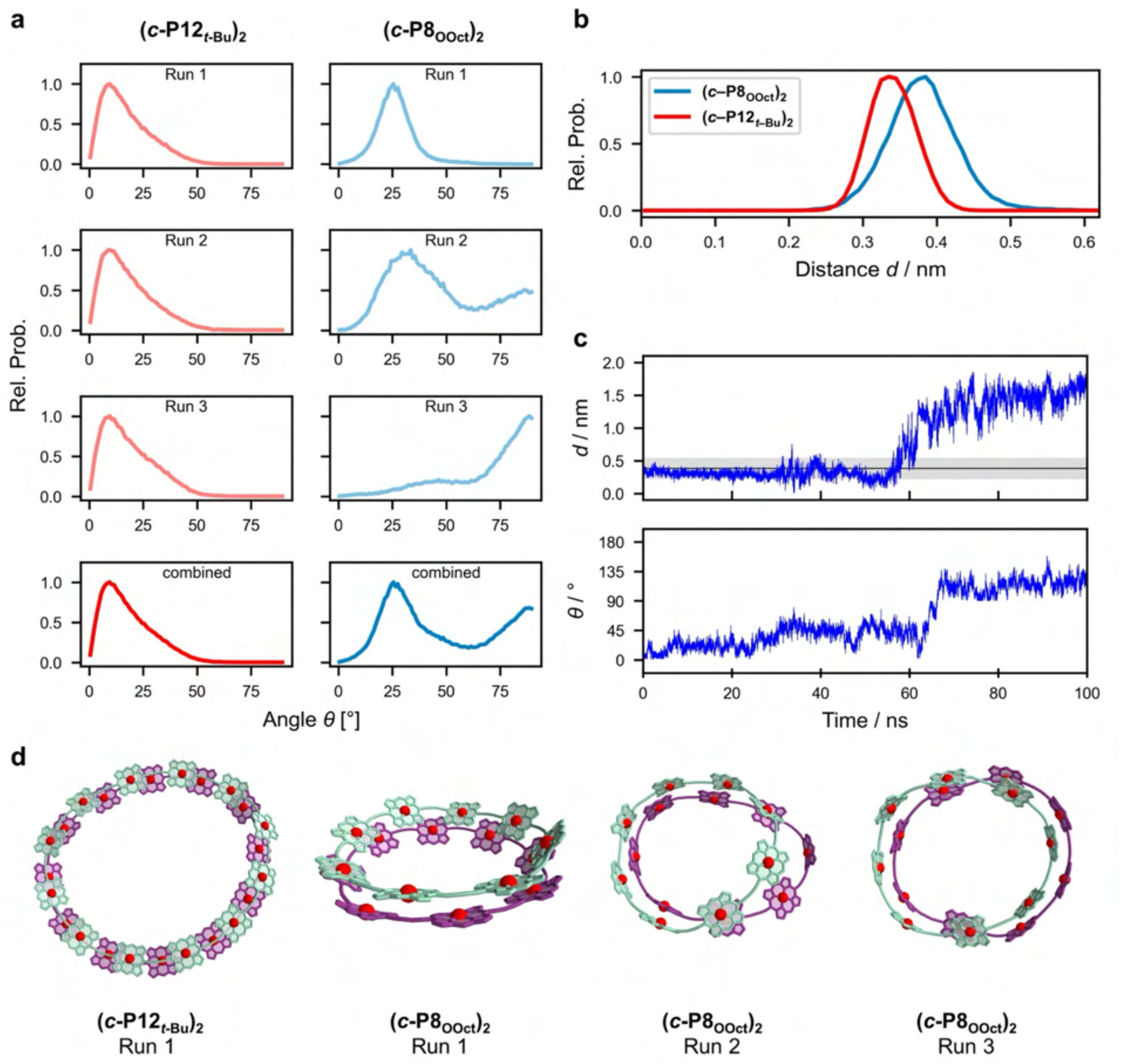

Figure S79. a) Distributions of angle θ between the plane of the ring and the plane of the porphyrin units. b) Distributions of distances d as defined in Section 14.1 of the aggregates. Only run 1 was used for **(*c*-P8$_{\mathbf{OOct}}$)$_2$** to capture a planar complex that is comparable to **(*c*-P12$_{\boldsymbol{t}\mathbf{\text{-}Bu}}$)$_2$**. c) Distance d and angle θ for the porphyrin that detaches during simulation run 2 of **(*c*-P8$_{\mathbf{OOct}}$)$_2$**. The black line and grey area in the distance plot indicate the mean and standard deviation of the assembled structure. (d) Representative structures of selected simulations.

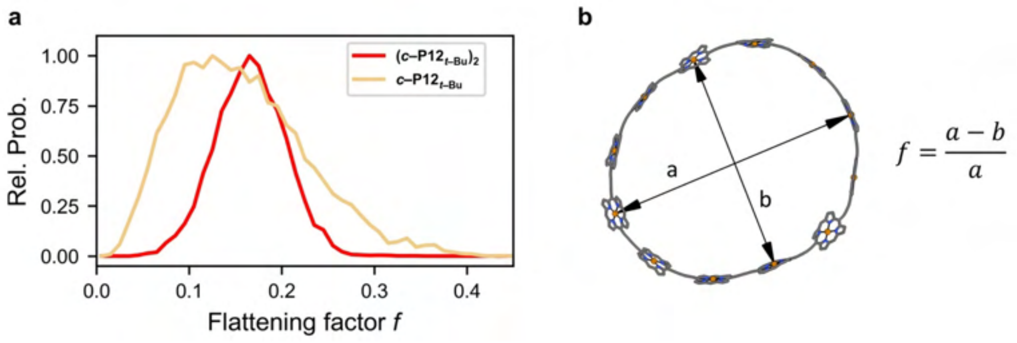

Figure S80. a) Flattening factors of the single-stranded ***c*-P12$_{\boldsymbol{t}\mathbf{\text{-}Bu}}$** and aggregated **(*c*-P12$_{\boldsymbol{t}\mathbf{\text{-}Bu}}$)$_2$** systems. b) Geometric definitions of long axis a, short axis b and flattening factor f.

Section 15. Steady-State Absorption and Fluorescence Spectroscopy

Steady-state UV-vis-NIR absorption spectra were acquired at 298 K on either a Perkin Elmer Lambda 20 spectrometer at 298 K equipped with a PTP-1 Peltier unit from Perkin Elmer or a Perkin Elmer Lambda 25 spectrometer equipped with a PTP-A Peltier unit from Perkin Elmer.

Steady-state fluorescence spectra were acquired at 298 K using an Edinburgh Instruments FS5 spectrofluorometer operating Fluoracle® software, equipped with a xenon arc lamp (providing 230–1000 nm excitation range), a thermostatic sample holder (SC-20) and both an R13456 PMT detector (200–950 nm spectral coverage, Hamamatsu) and an InGaAs analogue NIR detector (850–1650 nm spectral coverage).

The fluorescence quantum yields were determined using the relative method according to the following formula, Eqn. S12:[36]

$$\Phi_s = \Phi_{ref} \cdot \frac{\alpha_s}{\alpha_r} \cdot \frac{n_s^2}{n_r^2} \qquad \text{(Eqn. S12)}$$

The subscript 's' designates the sample of interest, while 'r' designates the reference dye (***l*-P2**, $\Phi_F = 0.25$ in toluene, $\lambda_{ex} = 450$ nm),[37] n is the refractive index of the solvent (1.5059 for toluene and 1.4495 for chloroform) and α is the slope obtained from a linear-fit to a set of data points (I_{int} vs. f_a). Here, I_{int} refers to the integrated emission intensity and f_a to the fraction of absorbed light, calculated according to the following formula, Eqn. S13:

$$f_a = \int_{\lambda_{ex}-1/2\Delta\lambda_{ex}}^{\lambda_{ex}-1/2\Delta\lambda_{ex}} (1 - 10^{-A(\lambda_{ex})}) d\lambda_{ex} \qquad \text{(Eqn. S13)}$$

where $\Delta\lambda_{ex}$ refers to the slit width of excitation. For all quantum yield determinations four or more data points were collected with a measured absorbance below 0.1 for the longest wavelength absorption. As reference dye we used the butadiyne-linked porphyrin dimer, ***l*-P2**, shown in Figure S81.

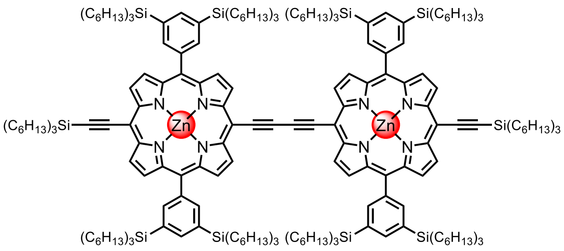

***l*-P2**

Figure S81. Structure of ***l*-P2** used as reference dye for fluorescence quantum yield measurements ($\Phi_F = 0.25$ in toluene, $\lambda_{ex} = 450$ nm).[37]

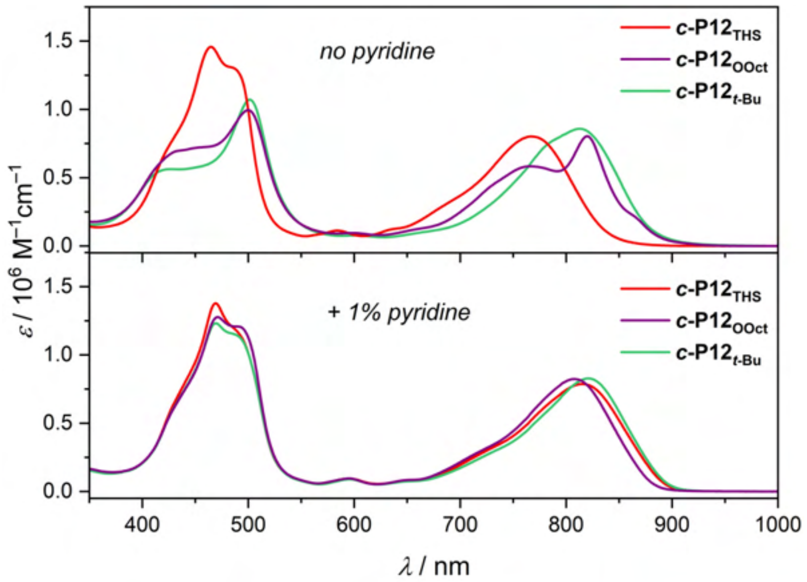

Figure S82. Absorption spectra of ***c*-P12** rings (THS, OOct, and *t*-Bu side chains) in the absence and presence of 1% pyridine, recorded in $CDCl_3$ at 25 °C.

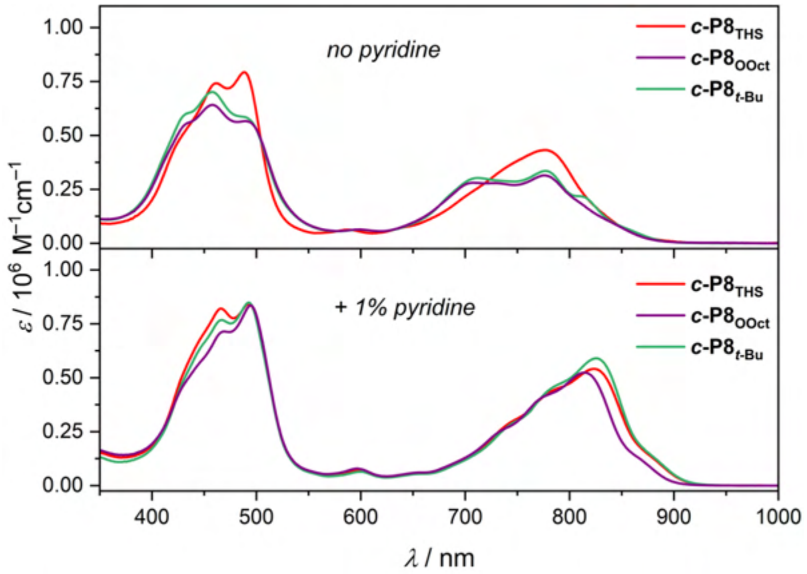

Figure S83. Absorption spectra of ***c*-P8** rings (THS, OOct, and *t*-Bu side chains) in the absence and presence of 1% pyridine, recorded in $CDCl_3$ at 25 °C.

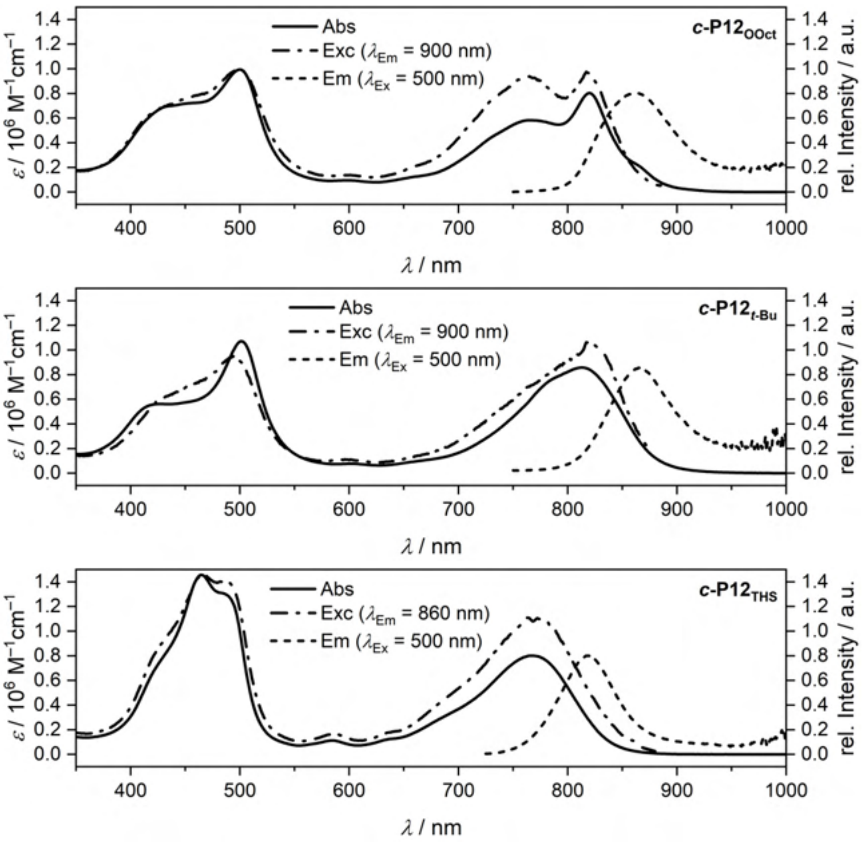

Figure S84. Absorption (Abs), excitation (Exc), and emission (Em) spectra of ***c*-P12$_{OOct}$** (top), ***c*-P12$_{t\text{-}Bu}$** (middle), and ***c*-P12$_{THS}$** (bottom) in $CDCl_3$ at 25 °C.

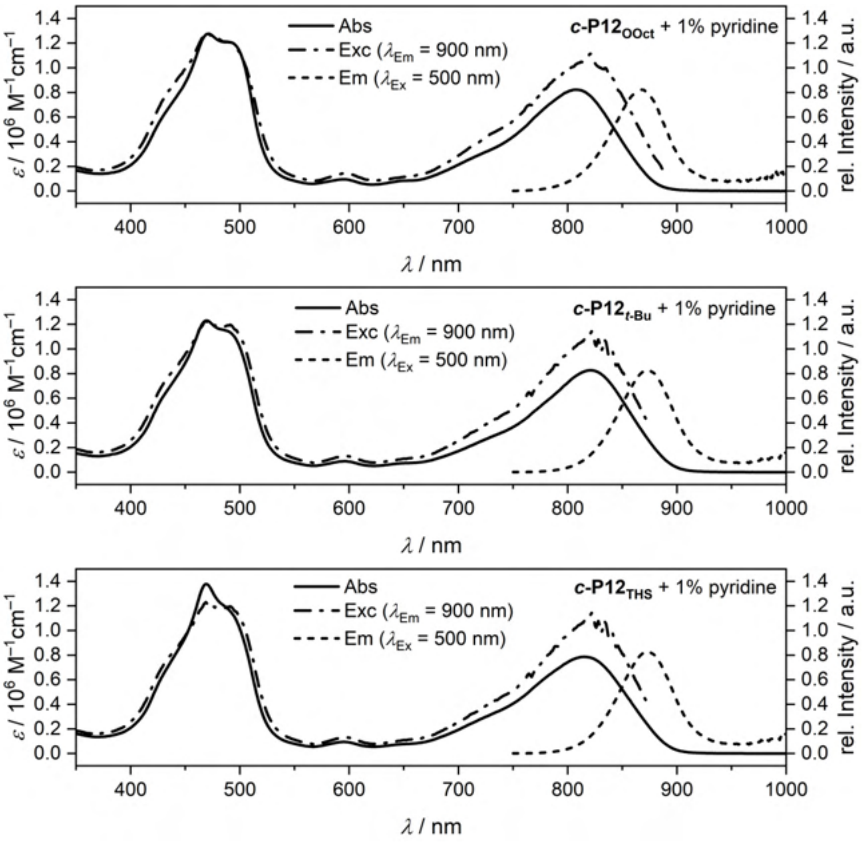

Figure S85. Absorption (Abs), excitation (Exc), and emission (Em) spectra of ***c*-P12$_{OOct}$** (top), ***c*-P12$_{t\text{-}Bu}$** (middle), and ***c*-P12$_{THS}$** (bottom) in $CDCl_3$ + 1% pyridine at 25 °C.

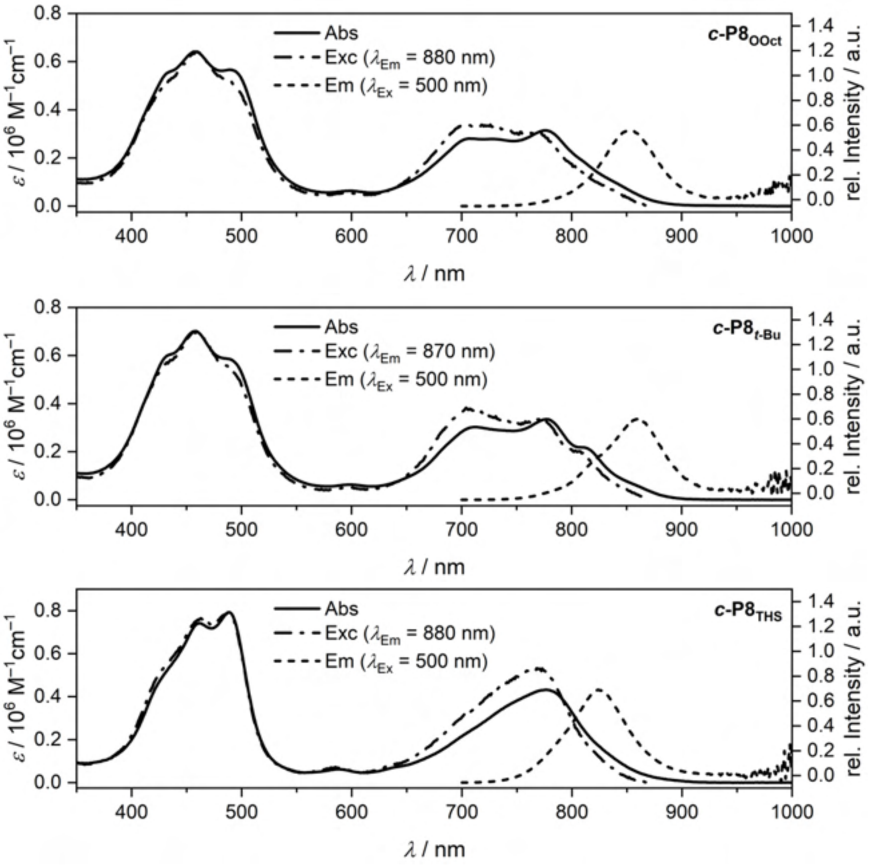

Figure S86. Absorption (Abs), excitation (Exc), and emission (Em) spectra of ***c*-P8**$_{\mathbf{OOct}}$ (top), ***c*-P8**$_{\boldsymbol{t}\text{-}\mathbf{Bu}}$ (middle), and ***c*-P8**$_{\mathbf{THS}}$ (bottom) in $CDCl_3$ at 25 °C.

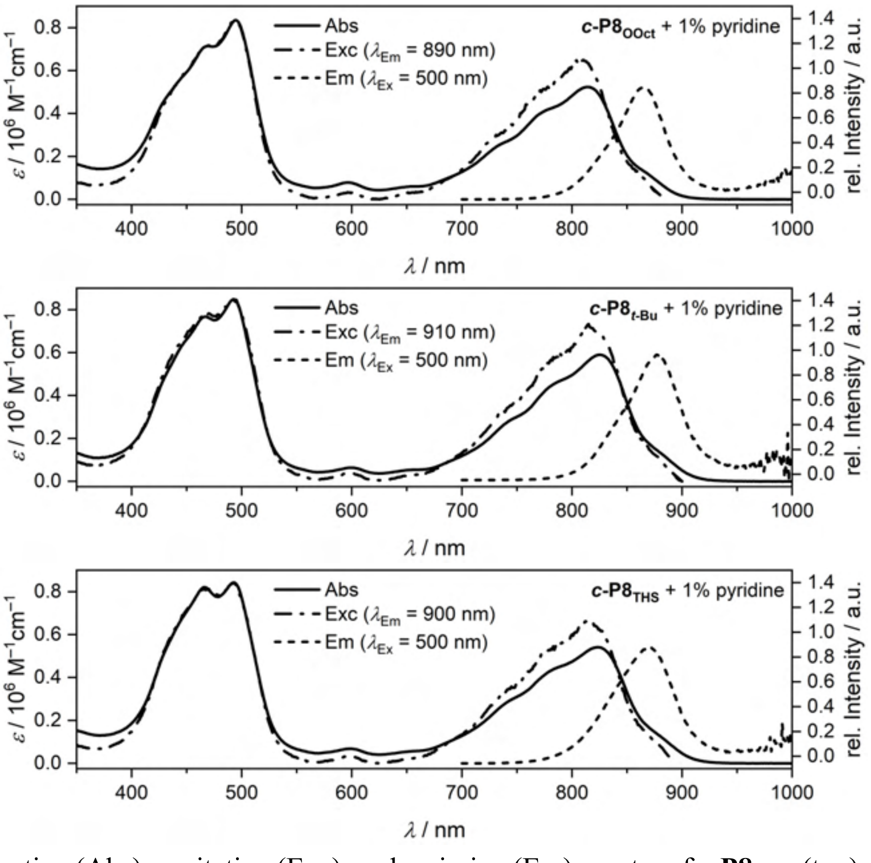

Figure S87. Absorption (Abs), excitation (Exc), and emission (Em) spectra of ***c*-P8**$_{\mathbf{OOct}}$ (top), ***c*-P8**$_{\boldsymbol{t}\text{-}\mathbf{Bu}}$ (middle), and ***c*-P8**$_{\mathbf{THS}}$ (bottom) in $CDCl_3$ + 1% pyridine at 25 °C.

Table S18. Summary of steady-state photophysical parameters in chloroform at 25 °C.

Compound	$\lambda_{Abs,max}$ / nm	ε_{max} / 10^6 M^{-1} cm^{-1}	$\lambda_{Em,max}$ / nm	τ_F[a] / ns	Φ_F
***c*-P12**$_{OOct}$	820, 767, 598, 500	0.803, 0.583, 0.095, 0.995	862	0.66	0.020
***c*-P12**$_{OOct}$ + 1% pyr	808, 595, 471	0.823, 0.096, 1.277	859	0.60	0.16
***c*-P12**$_{t\text{-}Bu}$	813, 601, 501	0.858, 0.0778, 1.071	867	0.59	0.0017
***c*-P12**$_{t\text{-}Bu}$ + 1% pyr	821, 596, 469	0.828, 0.0894, 1.231	873	0.50	0.11
***c*-P12**$_{THS}$	767, 584, 465	0.801, 0.113, 1.457	819	0.91	0.26
***c*-P12**$_{THS}$ + 1% pyr	815, 595, 469	0.787, 0.095, 1.379	867	0.55	0.15
***c*-P8**$_{OOct}$	775, 729, 707, 597, 489, 458,	0.314, 0.279, 0.281, 0.065, 0.566, 0.642	853	0.82	0.039
***c*-P8**$_{OOct}$ + 1% pyr	814, 596, 495, 469	0.524, 0.079, 0.834, 0.714	866	0.51	0.050
***c*-P8**$_{t\text{-}Bu}$	777, 712, 598, 458	0.335, 0.303, 0.063, 0.702	859	0.81	0.037
***c*-P8**$_{t\text{-}Bu}$ + 1% pyr	825, 600, 493, 467	0.590, 0.064, 0.848, 0.767	877	0.40	0.033
***c*-P8**$_{THS}$	776, 586, 488, 462	0.432, 0.063, 0.793, 0.742	824	0.88	0.12
***c*-P8**$_{THS}$ + 1% pyr	823, 599, 493, 466	0.541, 0.068, 0.842, 0.821	870	0.45	0.045

[a] Fluorescence lifetimes were measured using the Edinburgh Instruments FS5 spectrofluorometer in time-correlated single photon counting (TCSPC) mode using a picosecond pulsed diode laser (EPL-475) as the excitation source; excitation wavelength: 473 nm. Exponential re-convolution fits incorporating the measured instrument response function were used to extract fluorescence lifetimes.

Section 16. Aggregate Break-Up Experiments

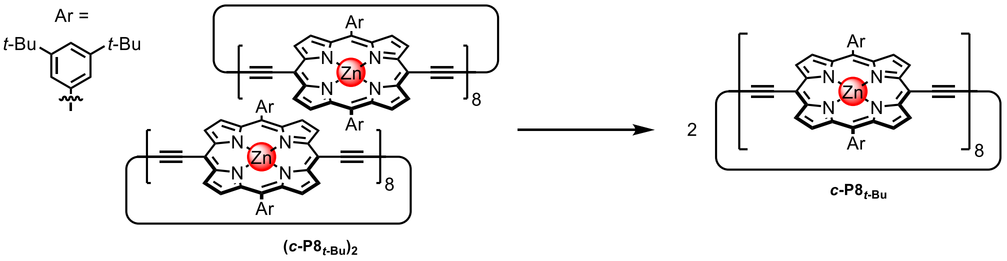

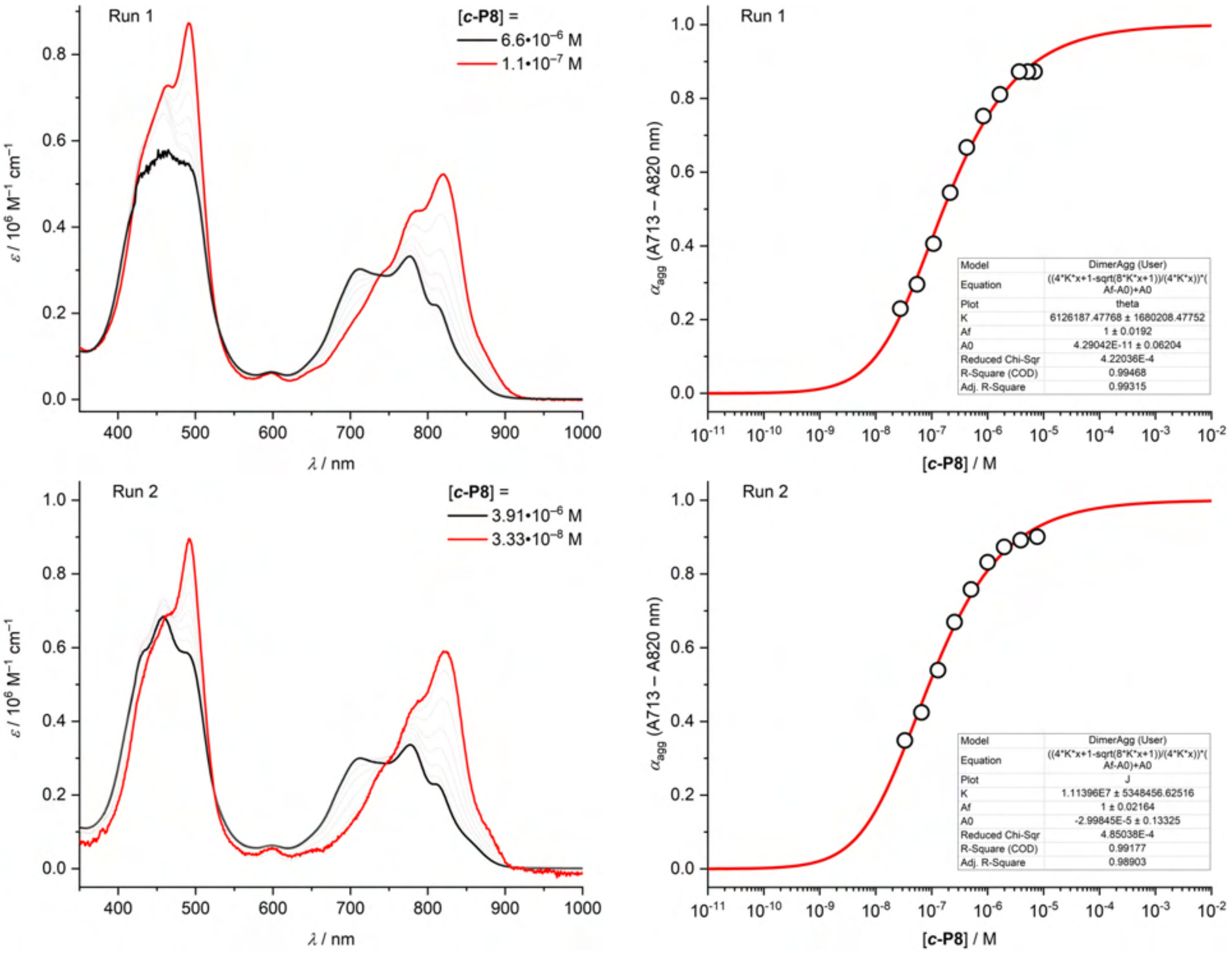

Figure S88. UV-vis dilution of **(c-P8$_{t\text{-Bu}}$)$_2$** in $CDCl_3$ at 25 °C and fitting of the isotherm to a dimerization model. The extent of dimerization α_{agg} was measured as the normalized change in wavelength ΔA = 713 – 820 nm. Run 1: c = from $6.6\cdot10^{-6}$ to $1.1\cdot10^{-7}$ M, $K_{agg} = 6.1\cdot10^{6}$ M^{-1}, $R^2 = 0.99315$. Run 2: c = from $3.9\cdot10^{-6}$ to $3.3\cdot10^{-8}$ M, $K_{agg} = 1.1\cdot10^{7}$ M^{-1}, $R^2 = 0.98903$. Average $K_{agg} = 8.6\cdot10^{6}$ M^{-1}.

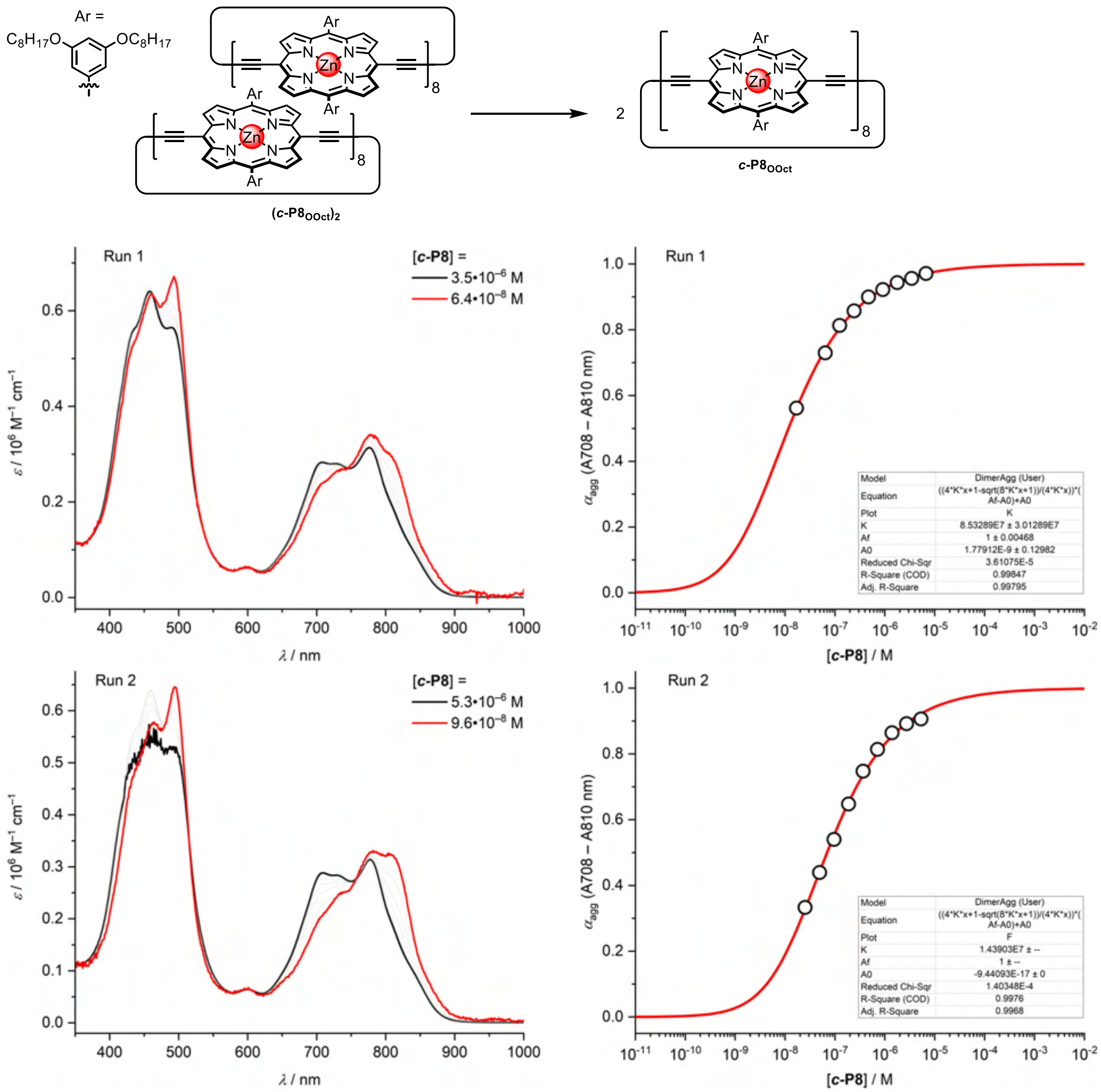

Figure S89. UV-vis dilution of **(*c*-P8$_{\text{OOct}}$)$_2$** in $CDCl_3$ at 25 °C and fitting of the isotherm to a dimerization model. The extent of dimerization α_{agg} was measured as the normalized change in wavelength $\Delta A = 708 - 810$ nm. Run 1: c = from $3.5 \cdot 10^{-6}$ to $6.4 \cdot 10^{-8}$ M, $K_{agg} = 8.5 \cdot 10^7$ M^{-1}, $R^2 = 0.99795$. Run 2: c = from $5.3 \cdot 10^{-6}$ to $9.6 \cdot 10^{-8}$ M, $K_{agg} = 1.4 \cdot 10^7$ M^{-1}, $R^2 = 0.99680$. Average $K_{agg} = 5.0 \cdot 10^7$ M^{-1}.

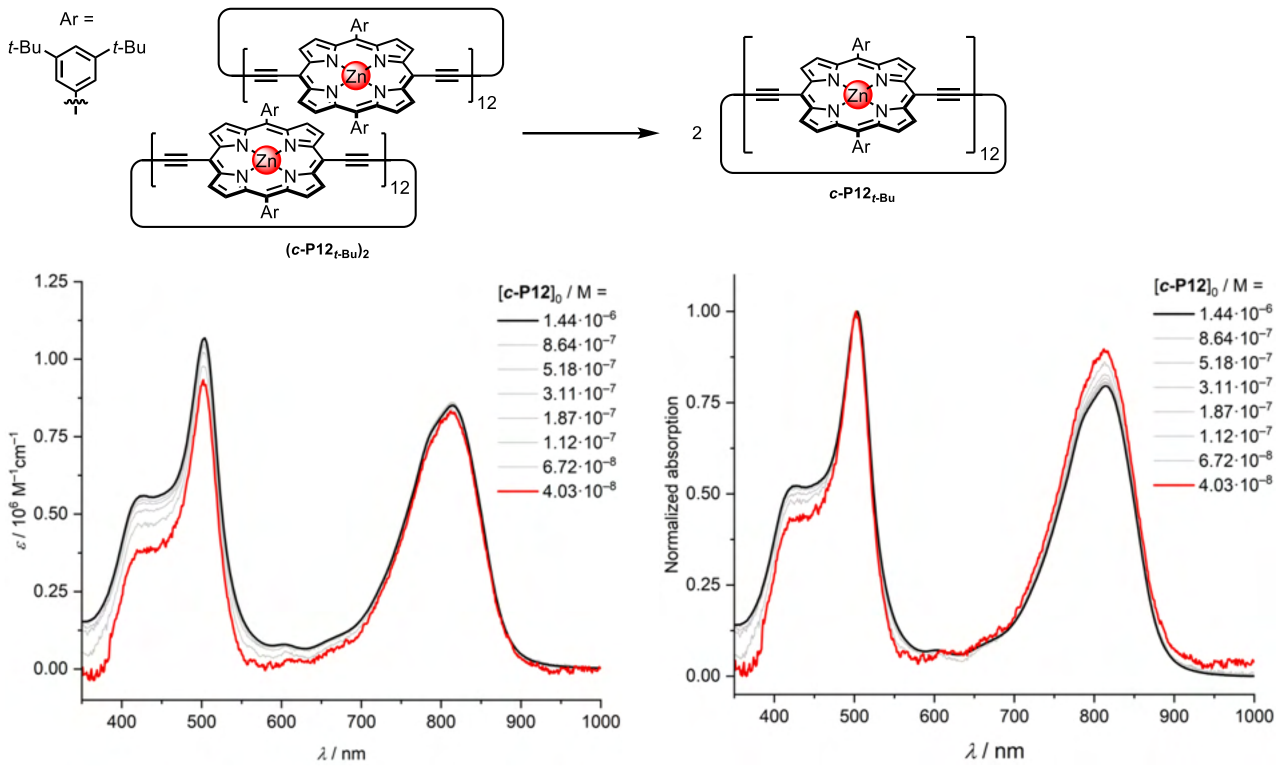

Figure S90. UV-vis dilution series of **(*c*-P12$_{t\text{-Bu}}$)$_2$** in $CDCl_3$ at 25 °C. Left: molar extinction coefficient (e) at different total concentrations ([***c*-P12**]$_0$) versus wavelength (λ). Right: normalized absorption at different total concentrations versus wavelength. At low concentrations it becomes difficult to measure the absorption reliably with our setup.

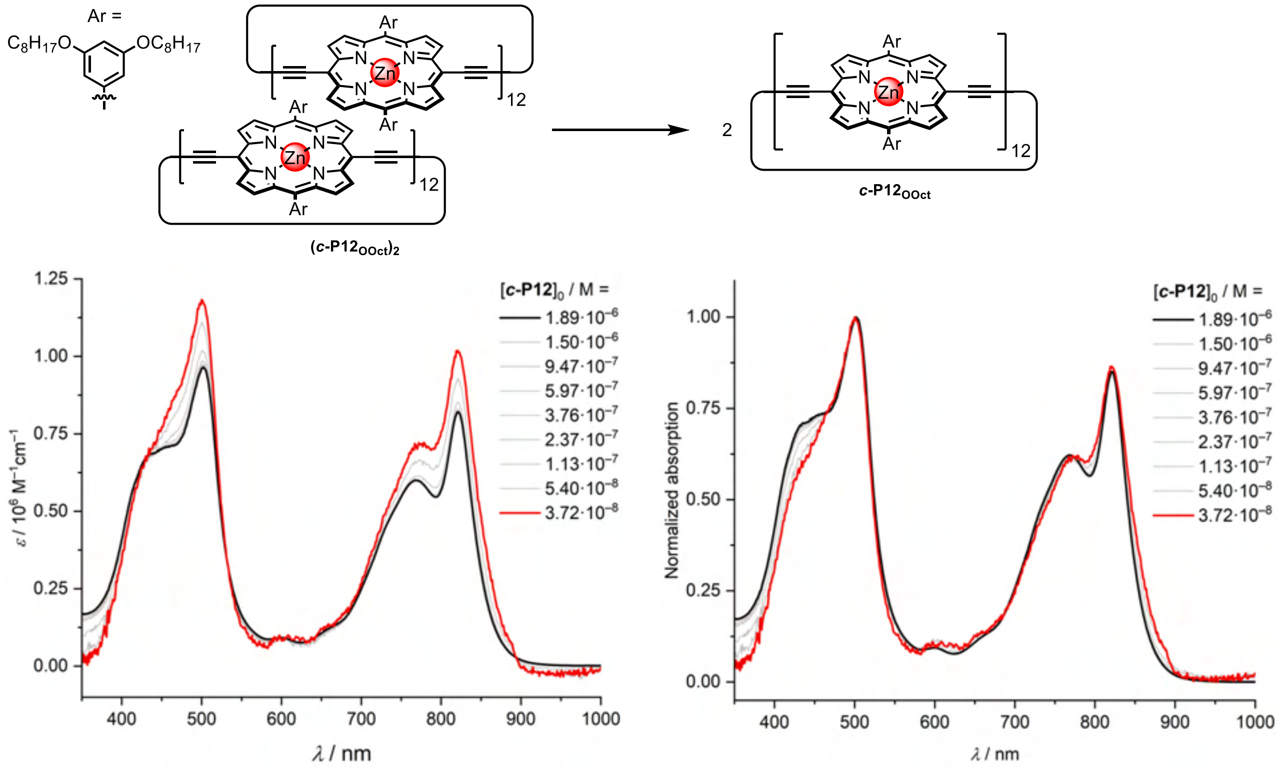

Figure S91. UV-vis dilution series of **(*c*-P12$_{OOct}$)$_2$** in $CDCl_3$ at 25 °C. Left: molar extinction coefficient (e) at different total concentrations ([***c*-P12**]$_0$) versus wavelength (λ). Right: normalized absorption at different total concentrations versus wavelength. At low concentrations it becomes difficult to measure the absorption reliably with our setup.

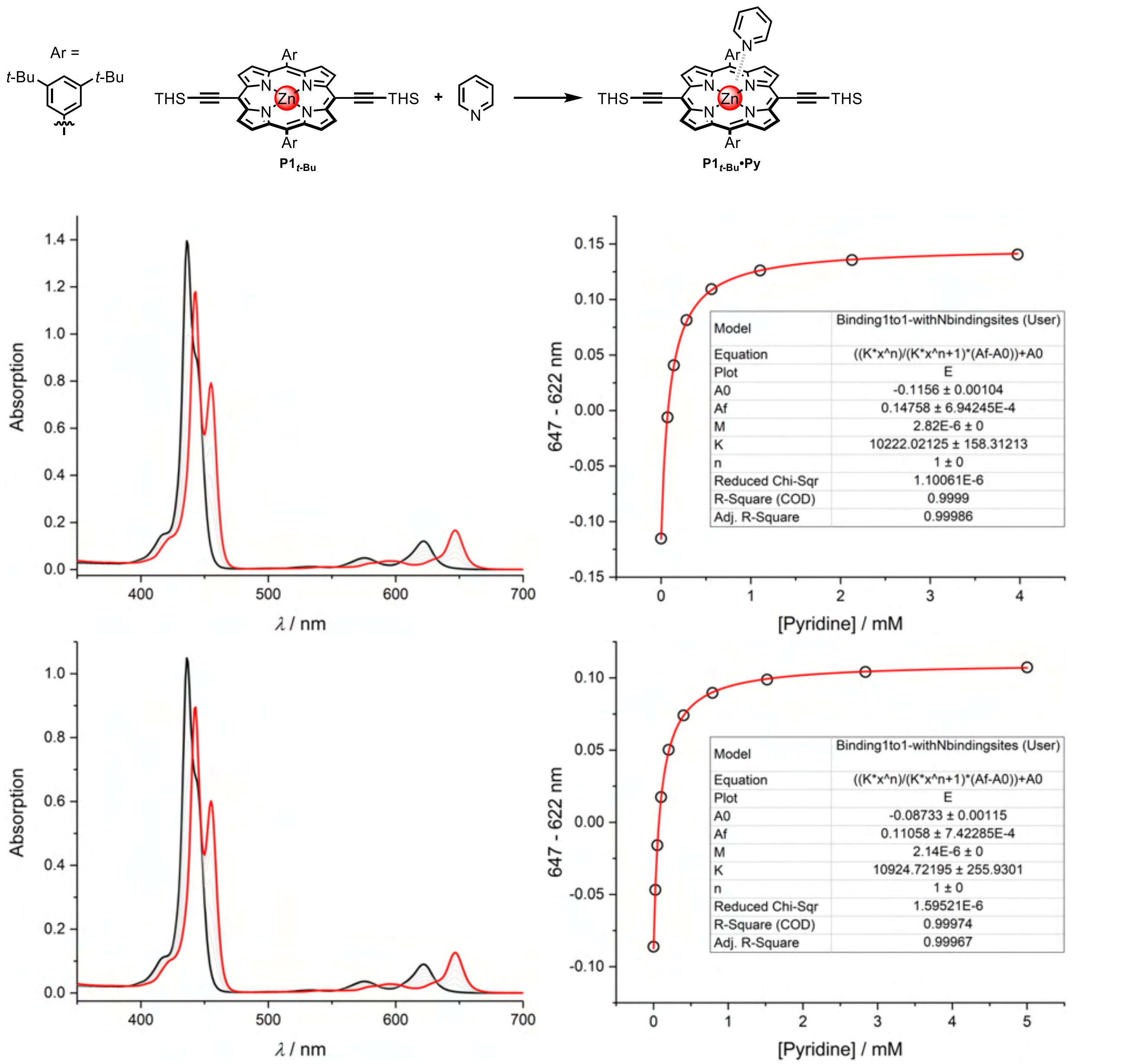

Figure S92. UV-vis titration of **P1**$_{t\text{-Bu}}$ and pyridine in $CDCl_3$ at 298 K and fitting of the data to a one-to-one binding model. Top: binding model scheme. Middle: Run 1 ([**P1**] = 2.82 × 10^{-6} M, K_f = 1.02 × 10^4 M^{-1}, R^2 = 0.99986); Bottom: Run 2 ([**P1**] = 2.14 × 10^{-6} M, K_f = 1.09 × 10^4 M^{-1}, R^2 = 0.99967). Average K_f = 1.06 × 10^4 M^{-1}. Ar = 3,5-bis(*tert*-butyl)phenyl.

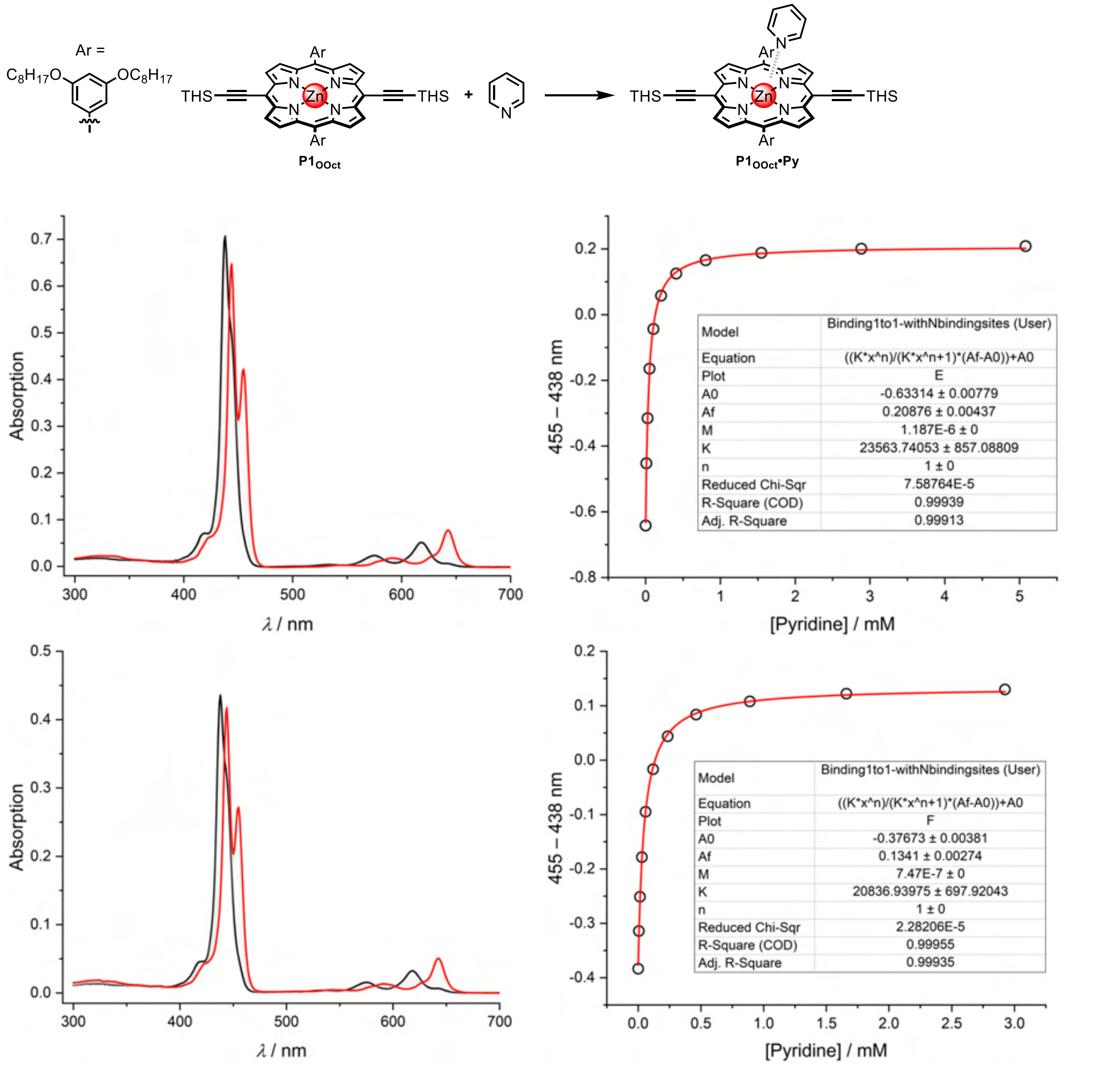

Figure S93. UV-vis titration of **P1**$_{\mathbf{OOct}}$ and pyridine in $CDCl_3$ at 298 K and fitting of the data to a one-to-one binding model. Top: binding model scheme. Middle: Run 1 ([**P1**] = 1.19 × 10^{-6} M, K_f = 2.36 × 10^4 M^{-1}, R^2 = 0.99913); Bottom: Run 2 ([**P1**] = 7.47 × 10^{-7} M, K_f = 2.08 × 10^4 M^{-1}, R^2 = 0.99935). Average K_f = 2.22 × 10^4 M^{-1}. Ar = 3,5-bis(octyloxy)phenyl.

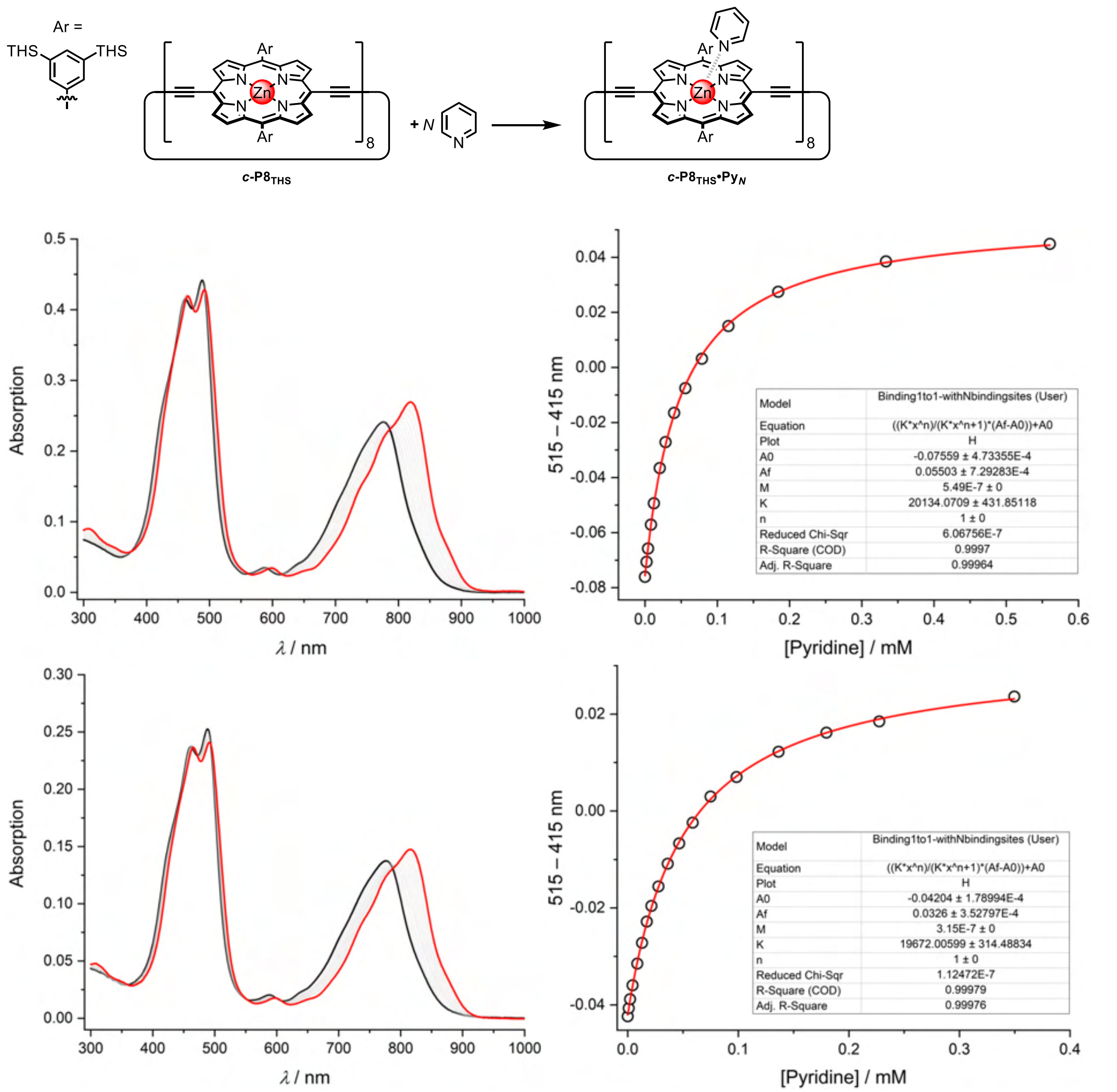

Figure S94. UV-vis titration of ***c*-P8**$_{\mathbf{THS}}$ and pyridine in $CDCl_3$ at 25 °C and fitting of the data to a one-to-one binding model. Top: model scheme. Middle: Run 1 ([***c*-P8**] = 5.49 × 10^{-7} M, K = 2.01 × 10^4 M^{-1}, R^2 = 0.99964); Bottom: Run 2 ([***c*-P8**] = 3.15 × 10^{-7} M, K = 1.97 × 10^4 M^{-1}, R^2 = 0.99976). Average K = 1.99 × 10^4 M^{-1}.

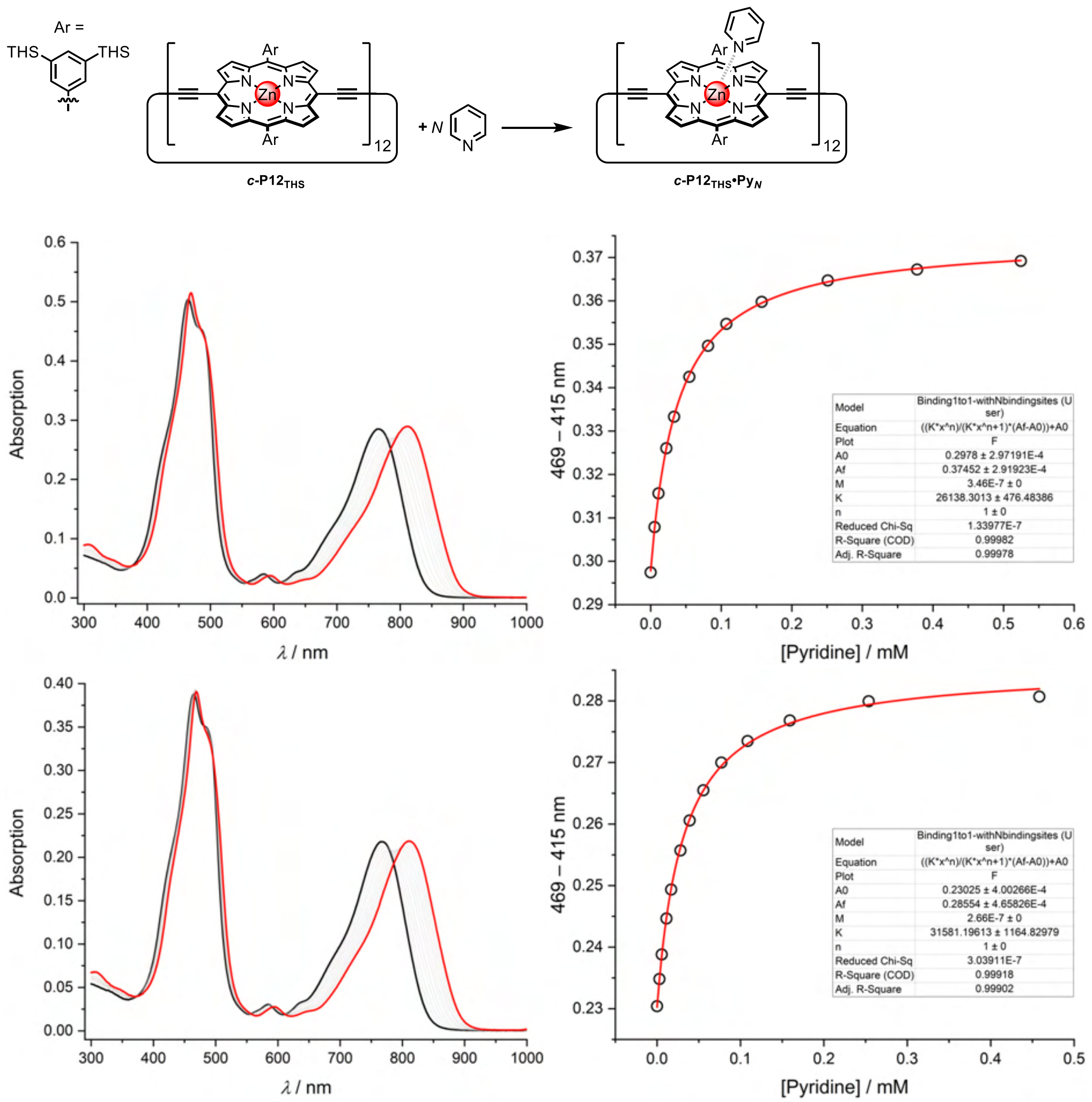

Figure S95. UV-vis titration of ***c*-P12**$_{\mathbf{THS}}$ and pyridine in $CDCl_3$ at 25 °C and fitting of the data to a one-to-one binding model. Top: model scheme. Middle: Run 1 ([***c*-P12**] = 1.34 × 10^{-7} M, K = 2.61 × 10^4 M^{-1}, R^2 = 0.99978); Bottom: Run 2 ([***c*-P12**] = 2.66 × 10^{-7} M, K = 3.16 × 10^4 M^{-1}, R^2 = 0.99902). Average K = 2.89 × 10^4 M^{-1}.

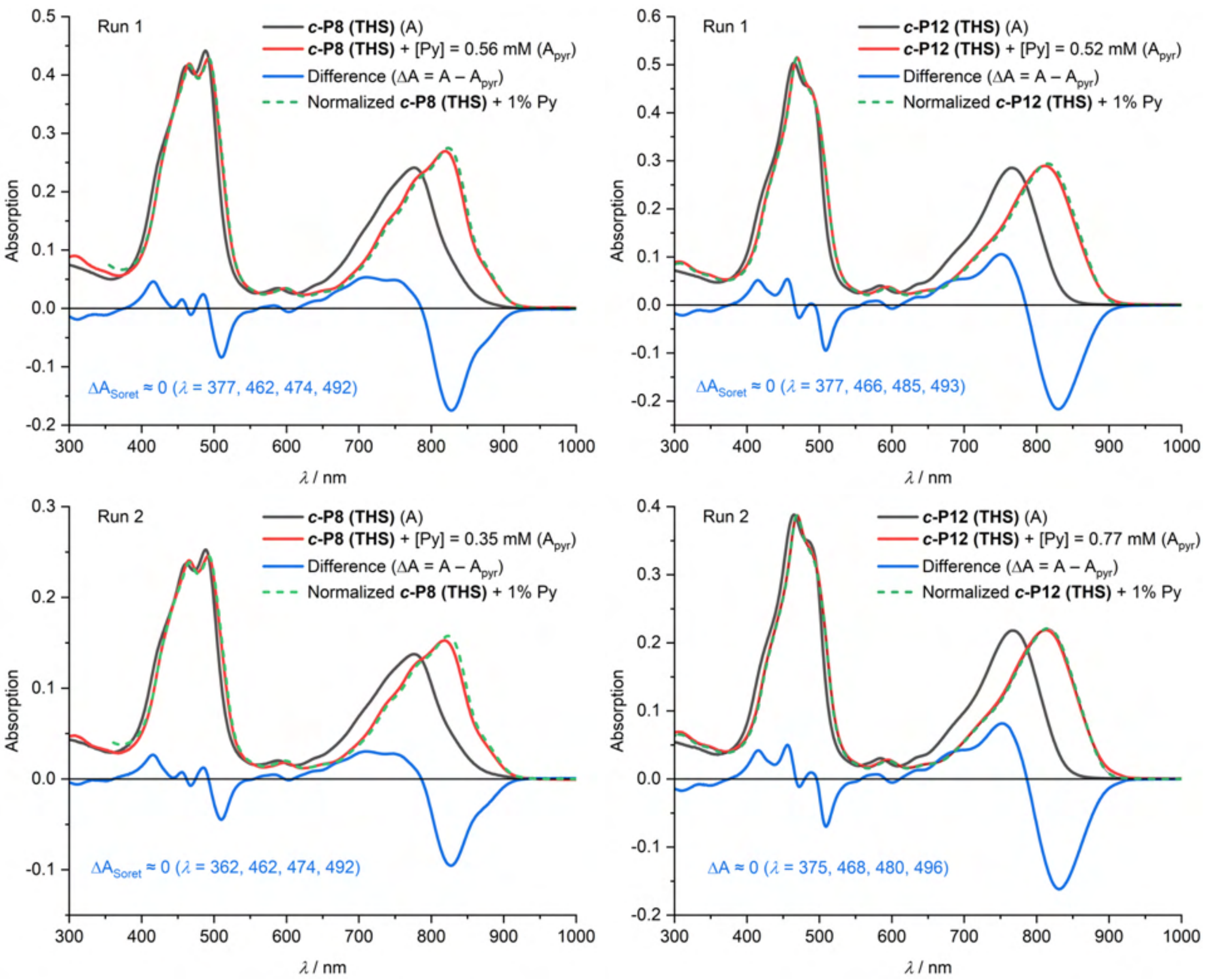

Figure S96. Change in absorption as a result of pyridine binding for ***c*-P8**$_{\text{THS}}$ (left) and ***c*-P12**$_{\text{THS}}$ (right) with THS sidechains. The absorption difference ($\Delta A = A - A_{pyr}$) is taken between the endpoints from titrations presented in Figure S94 (***c*-P8**$_{\text{THS}}$) and Figure S95 (***c*-P12**$_{\text{THS}}$). Normalized spectra of both ***c*-P8**$_{\text{THS}}$ and ***c*-P12**$_{\text{THS}}$ rings in presence of 1% pyridine (green, dashed line) are included to show that these are approximately identical to the A_{pyr} spectra (red, full line).

For dimer aggregates of ***c*-P8** and ***c*-P12** rings of any sidechain, a change in UV may stem from both binding of pyridine and from disaggregation. To monitor the extent of disaggregation, it is desirable to limit contributions due to binding of pyridine. To get an idea about the UV changes due to pyridine binding across the absorption spectrum, we compared ring systems ***c*-P8**$_{\text{THS}}$ and ***c*-P12**$_{\text{THS}}$, unable to form a bimolecular aggregate, in the absence and presence of pyridine. It is clear that changes due to pyridine binding is most pronounced in the Q band region and less so in the Soret band region. For ***c*-P8**$_{\text{THS}}$, especially around wavelengths 377/362, 462, 474, and 492 nm, $\Delta A \approx 0$ for binding of pyridine. For ***c*-P12**$_{\text{THS}}$, especially around wavelengths, 377/375, 466/468, 485/480, and 493/496 nm, $\Delta A \approx 0$ for binding of pyridine.

The type of sidechain may also impact the change in UV resulting from the binding of pyridine. Yet, from examination of the ***c*-P8** and ***c*-P12** absorption plots (Section 15, Figure S83 and S82), it is clear that the type of sidechain (*t*-Bu, OOct, or THS) only has a minor influence on the UV-absorption in the presence of pyridine (i.e., the Soret and Q band regions of *t*-Bu and OOct ***c*-P8** and ***c*-P12** rings are very similar to those of THS ***c*-P8** and ***c*-P12** rings, respectively).

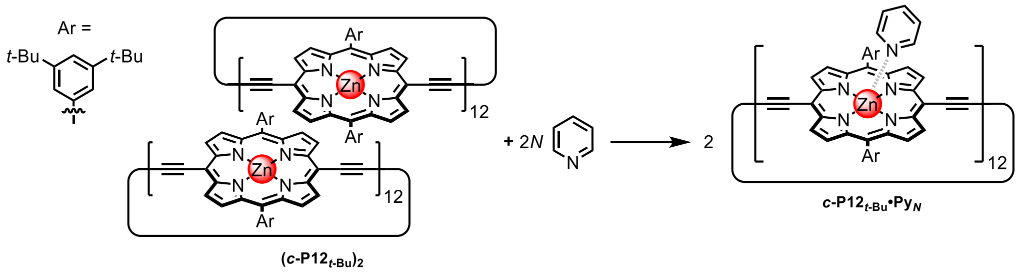

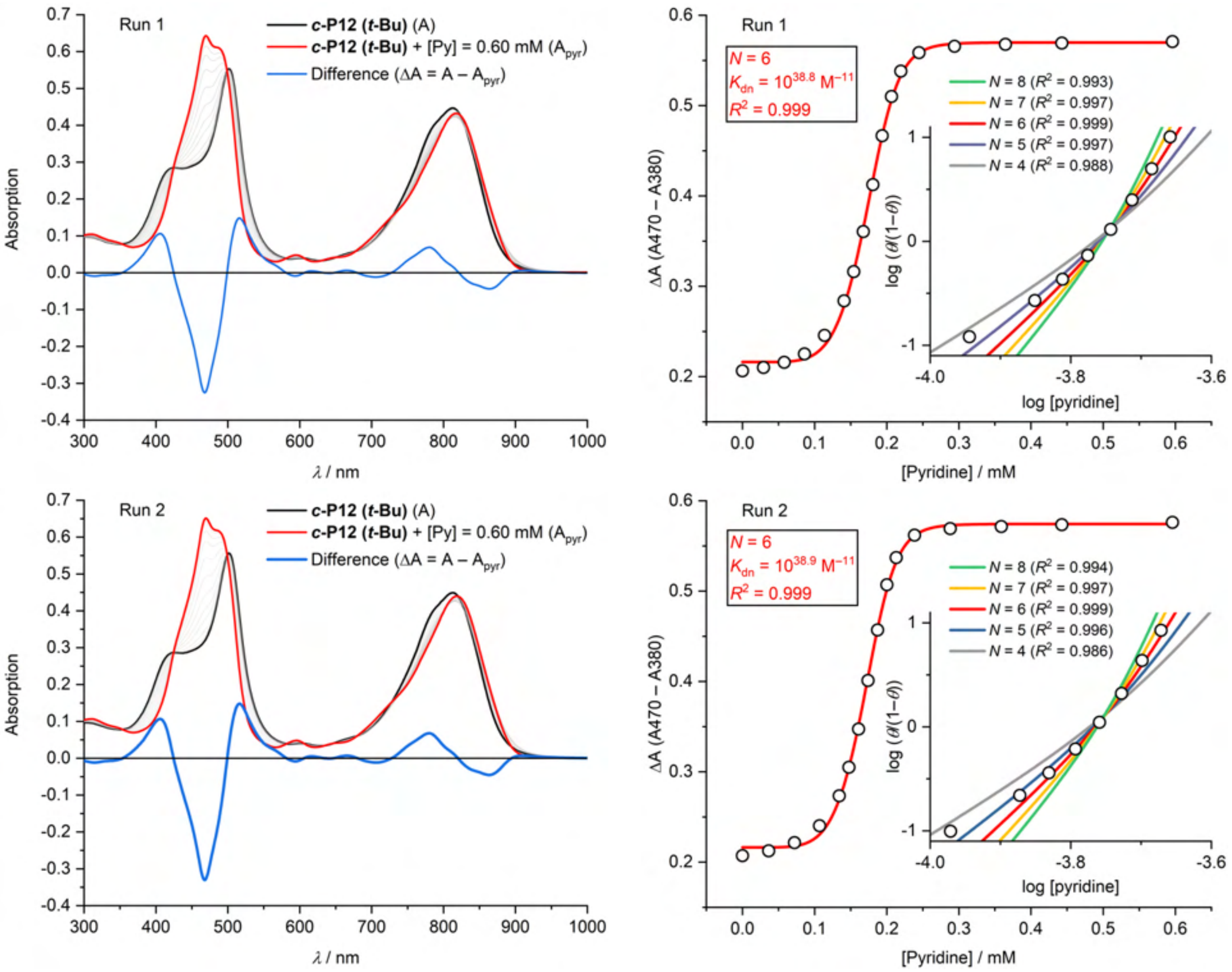

Figure S97. UV-Vis denaturation titrations of **(c-P12$_{t\text{-Bu}}$)$_2$** with pyridine in $CDCl_3$ at 25 °C. Absorption spectra and denaturation isotherm including Hill plots as the inset are shown for two runs 1 and 2. The changes observed in the Soret band region where fitted to a 1-to-2 denaturation model with $N = 6$ resulting in the best fit. Run 1: ([**c-P12**] = 5.00 × 10^{-7} M, K_{dn} = $10^{38.8}$ M^{-11}, $N = 6$, R^2 = 0.99872). Best Hill coefficient: n_H = 7.7. Run 2: ([**c-P12**] = 5.00 × 10^{-7} M, K_{dn} = $10^{38.9}$ M^{-11}, $N = 6$, R^2 = 0.99884). Average K_{dn} is = $10^{38.85}$ M^{-11}. Best Hill coefficient: n_H = 7.4.

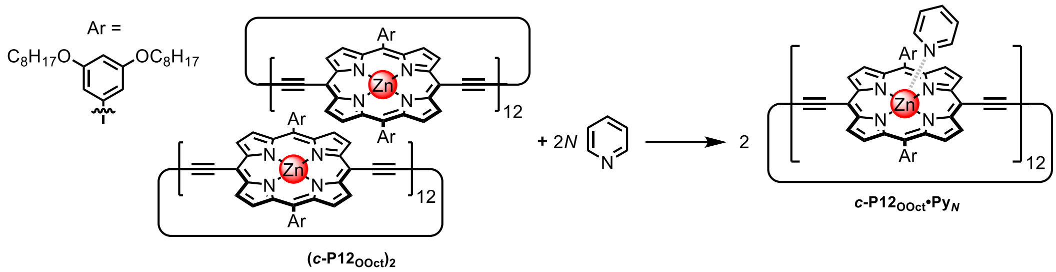

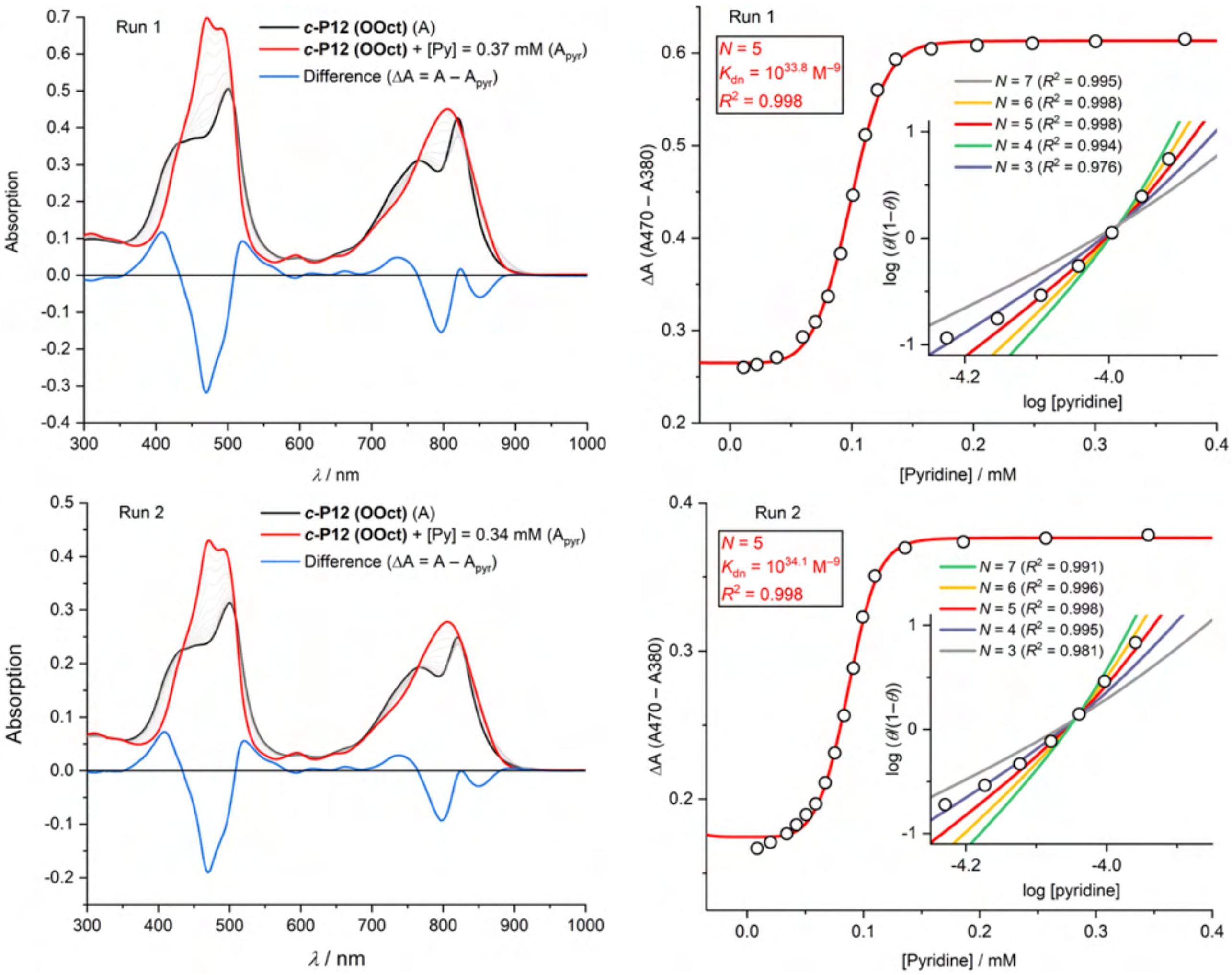

Figure S98. UV-Vis denaturation titrations of **(*c*-P12$_{OOct}$)$_2$** with pyridine in $CDCl_3$ at 25 °C. Absorption spectra and denaturation isotherm including Hill plots as the inset are shown for two runs 1 and 2. The changes observed in the Soret band region where fitted to a 1-to-2 denaturation model with $N = 5$ resulting in the best fit. Run 1: ([***c*-P12**] = 5.26 × 10^{-7} M, $K_{dn} = 10^{33.8}$ M^{-9}, $N = 5$, $R^2 = 0.99844$). Best Hill coefficient: $n_H = 6.6$. Run 2: ([***c*-P12**] = 3.28 × 10^{-7} M, $K_{dn} = 10^{34.1}$ M^{-9}, $N = 5$, $R^2 = 0.99778$). Average K_{dn} is = $10^{34.0}$ M^{-9}. Best Hill coefficient: $n_H = 6.5$.

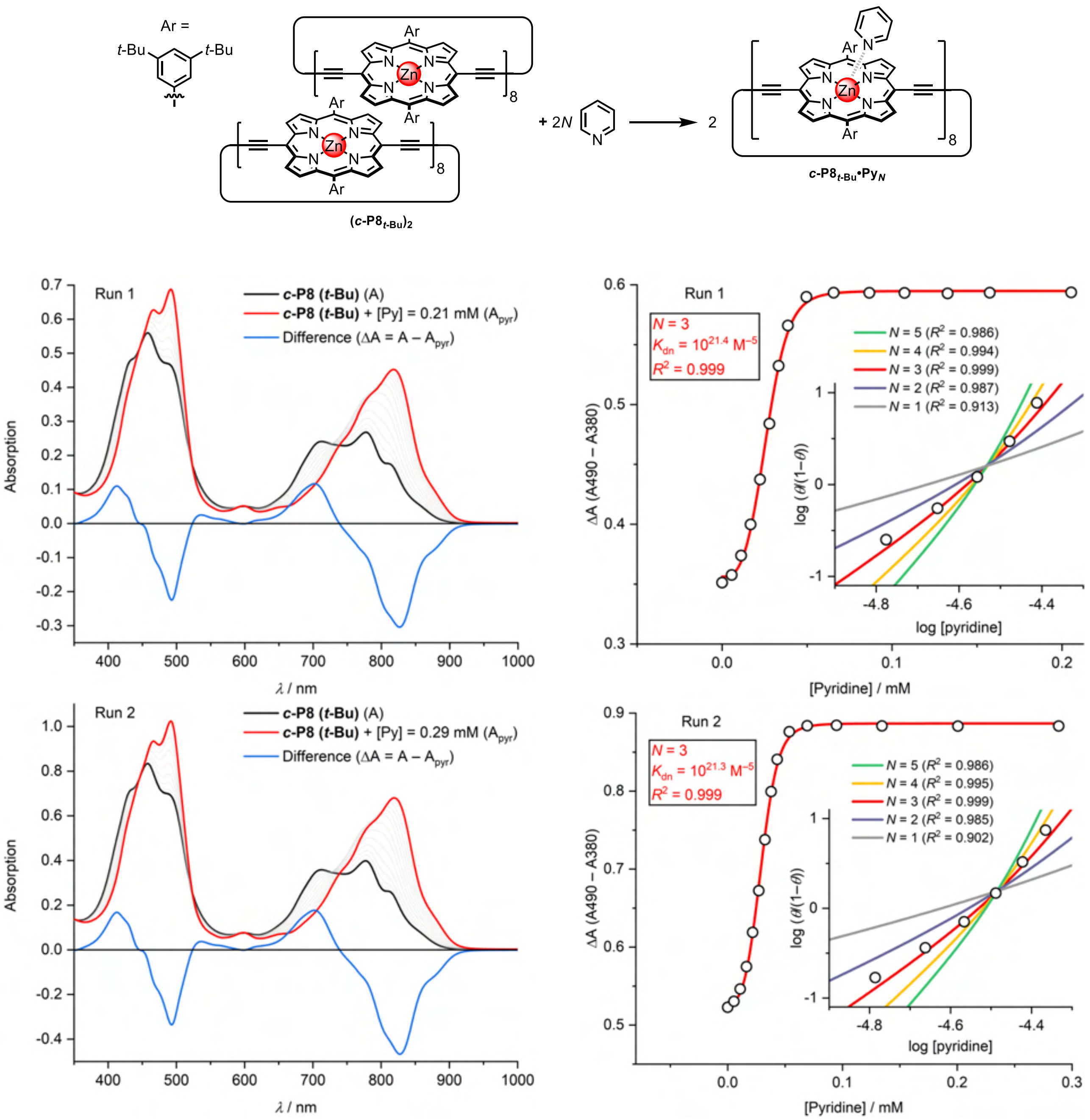

Figure S99. UV-Vis denaturation titrations of **(*c*-P8$_{t\text{-Bu}}$)$_2$** with pyridine in $CDCl_3$ at 25 °C. Absorption spectra and denaturation isotherm including Hill plots as the inset are shown for two runs 1 and 2. The changes observed in the Soret band region where fitted to a 1-to-2 denaturation model with $N = 3$ resulting in the best fit. Run 1: ([***c*-P8**] = 7.99 × 10^{-7} M, $K_{dn} = 10^{21.4}$ M^{-5}, $N = 3$, $R^2 = 0.99866$). Best Hill coefficient: $n_H = 3.5$. Run 2: ([***c*-P8**] = 1.19 × 10^{-6} M, $K_{dn} = 10^{21.3}$ M^{-5}, $N = 3$, $R^2 = 0.99883$). Average K_{dn} is = $10^{21.35}$ M^{-5}. Best Hill coefficient: $n_H = 3.9$.

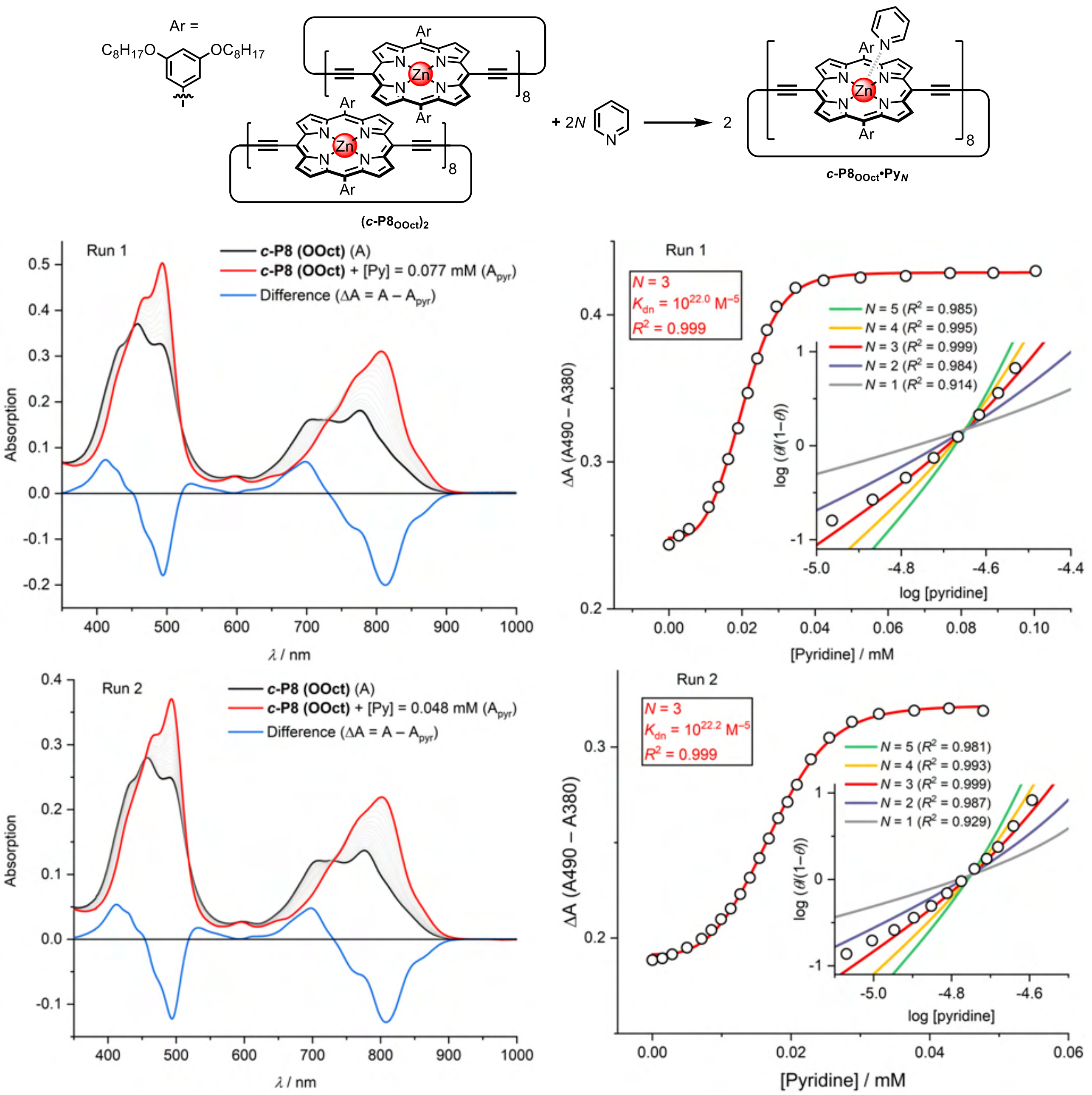

Figure S100. UV-Vis denaturation titrations of **(*c*-P8$_{\mathbf{OOct}}$)$_2$** with pyridine in $CDCl_3$ at 25 °C. Absorption spectra and denaturation isotherm including Hill plots as the inset are shown for two runs 1 and 2. The changes observed in the Soret band region where fitted to a 1-to-2 denaturation model with $N = 3$ resulting in the best fit. Run 1: ([***c*-P8**] = 7.28 × 10^{-7} M, $K_{dn} = 10^{22.0}$ M^{-5}, $N = 3$, $R^2 = 0.99872$). Best Hill coefficient: $n_H = 3.9$. Run 2: ([***c*-P8**] = 4.37 × 10^{-7} M, $K_{dn} = 10^{22.2}$ M^{-5}, $N = 3$, $R^2 = 0.99899$). Average K_{dn} is = $10^{22.1}$ M^{-5}. Best Hill coefficient: $n_H = 3.9$.

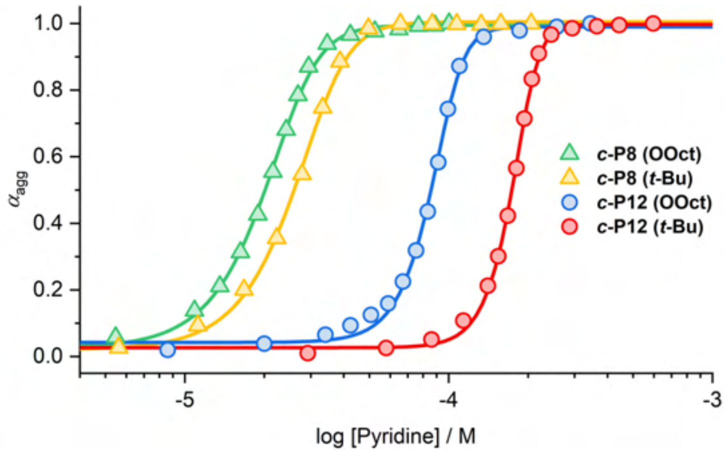

Figure S101. Extent of aggregation as a function of pyridine concentration for **(*c*-P8$_{OOct}$)$_2$** (run 1), **(*c*-P8$_{t\text{-Bu}}$)$_2$** (run 1), **(*c*-P12$_{OOct}$)$_2$** (run 1), and **(*c*-P12$_{t\text{-Bu}}$)$_2$** (run 1) in $CDCl_3$ at 25 °C as given by the change in the Soret band region (***c*-P8**: ΔA = 490–380 nm; ***c*-P12**: ΔA = 470–380 nm). Fits to a 1-to-2 denaturation model is included for each aggregate: **(*c*-P8$_{OOct}$)$_2$** (N = 3), **(*c*-P12$_{t\text{-Bu}}$)$_2$** (N = 3), **(*c*-P12$_{OOct}$)$_2$** (N = 5), and **(*c*-P12$_{t\text{-Bu}}$)$_2$** (N = 6).

Table S19. Summary of parameters from pyridine denaturation experiments on dimers of ***c*-P8$_{OOct}$**, ***c*-P8$_{t\text{-Bu}}$**, ***c*-P12$_{OOct}$**, and ***c*-P12$_{t\text{-Bu}}$** in $CHCl_3$ at 25 °C.

Ring	Run	c_{ring} / M	N_{fit} (N_{max})	K_{dn}	n_H ($n_{H,\,max}$)	$c_{pyr,50}$ / M
**(*c*-P8$_{OOct}$)$_2$**	1	$7.28{\cdot}10^{-7}$	3 (8)	$10^{22.0}$ M^{-5}	3.9 (8)	$2.04{\cdot}10^{-5}$
**(*c*-P8$_{OOct}$)$_2$**	2	$4.37{\cdot}10^{-7}$	3 (8)	$10^{22.2}$ M^{-5}	3.9 (8)	$1.73{\cdot}10^{-5}$
**(*c*-P8$_{t\text{-Bu}}$)$_2$**	1	$7.99{\cdot}10^{-7}$	3 (8)	$10^{21.4}$ M^{-5}	3.5 (8)	$2.65{\cdot}10^{-5}$
**(*c*-P8$_{t\text{-Bu}}$)$_2$**	2	$1.19{\cdot}10^{-6}$	3 (8)	$10^{21.3}$ M^{-5}	3.9 (8)	$3.00{\cdot}10^{-5}$
**(*c*-P12$_{OOct}$)$_2$**	1	$5.26{\cdot}10^{-7}$	5 (12)	$10^{33.8}$ M^{-9}	6.6 (12)	$9.89{\cdot}10^{-5}$
**(*c*-P12$_{OOct}$)$_2$**	2	$3.38{\cdot}10^{-7}$	5 (12)	$10^{34.1}$ M^{-9}	6.5 (12)	$8.76{\cdot}10^{-5}$
**(*c*-P12$_{t\text{-Bu}}$)$_2$**	1	$5.00{\cdot}10^{-7}$	6 (12)	$10^{38.8}$ M^{-11}	7.7 (12)	$1.76{\cdot}10^{-4}$
**(*c*-P12$_{t\text{-Bu}}$)$_2$**	2	$5.00{\cdot}10^{-7}$	6 (12)	$10^{38.9}$ M^{-11}	7.4 (12)	$1.74{\cdot}10^{-4}$

One-to-one binding model

The starting state (A) can react with *N* equivalents of pyridine and is converted into the final state, the coordinated state of A (A•Py):

$$A + N \cdot Py \rightarrow A \bullet Py_N$$

If the final state, for convenience, instead is abbreviated as B, the equilibrium can be shortened to:

$$A + N \cdot Py \rightarrow B$$

The expression for the associated equilibrium constant is:

$$K = \frac{[B]}{[A] \cdot [Py]^N}$$

$$0 = [B] - K \cdot [A] \cdot [Py]^N$$

The concentration of A, can be expressed using the starting concentration of A (c_0):

$$[A] = c_0 - [B]$$

Using that, the expression including the equilibrium constant, can be expanded and solved for the final state:

$$0 = [B] - K \cdot (c_0 - [B]) \cdot [Py]^N$$

$$0 = [B] - K \cdot c_0 \cdot [Py]^N + K \cdot [B] \cdot [Py]^N$$

$$[B] \cdot (1 + K \cdot [Py]^N) = K \cdot c_0 \cdot [Py]^N$$

$$[B] = \frac{K \cdot c_0 \cdot [Py]^N}{1 + K \cdot [Py]^N}$$

During a UV-vis titration, the denaturation is followed by the change in UV as a result of progressing from the initial state towards the final state. This progression can be expressed using the ratio of the change in absorption over the change in absorption between the final and initial states:

$$\frac{A - A_0}{A_f - A_0}$$

$$A = \varepsilon_A \cdot [A] + \varepsilon_B \cdot [B] = \varepsilon_A \cdot (c_0 - [B]) + \varepsilon_B \cdot [B]$$

$$A_0 = \varepsilon_A \cdot [A]_0 = \varepsilon_A \cdot c_0$$

$$A_f = \varepsilon_B \cdot [B]_f = \varepsilon_B \cdot c_0$$

$$\frac{A - A_0}{A_f - A_0} = \frac{(\varepsilon_A \cdot (c_0 - [B]) + \varepsilon_B \cdot [B]) - (\varepsilon_A \cdot c_0)}{(\varepsilon_B \cdot c_0) - (\varepsilon_A \cdot c_0)} = \frac{(\varepsilon_B - \varepsilon_A) \cdot [B]}{(\varepsilon_B - \varepsilon_A) \cdot c_0} = \frac{[B]}{c_0}$$

If re-written in terms of the absorption (A), this becomes:

$$A = \frac{[B]}{c_0} \cdot \left(A_f - A_0\right) + A_0$$

The final expression of absorption (A) as a function of the competing ligand (Py) used for fitting, appears when the expression for [B] from above is included:

$$A = \left(\frac{K \cdot c_0 \cdot [Py]^N}{1 + K \cdot [Py]^N}\right) \frac{1}{c_0} \cdot \left(A_f - A_0\right) + A_0$$

$$A = \left(\frac{K \cdot [Py]^N}{1 + K \cdot [Py]^N}\right) \cdot \left(A_f - A_0\right) + A_0$$

One-to-two denaturation model

The starting state can be dissociated by $2N$ equivalents of pyridine into the final state as shown:

$$A_2 + 2N \cdot Py \rightarrow 2A \bullet Py_N$$

If the final state ($A \bullet Py_N$), for convenience, instead is abbreviated as B, the equilibrium can be shortened to:

$$A_2 + 2N \cdot Py \rightarrow 2B$$

The expression for the associated equilibrium constant is given as:

$$K = \frac{[B]^2}{[A_2] \cdot [Py]^{2N}}$$

$$0 = [B]^2 - K \cdot [A_2] \cdot [Py]^{2N}$$

The concentration of A_2, can be expressed using the starting concentration of A (c_0):

$$[A_2] = \frac{1}{2}c_0 - \frac{1}{2}[B] = \frac{1}{2}(c_0 - [B])$$

If this is replaced instead of $[A_2]$, the equilibrium expression can be rewritten as:

$$0 = [B]^2 - K \cdot \left(\frac{1}{2}(c_0 - [B])\right) \cdot [Py]^{2N}$$

$$0 = [B]^2 - \frac{1}{2} \cdot K \cdot c_0 \cdot [Py]^{2N} + \frac{1}{2} \cdot K \cdot [B] \cdot [Py]^{2N}$$

$$0 = [B]^2 + \frac{1}{2} \cdot K \cdot [B] \cdot [Py]^{2N} - \frac{1}{2} \cdot K \cdot c_0 \cdot [Py]^{2N}$$

The latter is a quadratic equation ($a \cdot x^2 + b \cdot x + c$) with the following coefficients and root for [B]:

$$a = 1$$

$$b = \frac{1}{2} \cdot K \cdot [Py]^{2N}$$

$$c = -\frac{1}{2} \cdot K \cdot c_0 \cdot [Py]^{2N}$$

$$[B] = \frac{-b \pm \sqrt{b^2 - 4ac}}{2a}$$

Since only positive values of [B] are expected, it is only necessary to consider the expression with a positive contribution from the square-root term:

$$[B] = \frac{-b + \sqrt{b^2 - 4ac}}{2a}$$

$$[B] = \frac{-\left(\frac{1}{2} \cdot K \cdot [Py]^{2N}\right) + \sqrt{\left(\frac{1}{2} \cdot K \cdot [Py]^{2N}\right)^2 - 4 \cdot (1) \cdot \left(-\frac{1}{2} \cdot K \cdot c_0 \cdot [Py]^{2N}\right)}}{2 \cdot (1)}$$

$$[B] = \frac{-\frac{1}{2} \cdot K \cdot [Py]^{2N} + \sqrt{\frac{1}{4} \cdot K^2 \cdot [Py]^{4N} + 2 \cdot K \cdot c_0 \cdot [Py]^{2N}}}{2}$$

During a UV-vis titration, the denaturation is followed by the change in UV as a result of progressing from the initial state towards the final state. This progression can be expressed using the ratio of the change in absorption over the change in absorption between the final and initial states:

$$\frac{A - A_0}{A_f - A_0}$$

$$A = \varepsilon_{A_2} \cdot [A_2] + \varepsilon_B \cdot [B] = \varepsilon_{A_2} \cdot \left(\frac{1}{2}(c_0 - [B])\right) + \varepsilon_B \cdot [B]$$

$$A_0 = \varepsilon_{A_2} \cdot [A]_0 = \varepsilon_{A_2} \cdot \left(\frac{1}{2} \cdot c_0\right)$$

$$A_f = \varepsilon_B \cdot [B]_f = \varepsilon_B \cdot (c_0)$$

$$\frac{A - A_0}{A_f - A_0} = \frac{\varepsilon_{A_2} \cdot \left(\frac{1}{2}(c_0 - [B])\right) + \varepsilon_B \cdot [B] - \varepsilon_{A_2} \cdot \left(\frac{1}{2} \cdot c_0\right)}{\varepsilon_B \cdot (c_0) - \varepsilon_{A_2} \cdot \left(\frac{1}{2} \cdot c_0\right)} = \frac{\left(\varepsilon_B - \frac{1}{2}\varepsilon_{A_2}\right) \cdot [B]}{\left(\varepsilon_B - \frac{1}{2}\varepsilon_{A_2}\right) \cdot c_0} = \frac{[B]}{c_0}$$

If re-written in terms of the absorption (A), this becomes:

$$A = \frac{[B]}{c_0} \cdot \left(A_f - A_0\right) + A_0$$

The final expression of absorption (A) as a function of the competing ligand (Py), appears when the expression for [B] from above is included:

$$A = \frac{-\frac{1}{2} \cdot K \cdot [Py]^{2N} + \sqrt{\frac{1}{4} \cdot K^2 \cdot [Py]^{4N} + 2 \cdot K \cdot c_0 \cdot [Py]^{2N}}}{2} \cdot \frac{1}{c_0} \cdot \left(A_f - A_0\right) + A_0$$

$$A = \frac{-\frac{1}{2} \cdot K \cdot [Py]^{2N} + \sqrt{\frac{1}{4} \cdot K^2 \cdot [Py]^{4N} + 2 \cdot K \cdot c_0 \cdot [Py]^{2N}}}{2 \cdot c_0} \cdot \left(A_f - A_0\right) + A_0$$

For fitting purposes, *K* was raised to the power of 10 so that the values of *K* to be fitted would be smaller. The following is the equation used during fitting:

$$A = \frac{-\frac{1}{2} \cdot 10^K \cdot [Py]^{2N} + \sqrt{\frac{1}{4} \cdot 10^{2K} \cdot [Py]^{4N} + 2 \cdot 10^K \cdot c_0 \cdot [Py]^{2N}}}{2 \cdot c_0} \cdot \left(A_f - A_0\right) + A_0$$

Therefore, the actual *K* for the denaturation process has to be re-calculated after fitting as:

$$K_{actual} = 10^K$$

Section 17. Diffusion-Ordered ^{1}H NMR Experiments on Nanorings

Table S20. Summary of diffusion coefficients of ***c*-P6**, ***c*-P8**, and ***c*-P12** rings with 3,5-bis(octyloxy)phenyl (OOct), 3,5-bis(*tert*-butyl)phenyl (*t*-Bu), and 3,5-bis(trihexylsilyl)phenyl (THS) sidechains in absence (top) and presence (bottom) of pyridine-d_5.

In the absence of pyridine-d_5:			
**OOct:**	***c*-P6**	***c*-P8**	***c*-P12**
**State**	monomeric/non-discrete	dimeric	dimeric
***D* / m^2 s^{-1}**	n.d.[a]	$1.73 \cdot 10^{-10}$	$1.47 \cdot 10^{-10}$
***t*-Bu:**	***c*-P6**	***c*-P8**	***c*-P12**
**State**	monomeric	dimeric	dimeric
***D* / m^2 s^{-1}**	$2.77 \cdot 10^{-1}$	$1.89 \cdot 10^{-10}$	$1.45 \cdot 10^{-10}$
**THS:**	***c*-P6**	***c*-P8**	***c*-P12**
**State**	monomeric	monomeric	monomeric
***D* / m^2 s^{-1}**	$2.06 \cdot 10^{-10}$	$1.78 \cdot 10^{-10}$	$1.37 \cdot 10^{-10}$
In the presence of pyridine-d_5:			
**OOct:**	***c*-P6**	***c*-P8**	***c*-P12**
**State**	monomeric	monomeric	monomeric
***D* / m^2 s^{-1}**	$2.15 \cdot 10^{-10}$	$2.04 \cdot 10^{-10}$	$1.38 \cdot 10^{-10}$
***t*-Bu:**	***c*-P6**	***c*-P8**	***c*-P12**
**State**	monomeric	monomeric	monomeric
***D* / m^2 s^{-1}**	$2.50 \cdot 10^{-10}$	$1.79 \cdot 10^{-10}$	$1.62 \cdot 10^{-10}$
**THS:**	***c*-P6**	***c*-P8**	***c*-P12**
**State**	monomeric	monomeric	monomeric
***D* / m^2 s^{-1}**	$1.98 \cdot 10^{-10}$	$1.70 \cdot 10^{-10}$	$1.38 \cdot 10^{-10}$

[a]Not determined because proton resonances decayed too rapidly to record meaningful diffusion spectra.

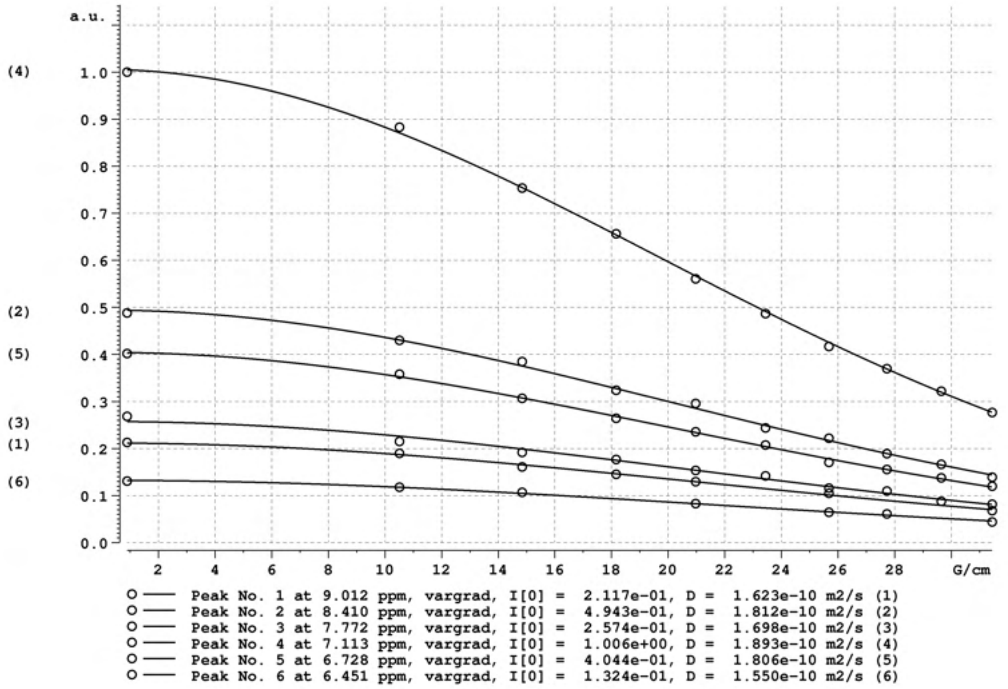

Figure S102. ^{1}H diffusion decay plots (500 MHz, $CDCl_3$, 298 K) of ***c*-P8$_{\mathbf{OOct}}$** based on signal intensity fitted to the Stejskal Tanner equation and the resulting diffusion coefficients obtained. Acquisition parameters: G = 0.90–40.58 G/cm, Δ = 0.1 s, and δ = 1600 μs. The average diffusion coefficient obtained is $D = 1.73 \cdot 10^{-10}$ m^{2}s^{-1}.

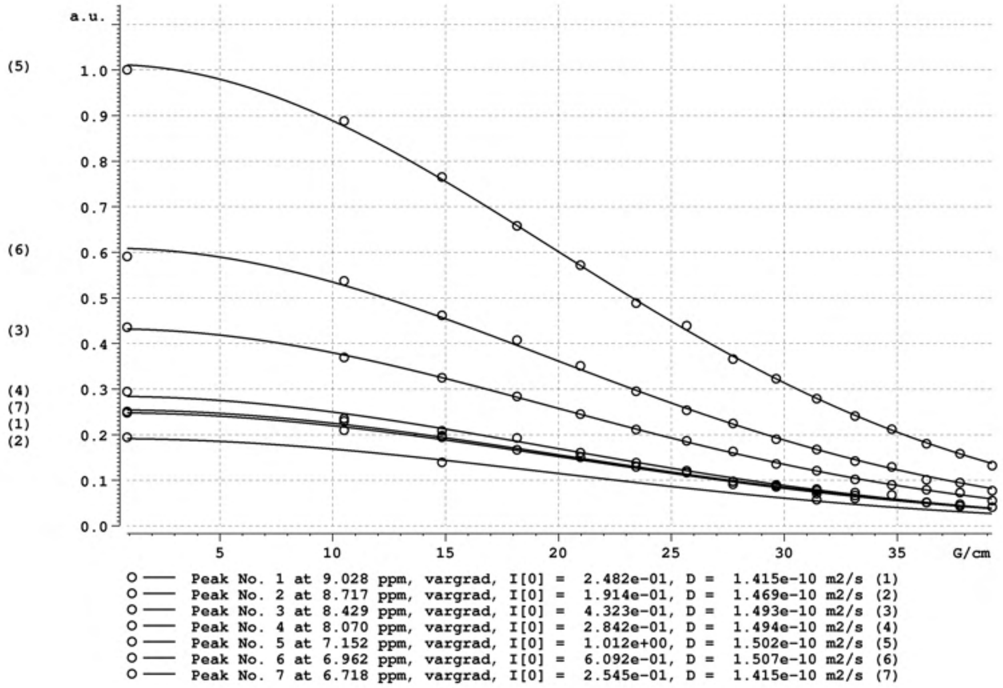

Figure S103. ^{1}H diffusion decay plots (500 MHz, $CDCl_3$, 298 K) of ***c*-P12$_{\mathbf{OOct}}$** based on signal intensity fitted to the Stejskal Tanner equation and the resulting diffusion coefficients obtained. Acquisition parameters: G = 0.90–37.78 G/cm, Δ = 0.1 s, and δ = 1800 μs. The average diffusion coefficient obtained is $D = 1.47 \cdot 10^{-10}$ m^2s^{-1}.

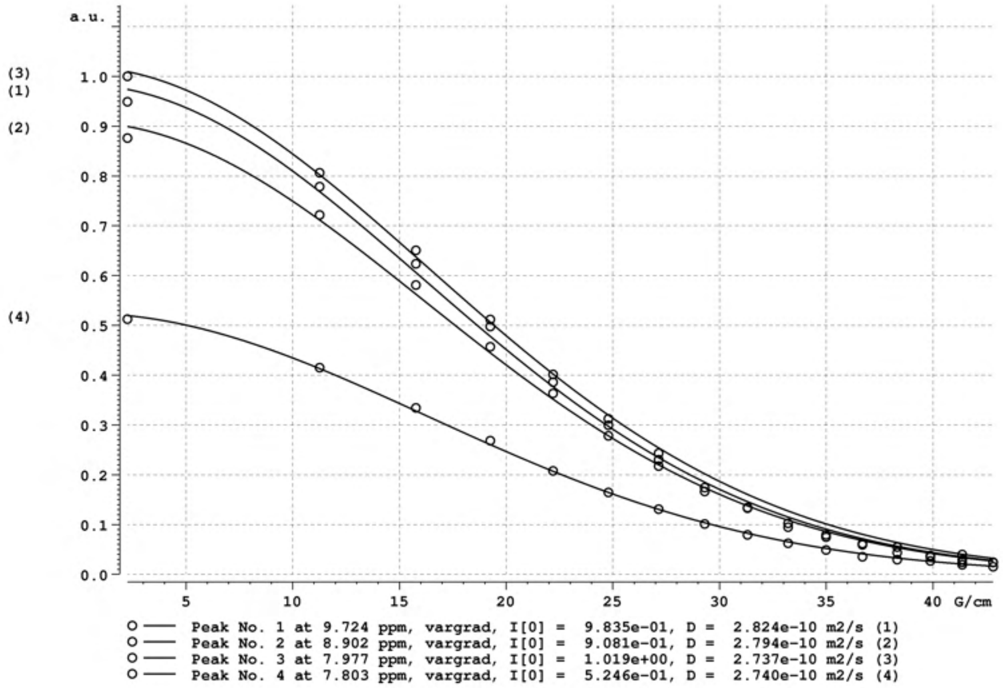

Figure S104. ^{1}H diffusion decay plots (500 MHz, $CDCl_3$, 298 K) of ***c*-P6$_{\boldsymbol{t}\text{-}\mathbf{Bu}}$** based on signal intensity fitted to the Stejskal Tanner equation and the resulting diffusion coefficients obtained. Acquisition parameters: G = 2.25–42.83 G/cm, Δ = 0.1 s, and δ = 1600 μs. The average diffusion coefficient obtained is $D = 2.77 \cdot 10^{-10}$ m^2s^{-1}.

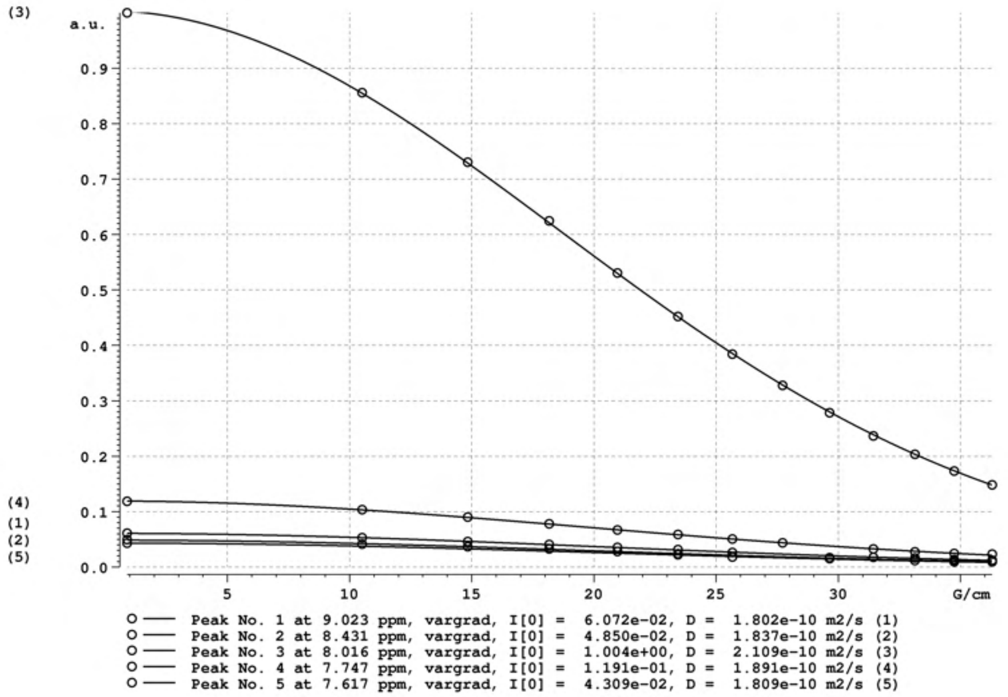

Figure S105. ^{1}H diffusion decay plots (500 MHz, $CDCl_3$, 298 K) of ***c*-P8$_{t\text{-Bu}}$** based on signal fitted to the Stejskal Tanner equation and the resulting diffusion coefficients obtained. Acquisition parameters: G = 0.90–40.58 G/cm, Δ = 0.1 s, and δ = 1600 μs. The average diffusion coefficient obtained is $D = 1.89{\cdot}10^{-10}$ m^{2}s^{-1}.

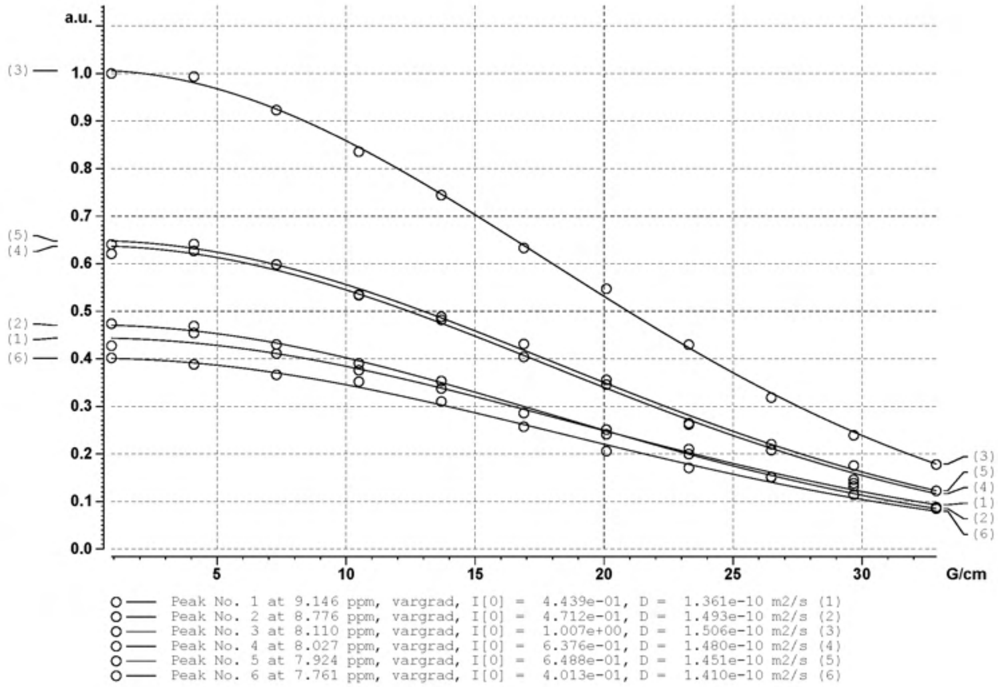

Figure S106. ^{1}H diffusion decay plots (500 MHz, $CDCl_3$, 298 K) of ***c*-P12$_{t\text{-Bu}}$** based on signal fitted to the Stejskal Tanner equation and the resulting diffusion coefficients obtained. Acquisition parameters: G = 2.25–42.83 G/cm, Δ = 0.1 s, and δ = 2000 μs. The average diffusion coefficient obtained is $D = 1.45{\cdot}10^{-10}$ m^{2}s^{-1}.

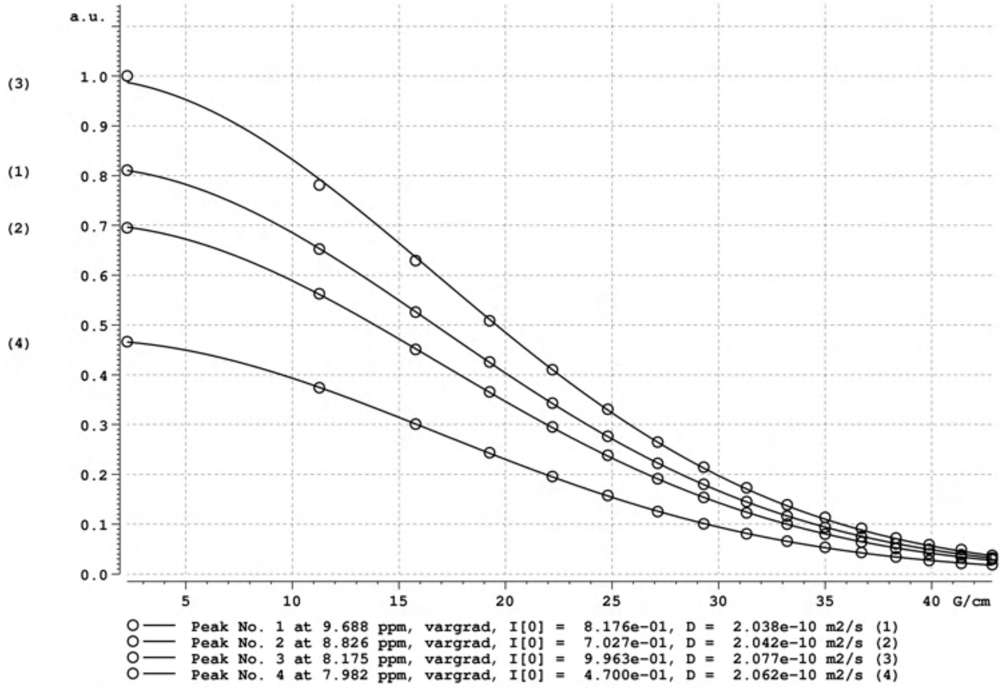

Figure S107. ^{1}H diffusion decay plots (500 MHz, $CDCl_3$, 298 K) of ***c*-P6$_{THS}$** based on signal fitted to the Stejskal Tanner equation and the resulting diffusion coefficients obtained. Acquisition parameters: G = 2.25–42.83 G/cm, Δ = 0.1 s, and δ = 1800 µs. The average diffusion coefficient obtained is $D = 2.06 \cdot 10^{-10}$ m^2s^{-1}.

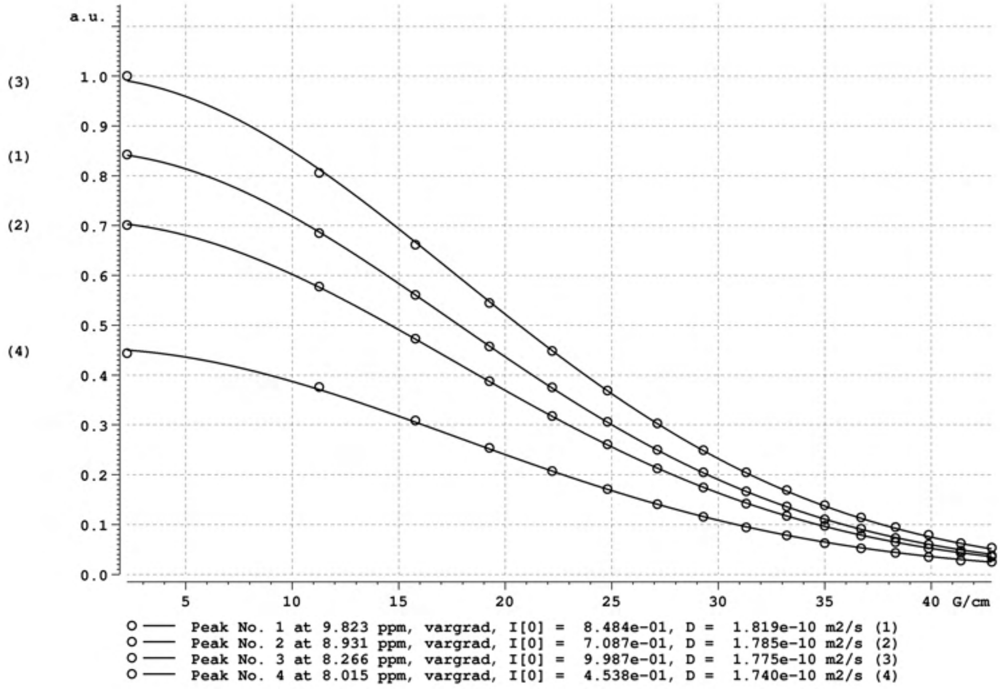

Figure S108. ^{1}H diffusion decay plots (500 MHz, $CDCl_3$, 298 K) of ***c*-P8$_{THS}$** based on signal intensity fitted to the Stejskal Tanner equation and the resulting diffusion coefficients obtained. Acquisition parameters: G = 2.25–42.83 G/cm, Δ = 0.1 s, and δ = 1850 µs. The average diffusion coefficient obtained is $D = 1.78 \cdot 10^{-10}$ m^2s^{-1}.

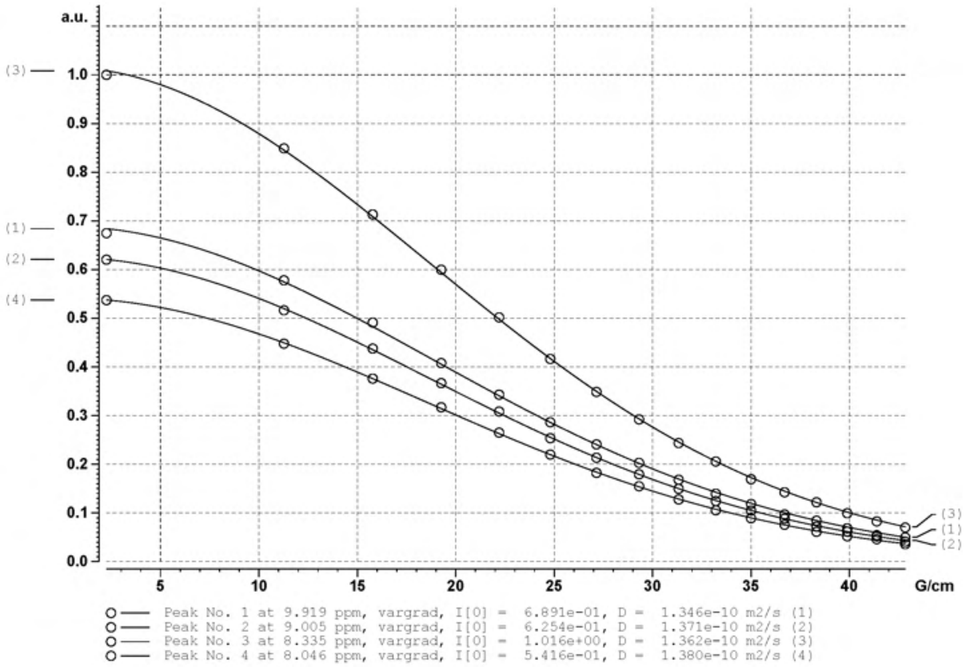

Figure S109. ^{1}H diffusion decay plots (500 MHz, $CDCl_3$, 298 K) of ***c*-P12$_{\mathbf{THS}}$** based on signal intensity fitted to the Stejskal Tanner equation and the resulting diffusion coefficients obtained. Acquisition parameters: G = 2.25–42.83 G/cm, Δ = 0.1 s, and δ = 2000 μs. The average diffusion coefficient obtained is $D = 1.37{\cdot}10^{-10}$ m^2s^{-1}.

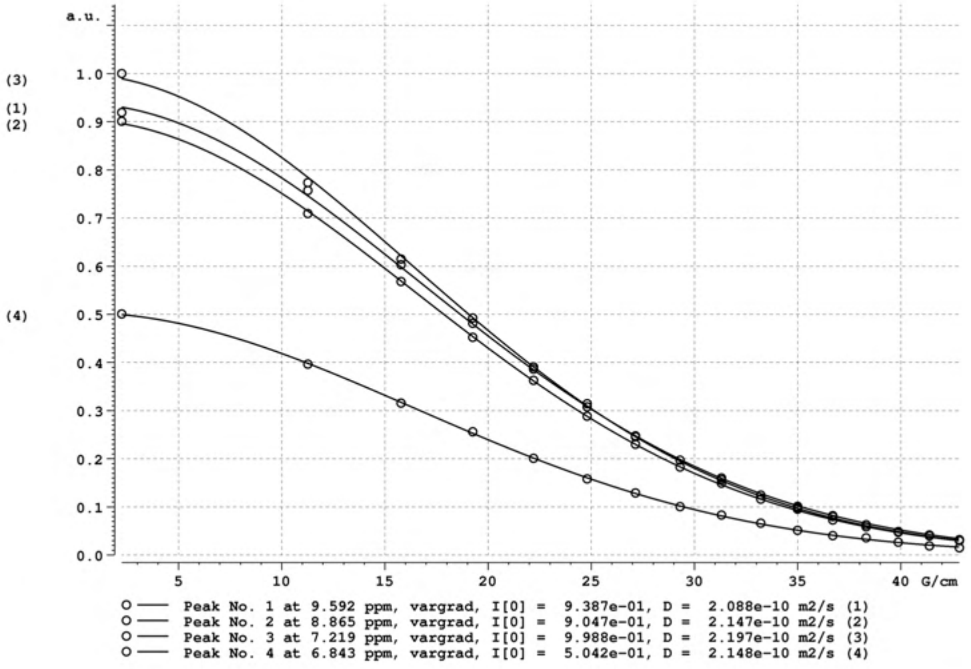

Figure S110. ^{1}H diffusion decay plots (500 MHz, $CDCl_3$, 298 K) of ***c*-P6$_{\mathbf{OOct}}$ + pyridine-*d*$_5$** based on signal intensity fitted to the Stejskal Tanner equation and the resulting diffusion coefficients obtained. Acquisition parameters: G = 2.25–42.83 G/cm, Δ = 0.1 s, and δ = 1800 μs. The average diffusion coefficient obtained is $D = 2.15{\cdot}10^{-10}$ m^2s^{-1}.

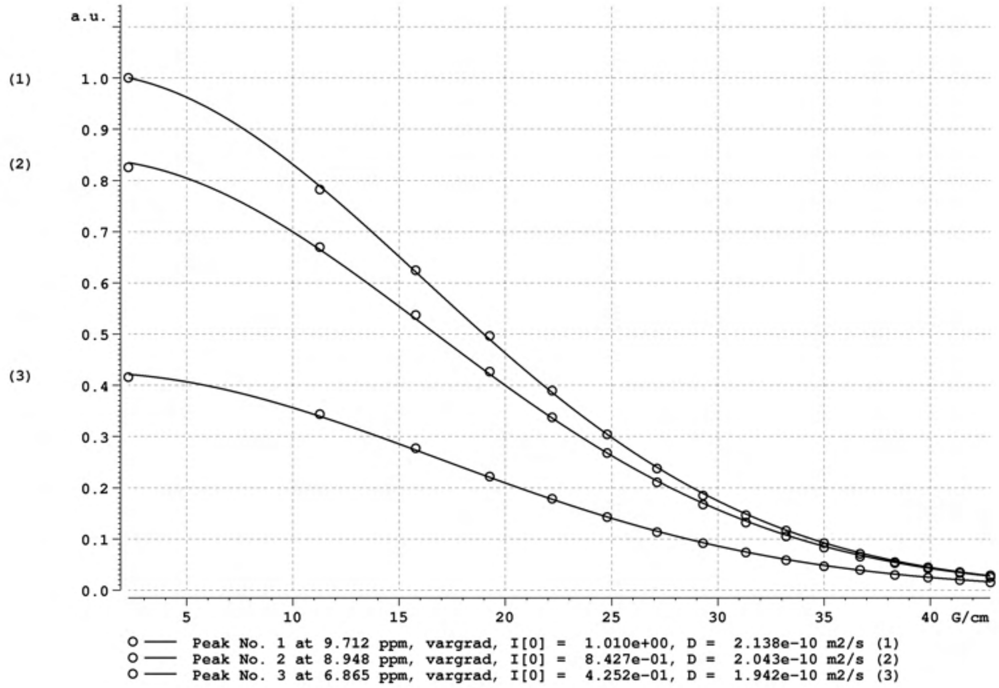

Figure S111. ^{1}H diffusion decay plots (500 MHz, $CDCl_3$, 298 K) of ***c*-P8$_{OOct}$ + pyridine-*d*$_5$** based on signal intensity fitted to the Stejskal Tanner equation and the resulting diffusion coefficients obtained. Acquisition parameters: $G = 2.25–42.83$ G/cm, $\Delta = 0.1$ s, and $\delta = 1850$ µs. The average diffusion coefficient obtained is $D = 2.04 \cdot 10^{-10}$ m^2s^{-1}.

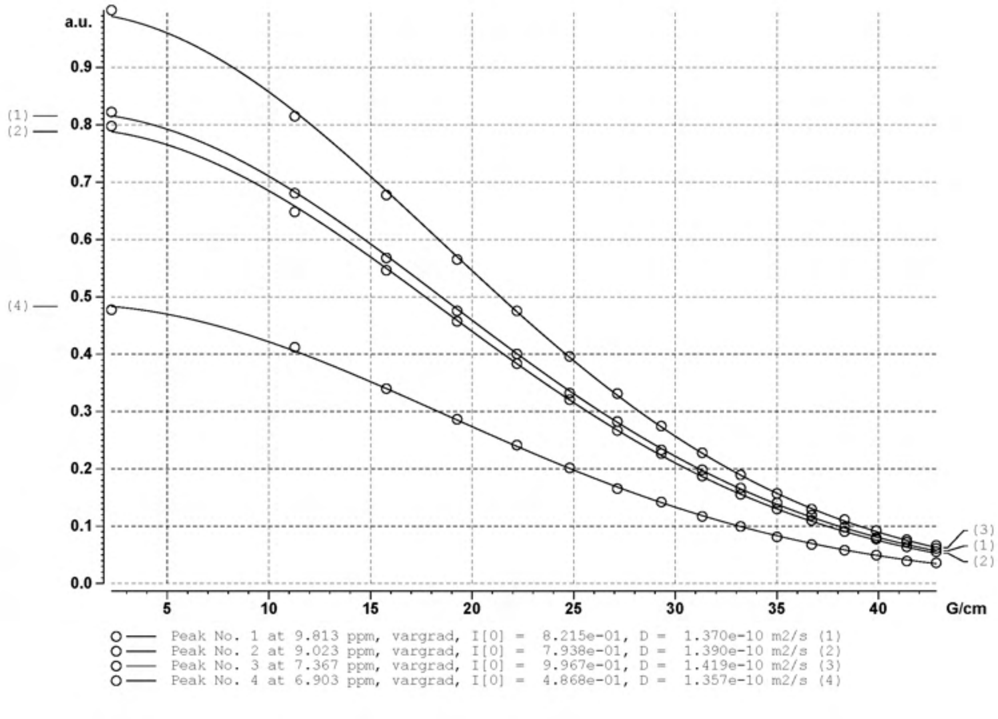

Figure S112. ^{1}H diffusion decay plots (500 MHz, $CDCl_3$, 298 K) of ***c*-P12$_{OOct}$ + pyridine-*d*$_5$** based on signal intensity fitted to the Stejskal Tanner equation and the resulting diffusion coefficients obtained. Acquisition parameters: $G = 2.25–42.83$ G/cm, $\Delta = 0.1$ s, and $\delta = 2000$ µs. The average diffusion coefficient obtained is $D = 1.38 \cdot 10^{-10}$ m^2s^{-1}.

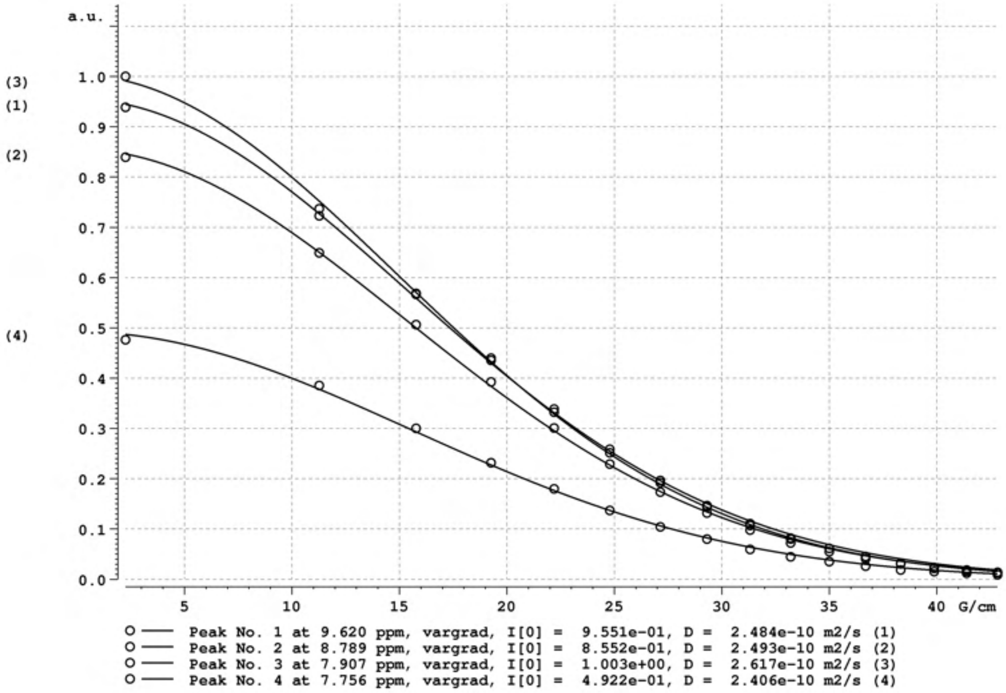

Figure S113. ^{1}H diffusion decay plots (500 MHz, $CDCl_3$, 298 K) of ***c*-P6$_{t\text{-Bu}}$ + pyridine-*d*$_5$** based on signal intensity fitted to the Stejskal Tanner equation and the resulting diffusion coefficients obtained. Acquisition parameters: G = 2.25–42.83 G/cm, Δ = 0.1 s, and δ = 1800 µs. The average diffusion coefficient obtained is D = $2.50 \cdot 10^{-10}$ m^2s^{-1}.

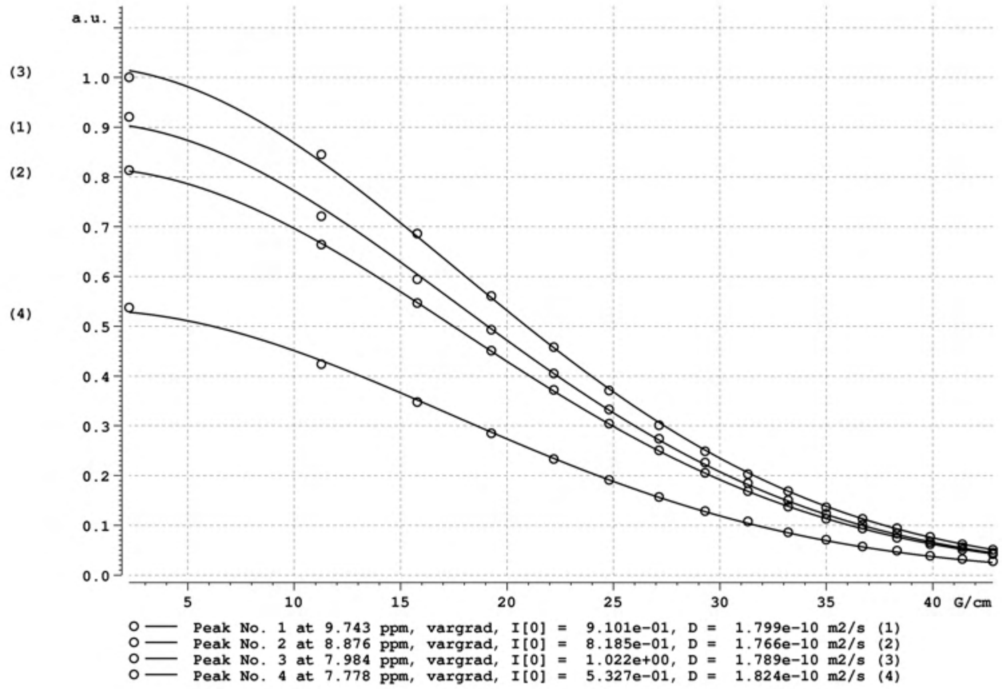

Figure S114. ^{1}H diffusion decay plots (500 MHz, $CDCl_3$, 298 K) of ***c*-P8$_{t\text{-Bu}}$ + pyridine-*d*$_5$** based on signal intensity fitted to the Stejskal Tanner equation and the resulting diffusion coefficients obtained. Acquisition parameters: G = 2.25–42.83 G/cm, Δ = 0.1 s, and δ = 1850 µs. The average diffusion coefficient obtained is D = $1.79 \cdot 10^{-10}$ m^2s^{-1}.

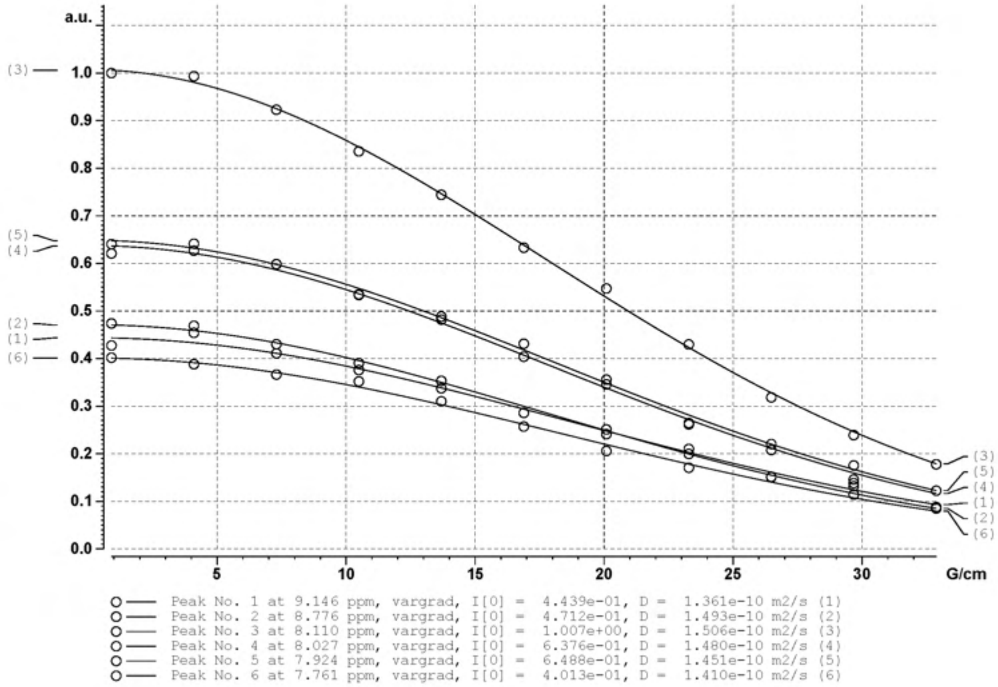

Figure S115. ^{1}H diffusion decay plots (500 MHz, $CDCl_3$, 298 K) of ***c*-P12*ₜ*** $_{t\text{-Bu}}$ **+ pyridine-*d*₅** based on signal intensity fitted to the Stejskal Tanner equation and the resulting diffusion coefficients obtained. Acquisition parameters: G = 2.25–42.83 G/cm, Δ = 0.1 s, and δ = 2000 μs. The average diffusion coefficient obtained is D = $1.62\cdot10^{-10}$ m^2s^{-1}.

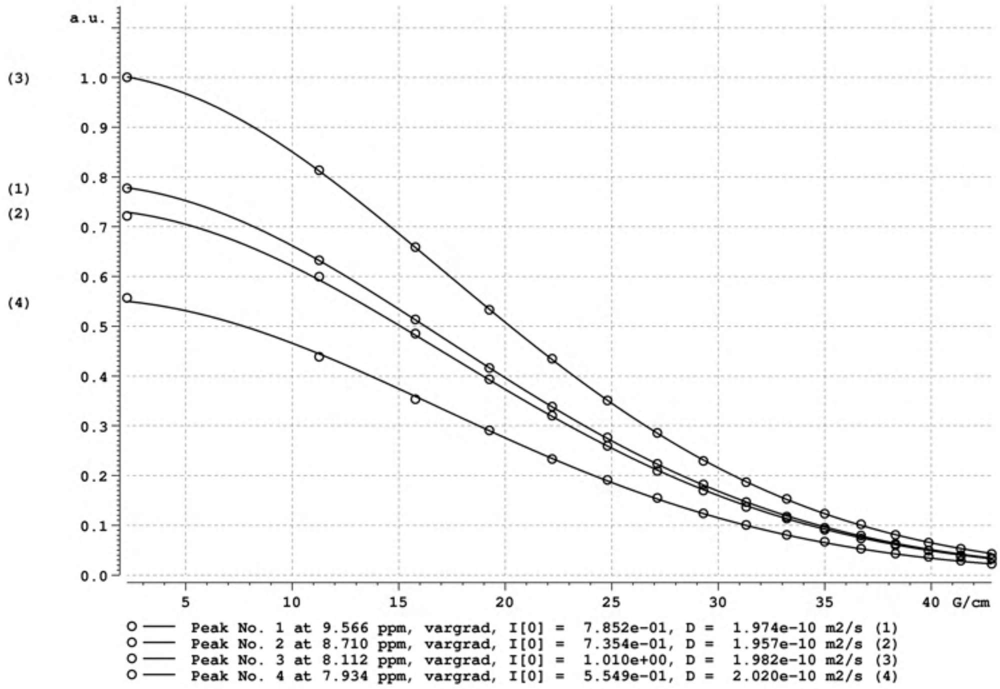

Figure S116. ^{1}H diffusion decay plots (500 MHz, $CDCl_3$, 298 K) of ***c*-P6** $_{\mathbf{THS}}$ **+ pyridine-*d*** $_5$ based on signal intensity fitted to the Stejskal Tanner equation and the resulting diffusion coefficients obtained. Acquisition parameters: G = 2.25–42.83 G/cm, Δ = 0.1 s, and δ = 1800 μs. The average diffusion coefficient obtained is D = $1.98\cdot10^{-10}$ m^2s^{-1}.

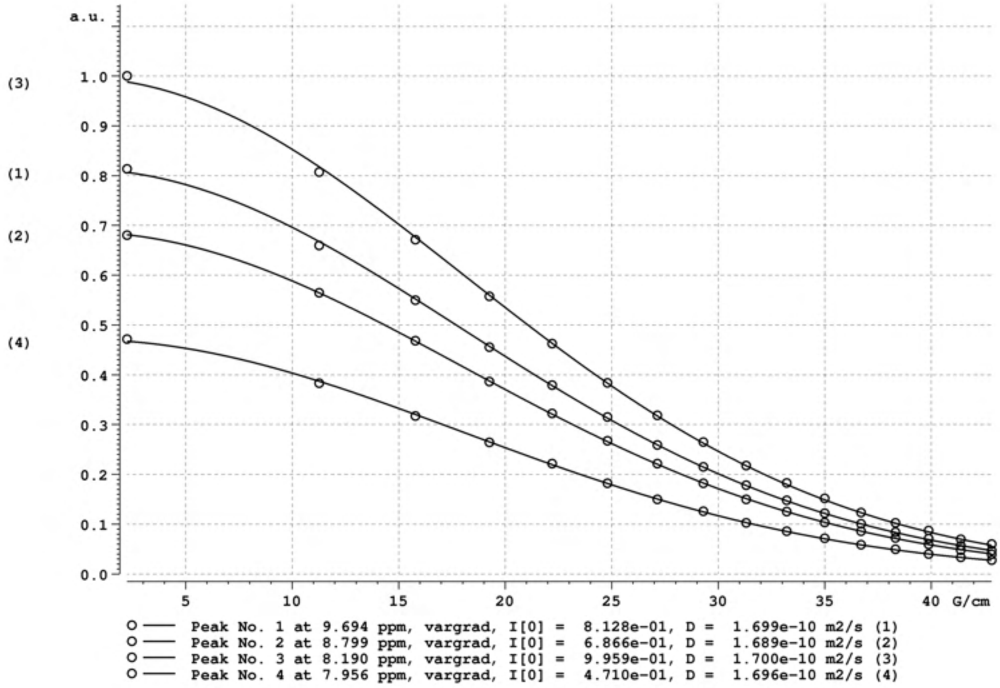

Figure S117. ^{1}H diffusion decay plots (500 MHz, $CDCl_3$, 298 K) of ***c*-P8$_{\mathbf{THS}}$ + pyridine-*d*$_5$** based on signal intensity fitted to the Stejskal Tanner equation and the resulting diffusion coefficients obtained. Acquisition parameters: G = 2.25–42.83 G/cm, Δ = 0.1 s, and δ = 1850 μs. The average diffusion coefficient obtained is D = $1.70 \cdot 10^{-10}$ m^2s^{-1}.

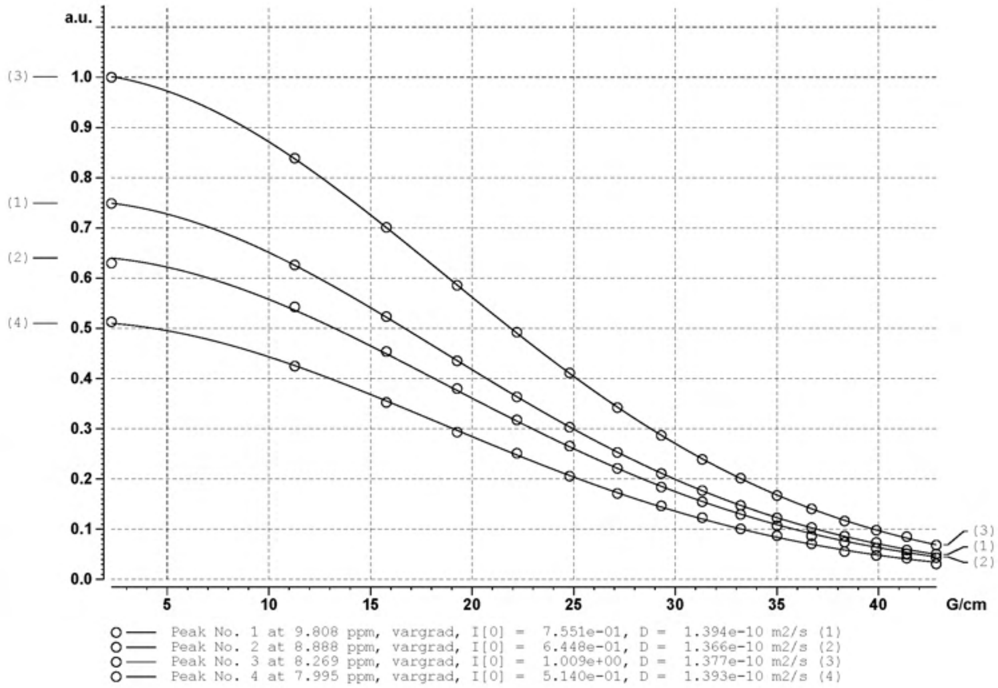

Figure S118. ^{1}H diffusion decay plots (500 MHz, $CDCl_3$, 298 K) of ***c*-P12$_{\mathbf{THS}}$ + pyridine-*d*$_5$** based on signal intensity fitted to the Stejskal Tanner equation and the resulting diffusion coefficients obtained. Acquisition parameters: G = 2.25–42.83 G/cm, Δ = 0.1 s, and δ = 2000 μs. The average diffusion coefficient obtained is D = $1.38 \cdot 10^{-10}$ m^2s^{-1}.

Section 18. NMR Spectra of Nanorings

c-P6$_{t\text{-Bu}}$ + pyridine-d_5

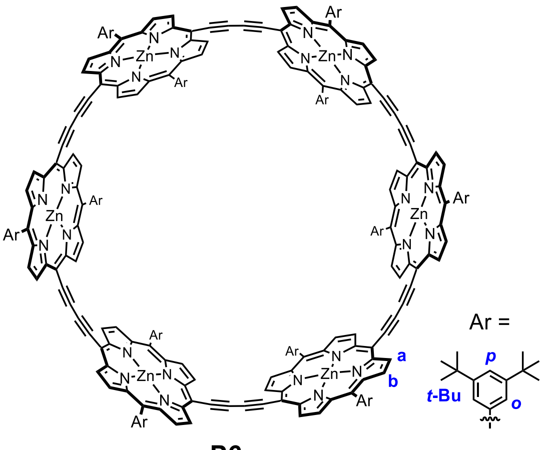

#	Assign.	^{1}H / ppm	Mult.	HSQC
**1**	a	9.60 (4H)	d, *J* = 4.4 Hz	130.2
**2**	b	8.77 (4H)	d, *J* = 4.4 Hz	133.0
**3**	*o*	7.88 (4H)	d, *J* = 1.7 Hz	129.4
**4**	*p*	7.73 (2H)	t, *J* = 1.7 Hz	120.7
**5**	*t*-Bu	1.47 (36H)	s	31.5

Figure S119. Top: Structure of ***c*-P6$_{t\text{-Bu}}$** with labels used for ^{1}H assignment. Bottom: Assigned ^{1}H resonances and ^{13}C chemical shift values for their associated carbon atoms. The number of protons is listed as per porphyrin unit.

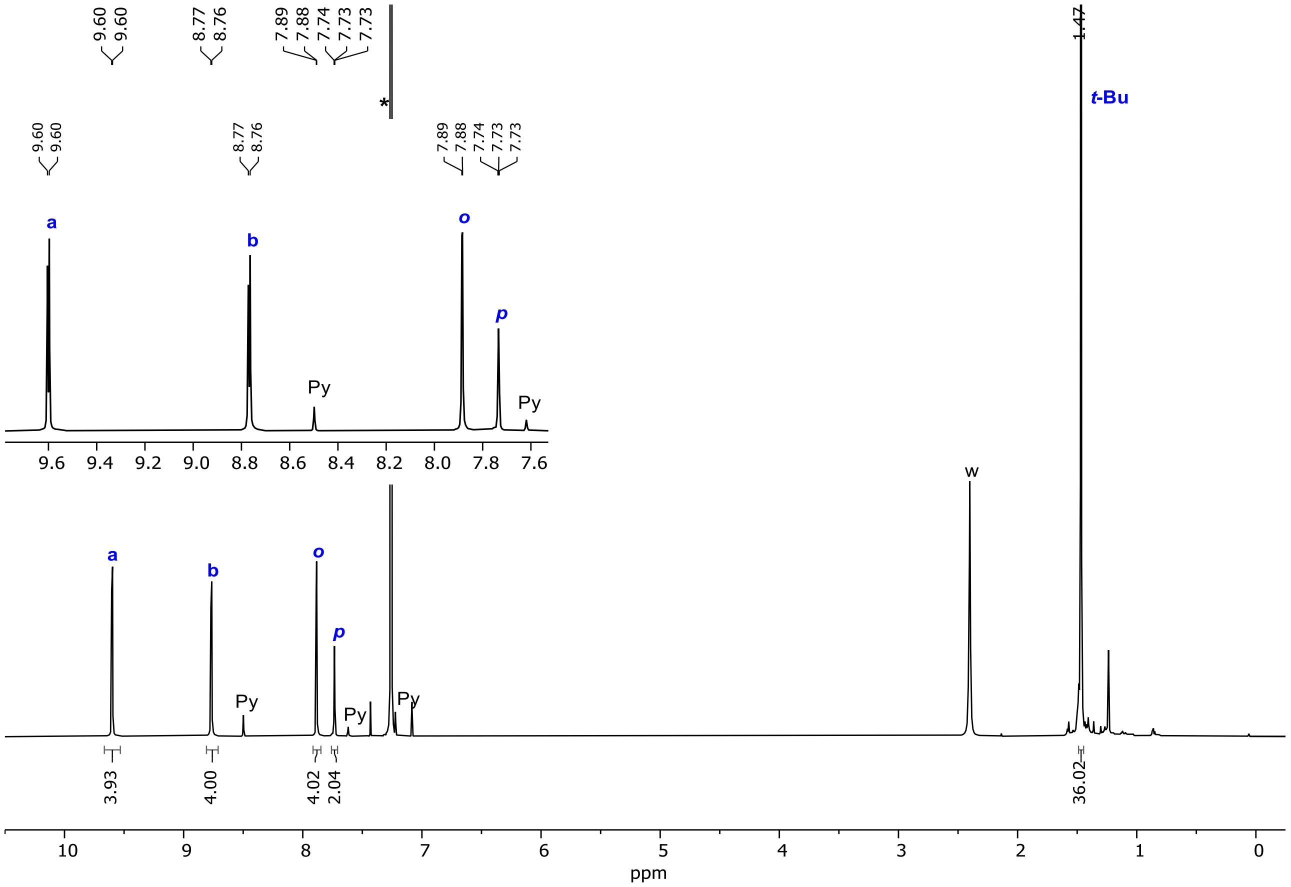

Figure S120. Assigned ^{1}H-NMR spectrum of ***c*-P6$_{t\text{-Bu}}$** (500 MHz, $CDCl_3$ + 5% pyridine-d_5, 298 K). Abbreviations: * = $CHCl_3$, Py = pyridine, w = water.

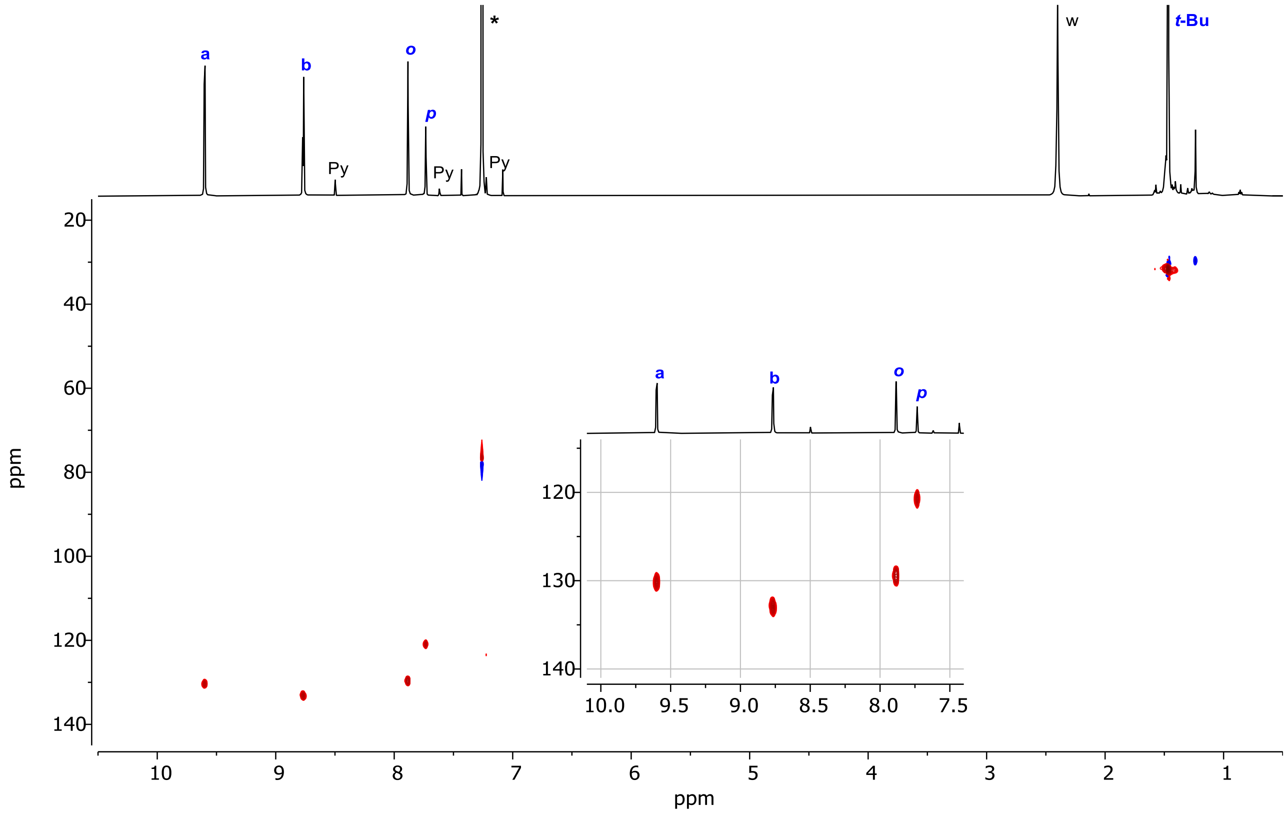

Figure S121. ^{1}H-^{13}C HSQC of ***c*-P6$_{t\text{-Bu}}$** (600 MHz, $CDCl_3$ + 5% pyridine-d_5, 298 K). Abbreviations: * = $CHCl_3$, Py = pyridine, w = water.

c-P6$_{t\text{-Bu}}$ in the absence of pyridine

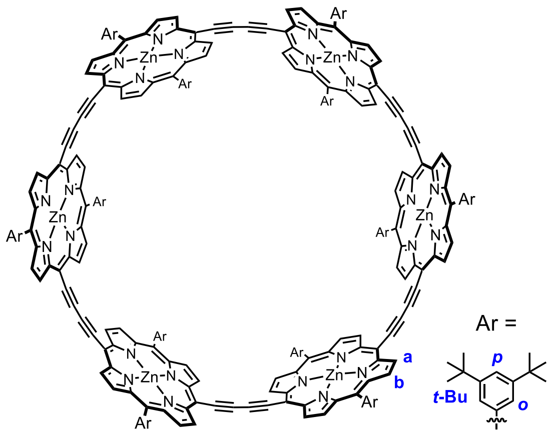

c-P6$_{t\text{-Bu}}$

#	Assign.	^{1}H / ppm	Mult.	HSQC
**1**	a	9.60 (4H)	d, J = 4.5 Hz	130.8
**2**	b	8.77 (4H)	d, J = 4.5 Hz	133.6
**3**	*o*	7.88 (4H)	s	129.6
**4**	*p*	7.73 (2H)	s	121.2
**5**	*t*-Bu	1.47 (36H)	s	31.7

Figure S122. Top: Structure of **c-P6$_{t\text{-Bu}}$** with labels used for ^{1}H assignment. Bottom: Assigned ^{1}H resonances and ^{13}C chemical shift values for their associated carbon atoms. The number of protons is listed as per porphyrin unit.

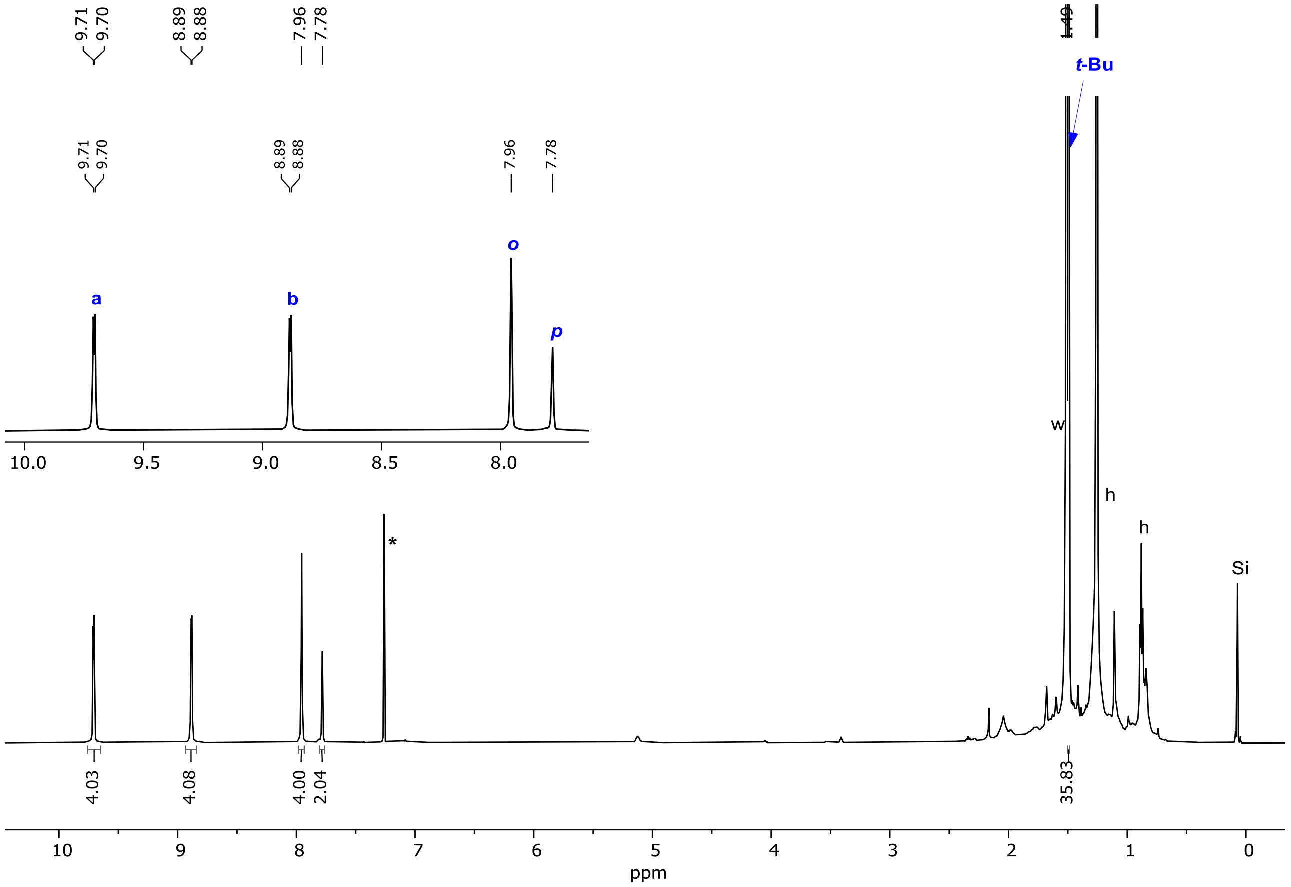

Figure S123. Assigned ^{1}H-NMR spectrum of ***c*-P6$_{t\text{-Bu}}$** (600 MHz, $CDCl_3$, 298 K). Abbreviations: * = $CHCl_3$, w = water, h = H-grease, Si = silicone grease.

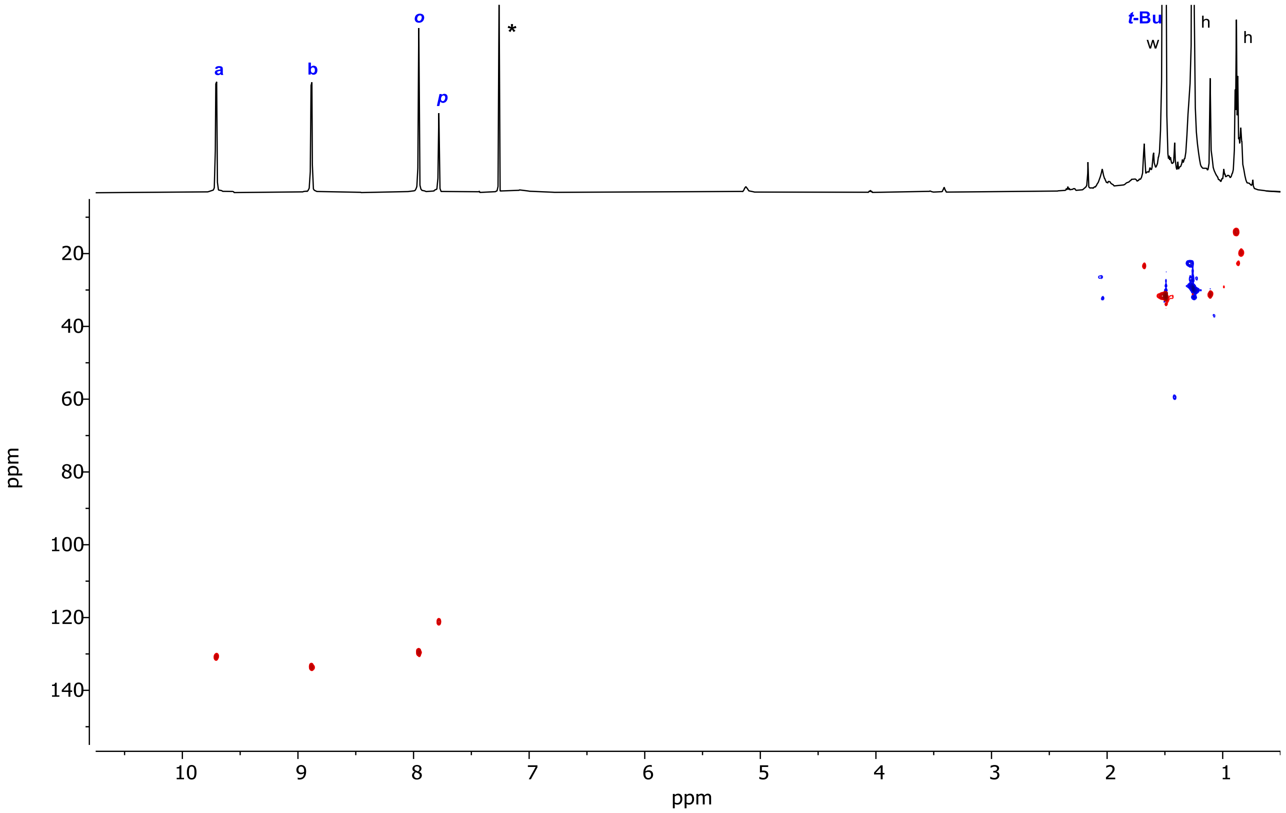

Figure S124. ^{1}H-^{13}C HSQC of ***c*-P6$_{t\text{-Bu}}$** (600 MHz, $CDCl_3$, 298 K). Abbreviations: * = $CHCl_3$, w = water, h = H-grease.

_c_-P6$_{OOct}$ + pyridine-d_5

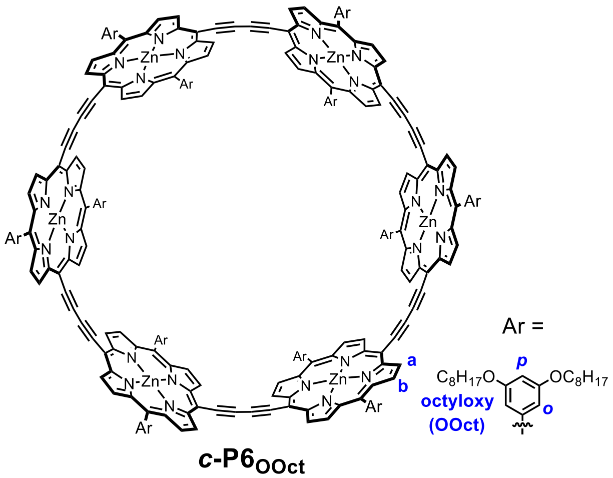

#	Assign.	^{1}H / ppm	Mult.	HSQC
**1**	a	9.57 (4H)	d, *J* = 4.5 Hz	130.4
**2**	b	8.84 (4H)	d, *J* = 4.5 Hz	133.0
**3**	*o*	7.21–7.18 (4H)	m	114.2
**4**	*p*	6.84–6.78 (2H)	m	100.7
**5**	OCH_2	4.05 (8H)	t, *J* = 6.7 Hz	68.3
**6**	aliphatic	1.83–1.74 (8H)	m	29.3
**7**	aliphatic	1.46–1.38 (8H)	m	26.0
**8**	aliphatic	1.33–1.14 (32H)	m	29.3, 29.5, 22.6, 31.7
**9**	aliphatic	0.79–0.73 (12H)	m	14.0

Figure S125. Top: Structure of ***c*-P6$_{OOct}$** with labels used for ^{1}H assignment. Bottom: Assigned ^{1}H resonances and ^{13}C chemical shift values for their associated carbon atoms. The number of protons is listed as per porphyrin unit.

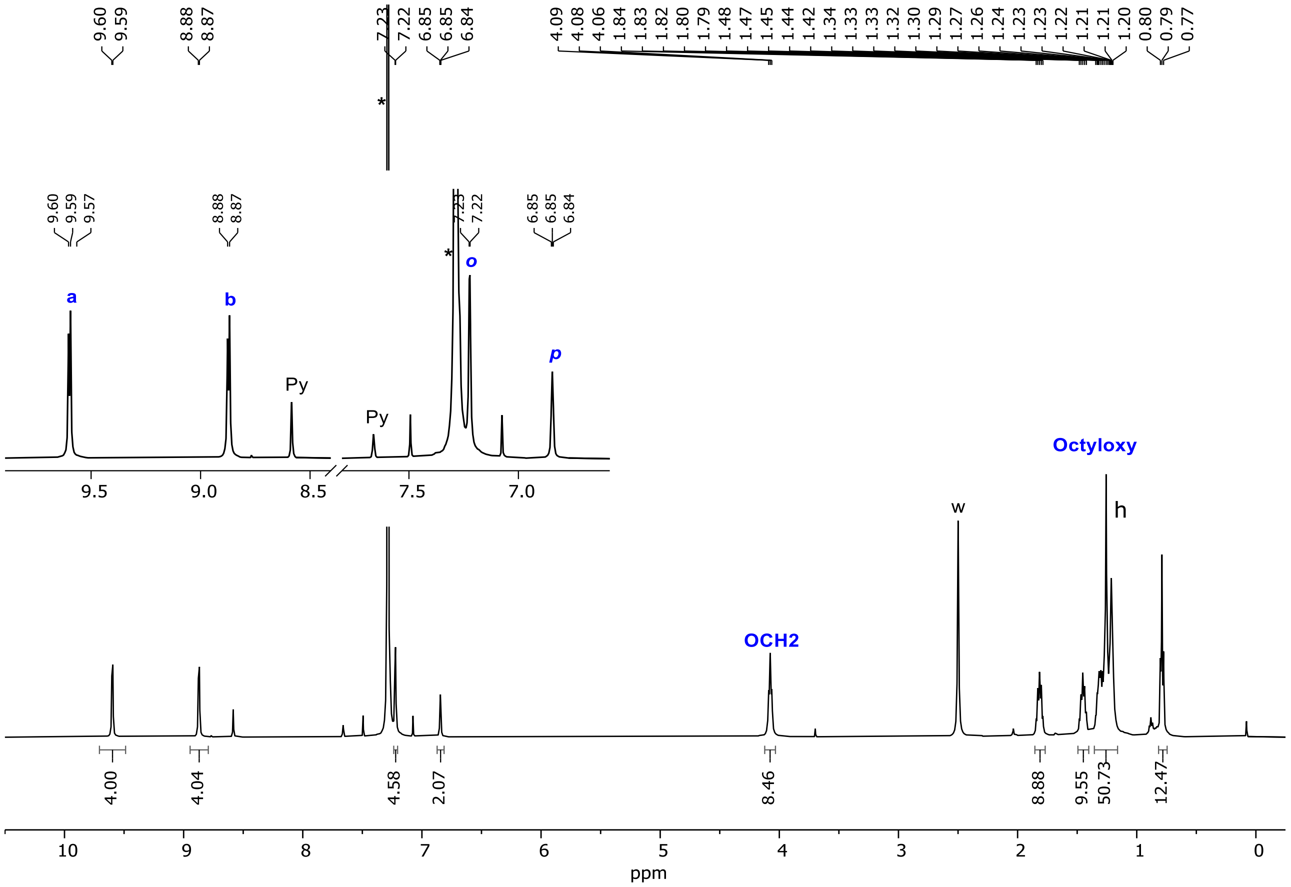

Figure S126. Assigned ^{1}H-NMR spectrum of ***c*-P6$_{OOct}$** (500 MHz, $CDCl_3$ + 5% pyridine-d_5, 298 K). Abbreviations: * = $CHCl_3$, Py = pyridine, w = water, h = H-grease.

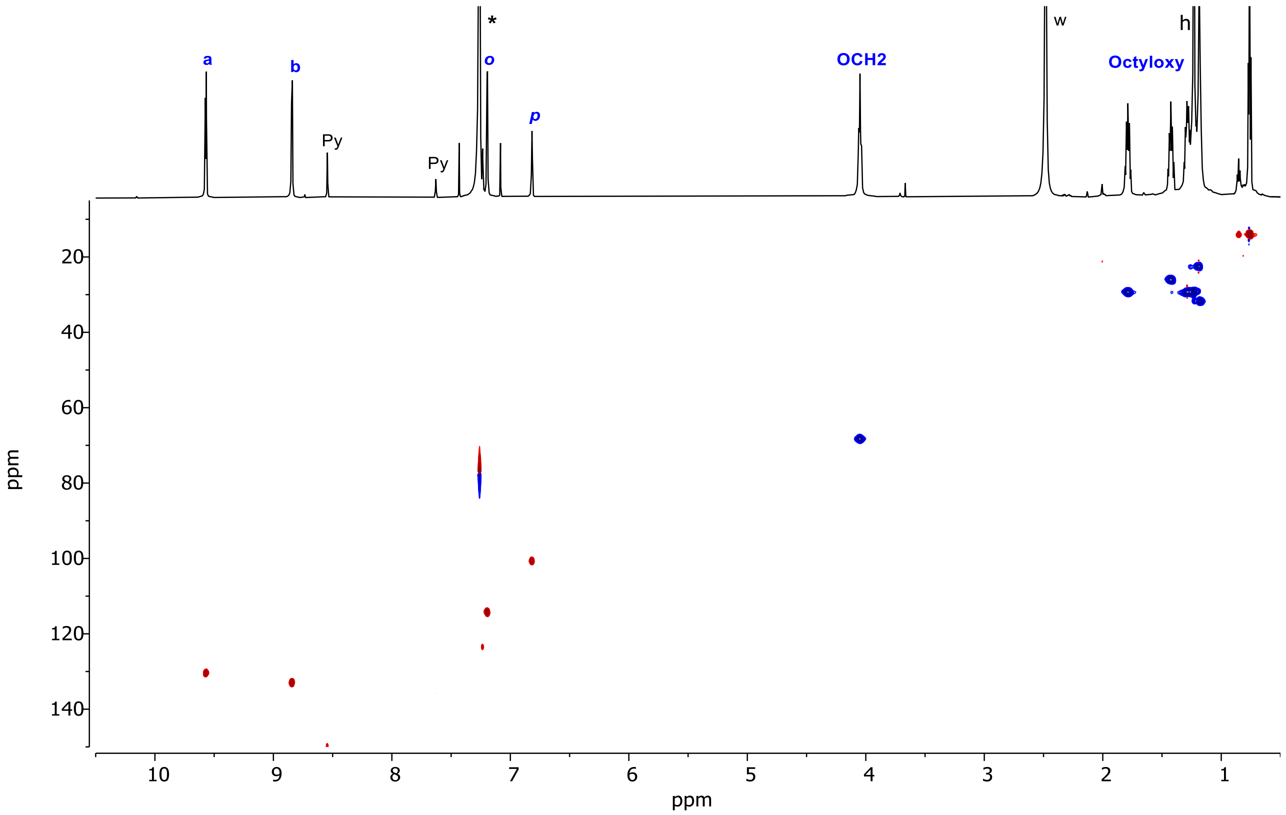

Figure S127. ^{1}H-^{13}C HSQC of ***c*-P6$_{OOct}$** (600 MHz, $CDCl_3$ + 5% pyridine-d_5, 298K). Abbreviations: * = $CHCl_3$, Py = pyridine, w = water, h = H-grease.

c-P6$_{OOct}$ in the absence of pyridine

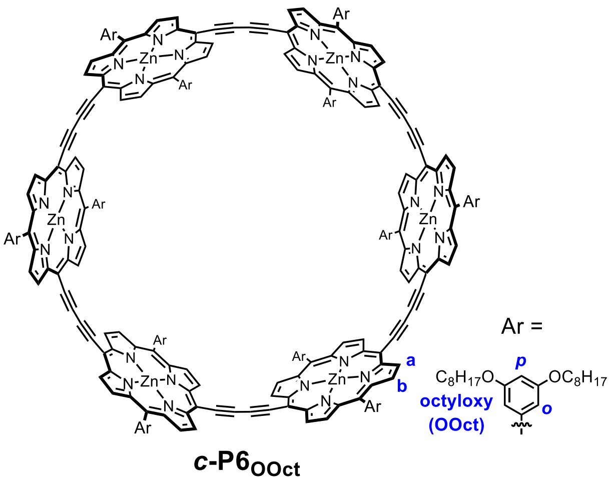

#	Assign.	^{1}H / ppm	Mult.
**1**	a	9.67 (4H)	br s
**2**	b	8.92 (4H)	br s
**3**	*o*	7.24–7.20 (4H)	br s (partially hidden by $CHCl_3$)
**4**	*p*	6.87–6.81 (2H)	br s
**5**	OCH_2	3.96 (8H)	br s
**6**	aliphatic	1.75 (8H)	br s
**7**	aliphatic	1.57–1.03 (8H)	m
**8**	aliphatic	1.57–1.03 (32H)	m
**9**	aliphatic	0.92–0.66 (12H)	m

Figure S128. Top: Structure of ***c*-P6$_{OOct}$** with labels used for ^{1}H assignment. Bottom: Assigned ^{1}H resonances. The number of protons is listed as per porphyrin unit.

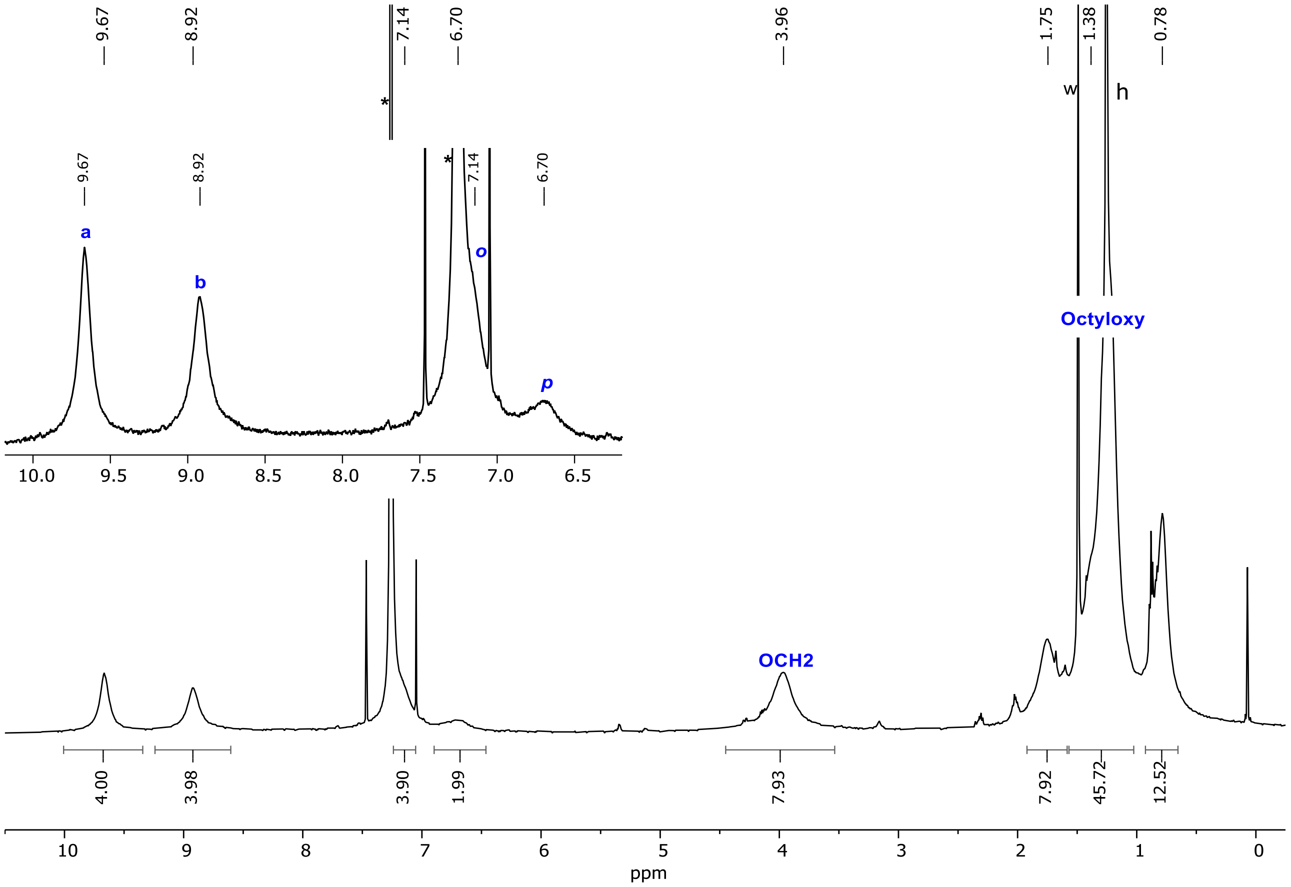

Figure S129. Assigned ^{1}H-NMR spectrum of ***c*-P6$_{OOct}$** (500 MHz, $CDCl_3$, 298 K). Abbreviations: * = residual $CHCl_3$ signal; w = water; h = H-grease.

c-P6$_{THS}$ + pyridine-d_5

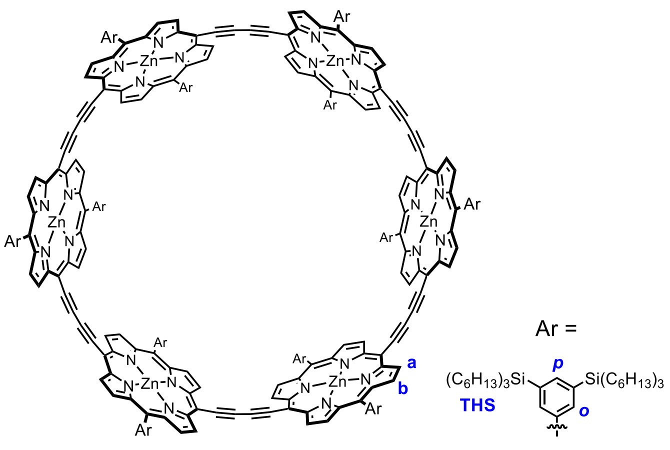

#	Assign.	^{1}H / ppm	Mult.	HSQC
**1**	a	9.55 (4H)	d, J = 4.5 Hz	130.3
**2**	b	8.69 (4H)	d, J = 4.5 Hz	132.9
**3**	*o*	8.09 (4H)	s	140.4
**4**	*p*	7.91 (2H)	s	139.0
**5**	THS	1.45– 1.37 (24H)	m	23.9
**6**	THS	1.32–1.26 (24H)	m	33.4
**7**	THS	1.26–1.18 (48H)	m	31.5, 22.5
**8**	THS	0.88–0.81 (24H)	m	12.6
**9**	THS	0.81–0.75 (36H)	m	14.0

Figure S130. Top: Structure of ***c*-P6$_{THS}$)** with labels used for ^{1}H assignment. Bottom: Assigned ^{1}H resonances and ^{13}C chemical shift values for their associated carbon atoms. The number of protons is listed as per porphyrin unit.

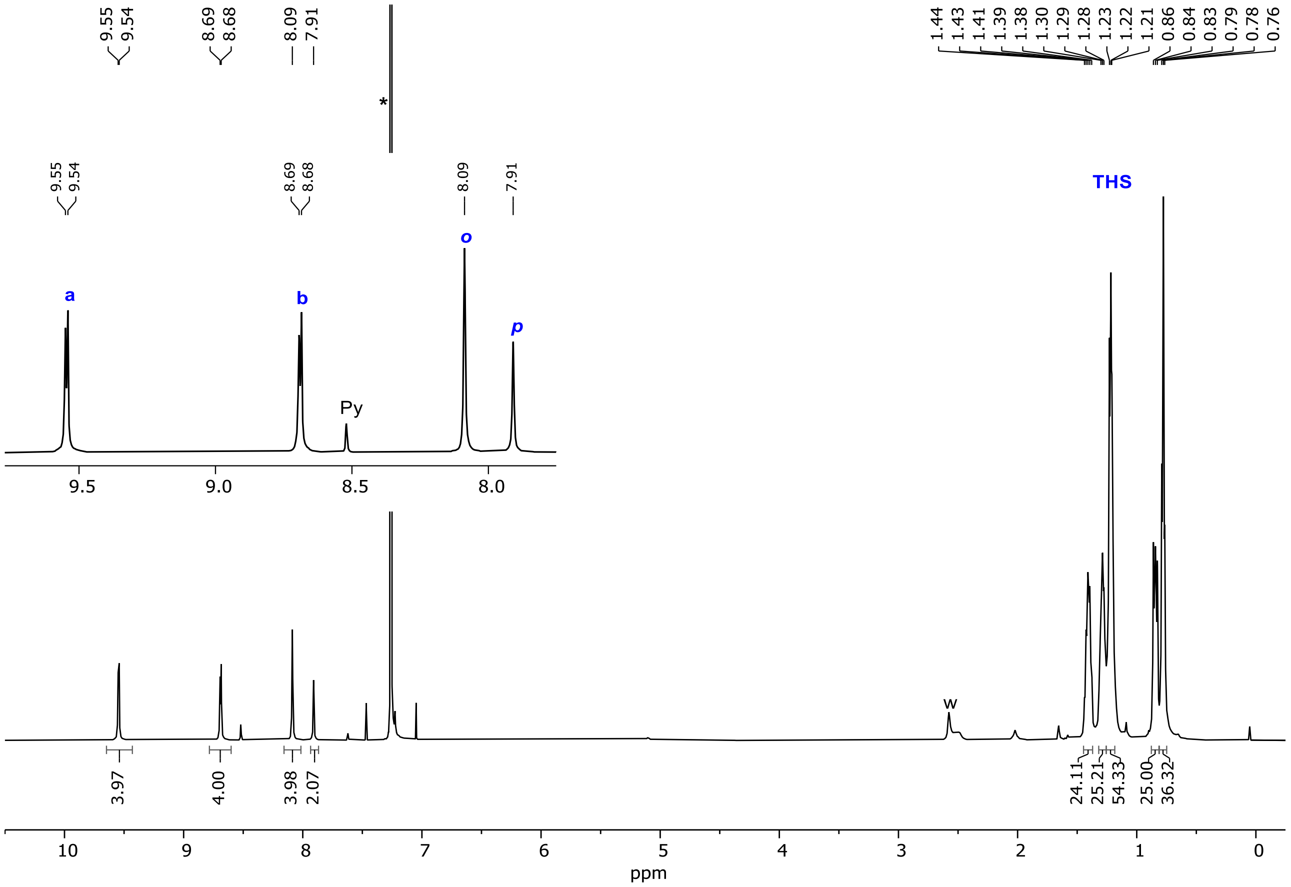

Figure S131. Assigned ^{1}H-NMR spectrum of ***c*-P6$_{THS}$** (500 MHz, $CDCl_3$ + 5% pyridine-d_5, 298 K). Abbreviations: * = $CHCl_3$, Py = pyridine, w = water.

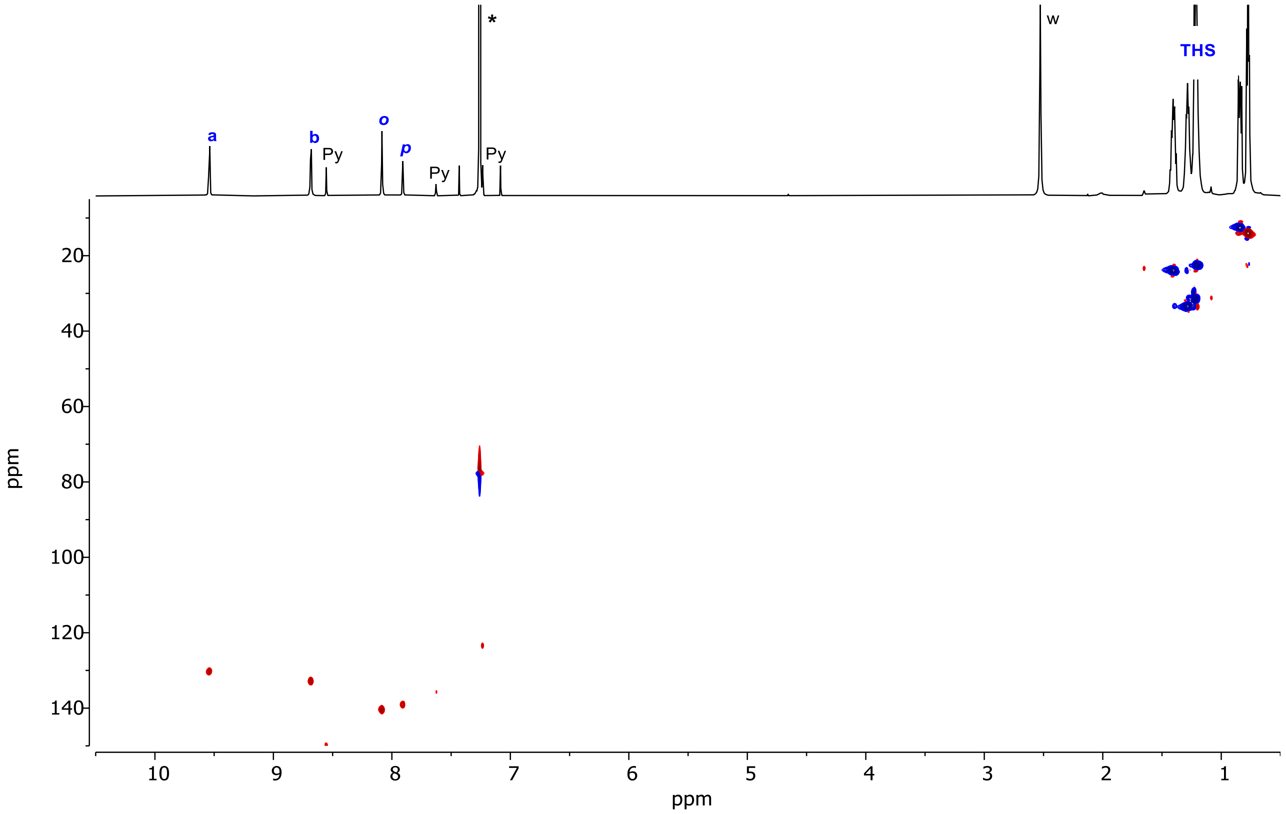

Figure S132. ^{1}H-^{13}C HSQC of ***c*-P6$_{THS}$** (600 MHz, $CDCl_3$ + 5% pyridine-d_5, 298 K). Abbreviations: * = $CHCl_3$, Py = pyridine, w = water.

c-P6$_{THS}$ in the absence of pyridine

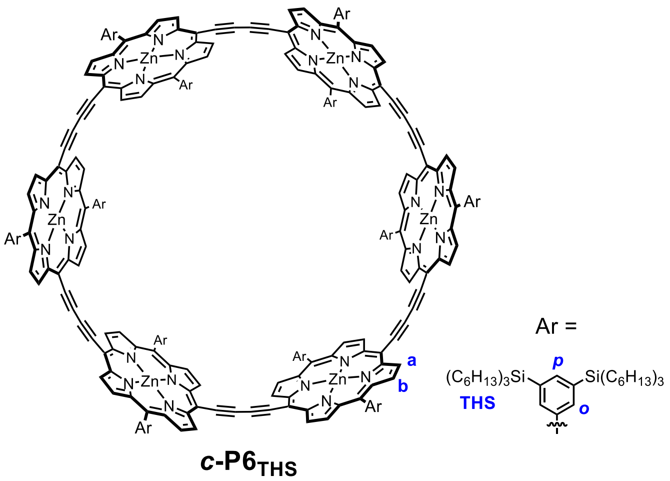

#	Assign.	^{1}H / ppm	Mult.	HSQC
1	a	9.67 (4H)	d, *J* = 4.5 Hz	130.2
2	b	8.81 (4H)	d, *J* = 4.5 Hz	132.8
3	*o*	8.15 (4H)	s	140.3
4	*p*	7.96 (2H)	s	139.0
5	THS	1.46–1.39 (24H)	m	23.8
6	THS	1.34–1.28 (24H)	m	33.4
7	THS	1.28–1.20 (48H)	m	31.4, 22.5
8	THS	0.89–0.84 (24H)	m	12.5
9	THS	0.83–0.76 (36H)	m	14.0

Figure S133. Top: Structure of ***c*-P6$_{THS}$** with labels used for ^{1}H assignment. Bottom: Assigned ^{1}H resonances and ^{13}C chemical shift values for their associated carbon atoms. The number of protons is listed as per porphyrin unit.

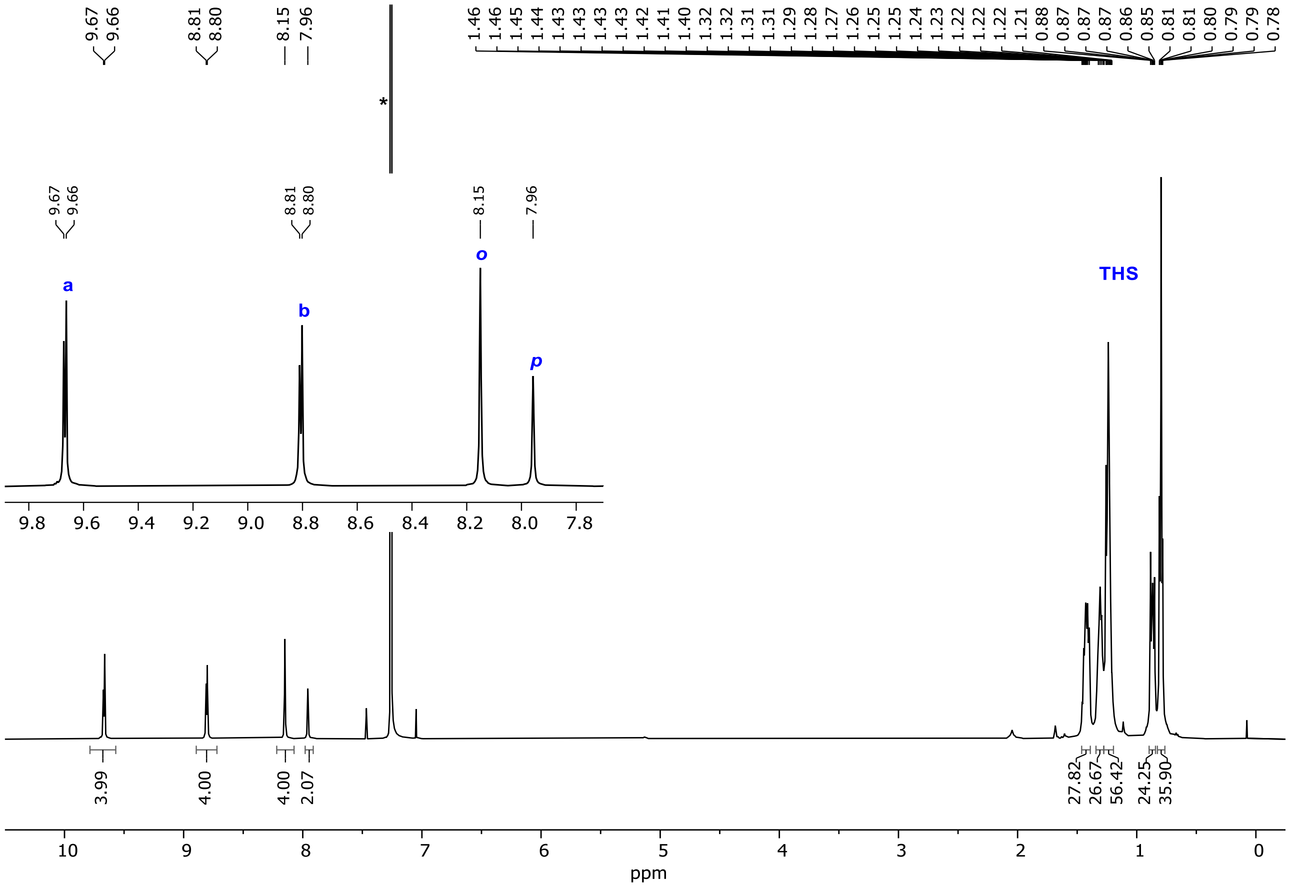

Figure S134. Assigned ^{1}H-NMR spectrum of ***c*-P6$_{THS}$** (500 MHz, $CDCl_3$, 298 K). Abbreviations: * = $CHCl_3$.

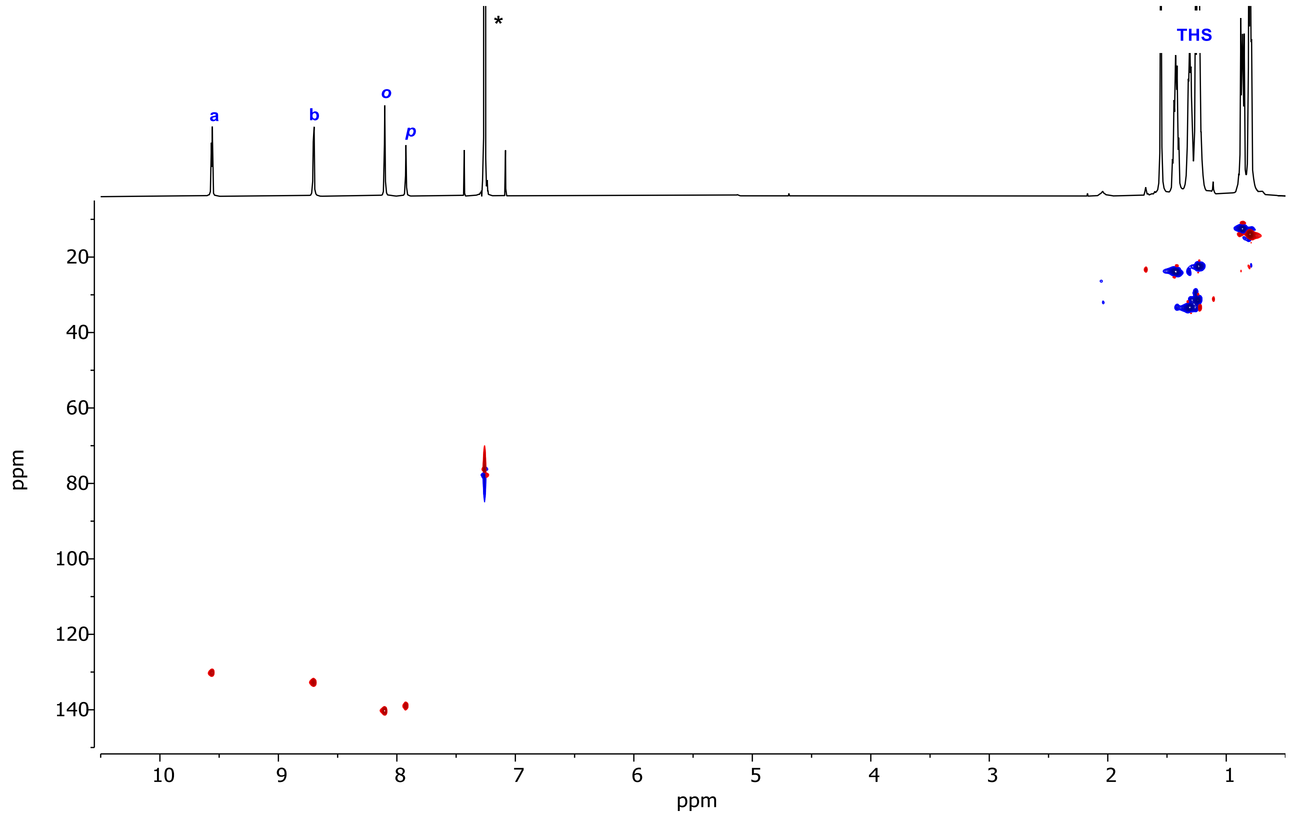

Figure S135. ^{1}H-^{13}C HSQC of ***c*-P6$_{THS}$** (600 MHz, $CDCl_3$, 298 K). Abbreviations: * = $CHCl_3$.

c-P8$_{t\text{-Bu}}$ + pyridine-d_5

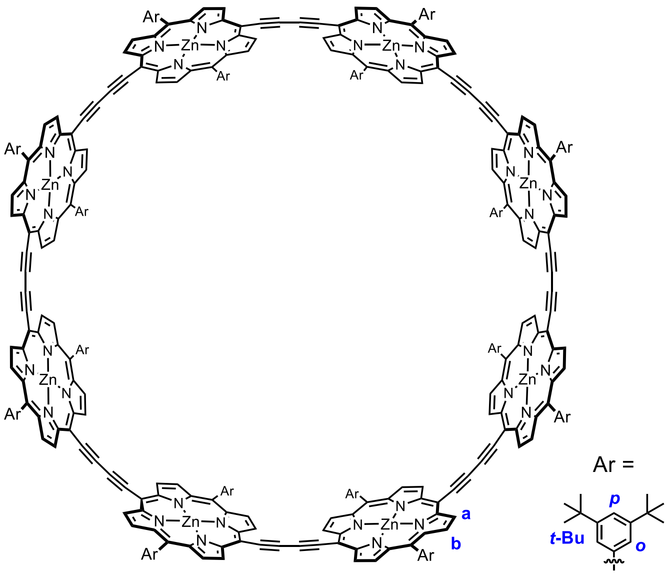

***c*-P8$_{t\text{-Bu}}$**

#	Assign.	^{1}H / ppm	Mult.	HSQC
**1**	a	9.72 (4H)	d, J = 4.4 Hz	130.2
**2**	b	8.86 (4H)	d, J = 4.4 Hz	133.0
**3**	*o*	7.96 (4H)	s	129.6
**4**	*p*	7.75 (2H)	s	120.7
**5**	*t*-Bu	1.49 (36H)	s	31.5

Figure S136. Top: Structure of ***c*-P8$_{t\text{-Bu}}$** with labels used for ^{1}H assignment. Bottom: Assigned ^{1}H resonances and ^{13}C chemical shift values for their associated carbon atoms. The number of protons is listed as per porphyrin unit.

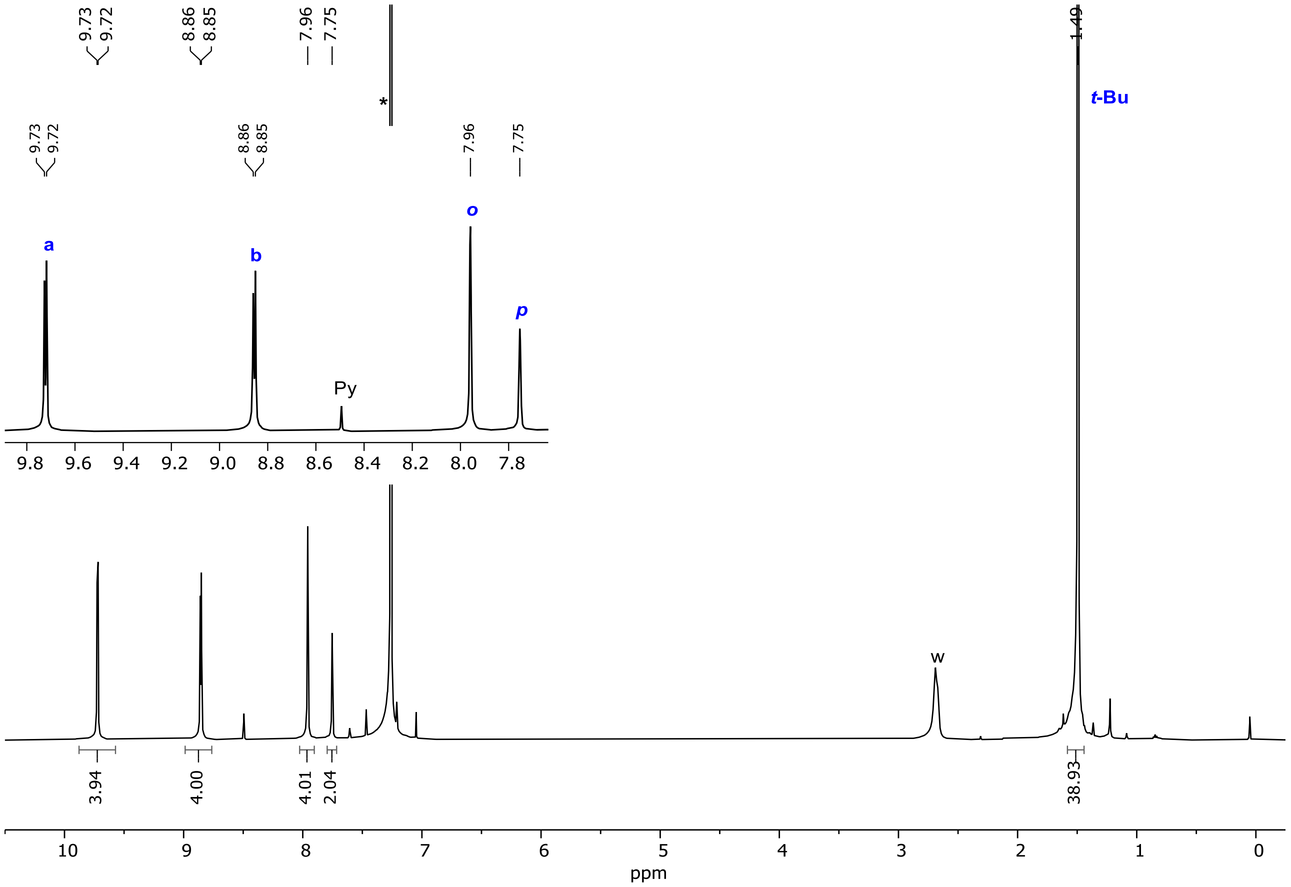

Figure S137. Assigned ^{1}H-NMR spectrum of ***c*-P8*$_{t\text{-Bu}}$*** (500 MHz, $CDCl_3$ + 5% pyridine-*d*$_5$, 298 K). Abbreviations: * = $CHCl_3$, Py = pyridine, w = water.

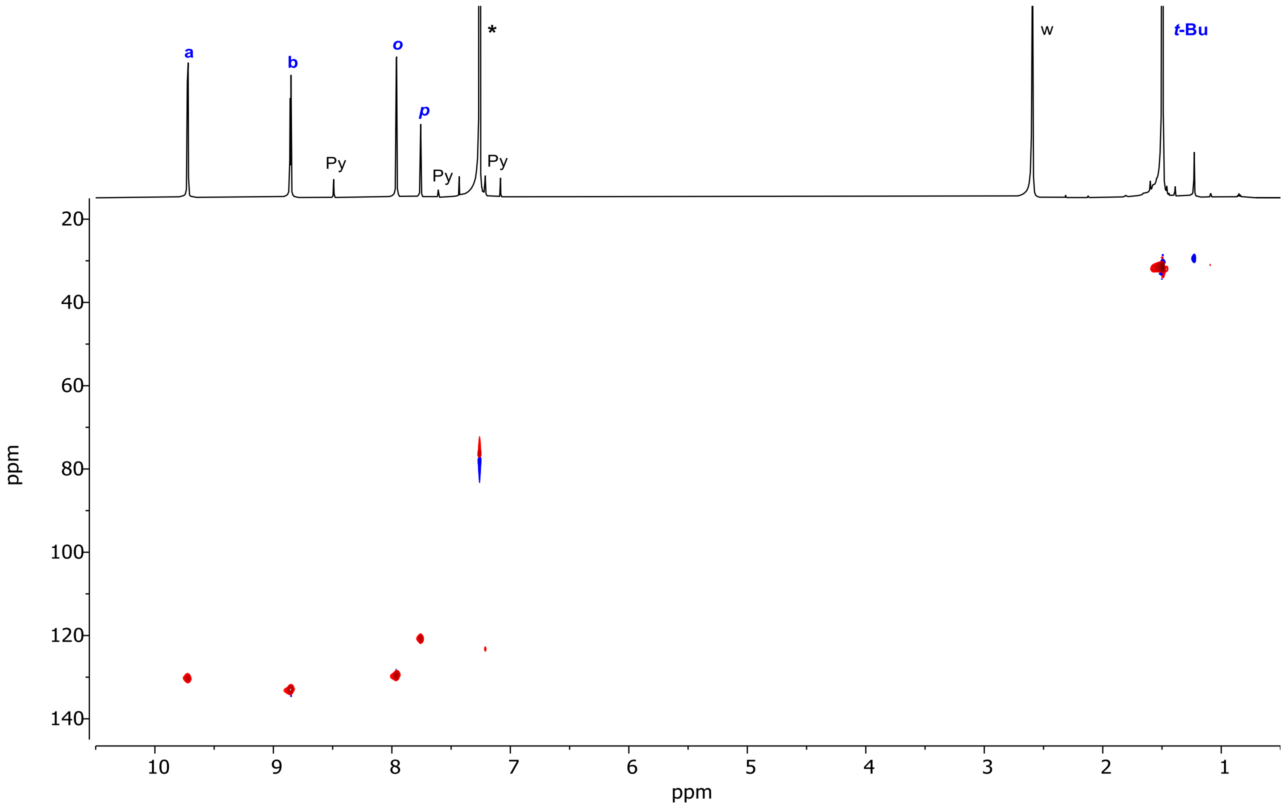

Figure S138. ^{1}H-^{13}C HSQC of ***c*-P8*$_{t\text{-Bu}}$*** (600 MHz, $CDCl_3$ + 5% pyridine-*d*$_5$, 298 K). Abbreviations: * = $CHCl_3$, Py = pyridine, w = water.

c-P8$_{t\text{-Bu}}$ **in the absence of pyridine**

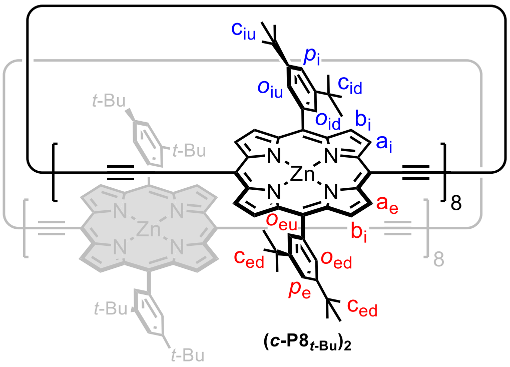

#	Assign.	^{1}H / ppm	Mult.	COSY	TOCSY	NOESY†	HSQC
1	o_{ed} + b_e	9.21–8.66 (3H)	m	3, 4	3, 4, 7	s: 4^X, 7; m: 2, 8; w: 3, 6, 10	129.5
2	o_{id} + b_i	8.65–8.11 (3H)	m	-	5, 6, 9	s: 6^X, 9, 10; m: 1, 4^X; w: 5, 7	129.2
3	p_e	7.99 (1H)	s	1, 4	1, 7, 8	s: 5^X, 7, 8; w: 1, 4, 10	121.1
4	o_{eu}	7.72 (1H)	br s	1, 3	1, 8	s: 1^X, 8; m: 2^X, 7; w: 3, 6	129.9
5	p_i	7.59 (1H)	br s	-	2, 9	s: 3^X, 9, 10; w: 2, 6, 7	120.7
6	o_{iu}	7.26–7.10 (m, 1H)	m	-	2	s: 2^X; m: 9, 10; w: 1, 4, 5	-
7	c_{ed}	1.92 (9H)	br s	-	1, 3, 8	s: 1, 3, 8; m: 4, 9^X, 10^X; w: 2, 5	32.2
8	c_{id}	1.55 (9H)	br s	-	3, 4, 7	s: 3, 4, 7; m: 1, 10^X	31.7
9	c_{eu}	1.48 (9H)	br s	-	2, 5, 10	s: 2, 5, 10; m: 6, 7^X	31.8
10	c_{iu}	1.19 (9H)	br s	-	9	s: 2, 5, 9; m: 6, 7^X, 8^X; w: 1, 3	31.4

Figure S139. Top: Structure of **(*c*-P8$_{t\text{-Bu}}$)$_2$** with labels used for ^{1}H assignment. Bottom: Assigned ^{1}H resonances and ^{13}C chemical shift values for their associated carbon atoms. The number of protons is listed as per porphyrin unit. †Relative strengths of NOE correlations: s = strong, m = medium, w = weak, vw = very weak. ^{X}Correlation in NOESY which (by ROESY) was found to stem from chemical exchange rather than a through-space dipolar interaction.

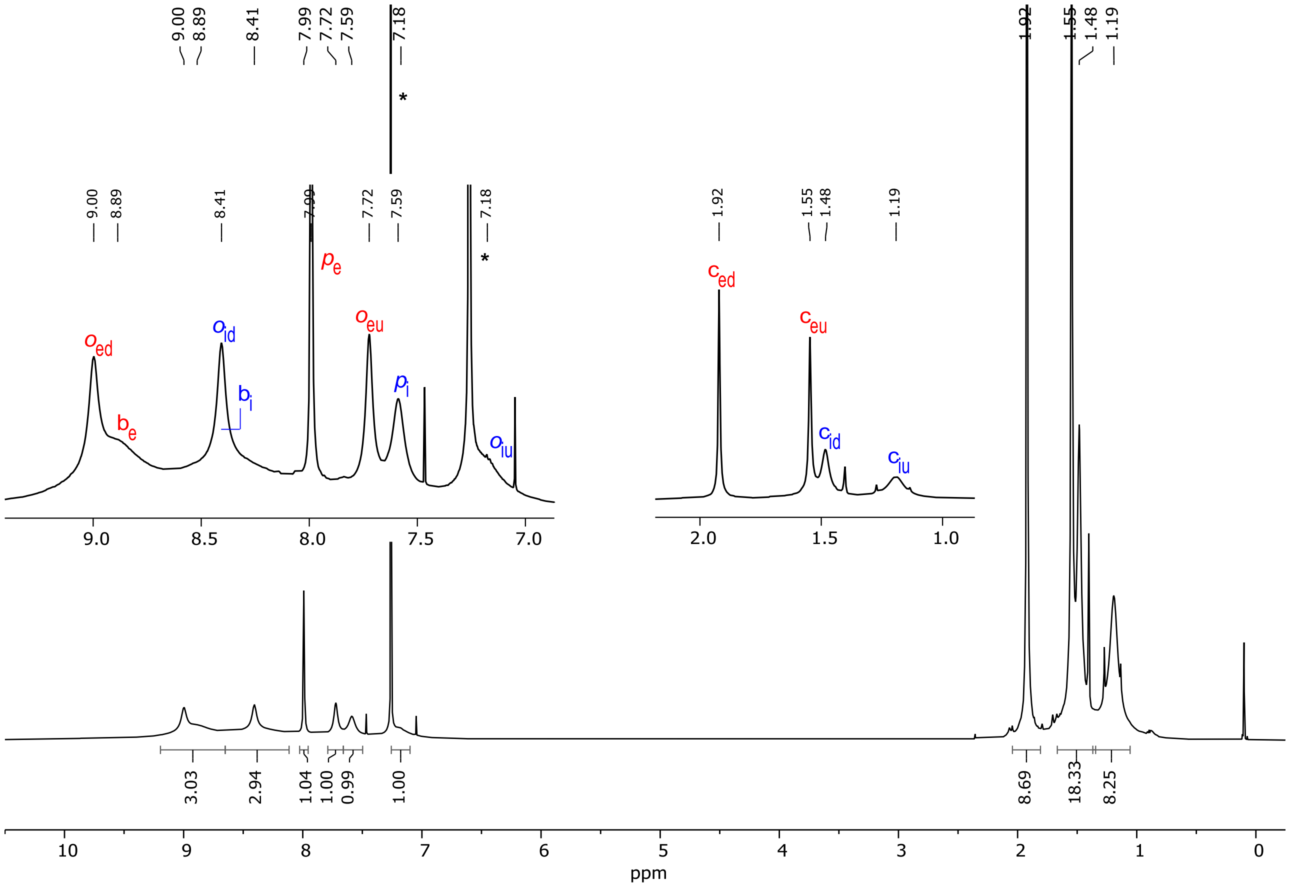

Figure S140. Assigned ^{1}H-NMR spectrum of **(*c*-P8$_{t\text{-Bu}}$)$_2$** (500 MHz, $CDCl_3$, 298 K). Abbreviations: * = residual $CHCl_3$ signal.

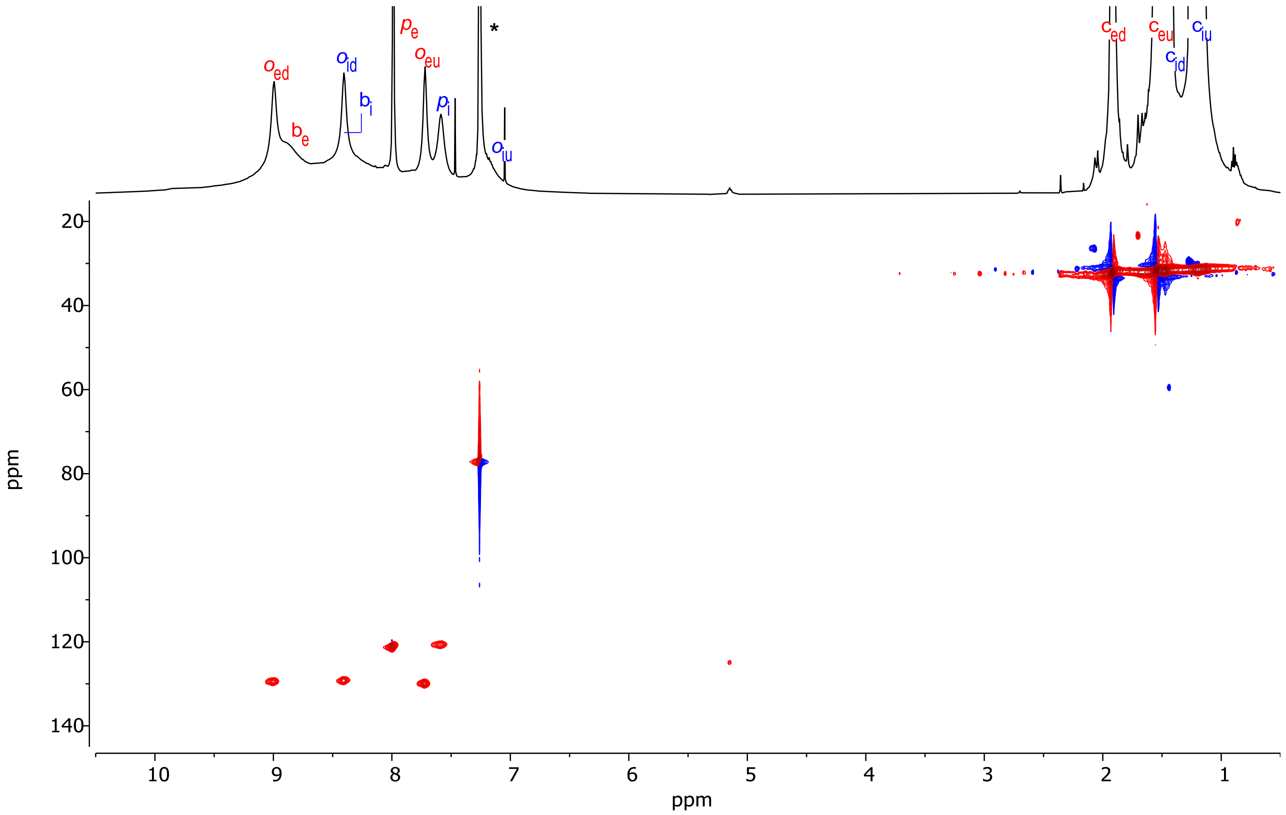

Figure S141. ^{1}H-^{13}C HSQC of **(*c*-P8$_{t\text{-Bu}}$)$_2$** (500 MHz, $CDCl_3$, 298 K). Abbreviations: * = $CHCl_3$.

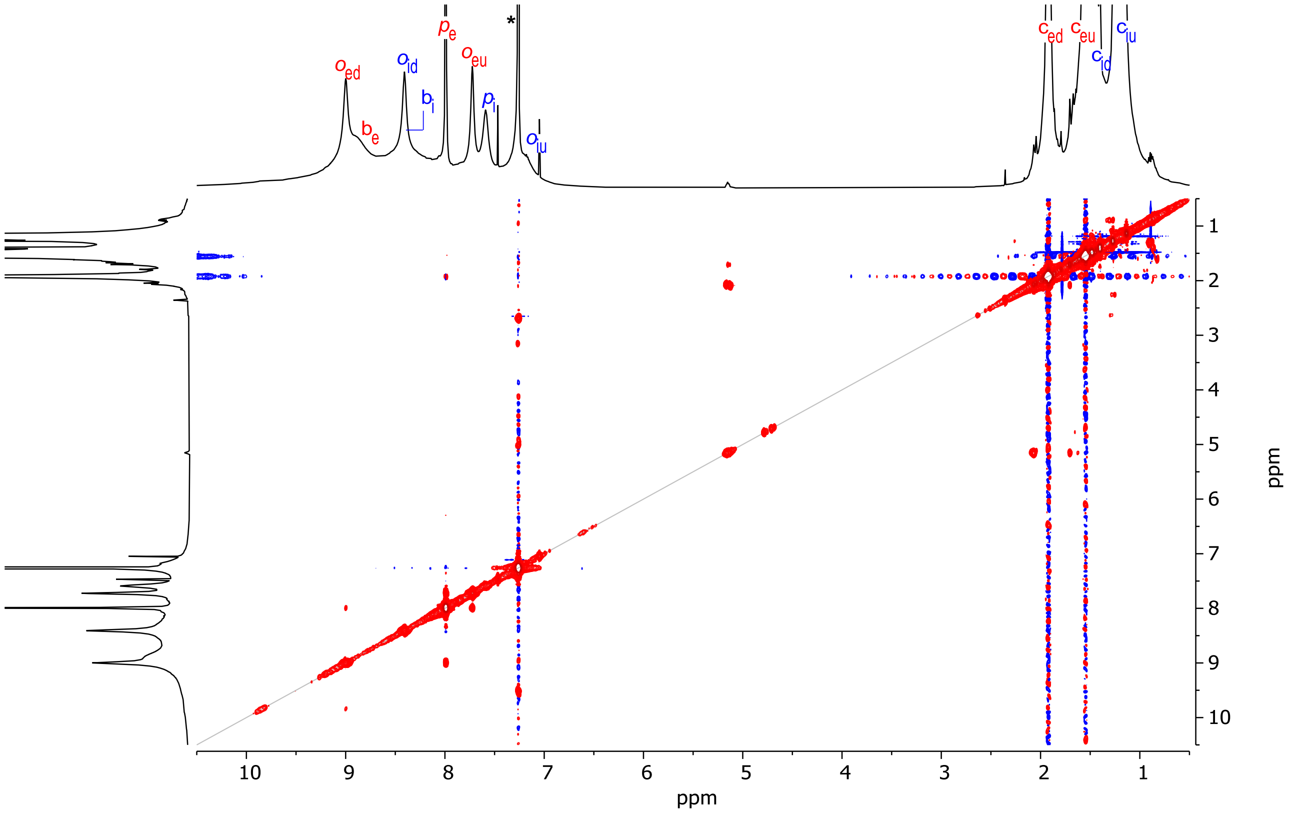

Figure S142. ^{1}H-^{1}H COSY spectrum of **(*c*-P8$_{t\text{-Bu}}$)$_2$** (500 MHz, $CDCl_3$, 298 K). Abbreviations: * = $CHCl_3$.

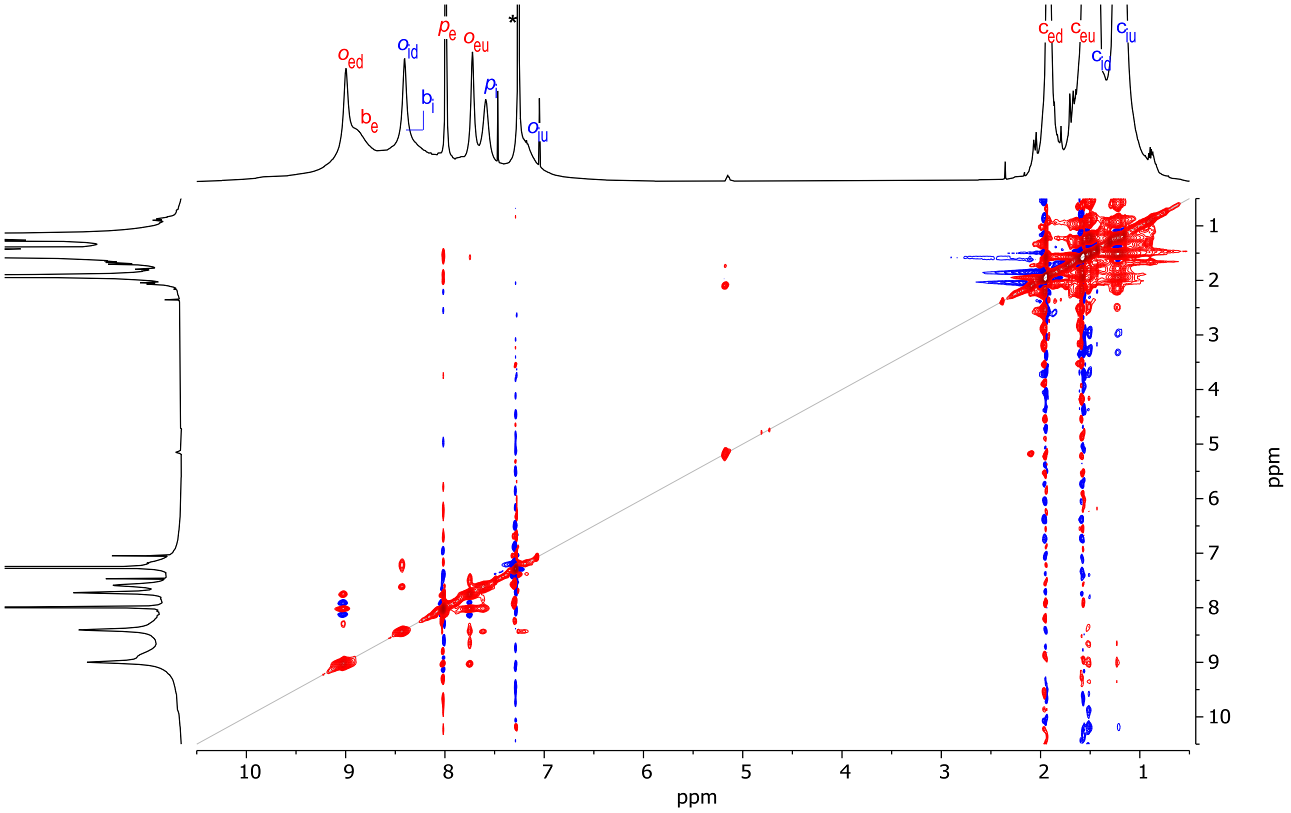

Figure S143. ^{1}H-^{1}H TOCSY spectrum of **(*c*-P8$_{t\text{-Bu}}$)$_2$** (500 MHz, $CDCl_3$, 298 K). Abbreviations: * = $CHCl_3$.

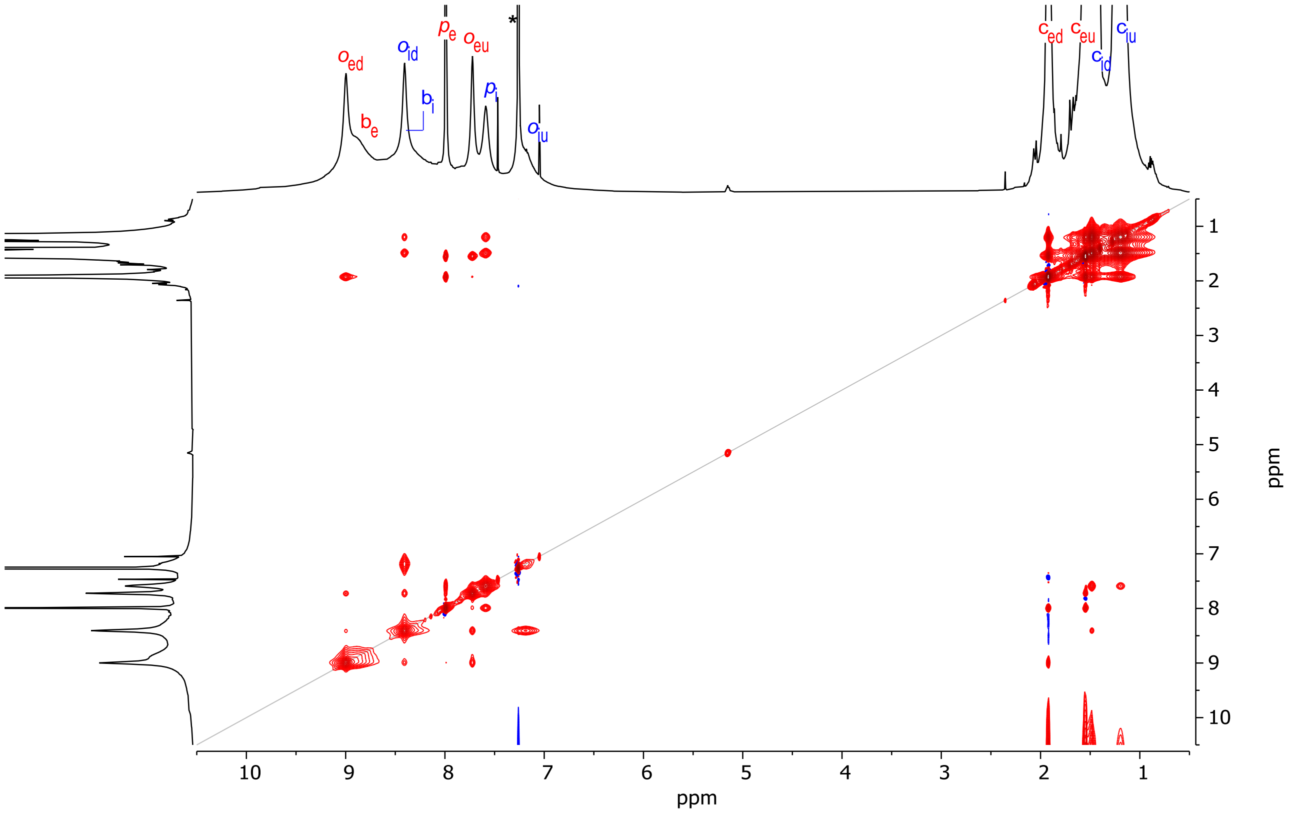

Figure S144. ^{1}H-^{1}H NOESY spectrum of **(*c*-P8*t*-Bu)2** (500 MHz, $CDCl_3$, 298 K). Abbreviations: * = $CHCl_3$.

c-P8$_{OOct}$ + pyridine-d_5

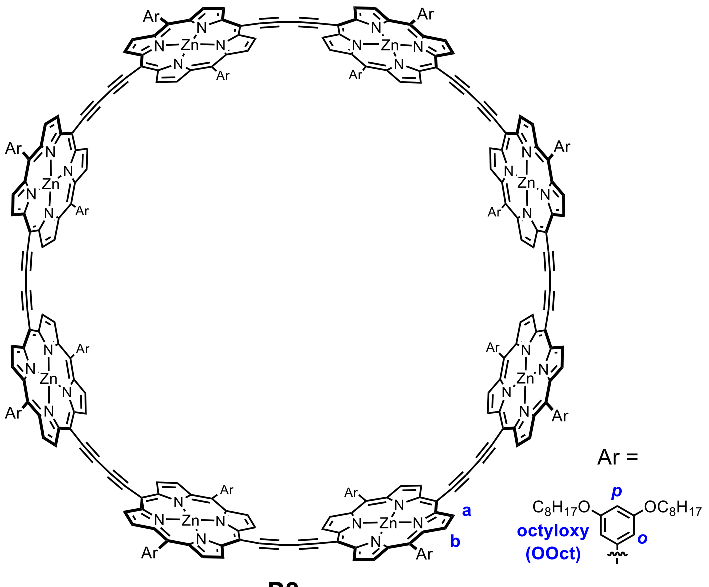

#	Assign.	^{1}H / ppm	Mult.	HSQC
**1**	a	9.69 (4H)	d, J = 4.5 Hz	130.3
**2**	b	8.93 (4H)	d, J = 4.5 Hz	132.8
**3**	*o*	7.27 (4H)	d, J = 2.0 Hz	114.1
**4**	*p*	6.84 (2H)	t, J = 2.0 Hz	100.5
**5**	OOct (OCH2)	4.08 (8H)	t, J = 6.3 Hz	68.1
**6**	OOct	1.85–1.77 (8H)	m	29.2
**7**	OOct	1.48–1.41 (8H)	m	25.9
**8**	OOct	1.35–1.15 (32H)	m	29.1, 29.0, 29.2, 22.3, 31.5
**9**	OOct	0.81–0.73 (12H)	m	13.8

Figure S145. Top: Structure of ***c*-P8$_{OOct}$** with labels used for ^{1}H assignment. Bottom: Assigned ^{1}H resonances and ^{13}C chemical shift values for their associated carbon atoms. The number of protons is listed as per porphyrin unit.

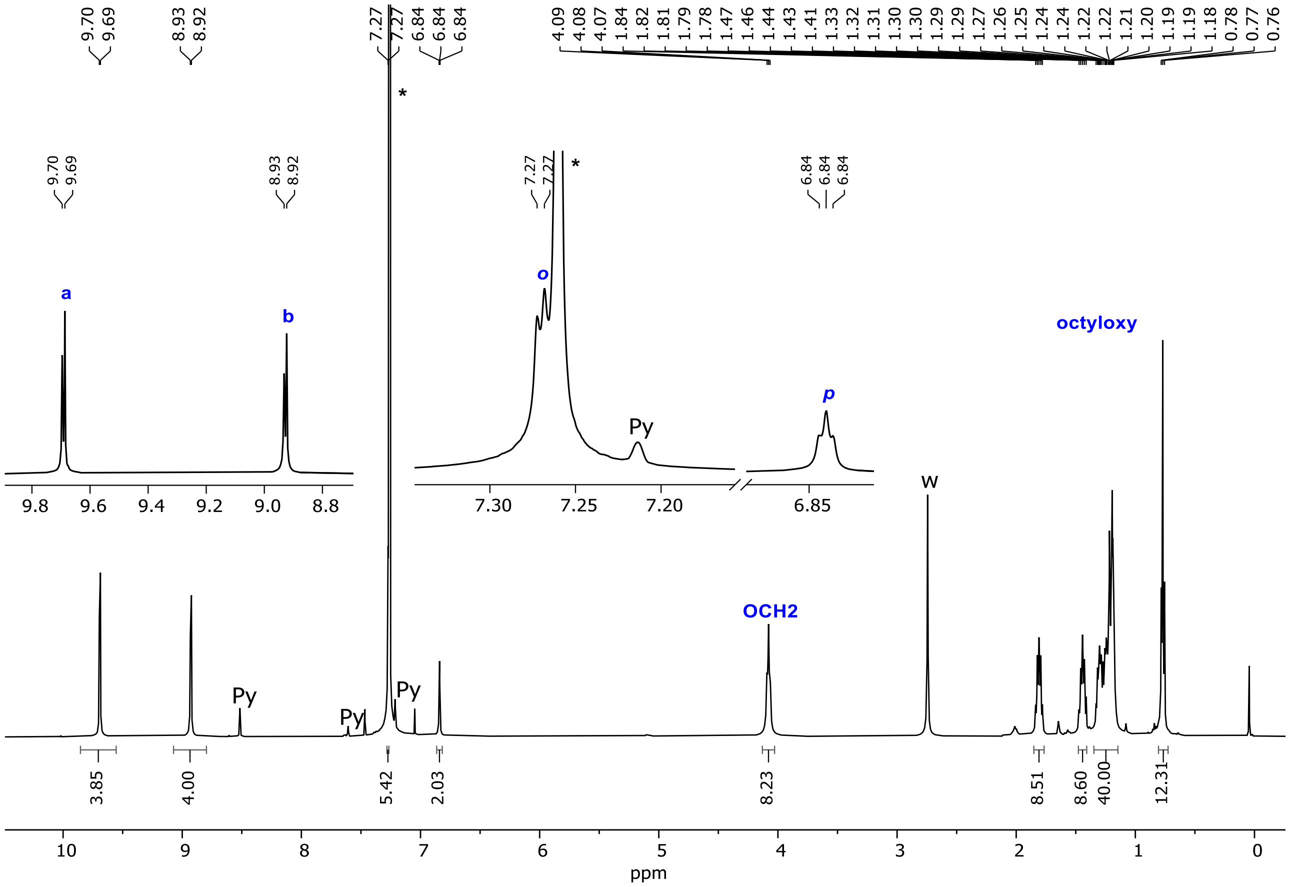

Figure S146. Assigned ^{1}H-NMR spectrum of ***c*-P8**$_{\mathbf{OOct}}$ (500 MHz, $CDCl_3$ + 5% pyridine-d_5, 298 K). Abbreviations: * = $CHCl_3$, Py = pyridine, w = water.

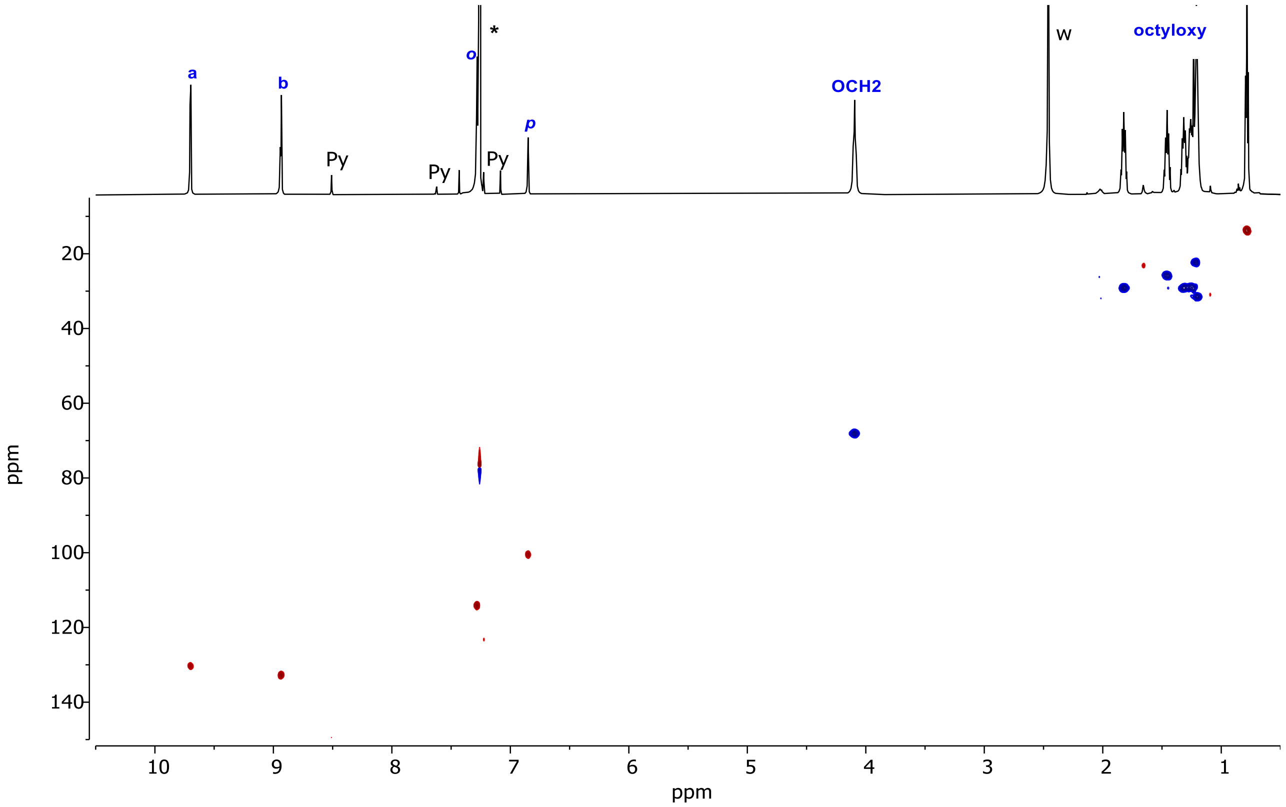

Figure S147. ^{1}H-^{13}C HSQC of ***c*-P8**$_{\mathbf{OOct}}$ (600 MHz, $CDCl_3$ + 5% pyridine-d_5, 298 K). Abbreviations: * = $CHCl_3$, Py = pyridine, w = water.

c-P8$_{OOct}$ in the absence of pyridine

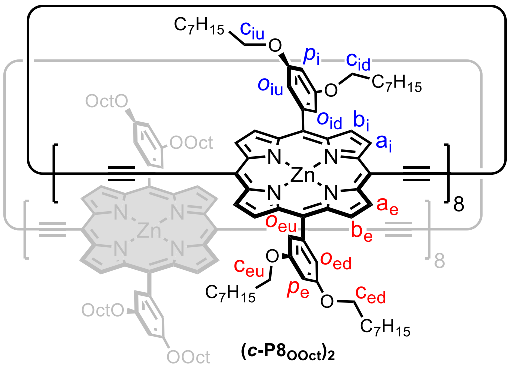

#	Assign.	^{1}H / ppm	Mult.	COSY	TOCSY	NOESY†	HSQC / ppm
1	b_e	8.98 (16H)	s	-	-	s: 2, 3; m: 7, 10; w: 5, 12	132.3
2	a_e	8.81 (16H)	br s	-	-	s: 1; m: 3	-
3	b_i + o_{ed}	8.54–8.25 (24H)	m	6, 7	6, 7, 8, 9	s: 1, 5, 9^X, 10; m: 2, 4, 7^X, 11, 13; w: 6, 12	132.2, 114.0
4	a_i	8.04 (16H)	br s	-	-	m: 3	-
5	o_{id}	7.74 (8H)	s	8, 9	6, 7, 8, 9	s: 3, 7^X, 11; m: 9^X, 12; w: 1, 13	114.3
6	p_e	7.09 (8H)	s	3, 7	3, 5, 7, 8, 9	s: 8^X, 10, 12; m: 11; w: 3, 13	101.4
7	o_{eu}	6.97 (8H)	s	3, 6	3, 5, 6, 8, 9	s: 5^X, 12; m: 1, 3^X, 11; w: 10	114.5
8	p_i	6.70 (8H)	s	5, 9	3, 5, 6, 7, 9	s: 6^X, 11, 13; m: 12; w: 10	100.9
9	o_{iu}	6.43 (8H)	s	5, 8	3, 5, 6, 7, 8	s: 3^X, 13; m: 5^X, 10; w: 11	114.2
10	c_{ed}	4.70–4.55 (16H)	m	14	12, 13, 14, 15, 16	s: 3, 6, 13^X, 14; m: 1, 9, 12^X, 15; w: 7, 8, 11^X, 16	69.0
11	c_{id}	4.22–4.09 (16H)	m	15	13, 15, 16	s: 5, 8; m: 3, 6, 7, 13^X; w: 9, 10^X	68.4
12	c_{eu}	4.10–3.99 (16H)	m	15	10, 15, 16	s: 6, 7, 15; m: 5, 8, 10^X, 16; w: 1, 3, 13^X	68.3
13	c_{iu}	3.78–3.59 (16H)	m	16	10, 11, 14, 15, 16	s: 8, 9, 10^X, 16; m: 3, 11^X; w: 5, 6, 12^X, 15	67.9
14	OOct	2.27–2.16 (16H)	m	10	10, 13	s: 10	29.8
15	OOct	1.94–1.73 (48H)	m	11	10, 11, 12, 13	s: 11, 12; m: 10; w: 13	29.6, 29.4, 26.4
16	OOct	1.63–0.88 (320H)	m	13, 17, 18, 19	10, 11, 12, 13, 17, 18, 19	s: 13, 17, 18, 19; m: 11, 12; w: 10	29.7, 29.2, 29.4, 26.2, 26.1, 31.9, 29.5, 22.6, 29.5, 22.5, 31.7, 25.8, 31.9, 22.4, 29.0, 22.3, 31.5
17	OOct (-CH$_3$)	0.86–0.77 (48H)	m	16, 16	16	s: 18, 19	14.0, 14.0
18	OOct (-CH$_3$)	0.64–0.55 (24H)	m	16	16	s: 17	13.8
19	OOct (-CH$_3$)	0.55–0.46 (24H)	m	16	16	s: 17	13.7

Figure S148. Top: Structure of **(c-P8$_{OOct}$)$_2$** with labels used for ^{1}H assignment. Bottom: Assigned ^{1}H resonances and ^{13}C chemical shift values for their associated carbon atoms. The number of protons is listed as per porphyrin unit. †Relative strengths of NOE correlations: s = strong, m = medium, w = weak, vw = very weak. ^{X}Correlation in NOESY which (by ROESY) was found to stem from chemical exchange rather than a through-space dipolar interaction.

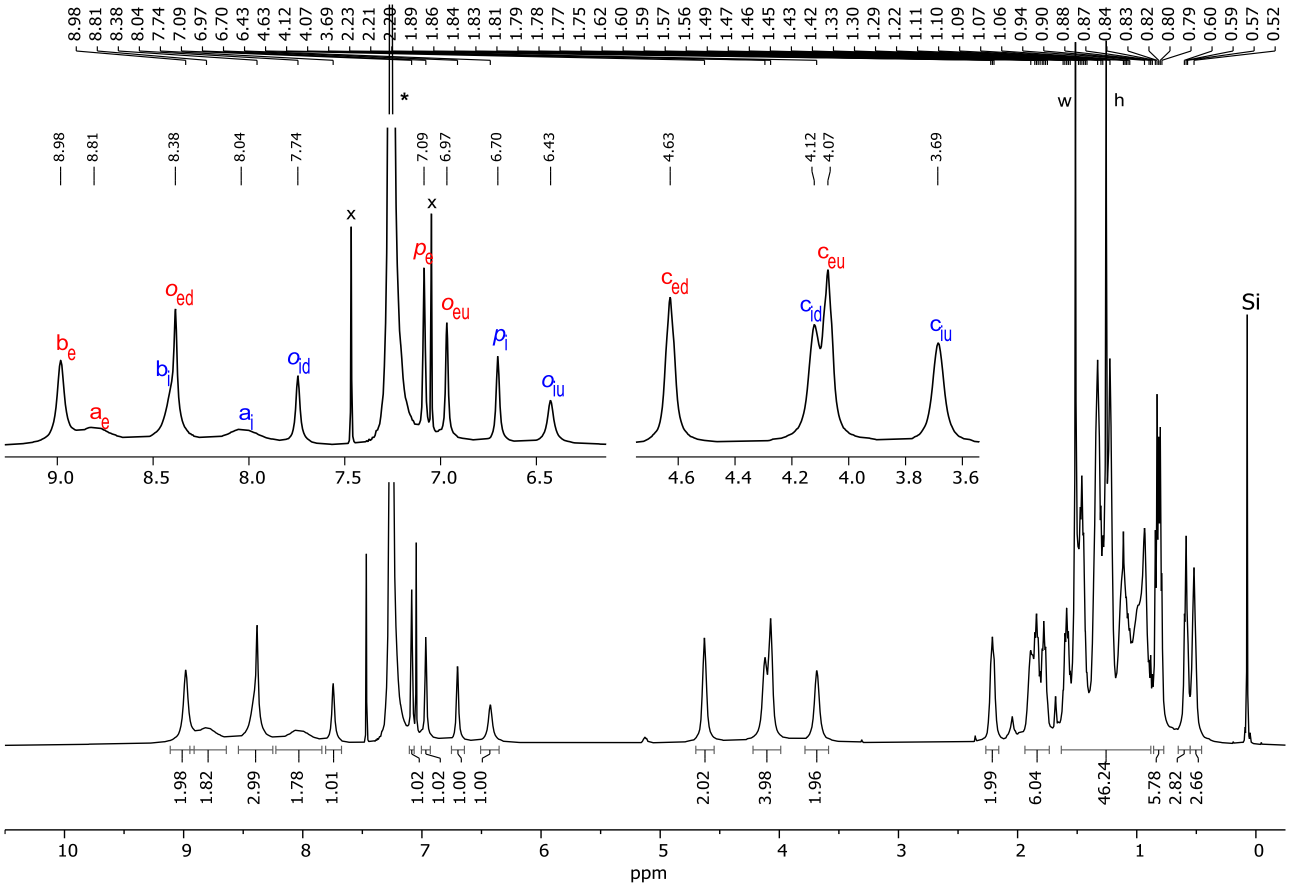

Figure S149. Assigned ^{1}H-NMR spectrum of **(*c*-P8$_{OOct}$)$_2$** (500 MHz, $CDCl_3$, 298 K). Abbreviations: * = $CHCl_3$, Py = pyridine, w = water, h = H-grease, Si = silicone grease. x = satellite signals of $^{13}CHCl_3$.

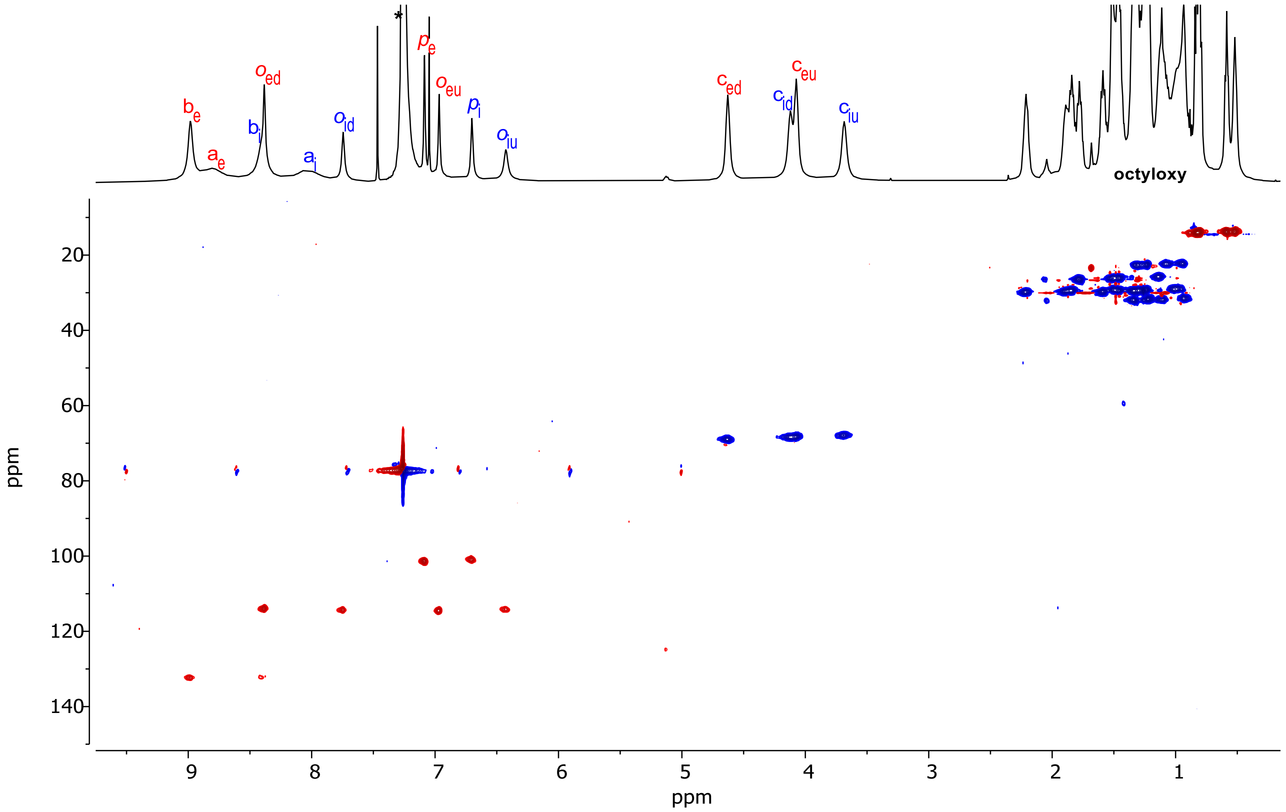

Figure S150. ^{1}H–^{13}C HSQC of **(*c*-P8$_{OOct}$)$_2$** (600 MHz, $CDCl_3$, 298 K). Abbreviations: * = $CHCl_3$, Si = silicone grease.

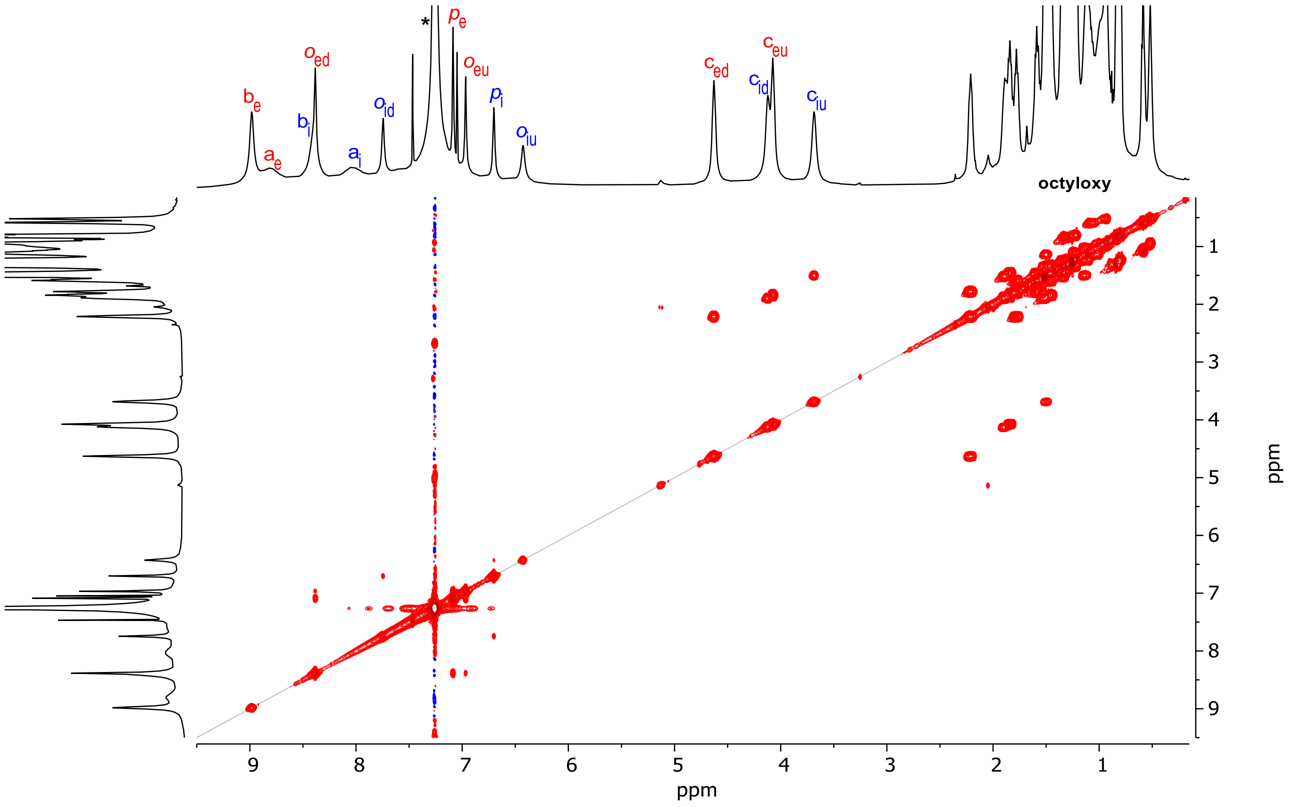

Figure S151. ^{1}H–^{1}H COSY spectrum of **(*c*-P8$_{OOct}$)$_2$** (500 MHz, $CDCl_3$, 298 K). Abbreviations: * = $CHCl_3$, Si = silicone grease.

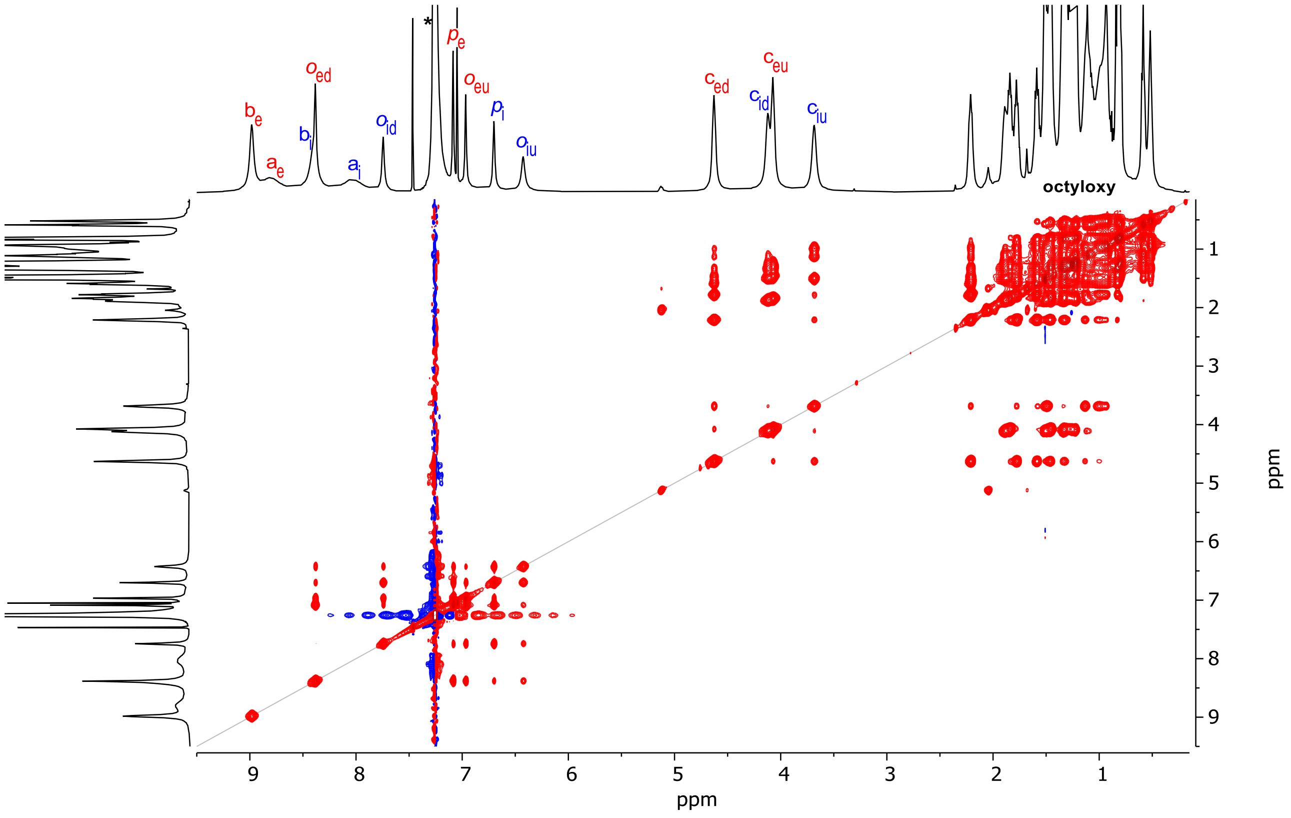

Figure S152. ^{1}H–^{1}H TOCSY spectrum of **(*c*-P8$_{OOct}$)$_2$** (500 MHz, $CDCl_3$, 298 K). Abbreviations: * = $CHCl_3$, Si = silicone grease.

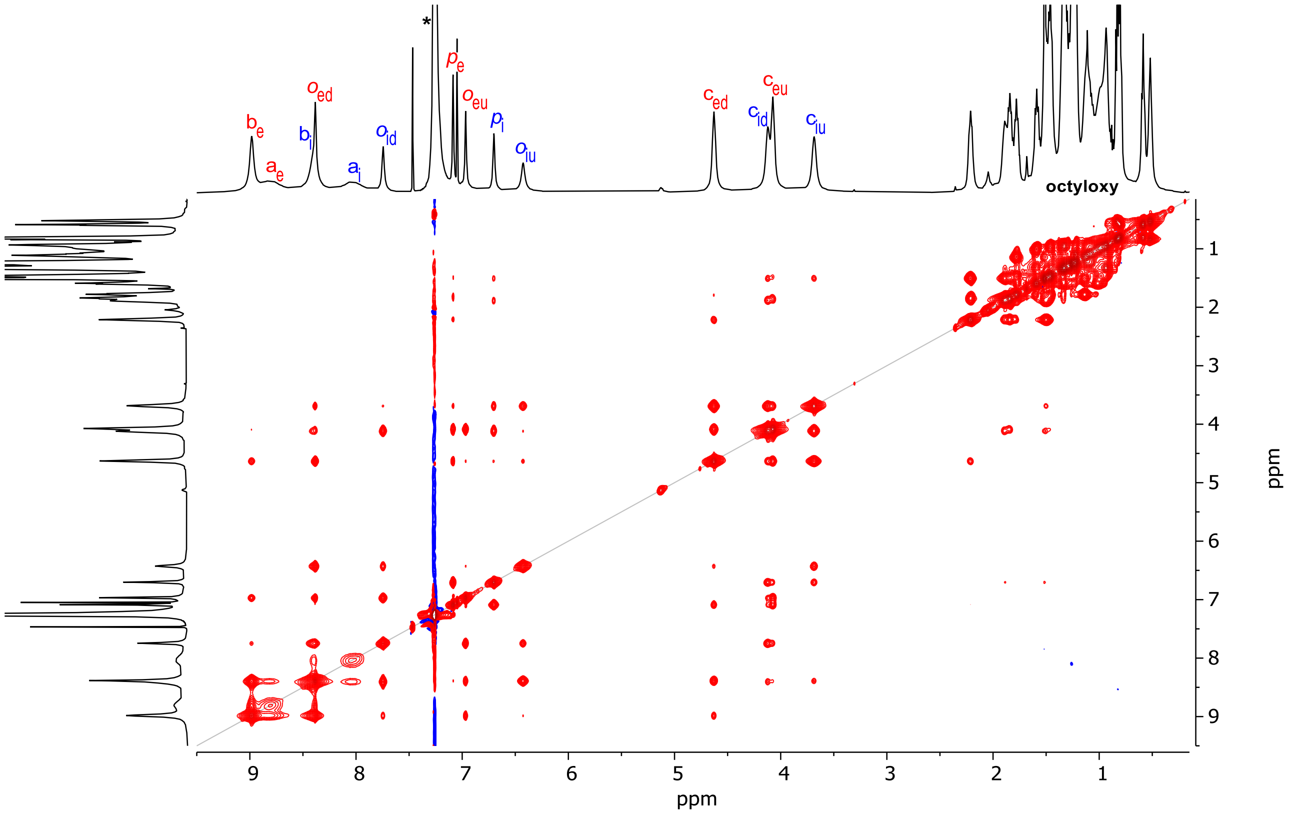

Figure S153. ^{1}H–^{1}H NOESY spectrum of **(*c*-P8$_{\mathbf{OOct}}$)$_2$** (500 MHz, $CDCl_3$, 298 K). Abbreviations: * = $CHCl_3$, Si = silicone grease.

c-**P8**$_{THS}$ + pyridine-d_5

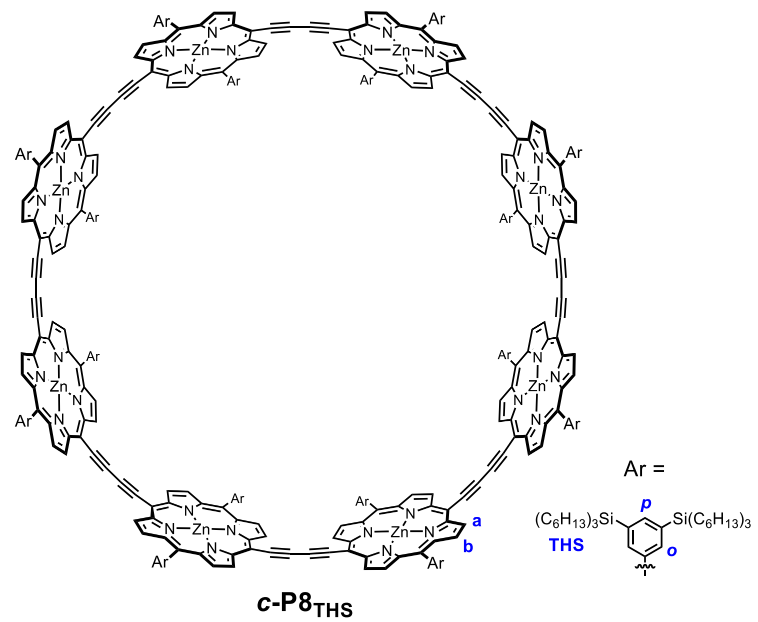

#	Assign.	^{1}H / ppm	Mult.	HSQC
**1**	a	9.67 (4H)	d, *J* = 4.5 Hz	130.3
**2**	b	8.78 (4H)	d, *J* = 4.5 Hz	132.8
**3**	*o*	8.16 (4H)	s	140.5
**4**	*p*	7.93 (2H)	s	138.9
**5**	THS	1.47–1.38 (24H)	m	23.9
**6**	THS	1.34–1.27 (24H)	m	33.4
**7**	THS	1.27–1.20 (48H)	m	31.5, 22.5
**8**	THS	0.90–0.84 (24H)	m	12.5
**9**	THS	0.83–0.76 (36H)	m	14.0

Figure S154. Top: Structure of ***c*-P8**$_{THS}$ with labels used for ^{1}H assignment. Bottom: Assigned ^{1}H resonances and ^{13}C chemical shift values for their associated carbon atoms. The number of protons is listed as per porphyrin unit.

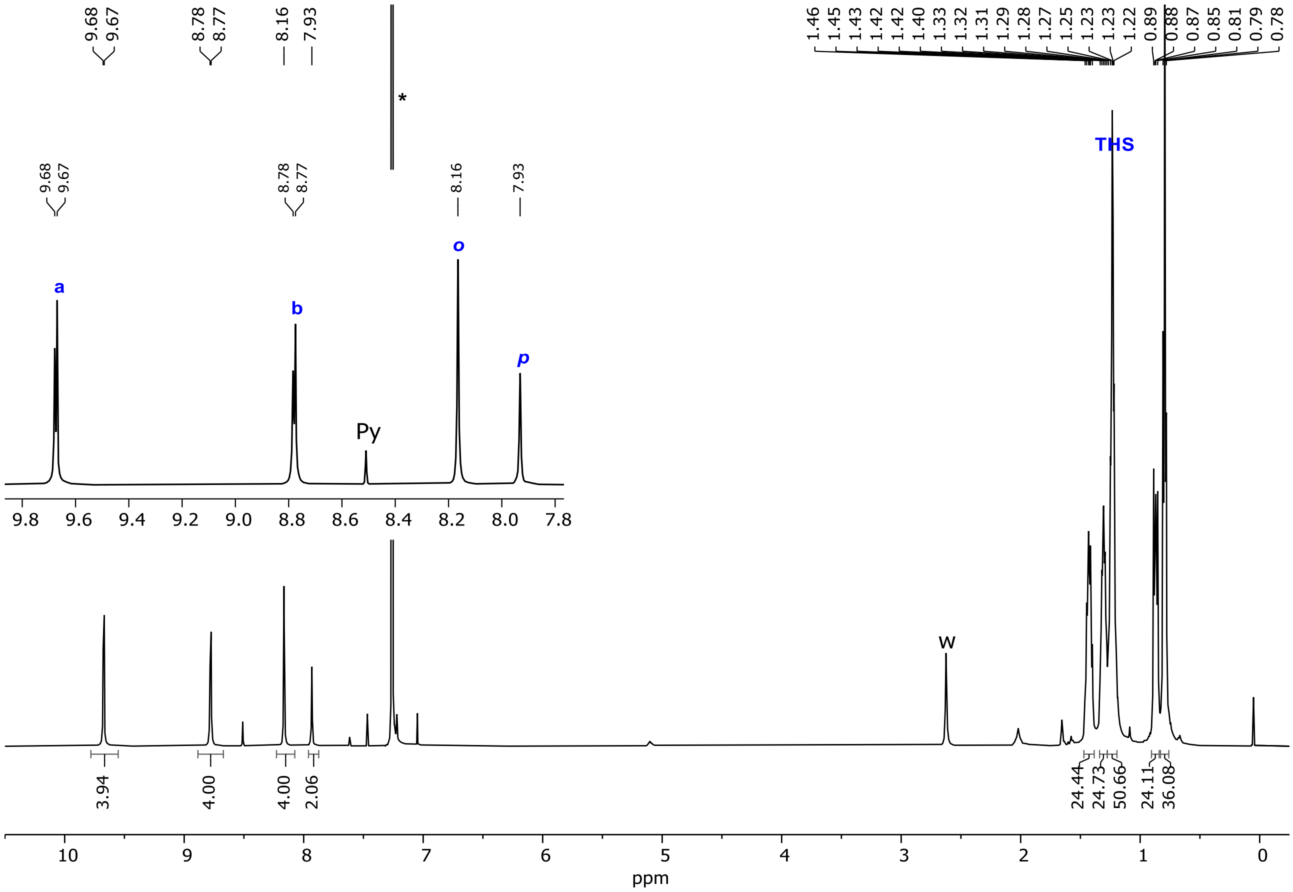

Figure S155. Assigned ^{1}H-NMR spectrum of ***c*-P8**$_{THS}$ (500 MHz, $CDCl_3$ + 5% pyridine-*d*$_5$, 298 K). Abbreviations: * = $CHCl_3$, Py = pyridine, w = water.

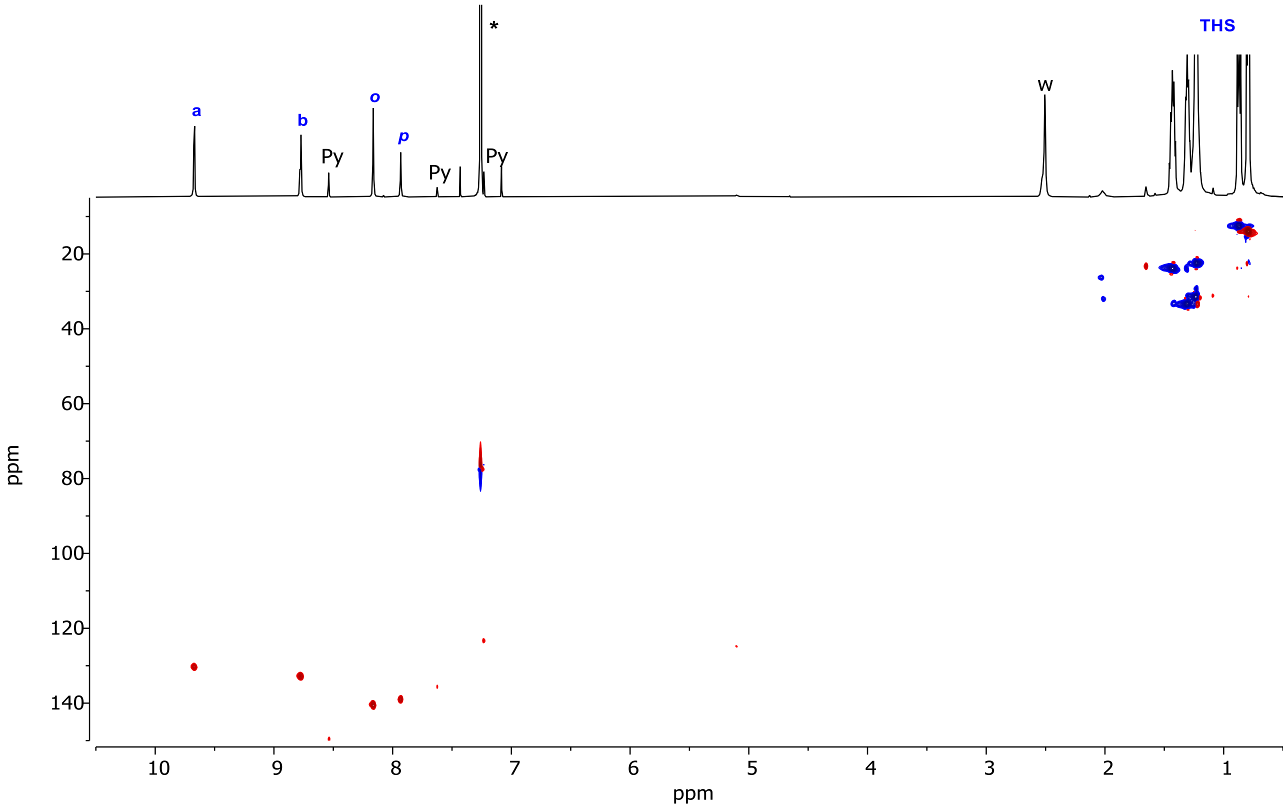

Figure S156. ^{1}H-^{13}C HSQC of ***c*-P8**$_{THS}$ (600 MHz, $CDCl_3$ + 5% pyridine-*d*$_5$, 298 K). Abbreviations: * = $CHCl_3$, Py = pyridine, w = water.

c-P8$_{THS}$ in the absence of pyridine

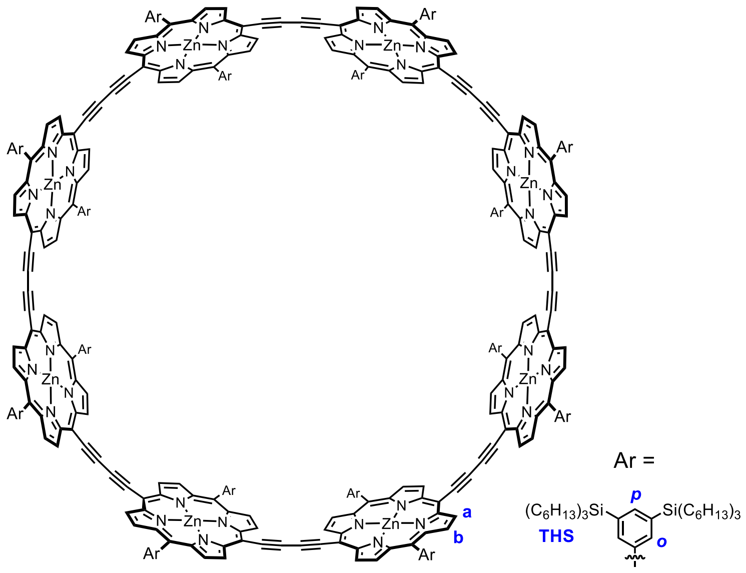

***c*-P8$_{THS}$**

#	Assign.	^{1}H / ppm	Mult.	HSQC
**1**	a	9.90 (4H)	d, *J* = 4.6 Hz	130.3
**2**	b	8.98 (4H)	d, *J* = 4.6 Hz	132.8
**3**	*o*	8.31 (4H)	s	140.4
**4**	*p*	8.02 (2H)	s	138.9
**5**	THS	1.53–1.46 (24H)	m	23.8
**6**	THS	1.40–1.34 (24H)	m	33.4
**7**	THS	1.34–1.26 (48H)	m	31.4, 22.4
**8**	THS	0.98–0.91 (24H)	m	12.5
**9**	THS	0.89–0.83 (36H)	m	14.0

Figure S157. Top: Structure of ***c*-P8$_{THS}$** with labels used for ^{1}H assignment. Bottom: Assigned ^{1}H resonances and ^{13}C chemical shift values for their associated carbon atoms. The number of protons is listed as per porphyrin unit.

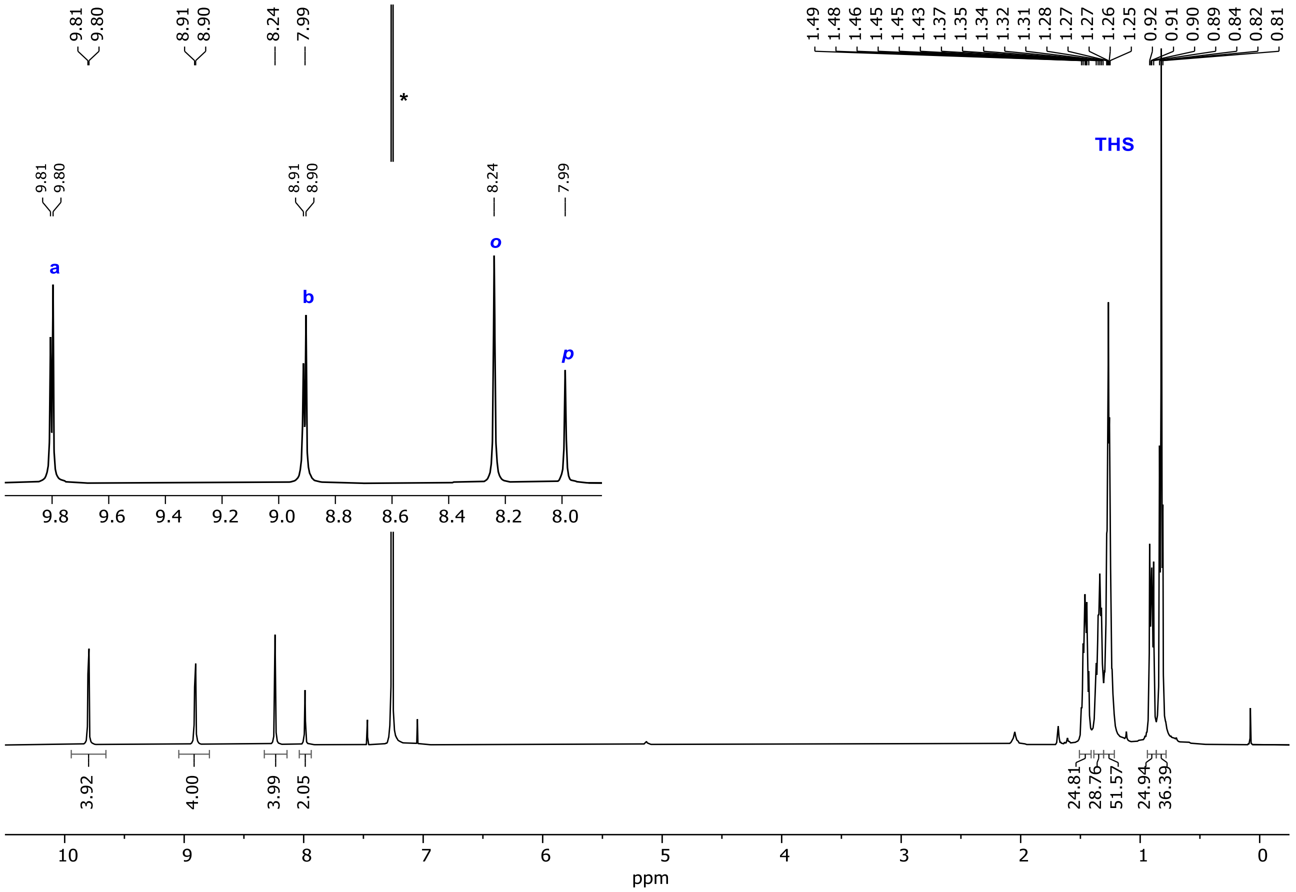

Figure S158. Assigned ^{1}H-NMR spectrum of ***c*-P8**$_{\mathbf{THS}}$ (500 MHz, $CDCl_3$, 298 K). Abbreviations: * = $CHCl_3$.

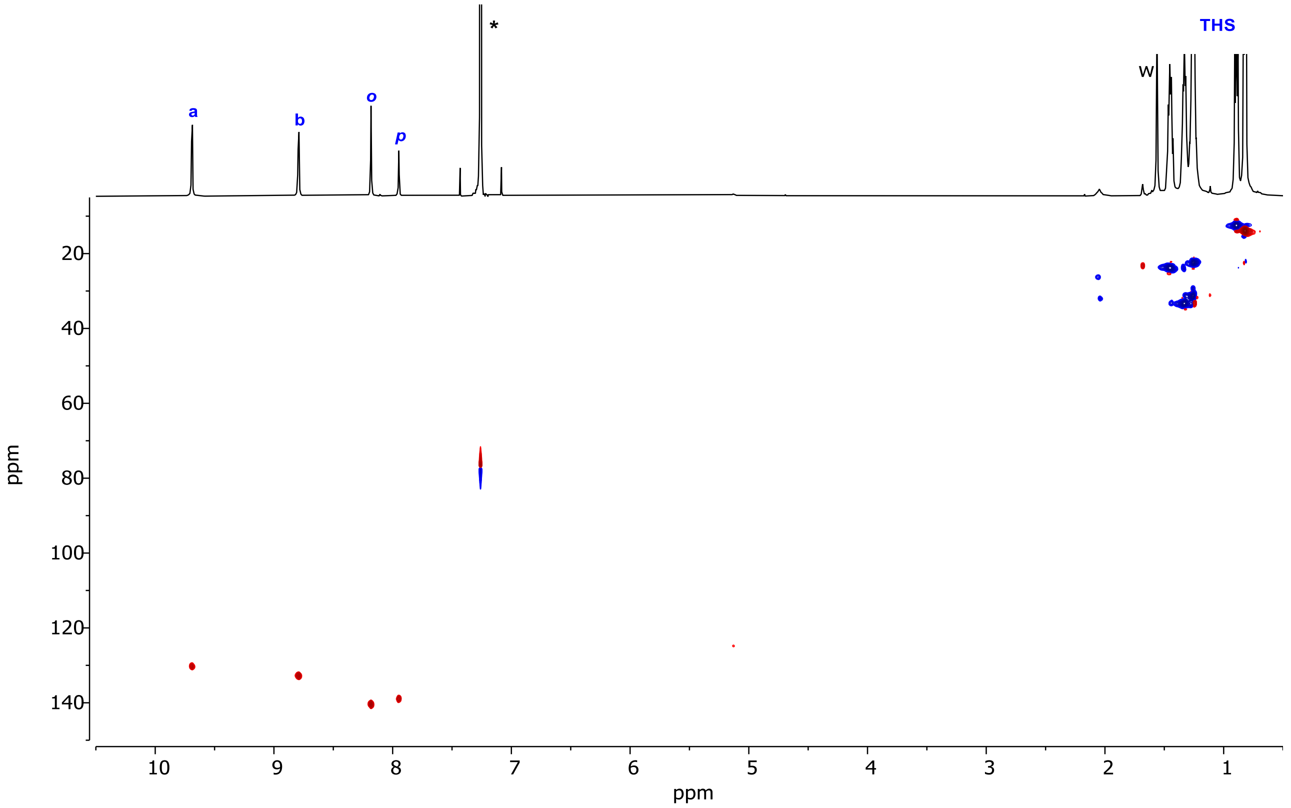

Figure S159. ^{1}H-^{13}C HSQC of ***c*-P8**$_{\mathbf{THS}}$ (600 MHz, $CDCl_3$, 298 K). Abbreviations: * = $CHCl_3$, w = water.

***c*-P12$_{t\text{-Bu}}$ + pyridine-d_5**

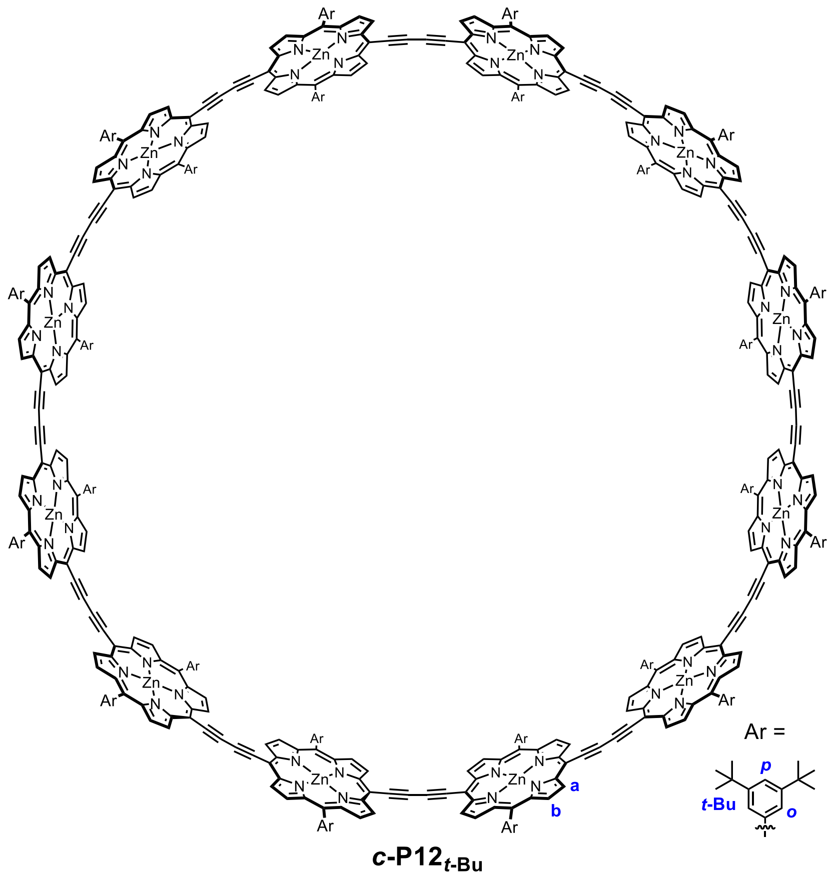

#	Assign.	^{1}H / ppm	Mult.	HSQC
**1**	a	9.83 (4H)	d, *J* = 4.4 Hz	130.4
**2**	b	8.94 (4H)	d, *J* = 4.4 Hz	133.1
**3**	*o*	8.04 (4H)	d, *J* = 1.6 Hz	129.9
**4**	*p*	7.80 (2H)	t, *J* = 1.6 Hz	120.8
**5**	*t*-Bu	1.55 (36H)	s	31.6

Figure S160. Top: Structure of ***c*-P12$_{t\text{-Bu}}$** with labels used for ^{1}H assignment. Bottom: Assigned ^{1}H resonances and ^{13}C chemical shift values for their associated carbon atoms. The number of protons is listed as per porphyrin unit.

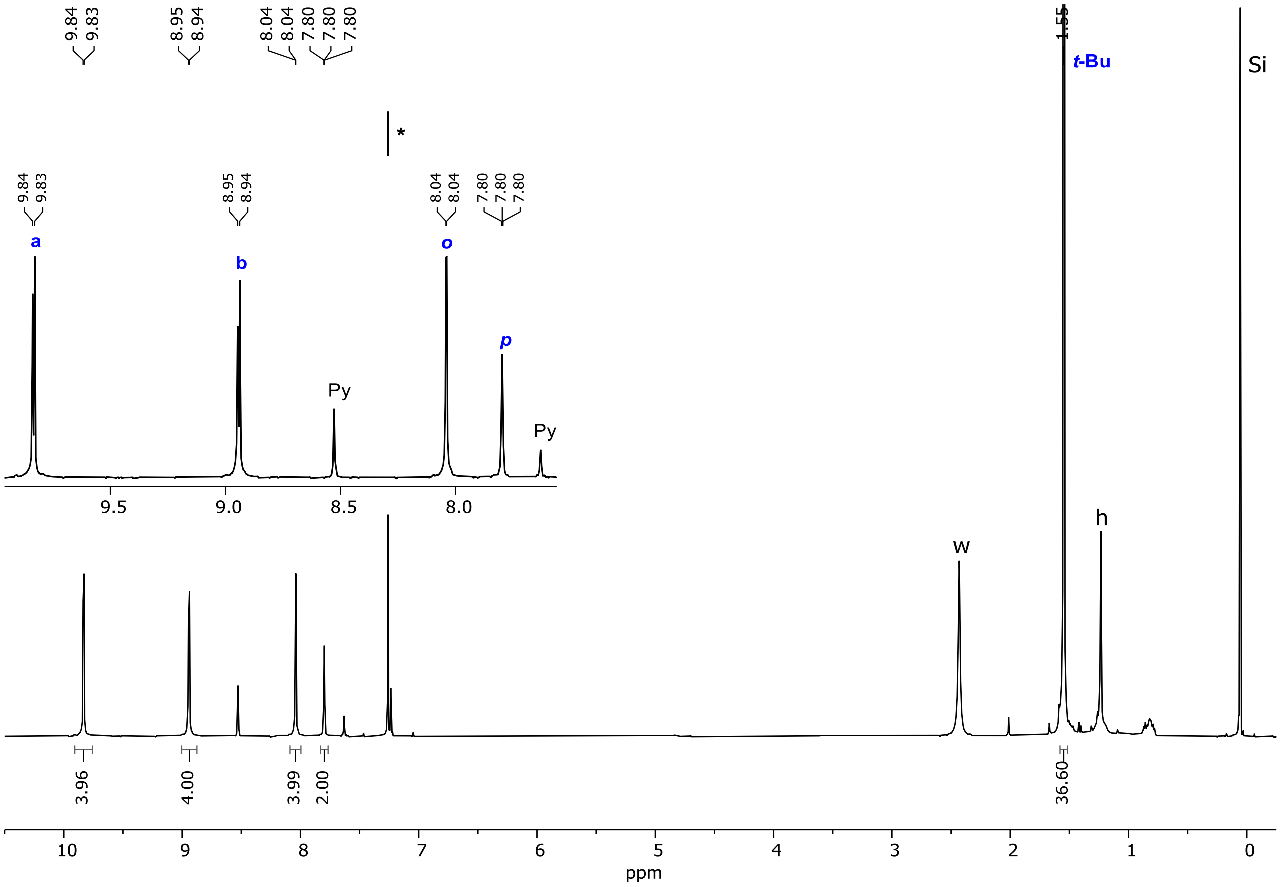

Figure S161. Assigned ^{1}H-NMR spectrum of ***c*-P12$_{t\text{-Bu}}$** (500 MHz, $CDCl_3$ + 5% pyridine-d_5, 298 K). Abbreviations: * = $CHCl_3$, Py = pyridine, w = water, h = H-grease, Si = silicone grease.

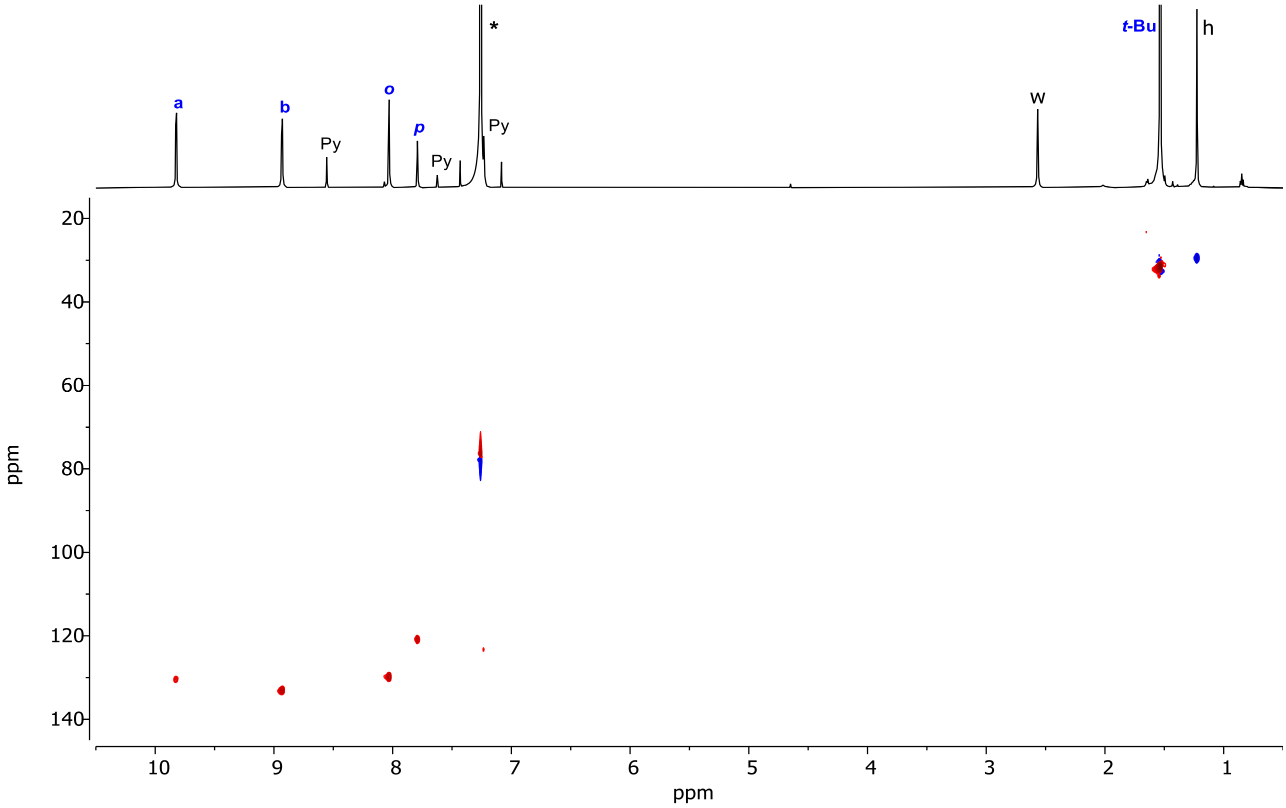

Figure S162. ^{1}H-^{13}C HSQC of ***c*-P12$_{t\text{-Bu}}$** (600 MHz, $CDCl_3$ + 5% pyridine-d_5, 298 K). Abbreviations: * = $CHCl_3$, Py = pyridine, w = water, h = H-grease.

c-P12$_{t\text{-Bu}}$ in the absence of pyridine

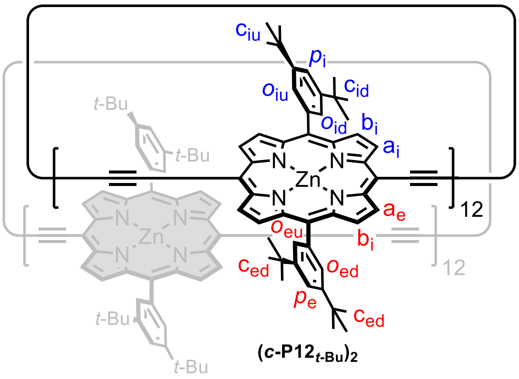

#	Assign.	^{1}H	Δδ	Mult.	COSY	TOCSY	NOESY†	HSQC
**1**	o_{ed}	9.15 (1H)	+1.11	s	5, 7	5, 7	s: 2, 9; m: 5, 7; w: 11	128.9
**2**	b_e	8.95 (2H)	+0.01	br s	-	-	s: 1, 9; m: 7; w: 11; vw: 5	-
**3**	o_{id}	8.78 (1H)	+0.74	s	6, 8	6, 8	s: 4, 8^X, 10; m: 6, 12	129.5
**4**	b_i	8.58 (2H)	–0.36	br s	-	-	s: 3, 8, 10; m: 12; w: 6	-
**5**	p_e	8.11 (1H)	+0.31	s	1, 7	1, 7	s: 9, m: 1, 7, 11; vw: 2	121.5
**6**	p_i	8.03 (1H)	+0.23	s	3, 8	3, 8	s: 10; m: 3, 8, 12; w: 4	121.2
**7**	o_{eu}	7.93 (1H)	–0.11	s	1, 5	1, 5	s: 11; m: 1, 2, 5, 9; vw: 4	129.8
**8**	o_{iu}	7.77 (1H)	–0.27	s	3, 6	3, 6	s: 3^X, 4, 12; m: 6, 10	129.2
**9**	c_{ed}	2.03 (9H)	+0.48	s	-	11	s: 1, 2, 5; m: 7, 11	32.45
**10**	c_{id}	1.84 (9H)	+0.29	br s	-	12	s: 3, 4, 6, 12^X; m: 8	32.52
**11**	c_{eu}	1.64 (9H)	+0.09	s	9	-	s: 7; m: 5, 9; w: 1, 2	31.87
**12**	c_{iu}	1.61 (9H)	+0.06	br s	-	9	s: 8, 10^X; m: 3, 4, 6	31.91

Figure S163. Top: Structure of **(c-P12$_{t\text{-Bu}}$)$_2$** with labels used for ^{1}H assignment. Bottom: Assigned ^{1}H resonances and ^{13}C chemical shift values for their associated carbon atoms. The number of protons is listed as per porphyrin unit. $\Delta\delta = \delta(\text{aggregate}) - \delta(\text{monomer})$. †Relative strengths of NOE correlations: s = strong, m = medium, w = weak, vw = very weak. ^{X}Correlation in NOESY which (via ROESY) was found to stem from chemical exchange rather than a through-space dipolar interaction.

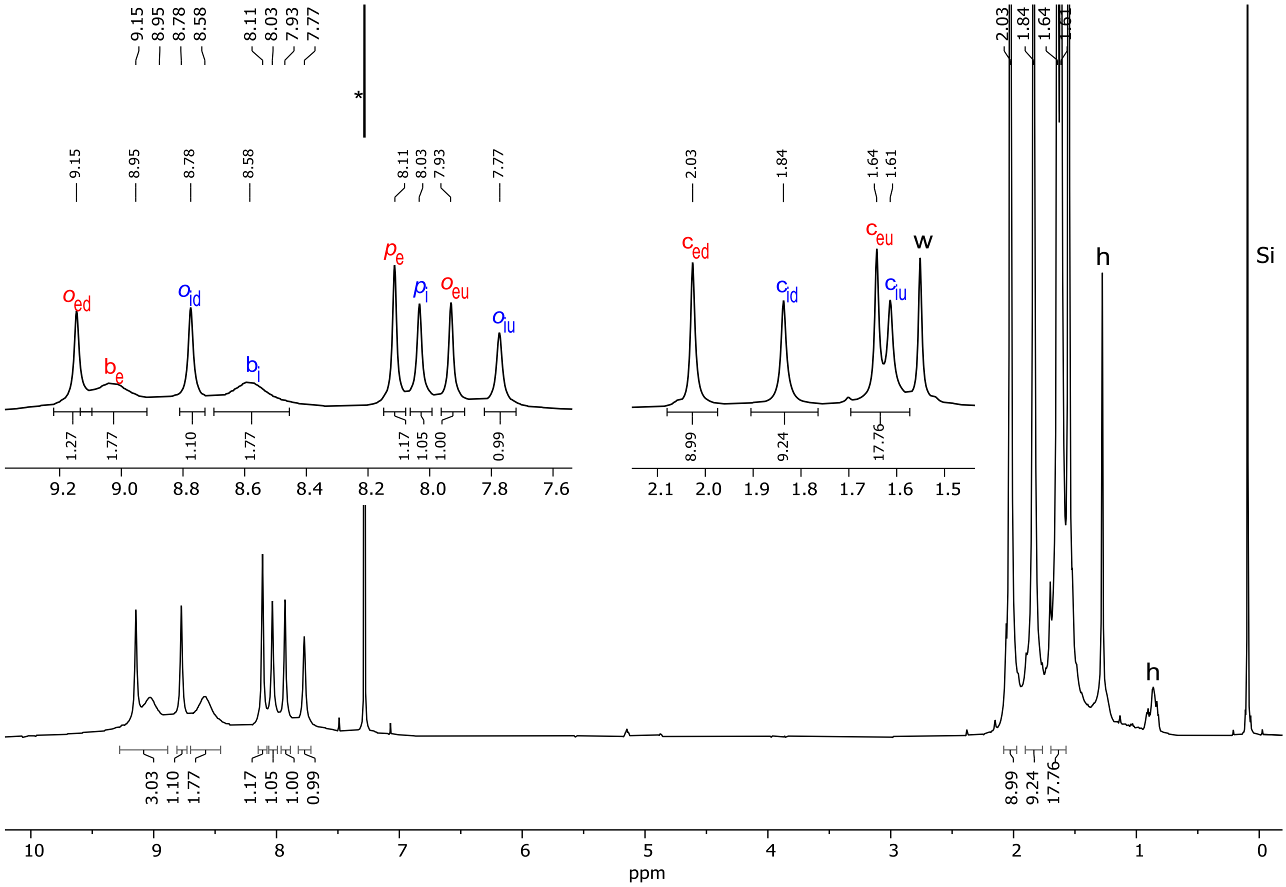

Figure S164. Assigned ^{1}H–NMR spectrum of **(*c*-P12$_{t\text{-Bu}}$)$_2$** (500 MHz, $CDCl_3$, 298 K). Abbreviations: * = $CHCl_3$, w = water, h = H-grease, Si = silicone grease.

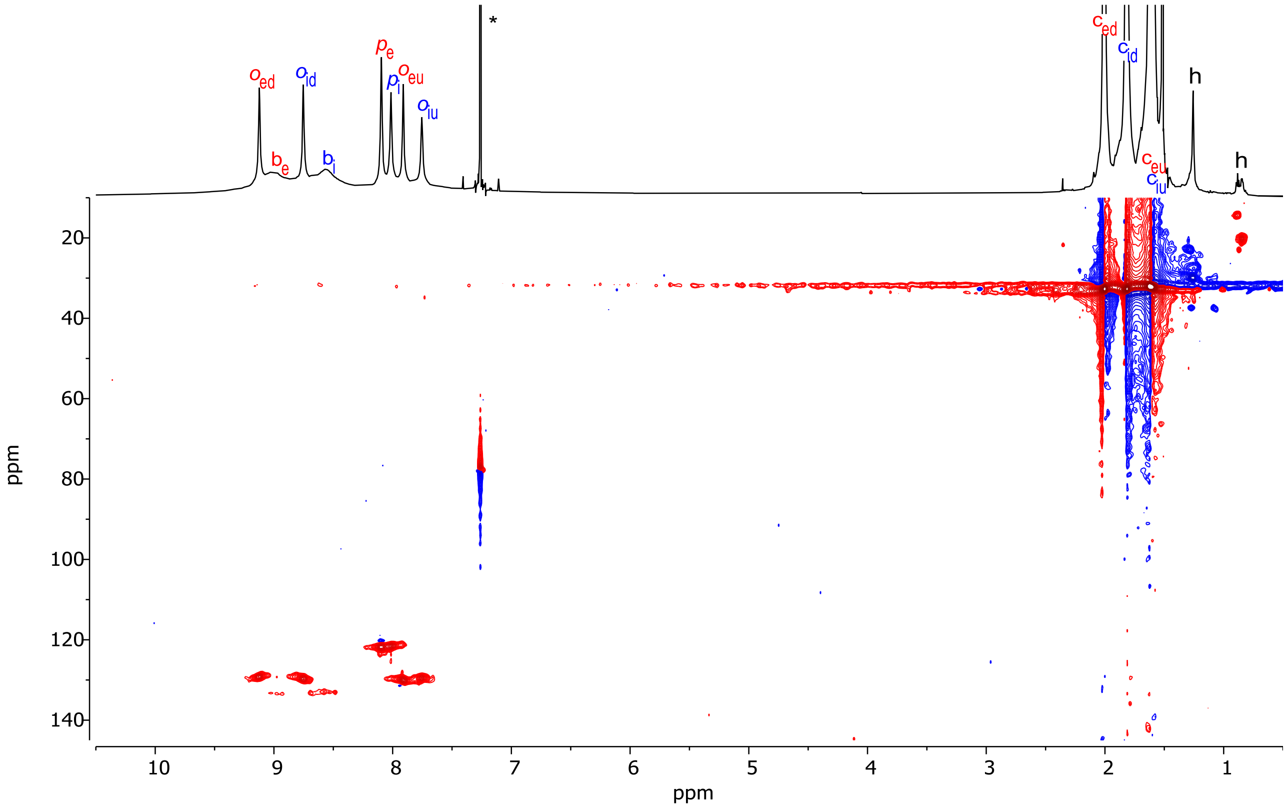

Figure S165. ^{1}H–^{13}C HSQC of **(*c*-P12$_{t\text{-Bu}}$)$_2$** (700 MHz, $CDCl_3$, 298 K). Abbreviations: * = $CHCl_3$, w = water, h = H-grease.

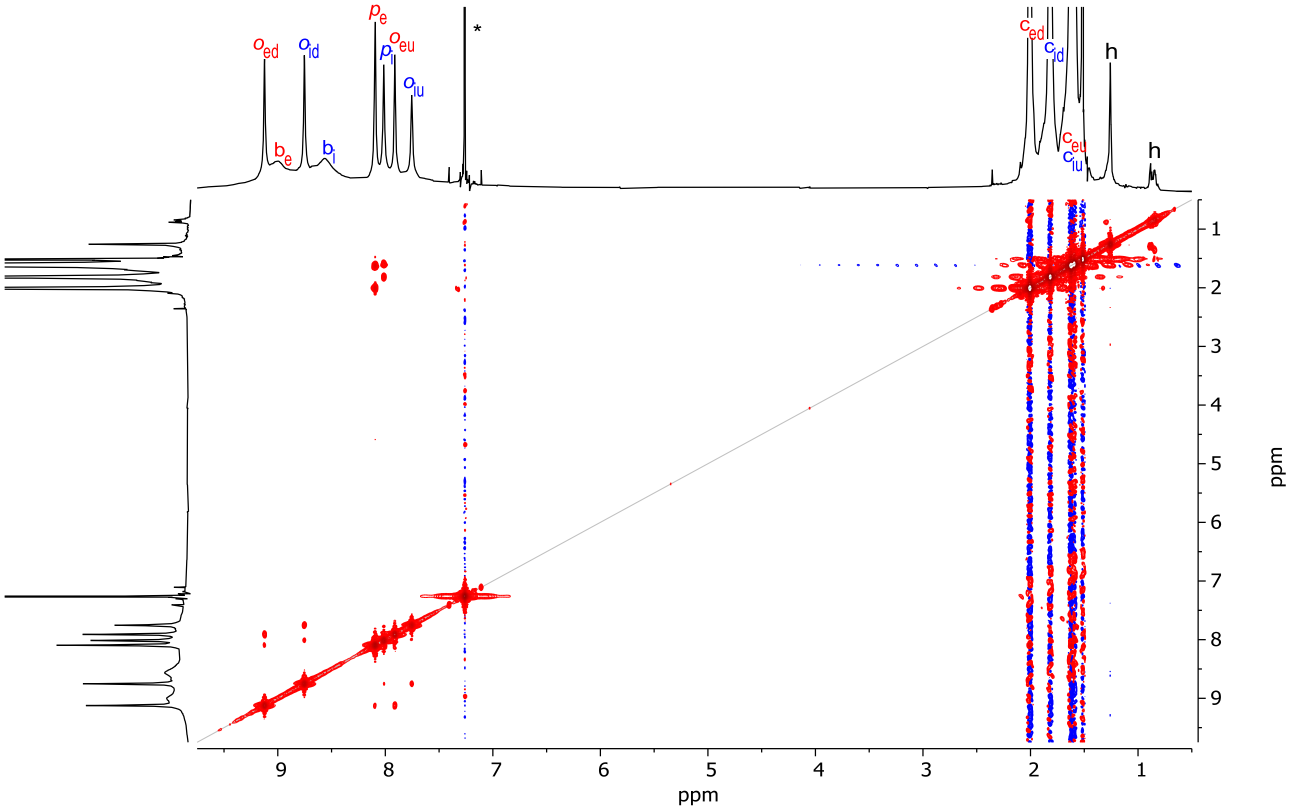

Figure S166. ^{1}H–^{1}H COSY spectrum of **(*c*-P12$_{t\text{-Bu}}$)$_2$** (700 MHz, $CDCl_3$, 298 K). Abbreviations: * = $CHCl_3$, w = water, h = H-grease.

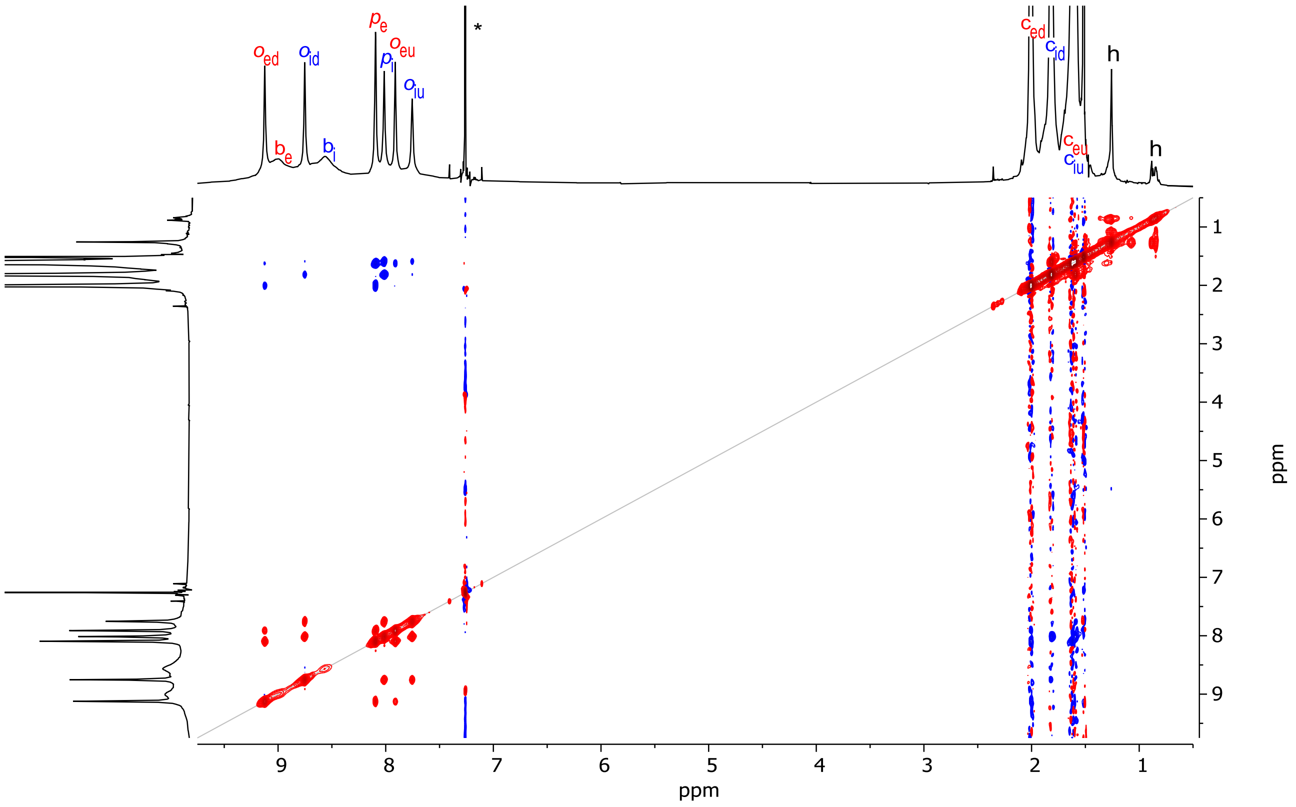

Figure S167. ^{1}H–^{1}H TOCSY spectrum of **(*c*-P12$_{t\text{-Bu}}$)$_2$** (700 MHz, $CDCl_3$, 298 K). Abbreviations: * = $CHCl_3$, w = water, h = H-grease.

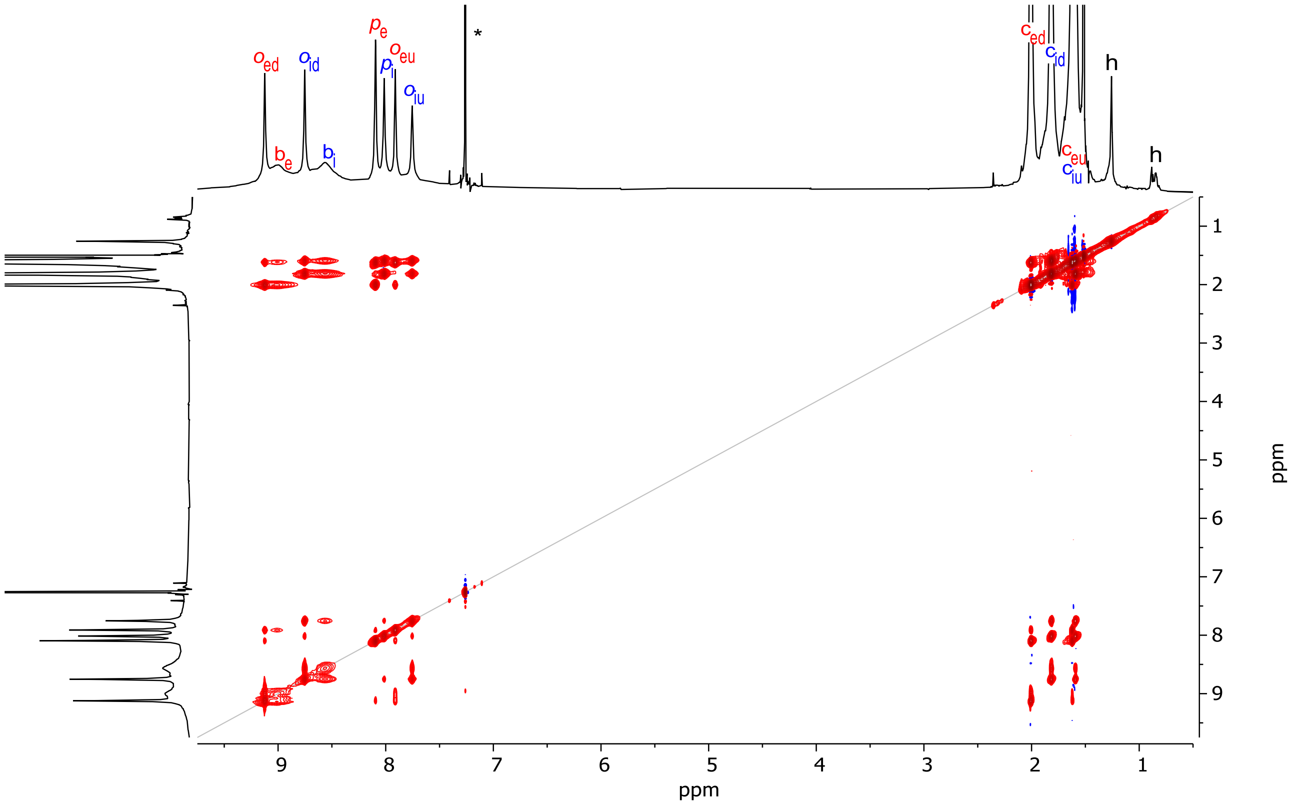

Figure S168. ^{1}H–^{1}H NOESY spectrum of **(*c*-P12$_{t\text{-Bu}}$)$_2$** (700 MHz, $CDCl_3$, 298 K, t_{mix} = 400 ms). Abbreviations: * = $CHCl_3$, w = water, h = H-grease.

c-P12$_{OOct}$ + pyridine-d_5

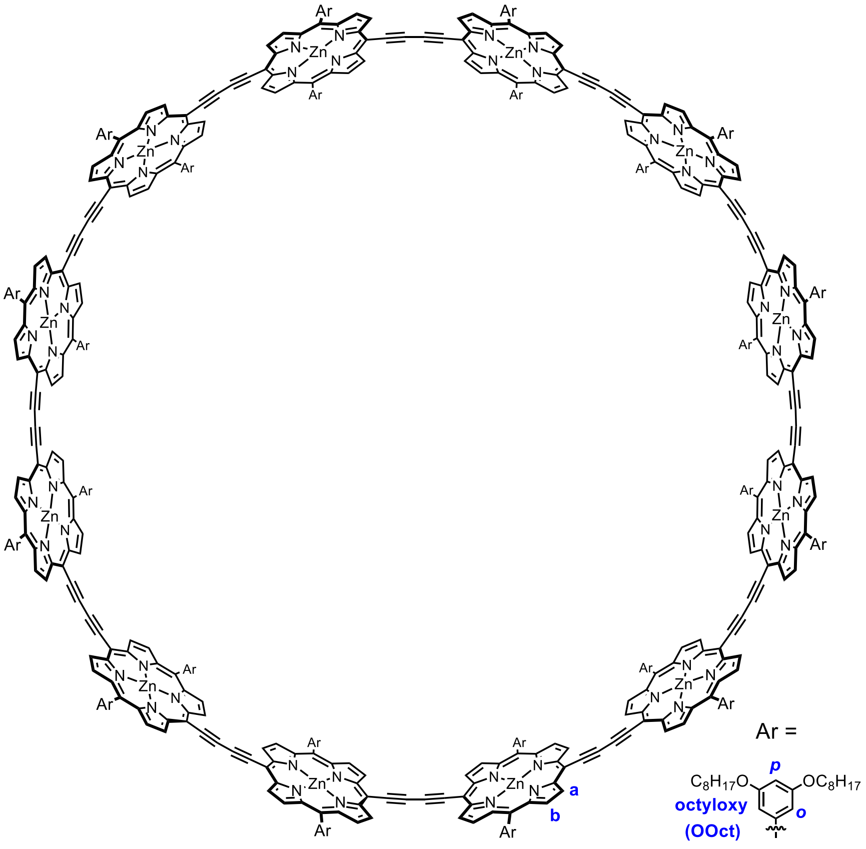

#	Assign.	^{1}H / ppm	Mult.	HSQC
1	a	9.80 (4H)	d, J = 4.5 Hz	130.5
2	b	9.01 (4H)	d, J = 4.5 Hz	132.9
3	*o*	7.35 (4H)	d, J = 1.7 Hz	114.3
4	*p*	6.92–6.86 (2H)	m	100.6
5	OOct (OCH2)	4.21–4.07 (8H)	m	68.2
6	OOct	1.92–1.81 (8H)	m	29.2
7	OOct	1.55–1.45 (8H)	m	25.9
8	OOct	1.39–1.20 (32H)	m	29.2, 29.1, 22.4, 31.6
9	OOct	0.85–0.78 (12H)	m	13.9

Figure S169. Top: Structure of ***c*-P12$_{OOct}$** with labels used for ^{1}H assignment. Bottom: Assigned ^{1}H resonances and ^{13}C chemical shift values for their associated carbon atoms. The number of protons is listed as per porphyrin unit.

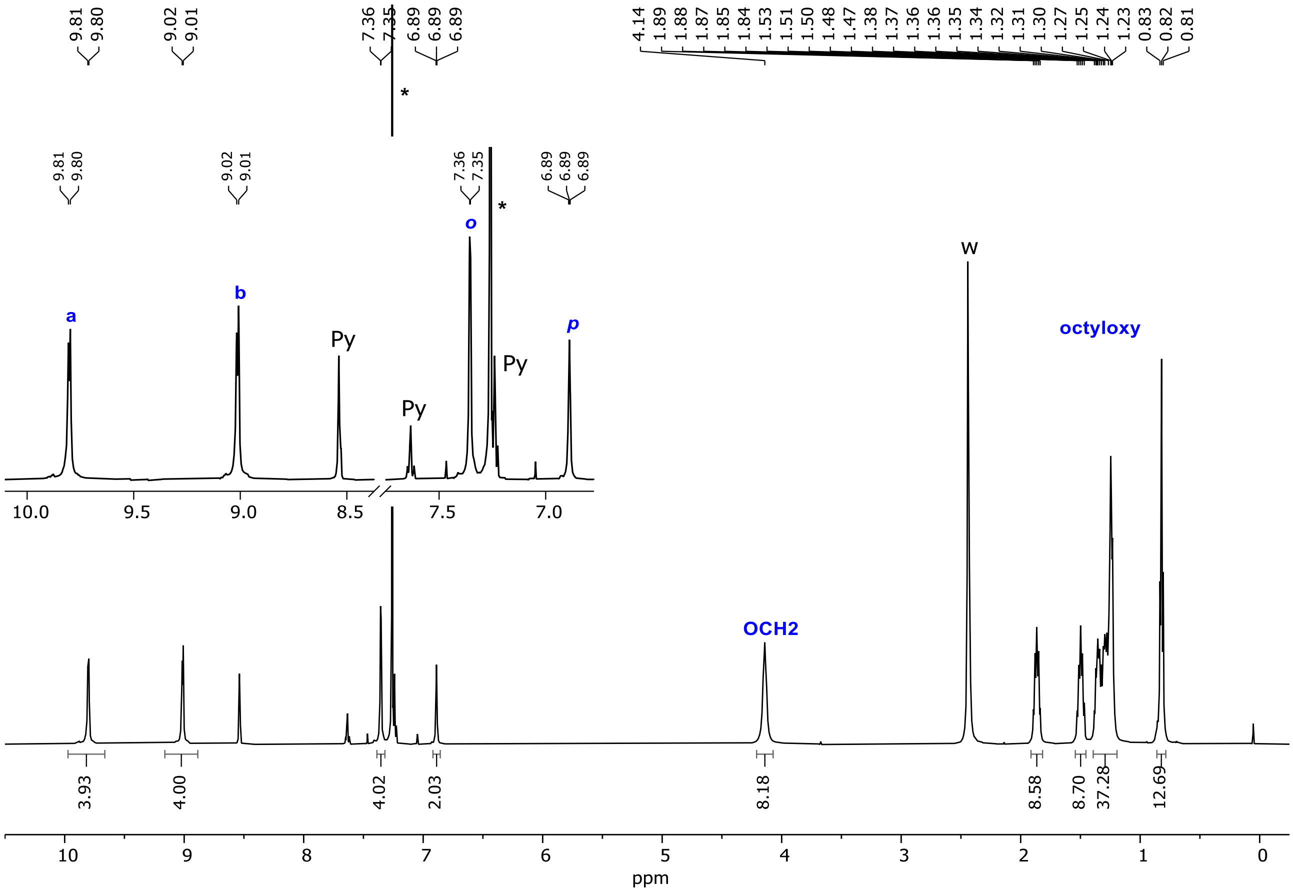

Figure S170. Assigned ^{1}H-NMR spectrum of ***c*-P12$_{\mathbf{OOct}}$** (500 MHz, $CDCl_3$ + 5% pyridine-d_5, 298 K). Abbreviations: * = $CHCl_3$, Py = pyridine, w = water.

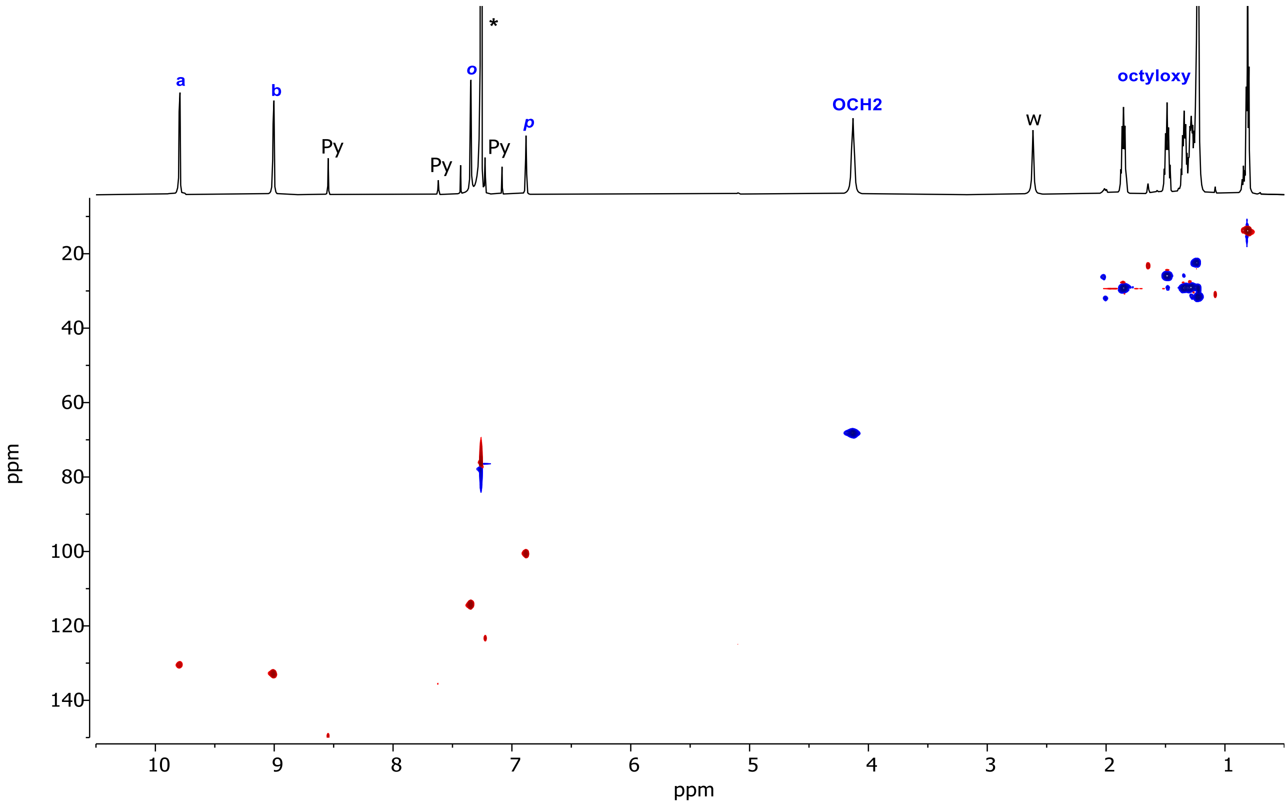

Figure S171. ^{1}H-^{13}C HSQC of ***c*-P12$_{\mathbf{OOct}}$** (600 MHz, $CDCl_3$ + 5% pyridine-d_5, 298 K). Abbreviations: * = $CHCl_3$, Py = pyridine, w = water.

c-P12$_{OOct}$ in the absence of pyridine

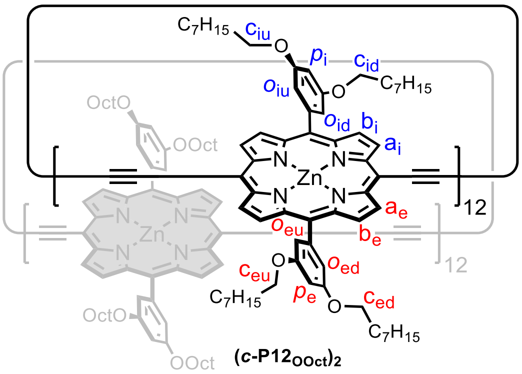

#	Assign.	^{1}H / ppm	Mult.	COSY	TOCSY	NOESY†	HSQC
1	b_e	9.00 (2H)	s	-	-	s: 2, 3; m: 7, 10; w: 6, 12, 14	132.3
2	a_e + b_i	8.89–8.56 (4H)	m	-	-	s: 1, 4, 5, 9; m: 11; w: 3, 8, 13	132.2
3	o_{ed}	8.40 (1H)	s	6, 7	6, 7	s: 1, 10; w: 2, 6, 7, 14, 16	113.9
4	a_i	8.38–8.14 (2H)	m	-	-	s: 2; m: 5	-
5	o_{id}	8.05 (1H)	s	8, 9	8, 9	s: 2, 11; m: 4; w: 8, 9^X, 13, 15	114.4
6	p_e	7.13 (1H)	s	3, 7	3, 7	s: 10, 12; m: 14; w: 1, 3, 7, 16	101.6
7	o_{eu}	6.98 (1H)	s	3, 6	3, 6	s: 12; m: 1; w: 3, 6, 10, 16	114.4
8	p_i	6.94 (1H)	s	5, 9	5, 9	m: 11, 13, 15; w: 2, 5, 9, 16	101.3
9	o_{iu}	6.69 (1H)	s	5, 8	5, 8	s: 2, 13; w: 5^X, 8, 11, 16	114.5
10	c_{ed}	4.71–4.57 (2H)	m	14	14, 16	s: 3, 6, 14; m: 1, 16; w: 7, 12	69.0
11	c_{id}	4.48–4.29 (2H)	m	15	15, 16	s: 5, 15; m: 2, 8, 13, 16; w: 9	68.7
12	c_{eu}	4.15–4.02 (2H)	m	16	16	s: 6, 7; m: 16; w: 1, 10, 14	68.3
13	c_{iu}	3.97–3.80 (2H)	m	16	16	s: 9; m: 8, 11, 16; w: 2, 5, 15	68.1
14	OOct	2.34–2.18 (4H)	m	10, 16	10	s: 10, 16; m: 6; w: 1, 3, 12	30.0, 30.0
15	OOct	2.19–2.07 (2H)	m	11, 16	11	s: 11, 16; m: 8; w: 5, 13	29.9
16	OOct	1.93–1.03 (42H)	m	12, 13, 14, 15, 17, 18, 19	10, 11, 12, 13, 17, 18, 19	s: 14, 15; m: 10, 11, 12, 13; w: 3, 6, 7, 8, 9	29.4, 26.6, 26.5, 29.5, 29.9, 29.8, 29.5, 29.5, 26.1, 32.0, 22.7, 32.0, 29.4, 26.0, 22.7, 29.4, 31.8, 29.3, 29.1, 22.5, 31.6
17	OOct (-CH_3)	0.90–0.86 (3H)	m	16	16	n/a	14.2
18	OOct (-CH_3)	0.85–0.77 (6H)	m	16	16	n/a	14.1, 14.1
19	OOct (-CH_3)	0.72–0.63 (3H)	m	16	16	n/a	13.9

Figure S172. Top: Structure of **(*c*-P12$_{OOct}$)$_2$** with labels used for ^{1}H assignment. Bottom: Assigned ^{1}H resonances and ^{13}C chemical shift values for their associated carbon atoms. The number of protons is listed as per porphyrin unit. †Relative strengths of NOE correlations: s = strong, m = medium, w = weak, vw = very weak. ^{X}Correlation in NOESY which (by ROESY) was found to stem from chemical exchange rather than a through-space dipolar interaction.

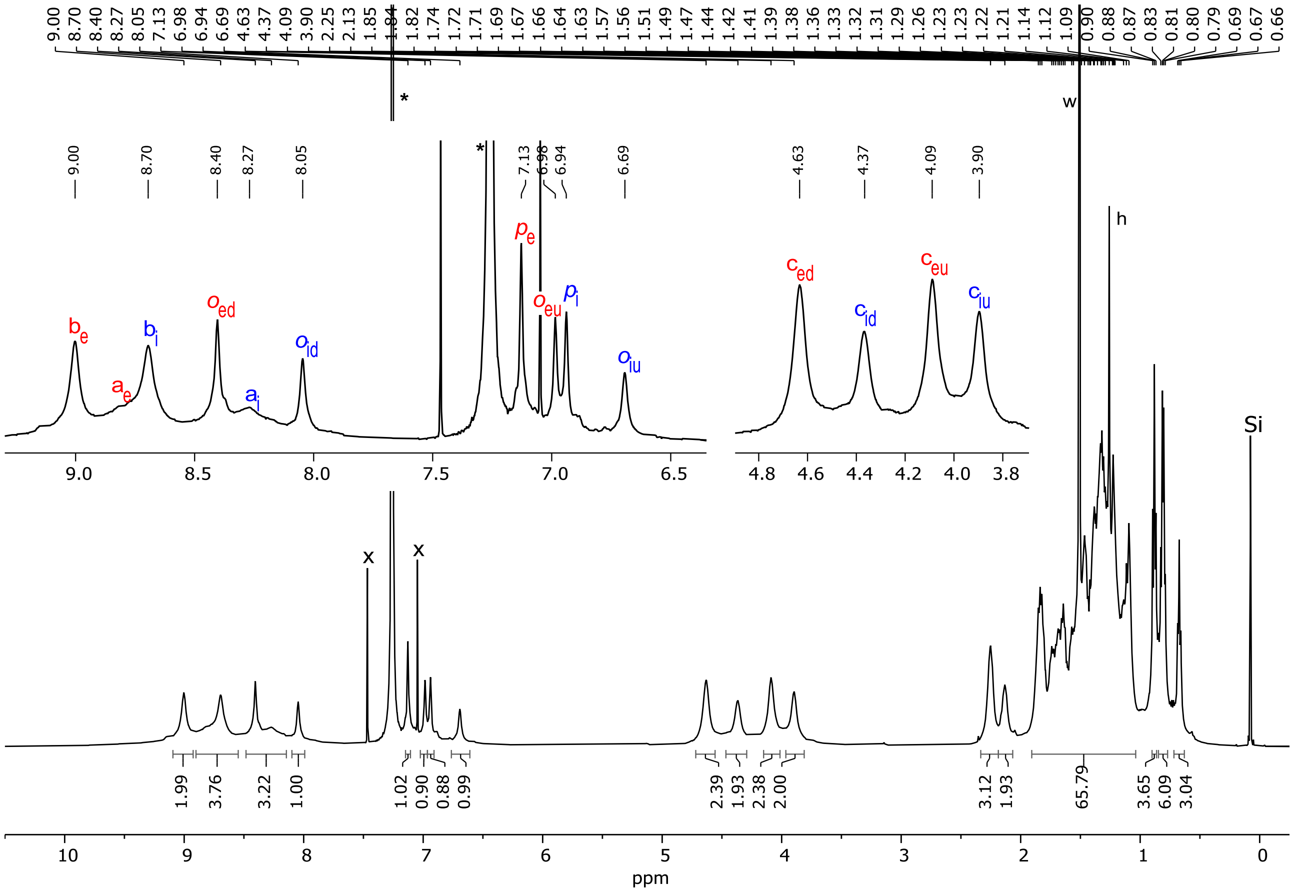

Figure S173. Assigned ^{1}H-NMR spectrum of **(*c*-P12$_{OOct}$)$_2$** (500 MHz, $CDCl_3$, 298 K). Abbreviations: * = $CHCl_3$, w = water, h = H-grease, Si = silicone grease, x = satellite signals from $^{13}CHCl_3$.

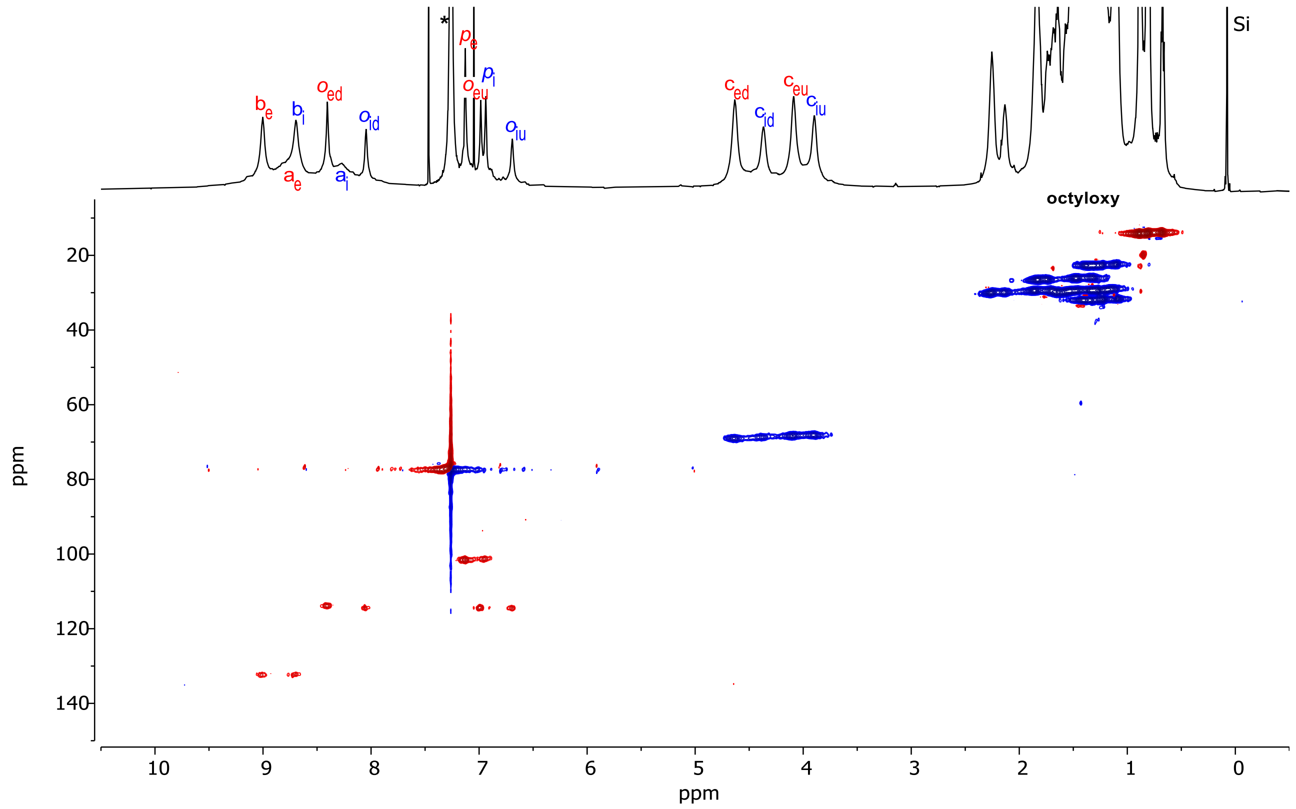

Figure S174. ^{1}H–^{13}C HSQC of **(*c*-P12$_{OOct}$)$_2$** (600 MHz, $CDCl_3$, 298 K). Abbreviations: * = $CHCl_3$, w = water, h = H-grease, Si = silicone grease.

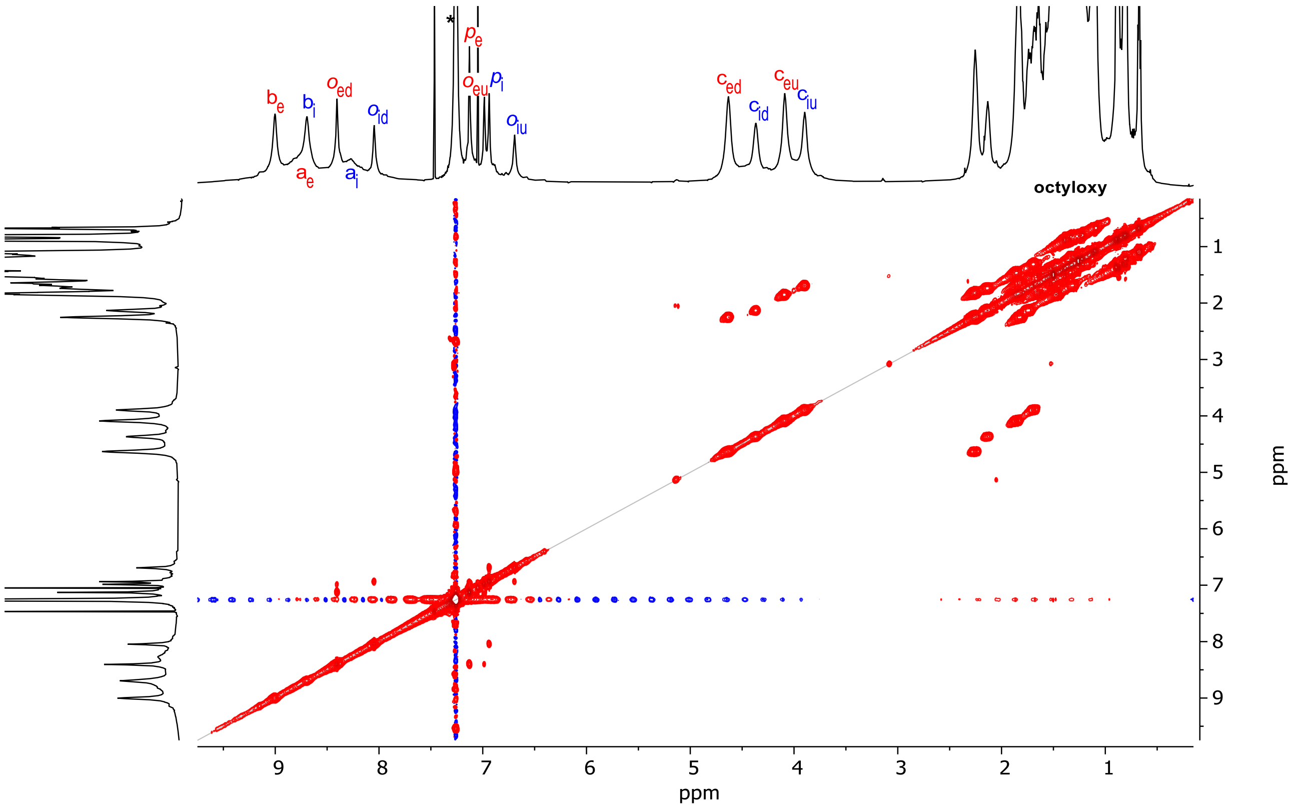

Figure S175. ^1H–^1H COSY spectrum of **(*c*-P12$_{OOct}$)$_2$** (500 MHz, $CDCl_3$, 298 K). Abbreviations: * = $CHCl_3$, w = water, h = H-grease, Si = silicone grease.

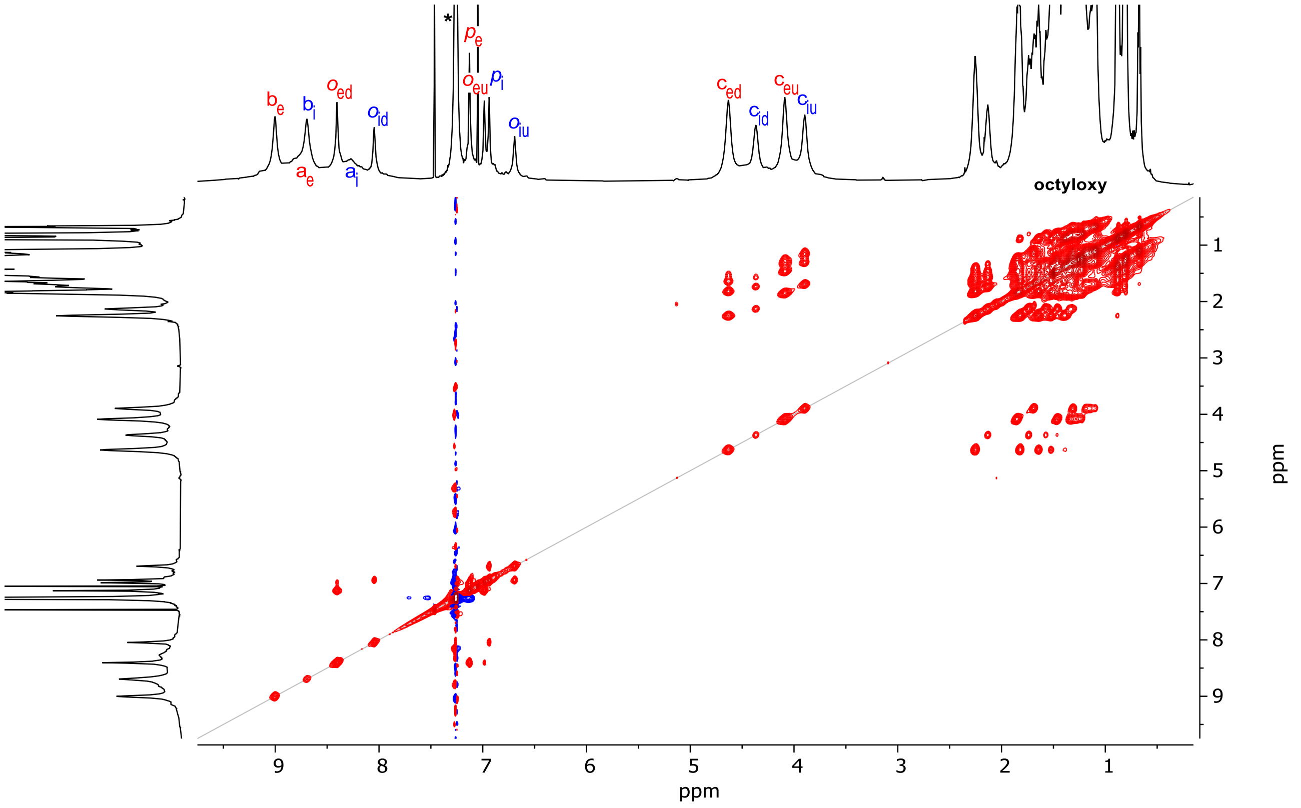

Figure S176. ^1H–^1H TOCSY spectrum of **(*c*-P12$_{OOct}$)$_2$** (500 MHz, $CDCl_3$, 298 K). Abbreviations: * = $CHCl_3$, w = water, h = H-grease, Si = silicone grease.

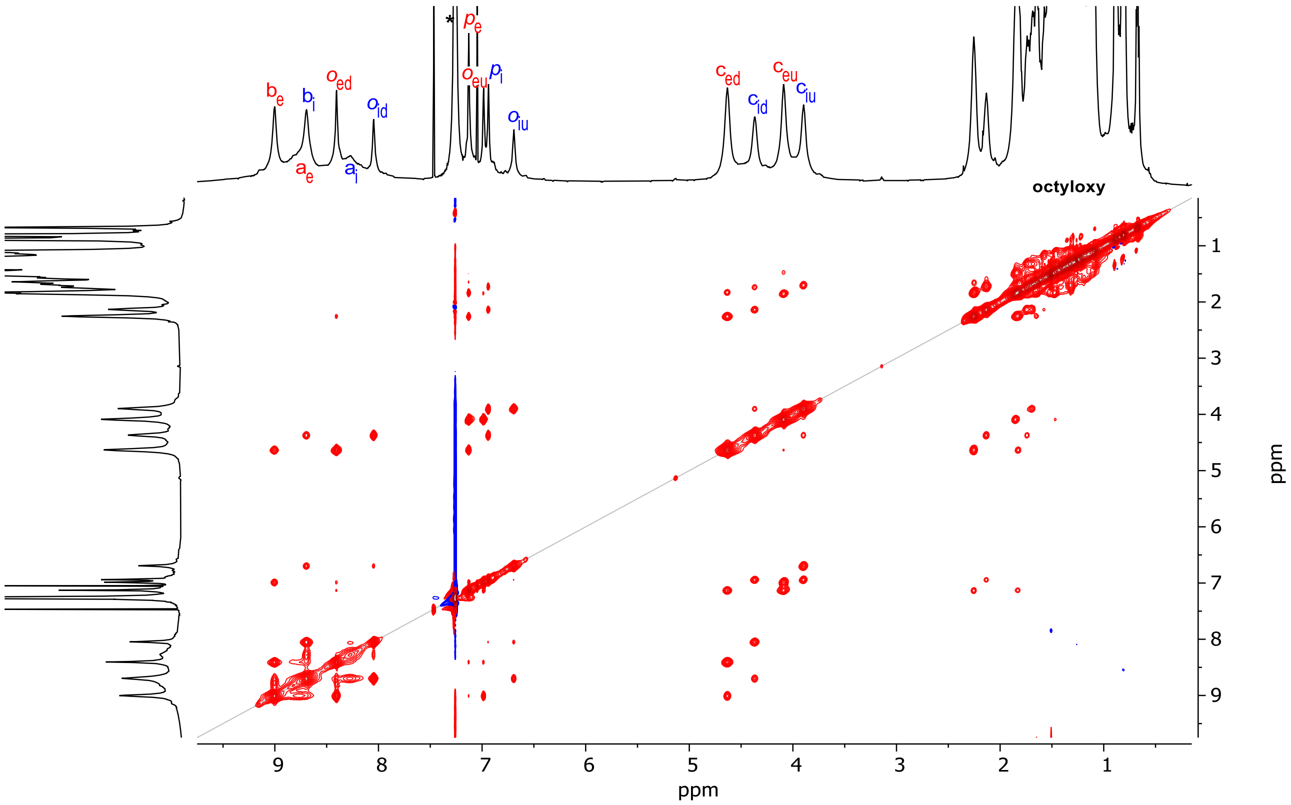

Figure S177. ^{1}H–^{1}H NOESY spectrum of **(*c*-P12$_{OOct}$)$_2$** (500 MHz, CDCl$_3$, 298 K, t_{mix} = 200 ms). Abbreviations: * = CHCl$_3$, w = water, h = H-grease, Si = silicone grease.

c-P12$_{THS}$ + pyridine-d_5

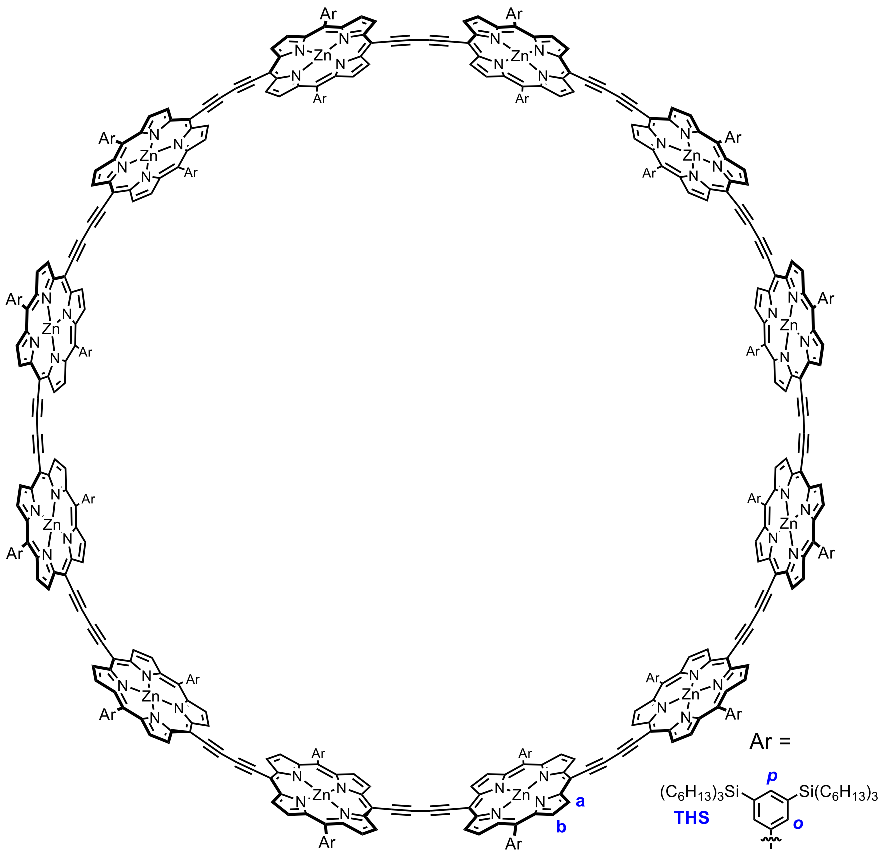

***c*-P12$_{THS}$**

#	Assign.	^{1}H / ppm	Mult.	HSQC
**1**	a	9.79 (4H)	d, *J* = 4.5 Hz	130.5
**2**	b	8.87 (4H)	d, *J* = 4.5 Hz	132.9
**3**	*o*	8.25 (4H)	s	140.6
**4**	*p*	7.98 (2H)	s	139.0
**5**	THS	1.52–1.44 (24H)	m	23.9
**6**	THS	1.40–1.32 (24H)	m	33.4
**7**	THS	1.33–1.22 (48H)	m	31.5, 22.5
**8**	THS	0.96–0.90 (24H)	m	12.6
**9**	THS	0.87 – 0.81 (36H)	m	14.0

Figure S178. Top: Structure of ***c*-P12$_{THS}$** with labels used for ^{1}H assignment. Bottom: Assigned ^{1}H resonances and ^{13}C chemical shift values for their associated carbon atoms. The number of protons is listed as per porphyrin unit.

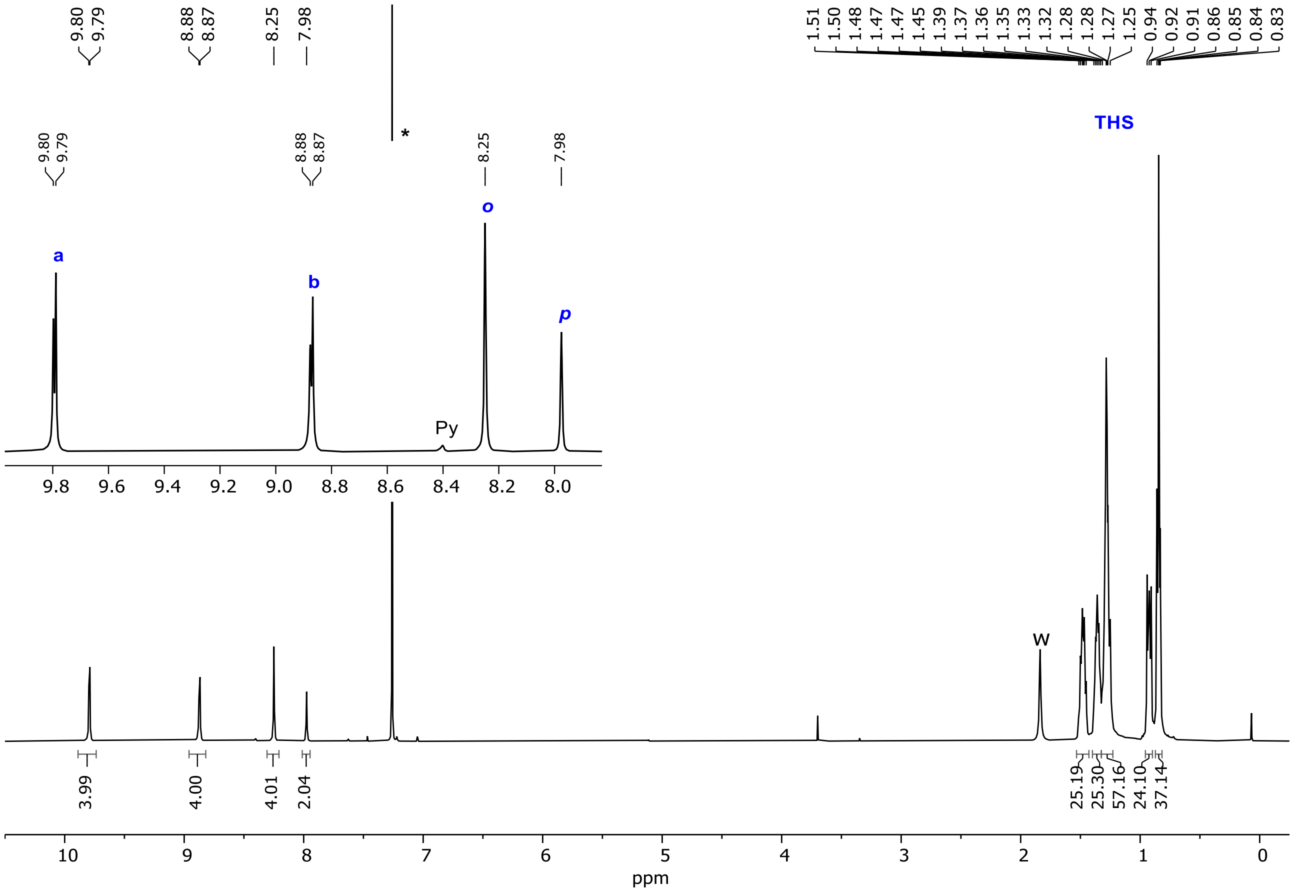

Figure S179. Assigned ^{1}H-NMR spectrum of ***c*-P12**$_{\mathbf{THS}}$ (500 MHz, $CDCl_3$ + 5% pyridine-d_5, 298 K). Abbreviations: * = $CHCl_3$, Py = pyridine, w = water.

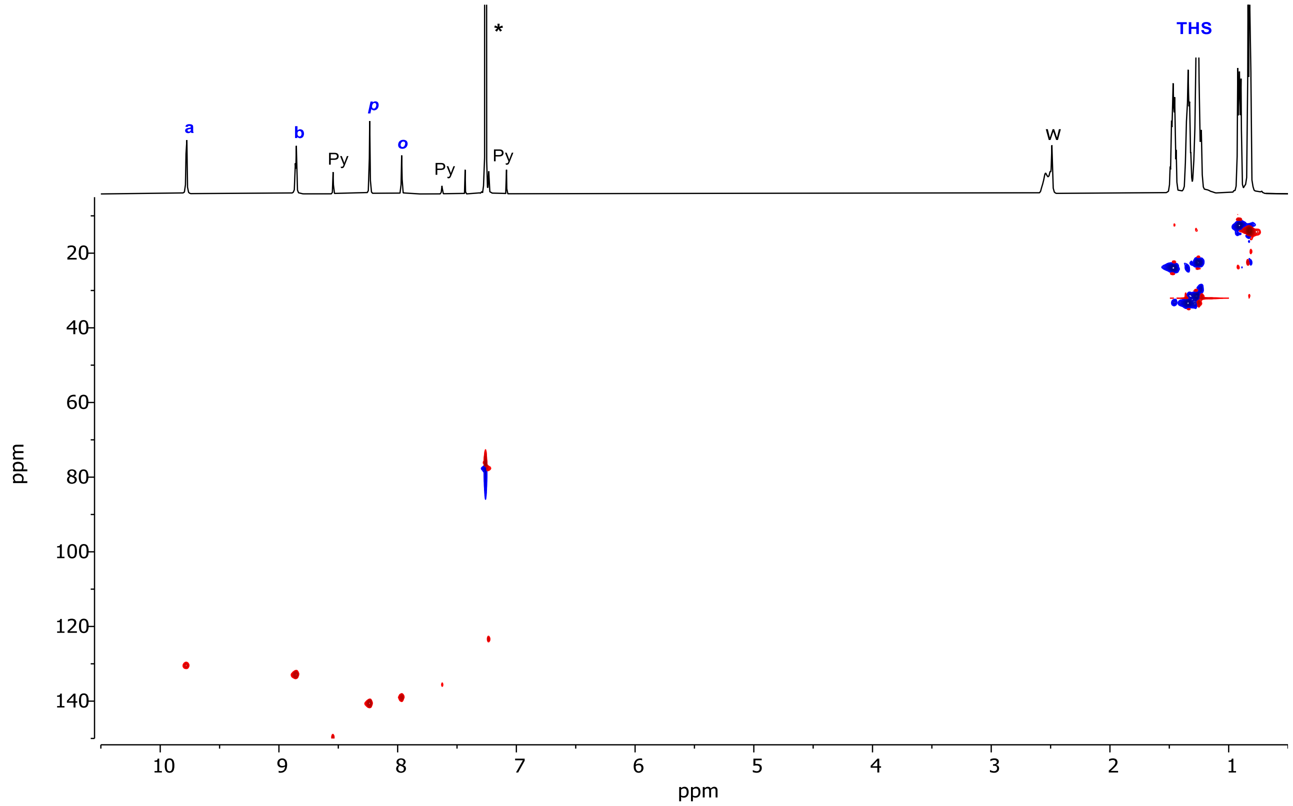

Figure S180. ^{1}H-^{13}C HSQC of ***c*-P12**$_{\mathbf{THS}}$ (600 MHz, $CDCl_3$ + 5% pyridine-d_5, 298 K). Abbreviations: * = $CHCl_3$, Py = pyridine, w = water.

c-P12$_{THS}$ in the absence of pyridine

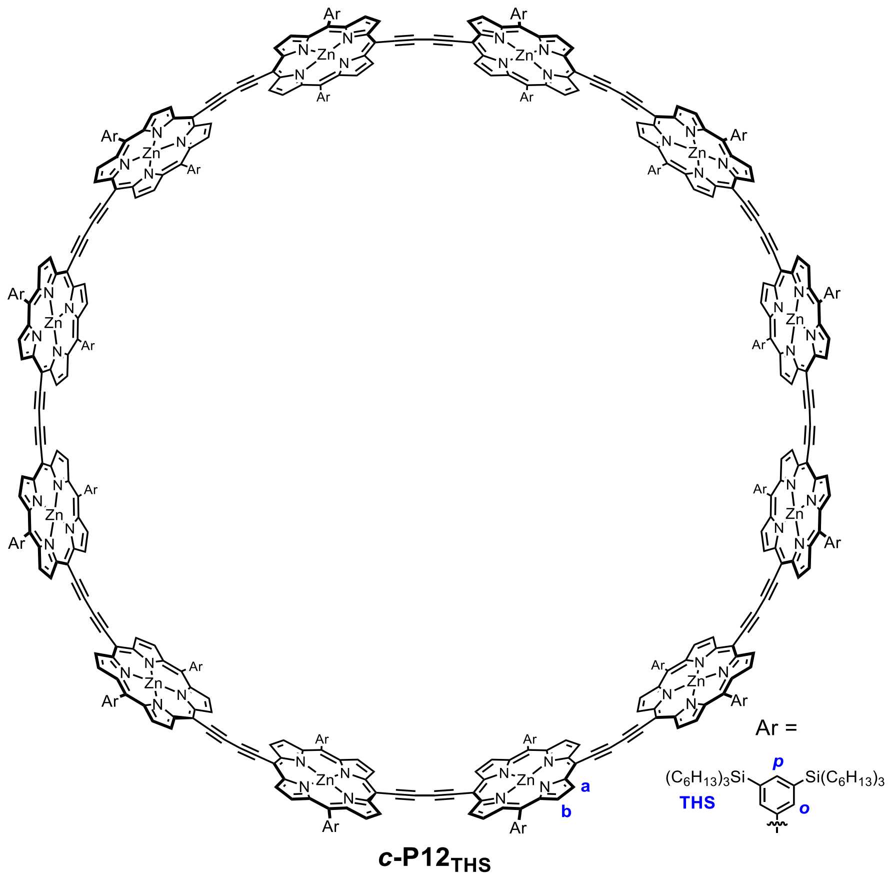

#	Assign.	^{1}H / ppm	Mult.	HSQC
**1**	a	9.90 (4H)	d, *J* = 4.6 Hz	130.6
**2**	b	8.98 (4H)	d, *J* = 4.6 Hz	133.0
**3**	*o*	8.31 (4H)	s	140.5
**4**	*p*	8.02 (2H)	s	139.1
**5**	THS	1.53–1.46 (24H)	m	23.9
**6**	THS	1.40–1.34 (24H)	m	33.4
**7**	THS	1.34–1.26 (48H)	m	31.4, 22.5
**8**	THS	0.98–0.91 (24H)	m	12.5
**9**	THS	0.89–0.83 (36H)	m	14.0

Figure S181. Top: Structure of ***c*-P12$_{THS}$** with labels used for ^{1}H assignment. Bottom: Assigned ^{1}H resonances and ^{13}C chemical shift values for their associated carbon atoms. The number of protons is listed as per porphyrin unit.

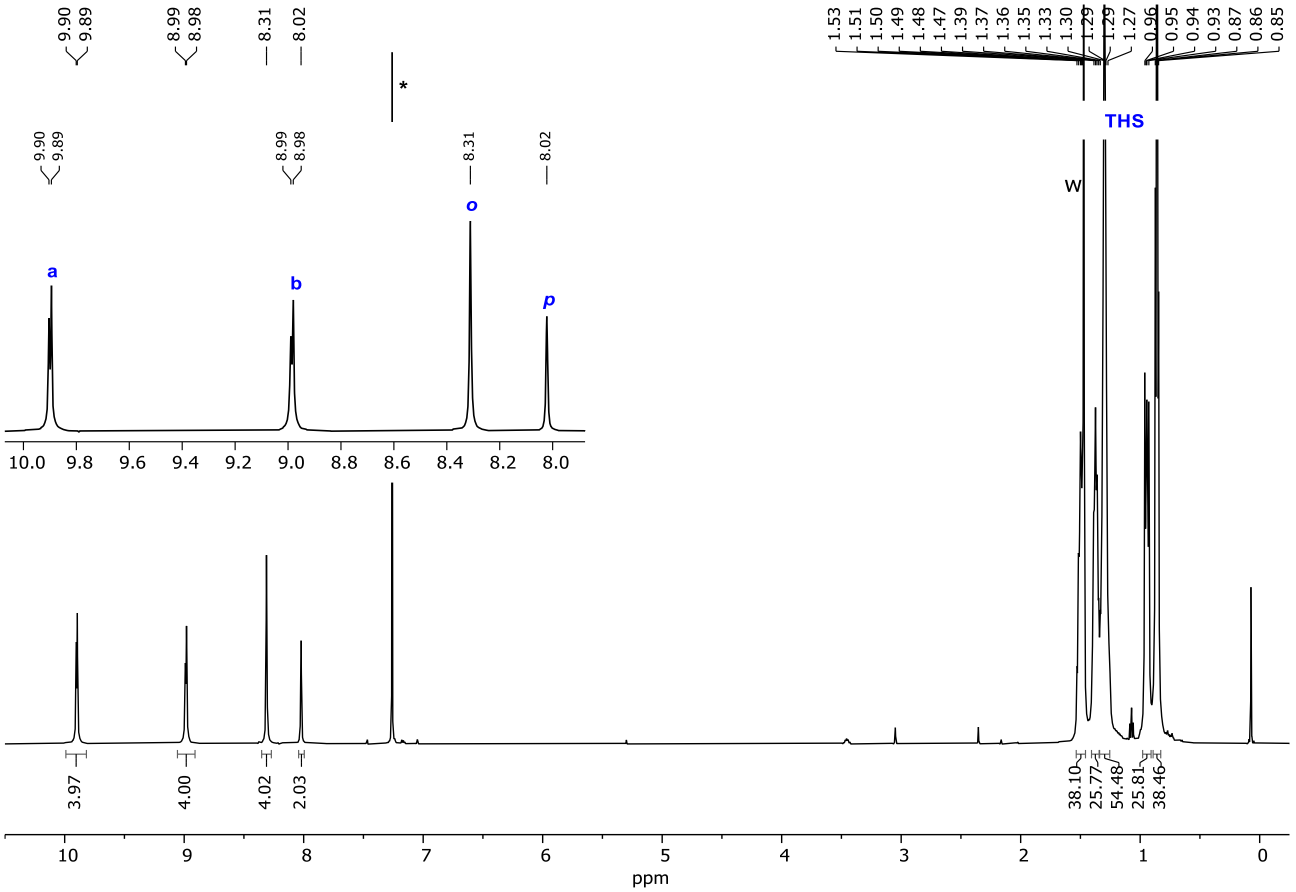

Figure S182. Assigned ^{1}H-NMR spectrum of ***c*-P12**$_{THS}$ (500 MHz, $CDCl_3$, 298 K). Abbreviations: * = $CHCl_3$, w = water.

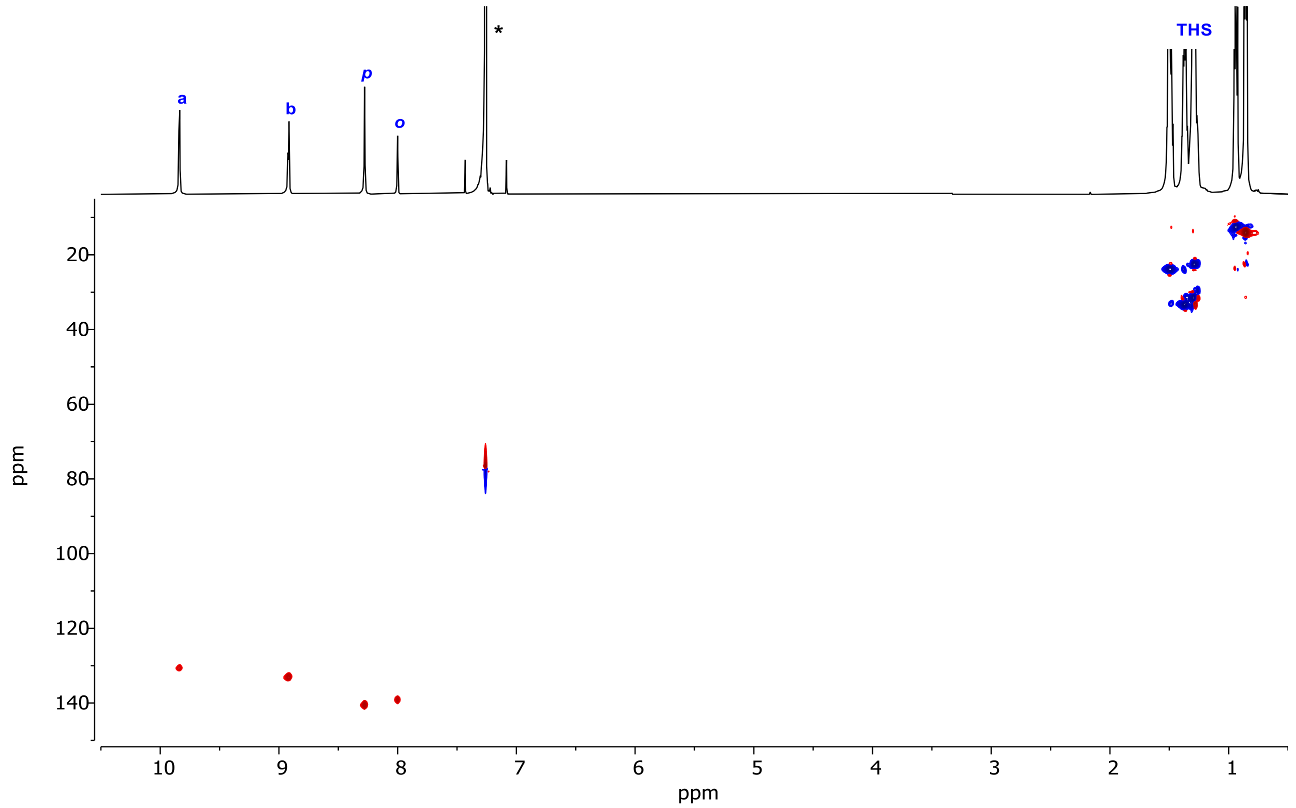

Figure S183. ^{1}H-^{13}C HSQC of ***c*-P12**$_{THS}$ (600 MHz, $CDCl_3$, 298 K). Abbreviations: * = $CHCl_3$, w = water.

Section 19. References

(1) Hoffmann, M.; Wilson, C. J.; Odell, B.; Anderson, H. L. Template-Directed Synthesis of a π-Conjugated Porphyrin Nanoring. *Angew. Chem., Int. Ed.* **2007**, *46*, 3122−3125.

(2) Liu, S.; Kondratuk, D. V.; Rousseaux, S. A. L.; Gil-Ramírez, G.; O'Sullivan, M. C.; Cremers, J.; Claridge, T. D. W.; Anderson, H. L. Caterpillar Track Complexes in Template-Directed Synthesis and Correlated Molecular Motion. *Angew. Chem., Int. Ed.* **2015**, *54*, 5355−5359.

(3) O'Sullivan, M. C.; Sprafke, J. K.; Kondratuk, D. V.; Rinfray, C.; Claridge, T. D. W.; Saywell, A.; Blunt, M. O.; O'Shea, J. N.; Beton, P. H.; Malfois, M.; Anderson, H. L. Vernier Templating and Synthesis of a 12-Porphyrin Nano-Ring. *Nature* **2011**, *469*, 72−75.

(4) Tait, C. E.; Neuhaus, P.; Peeks, M. D.; Anderson, H. L.; Timmel, C. R.; Transient EPR Reveals Triplet State Delocalization in a Series of Cyclic and Linear π-Conjugated Porphyrin Oligomers. *J. Am. Chem. Soc.* **2015**, *137*, 8284–8293.

(5) Kopp, S. M.; Gotfredsen, H.; Deng, J.-R. Deng.; Claridge, T. D. W.; Anderson, H. L. Global Aromaticity in a Partially Fused 8-Porphyrin Nanoring. *J. Am. Chem. Soc.* **2020**, *142*, 19393–19401.

(6) Parkinson, P.; Knappke, C. E. I.; Kamonsutthipaijit, N.; Sirithip, K.; Matichak, J. D.; Anderson, H. L.; Herz, L. M. Ultrafast Energy Transfer in Biomimetic Multistrand Nanorings. *J. Am. Chem. Soc.* **2014**, *136*, 8217–8220.

(7) Tropp, J. Dipolar relaxation and nuclear Overhauser effects in nonrigid molecules: effect of fluctuating internuclear distances. *J. Chem. Phys. 72*, **1980**, 6035–6043.

(8) Koning, T. M. G.; Boelens, R.; Kaptein, R. Calculation of the Nuclear Overhauser Effect and the Determination of Proton-Proton Distances in the Presence of Internal Motions. *J. Magn. Reson.* **1990**, *90*, 111–123.

(9) Kessler, H.; Oschkinat, H.; Griesinger, C.; Bermel, W. *J. Magn. Reson.* **1986**, *70*, 106–133.

(10) Stonehouse, J.; Adell, P.; Keeler, J.; Shaka, A. J. *J. Am. Chem. Soc.* **1994**, *116*, 6037–6038.

(11) Stott, K.; Stonehouse, J.; Keeler, J.; Hwang, T. L.; Shaka, A. J. *J. Am. Chem. Soc.* **1995**, *117*, 4199–4200.

(12) Marcoline, V. M.; Furth, J.; Nayak, S.; Grabe, M.; Macey, R. I. *CPT Pharmacometrics Syst Pharmacol.* **2022**, *11*, 290–301.

(13) M. L. H. Green, L.-L. Wong. Relationship between Intramolecular Chemical Exchange and NMR-Observed Rate Constants. *Organometallics* **1992**, *11*, 2660–2668.

(14) M. D. Peeks, T. D. W. Claridge, H. L. Anderson. Aromatic and antiaromatic ring currents in a molecular nanoring. *Nature* **2017**, *541*, 200–203.

(15) Prestegard, J. H; Bougault, C. M.; Koshore, A. I. Residual Dipolar Couplings in Structure Determination of Biomolecues. *Chem. Rev.* **2004**, *104*, 3519–3540

(16) Chiliveri, S. C.; Robertson, A. J.; Shen, Y.; Torchia, D. A.; Bax, A. Advances in NMR Spectroscopy of Weakly Aligned Biomolecular Systems. *Chem. Rev.* **2022**, *122*, 9307–9330

(17) Zweckstetter, M. NMR: prediction of molecular alignment from structure using the PALES software. *Nat. Protoc.* **2008**, *3*, 679–690.

(18) Lisicki, M. A.; Mishra, P. K.; Bothner-By, A. A.; Lindsey, J. S. *J. Phys. Chem.* **1988**, *92*, 3400–3403.

(19) Pracht, P.; Bohle, F.; Grimme, S. Automated exploration of the low-energy chemical space with fast quantum chemical methods. *Phys. Chem. Chem. Phys.* **2020**, *22*, 7169–7192.

(20) Bannwarth, C.; Ehlert, S.; Grimme, S. GFN2-xTB—An Accurate and Broadly Parametrized Self-Consistent Tight-Binding Quantum Chemical Method with Multipole Electrostatics and Density-Dependent Dispersion Contributions. *J. Chem. Theory Comput.* **2019**, *15*, 1652−1671.

(21) Neese, F. Software update: The ORCA program system—Version 5.0. *WIREs Comput Mol Sci.* **2022**, 12, e1606.

(22) Wang, J.; Wolf, R. M.; Caldwell, J. W.; Kollman, P. A.; Case, D. A. Development and testing of a general amber force field. *J. Comput. Chem.* **2005**, *25*, 1157–1174.

(23) Gotfredsen, H.; Deng, J.-R. Van Raden, J. M. Righetto, M.; Hergenhahn, J.; Clarke, M.; Bellamy-Carter, A.; Hart, J.; O'Shea, J.; Claridge, T. D. W.; Duarte, F.; Saywell, A.; Herz, L. M.; Anderson, H. L. Bending a photonic wire into a ring. *Nat. Chem.* **2022**, *14*, 1436–1442.

(24) Abraham, M. J.; Murtola, T.; Schulz, R.; Páll, S.; Smith, J. C.; Hess, B.; Lindahl, E. GROMACS: High performance molecular simulations through multi-level parallelism from laptops to supercomputers. *SoftwareX* **2015**, *1–2*, 19–25.

(25) Ásgeirsson, V.; Birgisson, B. R.; Bjornsson, R.; Becker, U.; Neese, F.; Riplinger, C.; Jónsson, H. Nudged Elastic Band Method for Molecular Reactions Using Energy-Weighted Springs Combined with Eigenvector Following. *J. Chem. Theory Comput.* **2021**, *17*, 4929−4945.

(26) Wang, L.-P.; Song, C. Geometry optimization made simple with translation and rotation coordinates. *J. Chem. Phys.* **2016**, *144*, 214108.

(27) Steinmetzer, J.; Kupfer, S.; Gräfe, S. pysisyphus: Exploring potential energy surfaces in ground and excited states. *Int. J. Quantum Chem.* **2021**, *121*, e26390.

(28) Lodewyk, M. W.; Siebert, M. R.; Tantillo, D. J. Computational Prediction of ^{1}H and ^{13}C Chemical Shifts: A Useful Tool for Natural Product, Mechanistic, and Synthetic Organic Chemistry. *Chem. Rev.* **2012**, *112*, 1839–1862.

(29) Bussi, G.; Donadio, D.; Parrinello, M. Canonical sampling through velocity rescaling. *J. Chem. Phys.* **2007**, *126*, 014101.

(30) Parrinello, M.; Rahman, A. Polymorphic transitions in single crystals: A new molecular dynamics method. *J. Appl. Phys.* **1981**, *52*, 7182–7190.

(31) Cieplak, P.; Caldwell, J.; Kollman, P. Molecular mechanical models for organic and biological systems going beyond the atom centered two body additive approximation: aqueous solution free energies of methanol and N-methyl acetamide, nucleic acid base, and amide hydrogen bonding and chloroform/water partition coefficients of the nucleic acid bases. *J. Comput. Chem.* **2001**, *22*, 1048–1057.

(32) Essmann, U.; Perera, L.; Berkowitz, M. L.; Darden, T.; Lee, H.; Pedersen, L. G. A smooth particle mesh Ewald method. *J. Chem. Phys.* **1995**, *103*, 8577–8593.

(33) Hess, B.; Bekker, H.; Berendsen, H. J. C.; Fraaije, J. G. E. M. LINCS: A linear constraint solver for molecular simulations. *J. Comput. Chem.* **1997**, *18*, 1463–1472.

(34) Peeks, M. D.; Neuhaus, P.; Anderson, H. L. Experimental and computational evaluation of the barrier to torsional rotation in a butadiyne-linked porphyrin dimer. *Phys. Chem. Chem. Phys.* **2016**, *18*, 5264–5274.

(35) Hutin, M.; Sprafke, J. K.; Odell, B.; Anderson, H. L.; Claridge, T. D. W. A Discrete Three-Layer Stack Aggregate of a Linear Porphyrin Tetramer: Solution-Phase Structure Elucidation by NMR and X-ray Scattering. *J. Am. Chem. Soc.* **2013**, *135*, 12798–12807.

(36) Würth, C.; Grabolle, M.; Pauli, J.; Spieles, M; Resch-Genger, U. Relative and absolute determination of fluorescence quantum yields of transparent samples. *Nat. Protoc.* **2013**, *8*, 1535–1550.

(37) Minotto, A.; Bulut, I.; Rapidis, A. G.; Carnicella, G.; Patrini, M.; Lunedei, E.; Anderson, H. L.; Cacialli, F. Towards efficient near-infrared fluorescent organic light-emitting diodes. *Light Sci. Appl.* **2021**, *10*, 18.
